# Synthesis and Antiviral Properties of Camphor-Derived Iminothiazolidine-4-Ones and 2,3-Dihydrothiazoles

**DOI:** 10.3390/molecules27154761

**Published:** 2022-07-25

**Authors:** Vladislav V. Oreshko, Kseniya S. Kovaleva, Ekaterina D. Mordvinova, Olga I. Yarovaya, Yuri V. Gatilov, Dmitry N. Shcherbakov, Nikolai I. Bormotov, Olga A. Serova, Larisa N. Shishkina, Nariman F. Salakhutdinov

**Affiliations:** 1 Department of Medicinal Chemistry, N. N. Vorozhtsov Novosibirsk Institute of Organic Chemistry, Siberian Branch of the Russian Academy of Sciences, Lavrentiev Ave. 9, 630090 Novosibirsk, Russia; vladoreshko28121999@gmail.com (V.V.O.); kseniya.kovaleva3103@yandex.ru (K.S.K.); gatilov@nioch.nsc.ru (Y.V.G.); anvar@nioch.nsc.ru (N.F.S.); 2 Zelman Institute for Medicine and Psychology, Novosibirsk State University, Pirogova St., 1, 630090 Novosibirsk, Russia; mordvinova97@mail.ru; 3 State Research Center of Virology and Biotechnology VECTOR, Rospotrebnadzor, 630559 Koltsovo, Russia; dnshcherbakov@gmail.com (D.N.S.); bormotov_ni@vector.nsc.ru (N.I.B.); serova.olga.a@yandex.ru (O.A.S.); shish@vector.nsc.ru (L.N.S.)

**Keywords:** monoterpenoids, monoterpenes, vaccinia virus, Marburg virus, heterocyclic compounds

## Abstract

A set of heterocyclic products was synthesized from natural (+)-camphor and semi-synthetic (−)-camphor. Then, 2-Imino-4-thiazolidinones and 2,3-dihydrothiazoles were obtained using a three-step procedure. For the synthesized compounds, their antiviral activity against the vaccinia virus and Marburg virus was studied. New promising agents active against both viruses were found among the tested compounds.

## 1. Introduction

The spread of viral infections poses a significant threat to healthcare and the economy and can lead to unpredictable consequences. Over the past decades, researchers have made significant progress in the development of antiviral agents certified for the treatment of human immunodeficiency virus, herpes virus, hepatitis virus, and respiratory viruses. Despite this, chemotherapy is still practically powerless against a number of infections caused by viruses. Lack of specific therapy can lead to a significant number of deaths and long-term consequences caused by complications from viral diseases. The 21st century is the century of widespread viral infections. Over the past 20 years, humanity has faced new diseases that were caused by previously unknown coronaviruses, such as SARS-CoV and MERS-CoV, varieties of bird flu, H1N1 swine flu, and the Ebola and Zika viruses. The year 2020 will be remembered in world history for the pandemic of a new coronavirus infection, COVID-19, caused by SARS-CoV-2. The complication of the epidemiological situation puts forward strict requirements for the rapid search for substances against a wide range of viruses. A serious problem is the ability of viruses to mutate rapidly, developing resistance to the few available antiviral drugs. Therefore, the development of new effective antiviral drugs with the widest possible spectrum of action is one of the primary tasks of medicinal chemistry.

One of the promising ways, in the development of new pharmacologically significant agents, is the use of natural compounds as starting substances [1,2]. Terpenes and terpenoids can serve as an example of promising starting molecules for medicinal chemistry [3]. Various terpenoids exhibit anti-inflammatory, antipyretic, and anti-allergic activity, as well as many other valuable properties. These compounds are found in the essential oils of plants, which allows them to be obtained in quantities sufficient for subsequent chemical studies. For example, camphor has been used in medicine for a very long time. Camphor is widely distributed in nature and is a constituent of many essential oils. D-(+)-camphor is obtained from camphor laurel wood (*Cinnamonum camphora*), while semi-synthetic L-(−)-camphor from fir oil. The terpene alcohols borneol and isoborneol, with a huge number of valuable pharmacological properties [4,5,6], have an identical 1,7,7-trimethylbicyclo[2.2.1]heptane backbone.

**Figure 1:**
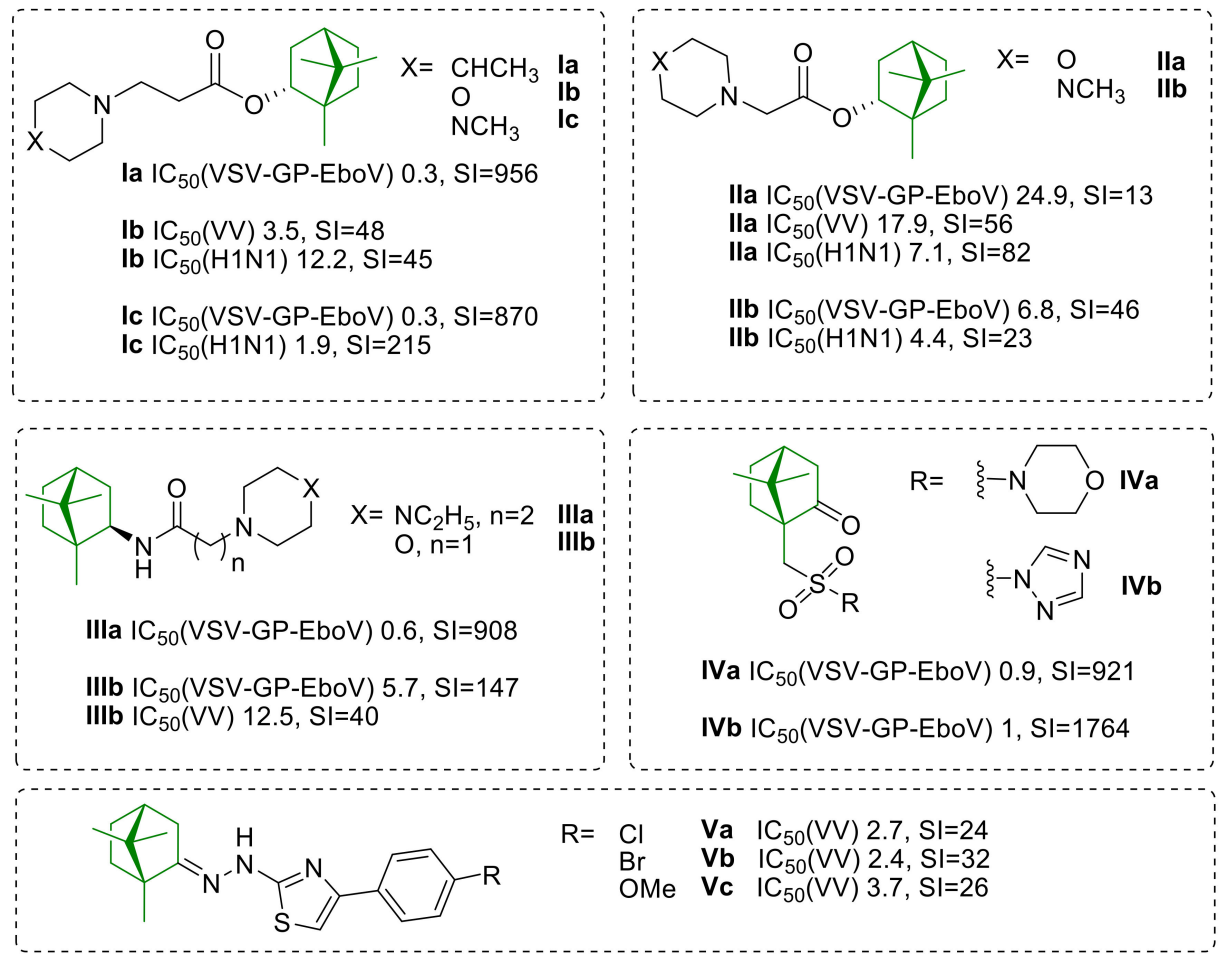
Compounds with 1,7,7-trimethylbicyclo[2.2.1]heptane backbone and their antiviral properties. All IC_50_ values are given in μM. Anti-influenza activity is described in [16], anti-orthopoxvirus—[14,17], anti-filovirus—[10,11,12].

Many different classes of camphor-based derivatives have been synthesized with a wide range of antiviral properties. We have previously shown that derivatives with a 1,7,7-trimethylbicyclo[2.2.1]heptane backbone exhibit a wide spectrum of antiviral activity against influenza [7,8], hantaviruses [9], filoviruses [10,11,12], orthopoxviruses [13,14] and SARS-CoV-2 [15]. Some compounds, with a heterocyclic fragment synthesized previously on the basis of camphor and borneol, which have shown significant antiviral properties, are presented in Figure 1.

The structure of previously identified leader compounds contains both a bicyclic bornane skeleton and a nitrogen-containing cyclic structural block. Thus, the design of the target molecules is promising, using a similar principle: a combination of a terpene block and a pharmacophoric heterocyclic core. In this work, the starting terpene compound is camphor, and 2,3-dihydrothiazoles and thiazolidinones are used as heterocyclic pharmacophores. Molecules constructed in this way have a high potential for the study of their antiviral properties and can become promising candidates for creating new medicinal agents on their basis.

## 2. Results and Discussion

### 2.1. Chemistry

**Scheme 1:**
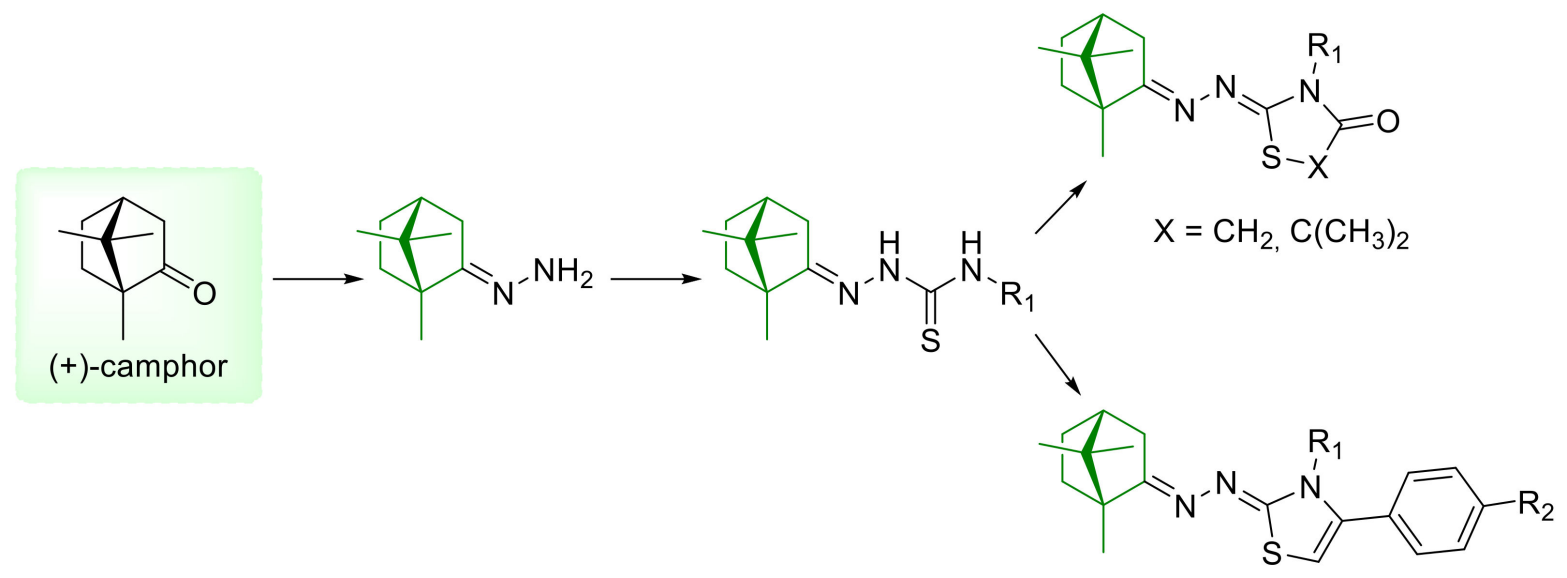
Design of thiazolidin-4-ones and 2,3-dihydrothiazoles with a camphor moiety.

To obtain the target compounds, a synthetic Scheme 1 was proposed, according to which, at the first stage, corresponding hydrazone is obtained from natural monoterpenoid (+)-camphor, and then a set of N-substituted thiosemicarbazones is obtained from it. The thiosemicarbazone group is a convenient platform for further transformations. In this work, we studied the interaction with α-halocarbonyl compounds, which allowed us to obtain derivatives with nitrogen- and sulfur-containing heterocyclic blocks.

The (+)-camphor isomer was reacted with hydrazine hydrate in the presence of glacial acetic acid to obtain hydrazone **1**. The reaction was carried out according to the well-known method [18], by refluxing in ethanol for 3 h, after which camphor hydrazone was isolated with an quantitative yield of almost 98%.

N-substituted thiosemicarbazones **2a-d** were obtained by reacting (+)-camphor hydrazone **1** with various isothiocyanates. Convenient conditions, not requiring post-processing or further purification, were chosen to isolate the target products in good yields. Hexane was used as a solvent, since the starting reagents are easily dissolved in it, while the target thiosemicarbazones precipitate after the completion of the reaction.

The thiosemicarbazone group is an excellent starting platform for the synthesis of nitrogen- and sulfur-containing products of various structures. The interaction of thiosemicarbazones with α-halocarbonyl compounds leads to the selective formation of a heterocyclic core.

Synthesized thiosemicarbazones **2a-d** were reacted with ethyl bromoacetate in the presence of Et_3_N at room temperature. As a result of this process, a series of 2-iminothiazolidin-4-ones **3a-d** was obtained, which were further purified using silica gel column chromatography.

Previously, camphor 2-imino-thiazolidin-4-one, unsubstituted at the N3 position, was obtained, and then its modifications were carried out [14]. In this work, we proposed an alternative scheme, according to which substituted thiosemicarbazones were obtained first, and then a thiazolidin-4-one ring was formed, with a substituent already present in the N3 position. This allowed us to expand the set of derivatives under study.

**Scheme 2:**
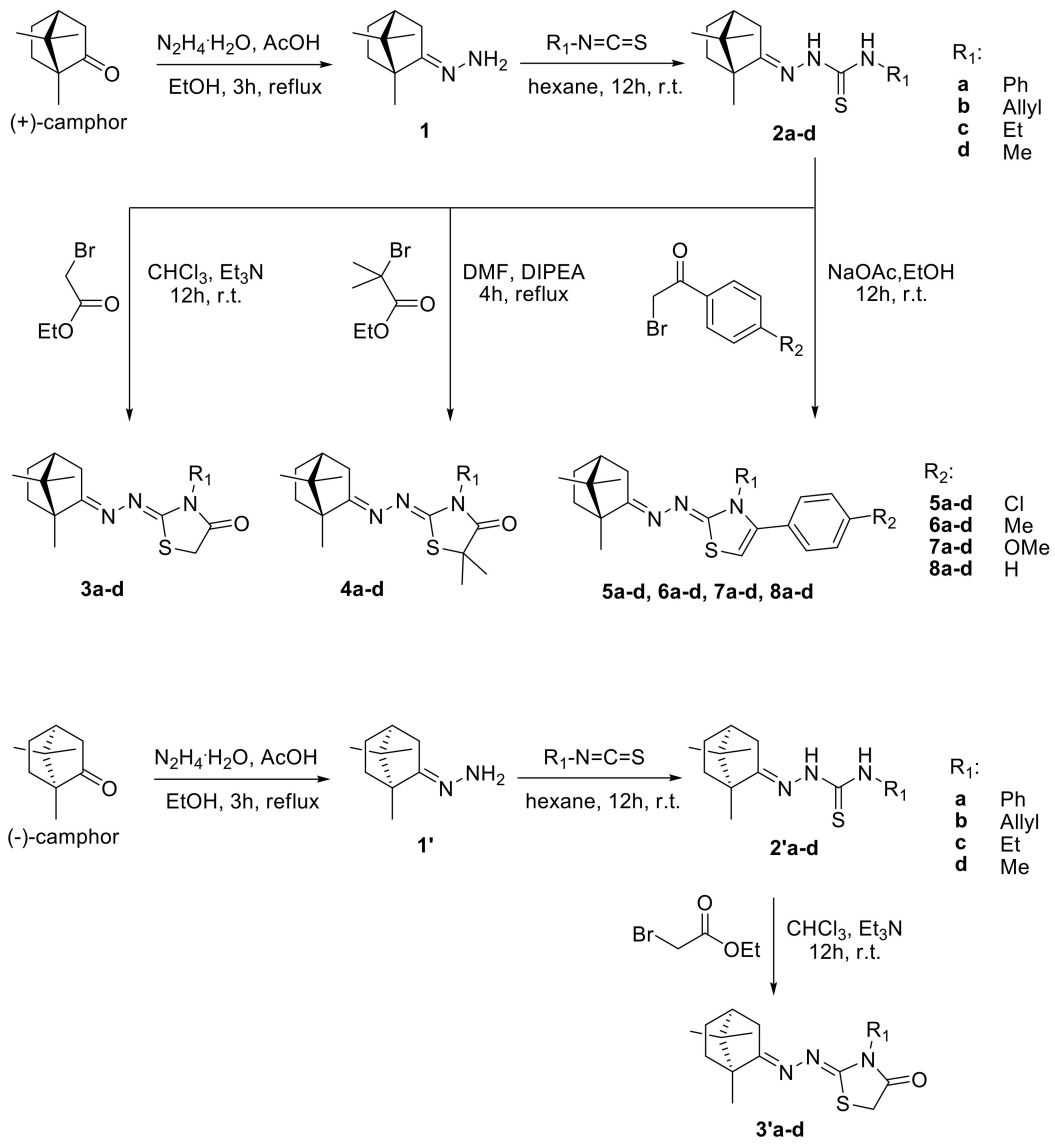
Synthesis of nitrogen- and sulfur-containing camphor derivatives.

It was not possible to obtain 5,5-dimethyl-2-iminothiazolidinones **4a-d** from thiosemicarbazones affected by ethyl 2-bromoisobutyrate under similar conditions, due to steric hindrance created by the gemdimethyl group of ethyl 2-bromoisobutyrate. We also failed to achieve the desired result when the reaction mixture was heated and when using an excess of ethyl 2-bromoisobutyrate. Complete conversion of thiosemicarbazones **2a-d** was achieved by using a DIPEA (N,N-diisopropylethylamine) base in DMF for 4 h. The target products can be purified and isolated individually using column chromatography (Scheme 2).

**Figure 2:**
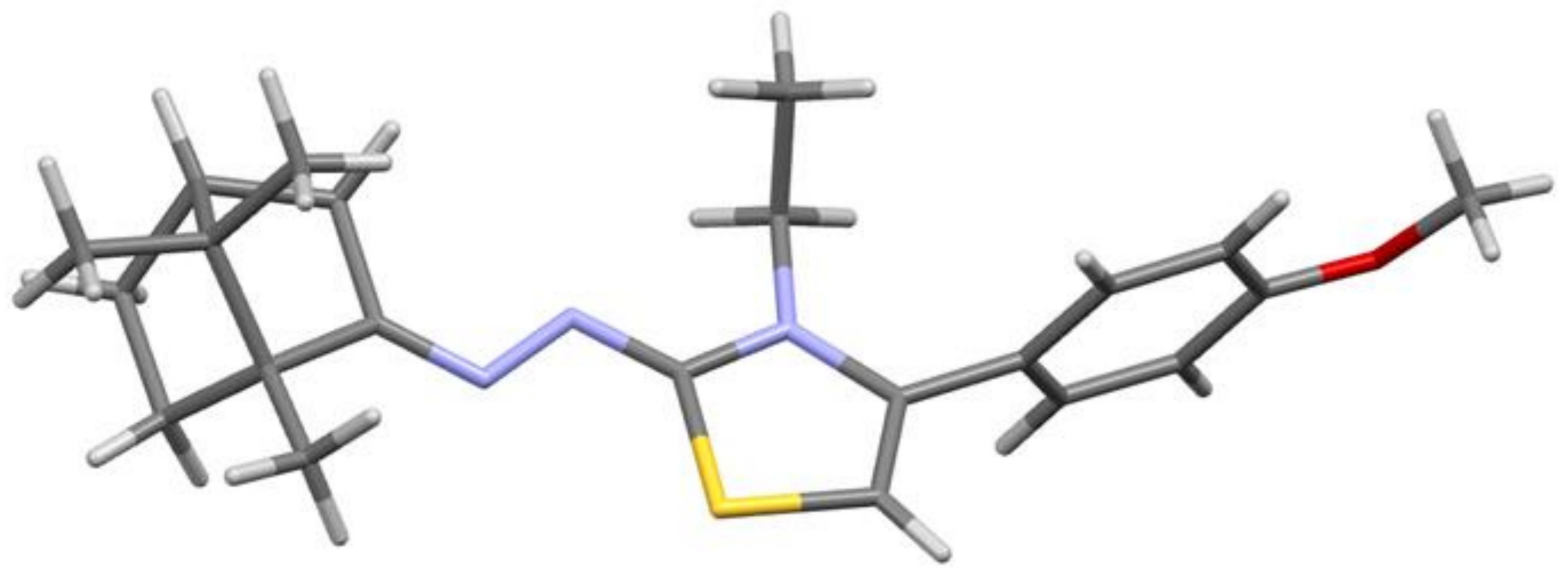
Structure of compound **7c** according to X-ray diffraction data (CCDC 2181023). Crystallographic data for **7c**: C_22_H_29_N_3_OS, M 383.54, orthorhombic, P2_1_2_1_2_1_, a 9.7454(4), b 12.4168(6), c 17.905(1), Å, V 2166.6(2)Å^3^, Z 4, D_calcd_ 1.176 g·cm^–3^, F(000) 824, colorless, (0.61 × 0.51 × 0.42) mm^3^, μ(Mo-Kα) 0.165 mm^–1^, transmission 0.8666–0.9143, (θ 2.00–26.09°, completeness 99.93%), 14,261 measured reflections in index range −11 ≤ h ≤ 9, −15 ≤ k ≤ 15, −22 ≤ l ≤ 22, 4262 independent (R_int_ 0.0344), 244 parameters (259 restraints), R_1_ 0.0673 (for 3664 observed I > 2σ(I)), wR_2_ = 0.201 (all data), GOOF 1.079, Flack parameter 0.01(3), largest diff. peak and hole 0.591 and −0.396 e.Å*^−^*^3^, respectively.

To obtain 2,3-dihydrothiazoles **5-8**, 2-bromoacetophenones with various substituents in the 4-position of the aromatic ring were used. The reaction was carried out in an EtOH/NaOAc system at room temperature, after which the solvent was evaporated, and the reaction mixture was washed with water and extracted with CHCl_3_. After processing, most of the target products were isolated in excellent yields >85% and did not require further purification, except for compounds **5a**, **7a**, and **8d**. Dihydrothiazoles **5a**, **7a**, and **8d** were further purified by column chromatography. As a result, a set of 16 heterocyclic derivatives was obtained. The structures of all obtained compounds were confirmed using ^1^H and ^13^C NMR, IR, and high-resolution mass spectroscopy. For compound **7c**, a single crystal was grown, and the structure was additionally confirmed by X-ray diffraction analysis (Figure 2).

The study of biological properties of optical isomers is of great importance for medicinal chemistry because the binding of different diastereomers to the target can vary and depends on the absolute configuration of asymmetric centers in the target [19,20]. As it is shown in Scheme 2, some of the transformations were also performed on the (−)-camphor isomer. (−)-Camphor hydrazone **1*′***, a set of thiosemicarbazones **2*′*a-d**, and thiazolidinones **3*′*a-d** were obtained. The reactions for the (+) and (−) isomers were carried out under identical conditions.

For convenience, all compound numbers, the types of camphor and nitrogen-containing fragments, and the final yields are summarized in Table 1.

Hydrazones of unsymmetrical ketones are known to possess E/Z isomerism related with the C=N bond. Camphor hydrazone exists as a single E-isomer, probably due to its steric hindrance. All derivatives derived from it also have the E-configuration of the C=N bond.

As a result of synthetic transformations, we obtained substances of four types: compounds **2a-d** and **2′a-d**-N-substituted thiosemicarbazones based on (+)- and (−)-camphor; 2-iminothiazolidin-4-ones **3a-d** and **3′a-d**; 5,5-dimethyl-2-iminothiazolidinones **4a-d**, and 2,3-dihydrothiazoles **5a-d-8a-d**. N-phenyl **2a** and N-methyl **2d** thiosemicarbazones of (+)-camphor were previously described in the literature [21,22]. (+)-Camphor 2-imino-4-thiazolidinones **3b** and **3d** have also been described [14,21]. N-methylthiosemicarbazone **2d** was obtained in a different way, by heating (+)-camphor with N-methylthiosemicarbazide, while compounds **3b** and **3d** were obtained by the alkylation of N-unsubstituted camphor 2-imino-4-thiazolidinone. Other compounds are new and have not been previously described in the literature.

### 2.2. Antiviral Activity Study

#### 2.2.1. Vaccinia Virus

Variola virus belongs to the family *Poxviridae*, genus *Orthopoxvirus*, and is a highly contagious viral infection transmitted by airborne droplets. The disease is believed to have been eradicated since 1980, following a successful worldwide vaccination program supervised by the World Health Organization (WHO). Mandatory smallpox vaccination was abolished [23]. However, in this regard, the population is increasingly becoming susceptible to related orthopoxvirus diseases. There have been documented cases of human infection with cowpox, camelpox, and monkeypox viruses that were transmitted from animals to humans and have posed a biological threat. Since the beginning of 2022, an increasing number of monkeypox cases have been reported [24,25]. There is also no guarantee that there will be no new outbreaks of smallpox in the future, since orthopoxviruses can survive for decades in nature at low temperatures [26]. To date, several drug agents with different mechanisms of action have been approved for the treatment of orthopoxvirus infections. Among them are the nucleotide analogue Cidofovir and its lipid conjugate Brincidofovir [27,28], as well as the low-molecular drug Tecovirimat [29]. Despite this, the search for new low-molecular compounds with anti-smallpox activity is still an urgent task. The primary screening of camphor derivatives synthesized in this work against the vaccinia virus (*Vaccinia virus*, VV) was carried out by the State Research Center of Virology and Biotechnology “Vector”, Koltsovo. The test results are shown in Table 2.

The key characteristic of the antiviral properties of the studied compounds is the selectivity index (SI), defined as the ratio of the concentration of a substance that causes the death of 50% of uninfected cells (CC_50_) to the concentration that is necessary to achieve a 50% level of inhibition of viral reproduction using this substance (IC_50_). It is generally accepted that compounds with a selectivity index greater than 8 exhibit activity [30]. The study of antiviral activity against the VV was carried out using Vero cells. As can be seen from the data presented in Table 2, N-substituted thiosemicarbazones **2b**, **2d**, and **2′d** showed the least cytotoxicity, however, the activity of these agents was low. Substances **2a** and **2′a** showed comparable activity in the lower micromolar range, however, they were quite toxic on the studied cells. 2-Iminothiazolidin-4-ones **3a-d** and **3′a-d** showed low toxicity in the experiment; however, they showed almost no activity against the VV. Among 5,5-dimethyl-2-iminothiazolidinones **4a-d**, agent **4d**, containing a methyl substituent at the nitrogen atom of the heterocyclic fragment, exhibited the highest activity and low toxicity; the selectivity index for this substance was 24, which exceeded that of the reference drug. The 2,3-Dihydrothiazoles **5b**, **5d**, **6b**, **6d**, and **8b-d** showed the best activity against the vaccinia virus with IC_50_ from 1.5 to 5.1 μM, and agent **5d** was the least toxic of these substances. For the latter compound, the selectivity index was 54, which was significantly higher than that of the reference drug. On the whole, it can be concluded that the presence of an allyl or methyl substituent at the nitrogen atom of the heterocyclic fragment is most promising for the manifestation of activity. In addition, comparing the substituents in the aromatic ring of 2,3-dihydrothiazoles, we can say that *p*-chlorophenyl substituents in the 4 position of the heterocycle are the most promising.

#### 2.2.2. Marburg Virus

The Marburg virus belongs to the filovirus family. The family of viruses causes severe hemorrhagic fevers in humans and non-human primates, which are characterized by high viremia and multiple organ failure with a mortality rate of up to 90%. To date, there are no formally approved vaccines or standard therapies, although some experimental vaccines and treatments have shown promising results in non-human primates [31]. Filoviruses, in particular, the Marburg virus, contain on their surface only one glycoprotein (GP) that ensures pathogen penetration into the cell. This protein is a suitable target, since it is absent in mammalian cells, as is RNA-dependent RNA polymerase. The synthesized compounds were tested against the Marburg pseudovirus at the State Research Center of Virology and Biotechnology “Vector”, and the results are presented in Table 3.

Sertraline was used as a reference substance in this work; this substance is capable of inhibiting the penetration of various filoviruses, presumably at the stage of entry [32]. The analysis of the biological tests on the activity of compounds against Marburg virus entry shows that the toxicity of compounds on the HEK293FT cell line does not always correlate with the toxicity of compounds on the Vero cell line. For example, thiosemicarbazones **2a** and **2′a** on the Vero cell line were significantly more toxic than in this experiment. Agents **3a**, **4b**, and **5a** showed the least toxicity in the pseudovirus experiment. Agents **2′a**, **2′b**, **2′c**, **5d**, **6b**, **7c**, and **7d** showed significant activity against Marburg pseudoviruses. The above agents inhibited the virus at doses ranging from 3.8 to 13.9 μM. Agent **2′a** had the highest index of selectivity. In general, we can conclude that moderate activity is found in thiosemicarbazones synthesized on (−)-camphor; agents **5d** and **4d**, which showed activity against the vaccine virus, are also of interest as inhibitors of filovirus entry. Since these viruses belong not only to different species but also have significant differences in the surface proteins and mechanisms of virus entry into the cell, it seems promising to study the mechanism of action of these substances.

## 3. Materials and Methods

### 3.1. Chemistry

#### 3.1.1. General Information

The ^1^H and ^13^C NMR spectra were recorded on Bruker spectrometers (Bruker BioSpin GmbH, Ettlingen, Germany), including an AV-300 instrument at 300.13 MHz (^1^H) and 75.47 MHz (^13^C), an AV-400 instrument at 400.13 MHz (^1^H) and 100.61 MHz (^13^C), and a DRX-500 instrument at 500.13 MHz (^1^H) and 125.76 MHz (^13^C) in CDCl_3_; chemical shifts δ were reported in ppm relative to residual CHCl_3_ [d(CHCl_3_) 7.24, d(CDCl_3_) 76.9 ppm], *J* in Hz. The atom numbering in the compounds is given for the assignment of the NMR spectra signals and does not match with the atom numbering in the nomenclature name. High-resolution mass spectra were recorded on DFS ThermoScientific and Agilent 7200 Accurate Mass Q-TOF spectrometers (Agilent Tech., Santa Clara, CA, USA), in full-scan mode in the range *m*/*z* 0–500, with electron impact ionization 70 eV at direct sample input. Chromato-mass spectra were recorded on an Agilent 7890 A gas chromatograph (Agilent Tech., Santa Clara, CA, USA), with an Agilent 5975C quadrupole mass spectrometer as a detector, HP-5MS 30,000 × 0.25 mm quartz column, and carrier gas—He. IR spectra were recorded on a Vector22 (KBr) spectrometer. Specific rotation was determined on a polAAr 3005 spectrometer; solution concentrations are given in g/100 mL. Melting points were determined on a Termosystem FP 900 instrument from Mettler Toledo. The X-ray diffraction experiment for crystals of 7c was carried out at 299(2)° on a Bruker KAPPA APEX II diffractometer (graphite-monochromated Mo Kα radiation). Reflection intensities were corrected for absorption by SADABS program. The structure was solved by direct methods using the using SHELXT 2014/5 program [33] and refined by anisotropic (isotropic for all H atoms) with full-matrix least squares method against *F^2^* of all reflections by SHELXL2018/3 [34]. The positions of the hydrogen were calculated geometrically and refined in riding model. Crystallographic data for 7c have been deposited at the Cambridge Crystallographic Data Centre as supplementary publication no. CCDC 2181023. Copy of the data can be obtained free of charge, on application to CCDC, 12 Union Road, Cambridge CB21EZ, UK (fax: +44 122 3336033 or e-mail: deposit@ccdc.cam.ac.uk; internet: www.ccdc.cam.ac.uk, accessed on 1 January 2020). Separation and isolation of the reaction products was carried out using silica gel column chromatography (60–200 μ, Masherey-Nagel). Thin-layer chromatography was performed on Merck silica gel (60 F254) TLC plates and visualized under 254 nm UV lamp. All reagents and other chemicals were purchased from Acros Organics (Geel, Belgium), Merck (Darmstadt, Germany), ABCR (Karlsruhe, Germany), and TCI Europe (Zwijndrecht, Belgium) and used without further purification.

#### 3.1.2. General Procedure for Camphor Thiosemicarbazones Synthesis **2a–d**, **2′a–d**

Hydrazones of (+) or (−)-camphor (0.5 g, 3 mmol) and the corresponding isothiocyanates were mixed in a round bottom flask in hexane (25 mL) in a molar ratio of 1:1. The reaction mixture was stirred overnight at room temperature. The precipitated white crystals were filtered off and washed with hexane (2 × 10 mL).

^1^H NMR and ^13^C NMR spectra of compounds **2a-8d**, High Resolution Mass Spectra of Compounds **2a-8d** are shown in Supplementary Materials.

(*E*)-N-phenyl-2-((1*R*,4*R*)-1,7,7-trimethylbicyclo[2.2.1]heptan-2-ylidene)hydrazinecarbothioamide (**2a**), white crystalline substance, yield 90% (0.81 g). ^1^H-NMR (CDCl_3_): δ_H_ 0.75 (3H, s, Me-9), 0.94 (3H, s, Me-8), 1.02 (3H, s, Me-10), 1.19–1.25 (1H, m, H-3), 1.37–1.43 (1H, m, H-2), 1.73–1.80 (1H, m, H-2), 1.84–1.94 (2H, m, H-5, H-3), 2.04 (1H, t, *J* = 3.7 Hz, H-4), 2.42 (1H, dt, *J* = 16.8 Hz, *J* = 3.6 Hz, H-5), 7.20 (1H, t, *J* = 7.2 Hz, H-15), 7.36 (2H, t, *J* = 7.9 Hz, H-14,16), 7.63 (2H, d, *J* = 8.1 Hz, H-13,17), 8.27 (1H, s, NH 19), 9.23 (1H, s, NH 18). ^13^C-NMR (CDCl_3_): δ_c_ 10.99 (Me-10), 18.45 and 19.36 (Me-8 and Me-9), 27.02 (C-3), 32.35 (C-2), 33.76 (C-5), 43.80 (C-4), 48.11 (C-7), 52.77 (C-1), 124.04 (C-13, C-17), 125.75 (C-15), 128.56 (C-14, C-16), 137.85 (C-12), 165.85 (C-6), 175.69 (C-11). HRMS (EI): *m/z* calcd for C_17_H_23_N_3_S: 301.1607; found 301.1605. [α]^D^_25_ *−*45.45 (c = 1.21 in CHCl_3_). IR (KBr, cm^−1^): ν 3600–3200 (NH), 1540 (C=N), 1186 (C=S). M.p. 182.1–182.8 °C.

(*E*)-N-allyl-2-((1*R*,4*R*)-1,7,7-trimethylbicyclo[2.2.1]heptan-2-ylidene)hydrazinecarbothioamide (**2b**), white crystalline substance, yield 60% (0.48 g).^1^H-NMR (CDCl_3_): δ_H_ 0.70 (3H, s, Me-9), 0.90 (3H, s, Me-8), 0.95 (3H, s, Me-10), 1.14–1.21 (1H, m, H-3), 1.30–1.37 (1H, m, H-2), 1.68–1.75 (1H, m, H-2), 1.78–1.89 (2H, m, H-5, H-3), 1.99 (1H, t, *J* = 3.7 Hz, H-4), 2.34 (1H, dt, *J* = 16.8 Hz, *J* = 3.7 Hz, H-5), 4.25–4.37 (2H, m, H-12), 5.14–5.26 (2H, m, H-14), 5.87–5.98 (1H, m, H-13), 7.49 (1H, s, NH 16), 8.16 (1H, s, NH 15). ^13^C-NMR (CDCl_3_): δ_c_ 10.95 (Me-10), 18.42 and 19.30 (Me-8 and Me-9), 27.00 (C-3), 32.34 (C-2), 33.59 (C-5), 43.76 (C-4), 46.55 (C-12), 47.98 (C-7), 52.59 (C-1), 116.50 (C-14), 133.48 (C-13), 165.38 (C-6), 177.49 (C-11). HRMS (EI): *m*/*z* calcd for C_14_H_22_N_3_S: 264.1529; found [M-H]^+^ 264.1533. [α]^D^_25_ *−*37.07 (c = 1.16 in CHCl_3_). IR (KBr, cm^−1^): ν 3600–3100 (NH), 2954 (C-H), 1537 (C=N), 1494 (CH_3_), 1213 (C=S). M.p. 111.5–112.4 °C.

(*E*)-N-ethyl-2-((1*R*,4*R*)-1,7,7-trimethylbicyclo[2.2.1]heptan-2-ylidene)hydrazinecarbothioamide (**2c**), white crystalline substance, yield 50% (0.38 g). ^1^H-NMR (CDCl_3_): δ_H_ 0.70 (3H, s, Me-9), 0.90 (3H, s, Me-8), 0.96 (3H, s, Me-10), 1.14–1.21 (1H, m, H-3), 1.24 (3H, t, *J* = 7.1 Hz, H-13), 1.30–1.37 (1H, m, H-2), 1.67–1.76 (1H, m, H-2), 1.80–1.88 (2H, m, H-5, H-3), 1.99 (1H, t, *J* = 3.7 Hz, H-4), 2.33 (1H, dt, *J* = 16.8 Hz, *J* = 3.7 Hz, H-5), 3.64–3.74 (2H, m, H-12), 7.37 (1H, s, NH 15), 8.07 (1H, s, NH 14). ^13^C-NMR (CDCl_3_): δ_c_ 10.96 (Me-10), 14.39 (C-13), 18.43 and 19.30 (Me-8 and Me-9), 27.01 (C-3), 32.36 (C-2), 33.57 (C-5), 39.08 (C-12), 43.75 (C-4), 47.97 (C-7), 52.54 (C-1), 165.03 (C-6), 177.10 (C-11). HRMS (EI): *m*/*z* calcd for C_13_H_23_N_3_S: 253.1607; found 253.1604. [α]^D^_25_ *−*54.97 (c = 1.91 in CHCl_3_). IR (KBr, cm^−1^): ν 3600–3100 (NH), 2958 (C-H), 1533 (C=N), 1494 (CH_3_), 1222 (C=S). M.p. 148.2–148.4 °C.

(*E*)-N-methyl-2-((1*R*,4*R*)-1,7,7-trimethylbicyclo[2.2.1]heptan-2-ylidene)hydrazinecarbothioamide (**2d**), white crystalline substance, yield 60% (0.43 g). ^1^H-NMR (CDCl_3_): δ_H_ 0.69 (3H, s, Me-9), 0.90 (3H, s, Me-8), 0.95 (3H, s, Me-10), 1.14–1.21 (1H, m, H-3), 1.28–1.35 (1H, m, H-2), 1.67–1.75 (1H, m, H-2), 1.79–1.88 (2H, m, H-5, H-3), 1.99 (1H, t, *J* = 3.7 Hz, H-4), 2.33 (1H, dt, *J* = 16.8 Hz, *J* = 3.7 Hz, H-5), 3.18 (3H, d, *J* = 4.9 Hz, H-12), 7.41 (1H, m, NH 14), 8.14 (1H, s, NH 13). ^13^C-NMR (CDCl_3_): δ_c_ 10.99 (Me-10), 18.42 and 19.28 (Me-8 and Me-9), 27.01 (C-3), 30.91 (C-12), 32.35 (C-2), 33.55 (C-5), 43.75 (C-4), 47.96 (C-7), 52.54 (C-1), 165.14 (C-6), 178.26 (C-11). HRMS (EI): *m*/*z* calcd for C_12_H_21_N_3_S: 239.1451; found 239.1454. [α]^D^_25_ *−*37.74 (c = 1.06 in CHCl_3_). IR (KBr, cm^−1^): ν 3600–3100 (NH), 2966 (C-H), 1540 (C=N), 1498 (CH_3_), 1241 (C=S). M.p. 138.2–139.3 °C.

(*E*)-N-phenyl-2-((1*S*,4*S*)-1,7,7-trimethylbicyclo[2.2.1]heptan-2-ylidene)hydrazinecarbothioamide (**2′a**), white crystalline substance, yield 85% (0.77 g). ^1^H-NMR (CDCl_3_): δ_H_ 0.75 (3H, s, Me-9), 0.94 (3H, s, Me-8), 1.02 (3H, s, Me-10), 1.17–1.28 (1H, m, H-3), 1.34–1.45 (1H, m, H-2), 1.70–1.82 (1H, m, H-2), 1.82–1.96 (2H, m, H-5, H-3), 2.04 (1H, t, *J* = 3.7 Hz, H-4), 2.42 (1H, dt, *J* = 16.8 Hz, *J* = 3.6 Hz, H-5), 7.16–7.23 (1H, m, H-15), 7.36 (2H, t, *J* = 8.1 Hz, H-14,16), 7.64 (2H, d, *J* = 7.5 Hz, H-13,17), 8.32 (1H, s, NH 19), 9.23 (1H, s, NH 18). ^13^C-NMR (CDCl_3_): δ_c_ 10.96 (Me-10), 18.43 and 19.33 (Me-8 and Me-9), 27.00 (C-3), 32.35 (C-2), 33.79 (C-5), 43.80 (C-4), 48.08 (C-7), 52.75 (C-1), 123.97 (C-13, C-17), 125.68 (C-15), 128.52 (C-14, C-16), 137.88 (C-12), 165.84 (C-6), 175.69 (C-11). HRMS (EI): *m*/*z* calcd for C_17_H_23_N_3_S: 301.1607; found 301.1605. [α]^D^_25_ *+*56.56 (c = 1.22 in CHCl_3_). IR (KBr, cm^−1^): ν 3650–3150 (NH), 2962 (C-H), 1540 (C=N), 1184 (C=S). M.p. 181.5–182.5 °C.

(*E*)-N-allyl-2-((1*S*,4*S*)-1,7,7-trimethylbicyclo[2.2.1]heptan-2-ylidene)hydrazinecarbothioamide (**2′b**), white crystalline substance, yield 55% (0.44 g). ^1^H-NMR (CDCl_3_): δ_H_ 0.70 (3H, s, Me-9), 0.90 (3H, s, Me-8), 0.95 (3H, s, Me-10), 1.15–1.21 (1H, m, H-3), 1.30–1.37 (1H, m, H-2), 1.68–1.75 (1H, m, H-2), 1.80–1.88 (2H, m, H-5, H-3), 1.99 (1H, t, *J* = 3.7 Hz, H-4), 2.34 (1H, dt, *J* = 16.8 Hz, *J* = 3.7 Hz, H-5), 4.26–4.36 (2H, m, H-12), 5.88–5.96 (1H, m, H-13), 5.14–5.25 (2H, m, H-14), 7.48 (1H, s, NH 16), 8.16 (1H, s, NH 15). ^13^C-NMR (CDCl_3_): δ_c_ 10.97 (Me-10), 18.47 and 19.34 (Me-8 and Me-9), 27.06 (C-3), 32.42 (C-2), 33.65 (C-5), 43.85 (C-4), 46.61 (C-12), 48.01 (C-7), 52.64 (C-1), 116.53 (C-14), 133.56 (C-13), 165.38 (C-6), 177.65 (C-11). HRMS (EI): *m*/*z* calcd for C_14_H_23_N_3_S: 265.1607; found 265.1608. [α]^D^_25_ *+*50.94 (c = 1.06 in CHCl_3_). IR (KBr, cm^−1^): ν 3600–3100 (NH), 2954 (C-H), 1537 (C=N), 1494 (CH_3_), 1213 (C=S). M.p. 112.6–113.7 °C.

(*E*)-N-ethyl-2-((1*S*,4*S*)-1,7,7-trimethylbicyclo[2.2.1]heptan-2-ylidene)hydrazinecarbothioamide (**2′c**), white crystalline substance, yield 70% (0.53 g). ^1^H-NMR (CDCl_3_): δ_H_ 0.69 (3H, s, Me-9), 0.89 (3H, s, Me-8), 0.95 (3H, s, Me-10), 1.13–1.20 (1H, m, H-3), 1.24 (3H, t, *J* = 7.1 Hz, H-13), 1.29–1.36 (1H, m, H-2), 1.67–1.74 (1H, m, H-2), 1.78–1.88 (2H, m, H-5, H-3), 1.98 (1H, t, *J* = 3.7 Hz, H-4), 2.33 (1H, dt, *J* = 16.8 Hz, *J* = 3.7 Hz, H-5), 3.62–3.74 (2H, m, H-12), 7.36 (1H, s, NH 15), 8.08 (1H, s, NH 14). ^13^C-NMR (CDCl_3_): δ_c_ 10.97 (Me-10), 14.39 (C-13), 18.43 and 19.30 (Me-8 and Me-9), 27.01 (C-3), 32.36 (C-2), 33.58 (C-5), 39.08 (C-12), 43.75 (C-4), 47.97 (C-7), 52.54 (C-1), 165.04 (C-6), 177.09 (C-11). HRMS (EI): *m*/*z* calcd for C_13_H_23_N_3_S: 253.1607; found 253.1610. [α]^D^_25_ *+*45.45 (c = 1.21 in CHCl_3_). IR (KBr, cm^−1^): ν 3600–3100 (NH), 2960 (C-H), 1533 (C=N), 1494 (CH_3_), 1222 (C=S). M.p. 146.9–147.7 °C.

(E)-N-methyl-2-((1*S*,4*S*)-1,7,7-trimethylbicyclo[2.2.1]heptan-2-ylidene)hydrazinecarbothioamide (**2′d**), white crystalline substance, yield 65% (0.47 g). ^1^H-NMR (CDCl_3_): δ_H_ 0.69 (3H, s, Me-9), 0.89 (3H, s, Me-8), 0.95 (3H, s, Me-10), 1.14–1.20 (1H, m, H-3), 1.28–1.35 (1H, m, H-2), 1.67–1.74 (1H, m, H-2), 1.79–1.88 (2H, m, H-5, H-3), 1.99 (1H, t, *J* = 3.7 Hz, H-4), 2.33 (1H, dt, *J* = 16.8 Hz, *J* = 3.7 Hz, H-5), 3.18 (3H, d, *J* = 5 Hz, H-12), 7.42 (1H, s, NH 14), 8.14 (1H, s, NH 13). ^13^C-NMR (CDCl_3_): δ_c_ 10.96 (Me-10), 18.39 and 19.25 (Me-8 and Me-9), 26.98 (C-3), 30.88 (C-12), 32.32 (C-2), 33.53 (C-5), 43.72 (C-4), 47.92 (C-7), 52.51 (C-1), 165.14 (C-6), 178.23 (C-11). HRMS (EI): *m*/*z* calcd for C_12_H_21_N_3_S: 239.1451; found 239.1446. [α]^D^_25_ *+*58.89 (c = 0.9 in CHCl_3_). IR (KBr, cm^−1^): ν 3600–3100 (NH), 2964 (C-H), 1540 (C=N), 1498 (CH_3_), 1241 (C=S). M.p. 133.3–136.2 °C.

#### 3.1.3. General Procedure for Camphor 2-Imino-4-Thiazolidinones Synthesis **3a–d**, **3′a–d**

(+) or (−)-Camphor thiosemicarbazones (1 mmol) were dissolved in CHCl_3_ (25 mL), and Et_3_N (2 mmol, 0.28 mL) and ethyl bromoacetate (1.1 mmol, 0.12 mL) were added. The reaction mixture was left under stirring overnight at room temperature. Then, it was washed with water (3 × 30 mL), the organic layer was dried over Na_2_SO_4_, and the solvent was distilled off. Compounds 3′a and 3′d were additionally purified by column chromatography, and the eluent was a solution of methanol in chloroform with a concentration gradient from 2% to 50%.

((*Z*)-3-phenyl-2-((E)-((1*R*,4*R*)-1,7,7-trimethylbicyclo[2.2.1]heptan-2-ylidene)hydrazono)thiazolidin-4-one (**3a**), yellow-white crystalline substance, yield 60% (0.2 g). ^1^H-NMR (CDCl_3_): δ_H_ 0.73 (3H, s, Me-9), 0.89 (3H, s, Me-8), 1.03 (3H, s, Me-10), 1.10–1.20 (1H, m, H-3), 1.37–1.45 (1H, m, H-2), 1.65–1.91 (4H, m, H-2,3,4,5), 2.33 (1H, dt, *J* = 16.8 Hz, *J* = 3.7 Hz, H-5), 3.86 (2H, s, H-13), 7.27–7.49 (5H, m, H-15, H-16, H-17, H-18, H-19). ^13^C-NMR (CDCl_3_): δ_c_ 10.96 (Me-10), 18.62 and 19.50 (Me-8 and Me-9), 27.00 (C-3), 32.30 (C-2), 32.50 (C-5), 35.84 (C-13), 43.63 (C-4), 47.90 (C-7), 52.78 (C-1), 127.47 (C-15,19), 128.47 (C-17), 128.91 (C-16,18), 134.57 (C-14), 158.71 (C-11), 171.59 (C-12), 181.10 (C-6). HRMS (EI): *m*/*z* calcd for C_19_H_23_ON_3_S: 341.1556; found 341.1559. [α]^D^_25_ *−*24.00 (c = 0.5 in CHCl_3_). IR (KBr, cm^−1^): ν 1733 (C=O), 1648 (C=N), 1589 (C=N). M.p. 152.6–155.5 °C.

(*Z*)-3-allyl-2-((*E*)-((1*R*,4*R*)-1,7,7-trimethylbicyclo[2.2.1]heptan-2-ylidene)hydrazono)thiazolidin-4-one (**3b**), yellow-white amorphous substance, yield 50% (0.15 g). ^1^H-NMR (CDCl_3_): δ_H_ 0.77 (3H, s, Me-9), 0.92 (3H, s, Me-8), 1.03 (3H, s, Me-10), 1.16–1.27 (1H, m, H-3), 1.37–1.48 (1H, m, H-2), 1.66–1.92 (3H, m, H-2, 3, 4), 2.04 (1H, d, *J* = 17.7 Hz, H-5), 2.52 (1H, dt, *J* = 16.8 Hz, *J* = 3.7 Hz, H-5), 3.70 (2H, s, H-13), 4.34 (2H, d, *J* = 6.4 Hz, H-14), 5.15–5.31 (2H, m, H-16), 5.77–5.92 (1H, m, H-15). ^13^C-NMR (CDCl_3_): δ_c_ 10.67 (Me-10), 18.34 and 19.21 (Me-8 and Me-9), 26.83 (C-3), 31.91 (C-2), 32.23 (C-5), 35.57 (C-13), 43.16 (C-4), 44.88 (C-14), 47.60 (C-7), 52.42 (C-1), 118.26 (C-16), 130.13 (C-15), 157.63 (C-11), 171.21 (C-12), 180.51 (C-6). HRMS (EI): *m*/*z* calcd for C_16_H_23_ON_3_S: 305.1556; found 305.1561. [α]^D^_25_ *−*46.02 (c = 1.13 in CHCl_3_). IR (KBr, cm^−1^): ν 1722 (C=O), 1660 (C=N), 1598 (C=N).

(*Z*)-3-ethyl-2-((*E*)-((1*R*,4*R*)-1,7,7-trimethylbicyclo[2.2.1]heptan-2-ylidene)hydrazono)thiazolidin-4-one (**3c**), yellow-white crystalline substance, yield 50% (0.13 g). ^1^H-NMR (CDCl_3_): δ_H_ 0.77 (3H, s, Me-9), 0.92 (3H, s, Me-8), 1.03 (3H, s, Me-10), 1.17–1.27 (4H, m, Me-15, H-3), 1.38–1.49 (1H, m, H-2), 1.66–1.92 (3H, m, H-2, 3, 4), 2.06 (1H, d, *J* = 18.3 Hz, H-5), 2.54 (1H, dt, *J* = 16.8 Hz, *J* = 3.7 Hz, H-5), 3.67 (2H, s, H-13), 3.80 (2H, q, *J* = 7.2 Hz, H-14). ^13^C-NMR (CDCl_3_): δ_c_ 10.97 (Me-10), 12.17 (C-15), 18.64 and 19.51 (Me-8 and Me-9), 27.14 (C-3), 32.30 (C-2), 32.56 (C-5), 35.82 (C-13), 38.23 (C-14), 43.71 (C-4), 47.88 (C-7), 52.72 (C-1), 158.29 (C-11), 171.69 (C-12), 180.54 (C-6). HRMS (EI): *m*/*z* calcd for C_15_H_23_ON_3_S: 293.1556; found 293.1558. [α]^D^_25_ *−*55.79 (c = 0.95 in CHCl_3_). IR (KBr, cm^−1^): ν 1718 (C=O), 1660 (C=N), 1591 (C=N). M.p. 78.4–80.2 °C.

(*Z*)-3-methyl-2-((*E*)-((1*R*,4*R*)-1,7,7-trimethylbicyclo[2.2.1]heptan-2-ylidene)hydrazono)thiazolidin-4-one (**3d**), white crystalline substance, yield 60% (0.17 g). ^1^H-NMR (CDCl_3_): δ_H_ 0.76 (3H, s, Me-9), 0.91 (3H, s, Me-8), 1.02 (3H, s, Me-10), 1.18–1.25 (1H, m, H-3), 1.38–1.45 (1H, m, H-2), 1.68–1.75 (1H, m, H-2), 1.78–1.86 (1H, m, H-3), 1.89 (1H, t, *J* = 3.7 Hz, H-4), 2.07 (1H, d, H-5), 2.54 (1H, dt, *J* = 16.8 Hz, *J* = 3.7 Hz, H-5), 3.21 (3H, s, Me-14), 3.68 (2H, s, H-13). ^13^C-NMR (CDCl_3_): δ_c_ 10.98 (Me-10), 18.63 and 19.48 (Me-8 and Me-9), 27.12 (C-3), 29.43 (Me-14), 32.19 (C-2), 32.54 (C-5), 35.81 (C-13), 43.68 (C-4), 47.84 (C-7), 52.75 (C-1), 159.47 (C-11), 172.03 (C-12), 180.76 (C-6). HRMS (EI): *m*/*z* calcd for C_14_H_21_ON_3_S: 279.1400; found 279.1403. [α]^D^_25_ *−*45.88 (c = 0.85 in CHCl_3_). IR (KBr, cm^−1^): ν 1729 (C=O), 1662 (C=N), 1598 (C=N). M.p. 115.5–117.9 °C.

(*Z*)-3-phenyl-2-((*E*)-((1*S*,4*S*)-1,7,7-trimethylbicyclo[2.2.1]heptan-2-ylidene)hydrazono)thiazolidin-4-one (**3′a**), yellow-white crystalline substance, yield 45% (0.15 g). ^1^H-NMR (CDCl_3_): δ_H_ 0.73 (3H, s, Me-9), 0.89 (3H, s, Me-8), 1.03 (3H, s, Me-10), 1.10–1.20 (1H, m, H-3), 1.34–1.45 (1H, m, H-2), 1.64–1.91 (4H, m, H-2, 3, 4, 5), 2.33 (1H, dt, *J* = 16.8 Hz, *J* = 3.7 Hz, H-5), 3.86 (2H, s, H-13), 7.28–7.49 (5H, m, H-15, H-16, H-17, H-18, H-19). ^13^C-NMR (CDCl_3_): δ_c_ 10.96 (Me-10), 18.62 and 19.50 (Me-8 and Me-9), 27.00 (C-3), 32.30 (C-2), 32.49 (C-5), 35.84 (C-13), 43.62 (C-4), 47.90 (C-7), 52.78 (C-1), 127.46 (C-15,19), 128.47 (C-17), 128.91 (C-16,18), 134.57 (C-14), 158.71 (C-11), 171.59 (C-12), 181.11 (C-6). HRMS (EI): *m*/*z* calcd for C_19_H_23_ON_3_S: 341.1556; found 341.1550. [α]^D^_25_ *+*24.47 (c = 0.94 in CHCl_3_).

(*Z*)-3-allyl-2-((*E*)-((1*S*,4*S*)-1,7,7-trimethylbicyclo[2.2.1]heptan-2-ylidene)hydrazono)thiazolidin-4-one (**3′b**), yellow-white amorphous substance, yield 60% (0.18 g). ^1^H-NMR (CDCl_3_): δ_H_ 0.77 (3H, s, Me-9), 0.92 (3H, s, Me-8), 1.03 (3H, s, Me-10), 1.16–1.27 (1H, m, H-3), 1.37–1.49 (1H, m, H-2), 1.66–1.92 (3H, m, H-2, 3, 4), 2.04 (1H, d, *J* = 18.3 Hz, H-5), 2.52 (1H, dt, *J* = 16.8 Hz, *J* = 3.7 Hz, H-5), 3.70 (2H, s, H-13), 4.34 (2H, d, *J* = 6 Hz, H-14), 5.14–5.31 (2H, m, H-16), 5.77–5.93 (1H, m, H-15). ^13^C-NMR (CDCl_3_): δ_c_ 10.98 (Me-10), 18.64 and 19.51 (Me-8 and Me-9), 27.12 (C-3), 32.22 (C-2), 32.52 (C-5), 35.87 (C-13), 43.67 (C-4), 45.18 (C-14), 47.91 (C-7), 52.71 (C-1), 118.57 (C-16), 130.41 (C-15), 157.94 (C-11), 171.52 (C-12), 180.84 (C-6). HRMS (EI): *m*/*z* calcd for C_16_H_23_ON_3_S: 305.1556; found 305.1558. [α]^D^_25_ *+* 48.44 (c = 1.28 in CHCl_3_). IR (KBr, cm^−1^): ν 1722 (C=O), 1660 (C=N), 1598 (C=N).

(*Z*)-3-ethyl-2-((*E*)-((1*S*,4*S*)-1,7,7-trimethylbicyclo[2.2.1]heptan-2-ylidene)hydrazono)thiazolidin-4-one (**3′c**), yellow-white crystalline substance, yield 70% (0.21 g). ^1^H-NMR (CDCl_3_): δ_H_ 0.78 (3H, s, Me-9), 0.92 (3H, s, Me-8), 1.03 (3H, s, Me-10), 1.17–1.27 (4H, m, Me-15, H-3), 1.38–1.49 (1H, m, H-2), 1.67–1.92 (3H, m, H-2, 3, 4), 2.06 (1H, d, *J* = 17.8 Hz, H-5), 2.54 (1H, dt, *J* = 16.8 Hz, *J* = 3.7 Hz, H-5), 3.67 (2H, s, H-13), 3.80 (2H, q, *J* = 3.9 Hz, H-14). ^13^C-NMR (CDCl_3_): δ_c_ 10.99 (Me-10), 12.18 (C-15), 18.64 and 19.51 (Me-8 and Me-9), 27.13 (C-3), 32.31 (C-2), 32.55 (C-5), 35.82 (C-13), 38.23 (C-14), 43.69 (C-4), 47.88 (C-7), 52.72 (C-1), 158.29 (C-11), 171.71 (C-12), 180.60 (C-6). HRMS (EI): *m*/*z* calcd for C_15_H_23_ON_3_S: 293.1556; found 293.1551. [α]^D^_25_ *+*74.15 (c = 1.47 in CHCl_3_). IR (KBr, cm^−1^): ν 1718 (C=O), 1660 (C=N), 1591 (C=N). M.p. 79.1 °C.

(*Z*)-3-methyl-2-((*E*)-((1*S*,4*S*)-1,7,7-trimethylbicyclo[2.2.1]heptan-2-ylidene)hydrazono)thiazolidin-4-one (**3′d**), yellow-white crystalline substance, yield 60% (0.17 g). ^1^H-NMR (CDCl_3_): δ_H_ 0.77 (3H, s, Me-9), 0.92 (3H, s, Me-8), 1.03 (3H, s, Me-10), 1.17–1.28 (1H, m, H-3), 1.37–1.48 (1H, m, H-2), 1.67–1.93 (3H, m, H-2, 3, 4), 2.08 (1H, d, *J* = 18.1 Hz, H-5), 2.54 (1H, dt, *J* = 16.8 Hz, *J* = 3.7 Hz, H-5), 3.21 (3H, s, Me-14), 3.69 (2H, s, H-13). ^13^C-NMR (CDCl_3_): δ_c_ 10.99 (Me-10), 18.64 and 19.49 (Me-8 and Me-9), 27.13 (C-3), 29.46 (Me-14), 32.20 (C-2), 32.55 (C-5), 35.82 (C-13), 43.70 (C-4), 47.87 (C-7), 52.76 (C-1), 159.48 (C-11), 172.04 (C-12), 180.77 (C-6). HRMS (EI): *m*/*z* calcd for C_14_H_21_ON_3_S: 279.1400; found 279.1404. [α]^D^_25_ *+* 67.24 (c = 1.74 in CHCl_3_). IR (KBr, cm^−1^): ν 1727 (C=O), 1662 (C=N), 1598 (C=N). M.p. 108.7–116.5 °C.

#### 3.1.4. General Procedure for Camphor 5,5-Dimethyl-2-Imino-4-Thiazolidinones Synthesis **4a–d**

(+) or (−)-Camphor thiosemicarbazones (1 mmol) were dissolved in DMF (15 mL), and DIPEA (2 mmol, 0.35 mL) and ethyl α-bromoisobutyrate (10 mmol, 1.46 mL) were added. Refluxed for 4 h, then cooled to room temperature. Next, 50 mL of water was added to the reaction mixture and extracted with CHCl_3_ (3 × 30 mL). The organic extract was dried over Na_2_SO_4_, then the solvent was evaporated. The product was purified by column chromatography, and the eluent was a solution of ethyl acetate in hexane with a concentration gradient from 2% to 50%.

(*Z*)-5,5-dimethyl-3-phenyl-2-((*E*)-((1*R*,4*R*)-1,7,7-trimethylbicyclo[2.2.1]heptan-2-ylidene)hydrazono)thiazolidin-4-one (**4a**), yellow-white crystalline substance, yield 50% (0.18 g). ^1^H-NMR (CDCl_3_): δ_H_ 0.73 (3H, s, Me-9), 0.89 (3H, s, Me-8), 1.03 (3H, s, Me-10), 1.10–1.20 (1H, m, H-3), 1.36–1.44 (1H, m, H-2), 1.62–1.82 (9H, m, H-3,4,2, Me-14, Me-15), 1.88 (1H, d, *J* = 18.1 Hz, H-5), 2.33 (1H, dt, *J* = 18.3 Hz, *J* = 3.5 Hz, H-5), 7.30–7.47 (5H, m, H-20, H-21, H-17, H-18, H-19). ^13^C-NMR (CDCl_3_): δ_c_ 10.96 (Me-10), 18.64 and 19.56 (Me-8 and Me-9), 27.03 (C-3), 28.68 and 28.49 (C-14 and C-15), 32.56 (C-2), 35.87 (C-5), 43.70 (C-4), 47.90 (C-7), 50.52 (C-13), 52.74 (C-1), 127.54 (C-17,21), 128.25 (C-19), 128.74 (C-20,18), 134.91 (C-16), 156.64 (C-11), 177.66 (C-6), 180.67 (C-12). HRMS (EI): *m*/*z* calcd for C_21_H_27_ON_3_S: 369.1869; found 369.1865. IR (KBr, cm^−1^): ν 1730 (C=N), 1666 (C=O), 1588 (C=N). M.p. 164.2 °C.

(*Z*)-3-allyl-5,5-dimethyl-2-((*E*)-((1*R*,4*R*)-1,7,7-trimethylbicyclo[2.2.1]heptan-2-ylidene)hydrazono)thiazolidin-4-one (**4b**), yellow-white crystalline substance, yield 50% (0.17 g). ^1^H-NMR (CDCl_3_): δ_H_ 0.77 (3H, s, Me-9), 0.91 (3H, s, Me-8), 1.02 (3H, s, Me-10), 1.18–1.24 (1H, m, H-3), 1.40–1.48 (1H, m, H-2), 1,60 (6H, s, Me-14 and Me-15), 1.68–1.75 (1H, m, H-2), 1.78–1.86 (1H, m, H-3), 1.88 (1H, t, *J* = 4.3 Hz, H-4), 2.04 (1H, d, *J* = 18.4 Hz, H-5), 2.51 (1H, dt, *J* = 18.2 Hz, *J* = 3.7 Hz, H-5), 4.34 (2H, d, *J* = 5.8 Hz, H-16), 5.15–5.27 (2H, m, H-18), 5.81–5.90 (1H, m, H-17). ^13^C-NMR (CDCl_3_): δ_c_ 10.94 (Me-10), 18.61 and 19.52 (Me-8 and Me-9), 27.08 (C-3), 28.21 and 28.38 (Me-14 and Me-15), 32.50 (C-2), 35.83 (C-5), 43.63 (C-4), 44.98 (C-16), 47.86 (C-7), 50.78 (C-13), 52.62 (C-1), 118.08 (C-18), 130.59 (C-17), 155.84 (C-11), 177.54 (C-6), 180.48 (C-12). HRMS (EI): *m*/*z* calcd for C_18_H_27_ON_3_S: 333.1869; found 333.1867. IR (KBr, cm^−1^): ν 3085 (HRC=CH_2_), 1707 (C=N), 1666 (C=O), 1596 (C=N). M.p. 107.0–109.0 °C.

((*Z*)-3-ethyl-5,5-dimethyl-2-((*E*)-((1*R*,4*R*)-1,7,7-trimethylbicyclo[2.2.1]heptan-2-ylidene)hydrazono)thiazolidin-4-one (**4c**), yellow-white crystalline substance, yield 60% (0.19 g). ^1^H-NMR (CDCl_3_): δ_H_ 0.78 (3H, s, Me-9), 0.92 (3H, s, Me-8), 1.03 (3H, s, Me-10), 1.18–1.26 (4H, m, Me-17, H-3), 1.40–1.50 (1H, m, H-2), 1,59 (6H, s, Me-14 and Me-15), 1.68–1.76 (1H, m, H-2), 1.78–1.85 (1H, m, H-3), 1.89 (1H, t, *J* = 4.3 Hz, H-4), 2.07 (1H, d, *J* = 18.3 Hz, H-5), 2.54 (1H, dt, *J* = 18.3 Hz, *J* = 3.9 Hz, H-5), 3.80 (2H, q, *J* = 7.1 Hz, H-16). ^13^C-NMR (CDCl_3_): δ_c_ 11.00 (Me-10), 12.30 (C-17), 18.67 and 19.59 (Me-8 and Me-9), 27.17 (C-3), 28.20 and 28.35 (Me-14 and Me-15), 32.60 (C-2), 35.85 (C-5), 38.15 (C-16), 43.74 (C-4), 47.90 (C-7), 50.52 (C-13), 52.69 (C-1), 156.25 (C-11), 177.72 (C-6), 180.22 (C-12). HRMS (EI): *m*/*z* calcd for C_17_H_27_ON_3_S: 321.1869; found 321.1861. IR (KBr, cm^−1^): ν 1704 (C=N), 1666 (C=O), 1596 (C=N). M.p. 116.9–123.0 °C.

(*Z*)-3,5,5-trimethyl-2-((*E*)-((1*R*,4*R*)-1,7,7-trimethylbicyclo[2.2.1]heptan-2-ylidene)hydrazono)thiazolidin-4-one (**4d**), orange crystalline substance, yield 70% (0.22 g). ^1^H-NMR (CDCl_3_): δ_H_ 0.77 (3H, s, Me-9), 0.91 (3H, s, Me-8), 1.02 (3H, s, Me-10), 1.18–1.25 (1H, m, H-3), 1.39–1.47 (1H, m, H-2), 1,60 (6H, s, Me-14 and Me-15), 1.68–1.75 (1H, m, H-2), 1.79–1.86 (1H, m, H-3), 1.87–1.91 (1H, m, H-4), 2.08 (1H, d, *J* = 18.0 Hz, H-5), 2.55 (1H, dt, *J* = 18.3 Hz, *J* = 3.6 Hz, H-5), 3.22 (3H, s, Me-16). ^13^C-NMR (CDCl_3_): δ_c_ 11.00 (Me-10), 18.67 and 19.56 (Me-8 and Me-9), 27.17 (C-3), 28.24 and 28.38 (Me-14 and Me-15), 29.56 (Me-16), 32.62 (C-2), 35.86 (C-5), 43.76 (C-4), 47.88 (C-7), 50.85 (C-13), 52.76 (C-1), 157.56 (C-11), 178.15 (C-6), 180.46 (C-12). HRMS (EI): *m/z* calcd for C_16_H_25_ON_3_S: 307.1713; found 307.1709. IR (KBr, cm^−1^): ν 1720 (C=N), 1656 (C=O), 1602 (C=N). M.p. 115.3 °C.

#### 3.1.5. General Procedure for Camphor 2,3-Dihydrothiazoles Synthesis **5-8 a–d**

(+)-Camphor thiosemicarbazones (1 mmol) were dissolved in EtOH (25 mL), and NaOAc (2 mmol, 0.16 g) and the corresponding 2-bromoacetophenone (1 mmol) were added. The mixture was stirred for 12 h. The EtOH was then evaporated in vacuo, and the dry mixture was dissolved in 30 mL CHCl_3_ and washed with water (3 × 30 mL). The organic layer was dried over Na_2_SO_4_, and the solvent was evaporated. Some products (**5a**, **7a**, **8d**) were purified by column chromatography, using ethyl acetate in hexane with a concentration gradient from 2% to 50% as the eluent.

(*E*)-4-(4-chlorophenyl)-3-phenyl-2-((*E*)-((1*R*,4*R*)-1,7,7-trimethylbicyclo[2.2.1]heptan-2-ylidene)hydrazono)-2,3-dihydrothiazole (**5a**), white crystalline substance, yield 55% (0.24 g). ^1^H-NMR (CDCl_3_): δ_H_ 0.75 (3H, s, Me-9), 0.89 (3H, s, Me-8), 1.05 (3H, s, Me-10), 1.13–1.20 (1H, m, H-3), 1.39–1.47 (1H, m, H-2), 1.64–1.72 (1H, m, H-2), 1.73–1.82 (2H, m, H-3, H-4), 1.96 (1H, d, *J* = 17.9 Hz, H-5), 2.42 (1H, d, *J* = 18.6 Hz, H-5), 6.05 (1H, s, H-13), 6.95–7.30 (9H, m, H-15, 16, 18, 19, 21, 22, 23, 24, 25). ^13^C-NMR (CDCl_3_): δ_c_ 11.18 (Me-10), 18.73 and 19.58 (Me-8 and Me-9), 27.17 (C-3), 43.88 (C-4), 32.78 (C-2), 35.84 (C-5), 47.81 (C-7), 52.27 (C-1), 101.33 (C-13), 126.90 (C-23), 128.08, 128.32, 128,41, 129.00 (C-15,16,18,19,25,21,24,22), 130.05 (C-14), 133.79 (C-17), 137.86 (C-20), 138.52 (C-12), 165.56 (C-11), 175.62 (C-6). HRMS (EI): *m*/*z* calcd for C_25_H_26_N_3_ClS: 435.1531; found 435.1533. [α]^D^_25_ 0 (c = 0.99 in CHCl_3_). IR (KBr, cm^−1^): ν 1656 (C=N), 1550 (C=N), 1089 (C-Cl). M.p. 195.5–200.3 °C.

(*E*)-3-allyl-4-(4-chlorophenyl)-2-((*E*)-((1*R*,4*R*)-1,7,7-trimethylbicyclo[2.2.1]heptan-2-ylidene)hydrazono)-2,3-dihydrothiazole (**5b**), orange crystalline substance, yield 90% (0.36 g). ^1^H-NMR (CDCl_3_): δ_H_ 0.77 (3H, s, Me-9), 0.91 (3H, s, Me-8), 1.06 (3H, s, Me-10), 1.17–1.27 (1H, m, H-3), 1.43–1.51 (1H, m, H-2), 1.66–1.75 (1H, m, H-2), 1.76–1.89 (2H, m, H-4, H-3), 2.08 (1H, d, *J* = 18.3 Hz, H-5), 2.54 (1H, d, *J* = 17.7 Hz, H-5), 4.27 (2H, s, H-20), 4.94–5.13 (2H, m, H-22), 5.79–5.90 (2H, m, H-21, 13), 7.21–7.38 (4H, m, H-15, H-16, H-18, H-19). ^13^C-NMR (CDCl_3_): δ_c_ 11.20 (Me-10), 18.74 and 19.58 (Me-8 and Me-9), 27.25 (C-3), 32.78 (C-2), 35.84 (C-5), 43.87 (C-4), 47.82 (C-20), 48.09 (C-7), 52.26 (C-1), 99.44 (C-13), 117.05 (C-22), 128.66 (C-19,15), 129.99 (C-14,18,16), 132.20 (C-21), 134.90 (C-17), 139.26 (C-12), 165.55 (C-11), 174.76 (C-6). HRMS (EI): *m*/*z* calcd for C_22_H_26_N_3_ClS: 399.1531; found 399.1529. [α]^D^_25_ *−*13.08 (c = 1.3 in CHCl_3_). IR (KBr, cm^−1^): ν 1654 (C=N), 1565 (C=N), 1091 (C-Cl). M.p. 73.2 °C.

(*E*)-4-(4-chlorophenyl)-3-ethyl-2-((*E*)-((1*R*,4*R*)-1,7,7-trimethylbicyclo[2.2.1]heptan-2-ylidene)hydrazono)-2,3-dihydrothiazole (**5c**), orange crystalline substance, yield 70% (0.27 g). ^1^H-NMR (CDCl_3_): δ_H_ 0.79 (3H, s, Me-9), 0.92 (3H, s, Me-8), 1.06 (3H, s, Me-10), 1.13 (3H, t, *J* = 7.0 Hz, Me-21), 1.20–1.28 (1H, m, H-3), 1.44–1.51 (1H, m, H-2), 1.67–1.76 (1H, m, H-2), 1.78–1.90 (2H, m, H-4, H-3), 2.12 (1H, d, *J* = 17.6 Hz, H-5), 2.57 (1H, d, *J* = 17.8 Hz, H-5), 3.73 (2H, m, H-20), 5.78 (1H, s, H-13), 7.23–7.40 (4H, m, H-15, H-16, H-18, H-19). ^13^C-NMR (CDCl_3_): δ_c_ 11.22 (Me-10), 13.07 (C-21), 18.75 and 19.60 (Me-8 and Me-9), 27.27 (C-3), 32.83 (C-2), 35.94 (C-5), 40.98 (C-20), 43.95 (C-4), 47.82 (C-7), 52.36 (C-1), 99.71 (C-13), 128.85 (C-15,19), 129.95 (C-14,18,16), 134.95 (C-17), 139.19 (C-12), 165.95 (C-11), 174.50 (C-6). HRMS (EI): *m*/*z* calcd for C_21_H_26_N_3_ClS: 387.1531; found 387.1532. [α]^D^_25_ *−*17.78 (c = 0.9 in CHCl_3_). IR (KBr, cm^−1^): ν 1650 (C=N), 1560 (C=N), 1087 (C-Cl). M.p. 65.3 °C.

(*E*)-4-(4-chlorophenyl)-3-methyl-2-((*E*)-((1*R*,4*R*)-1,7,7-trimethylbicyclo[2.2.1]heptan-2-ylidene)hydrazono)-2,3-dihydrothiazole (**5d**), orange crystalline substance, yield 90% (0.34 g). ^1^H-NMR (CDCl_3_): δ_H_ 0.78 (3H, s, Me-9), 0.92 (3H, s, Me-8), 1.05 (3H, s, Me-10), 1.20–1.28 (1H, m, H-3), 1.43–1.50 (1H, m, H-2), 1.68–1.75 (1H, m, H-2), 1.78–1.86 (1H, m, H-3), 1.89 (1H, t, *J* = 4.4 Hz, H-4), 2.15 (1H, d, *J* = 17.7 Hz, H-5), 2.60 (1H, d, *J* = 18.2 Hz, H-5), 3.28 (3H, s, H-20), 5.88 (1H, s, H-13), 7.23–7.40 (4H, m, H-15, H-16, H-18, H-19). ^13^C-NMR (CDCl_3_): δ_c_ 11.20 (Me-10), 18.73 and 19.56 (Me-8 and Me-9), 27.26 (C-3), 32.84 (C-2), 33.80 (C-20), 35.92 (C-5), 43.99 (C-4), 47.78 (C-7), 52.43 (C-1), 99.89 (C-13), 128.90 (C-15,19), 129.76 (C-14,16,18), 135.04 (C-17), 139.57 (C-12), 167.48 (C-11), 174.50 (C-6). HRMS (EI): *m*/*z* calcd for C_20_H_24_N_3_ClS: 373.1374; found 373.1376. [α]^D^_25_ *−*16.81 (c = 1.19 in CHCl_3_). IR (KBr, cm^−1^): ν 1646 (C=N), 1565 (C=N), 1089 (C-Cl). M.p. 126.8 °C.

(*E*)-3-phenyl-4-p-tolyl-2-((*E*)-((1*R*,4*R*)-1,7,7-trimethylbicyclo[2.2.1]heptan-2-ylidene)hydrazono)-2,3-dihydrothiazole (**6a**), orange crystalline substance, yield 95% (0.39 g). ^1^H-NMR (CDCl_3_): δ_H_ 0.75 (3H, s, Me-9), 0.89 (3H, s, Me-8), 1.05 (3H, s, Me-10), 1.12–1.21 (1H, m, H-3), 1.38–1.48 (1H, m, H-2), 1.64–1.82 (3H, m, H-2,3,4), 1.96 (1H, d, *J* = 18.1 Hz, H-5), 2.24 (3H, s, H-26), 2.41 (1H, dt, *J* = 18 Hz, *J* = 3.4 Hz, H-5), 5.99 (1H, s, H-13), 6.95 (4H, m, H-15, H-16, H-18, H-19), 7.14–7.27 (5H, H-21,22,23,24,25). ^13^C-NMR (CDCl_3_): δ_c_ 10.88 (Me-10), 18.43 and 19.29 (Me-8 and Me-9), 20.76 (C-26), 26.87 (C-3), 32.49 (C-2), 35.50 (C-5), 43.60 (C-4), 47.48 (C-7), 51.92 (C-1), 99.70 (C-13), 126.33 (C-23), 127.43 (C-25,21), 127.87 (C-18,16), 127.90 (C-19,15), 128.40 (C-24,22), 128.47 (C-14), 137.86 (C-20), 137.47 (C-17), 139.45 (C-12), 165.57 (C-11), 174.86 (C-6). HRMS (EI): *m*/*z* calcd for C_26_H_29_N_3_S: 415.2070; found 415.2070. [α]^D^_25_ 0 (c = 1.32 in CHCl_3_). IR (KBr, cm^−1^): ν 1654 (C=N) 1552 (C=N). M.p. 151.5–156.3 °C.

(*E*)-3-allyl-4-p-tolyl-2-((*E*)-((1*R*,4*R*)-1,7,7-trimethylbicyclo[2.2.1]heptan-2-ylidene)hydrazono)-2,3-dihydrothiazole (**6b**), orange amorphous substance, yield 97% (0.37 g). ^1^H-NMR (CDCl_3_): δ_H_ 0.78 (3H, s, Me-9), 0.91 (3H, s, Me-8), 1.06 (3H, s, Me-10), 1.18–1.27 (1H, m, H-3), 1.44–1.52 (1H, m, H-2), 1.66–1.75 (1H, m, H-2), 1.76–1.88 (2H, m, H-4, H-3), 2.08 (1H, d, *J* = 18 Hz, H-5), 2.36 (3H, s, H-23), 2.54 (1H, dt, *J* = 18.3 Hz, *J* = 3.4 Hz, H-5), 4.28 (2H, d, *J* = 5 Hz, H-20), 4.95–5.12 (2H, m, H-22), 5.76 (1H, s, H-13), 5.79–5.92 (1H, m, H-21), 7.15–7.25 (4H, m, H-15, H-16, H-18, H-19). ^13^C-NMR (CDCl_3_): δ_c_ 11.25 (Me-10), 18.79 and 19.63 (Me-8 and Me-9), 21.20 (C-23), 27.32 (C-3), 32.86 (C-2), 35.76 (C-5), 43.98 (C-4), 47.81 (C-20), 47.93 (C-7), 52.17 (C-1), 98.08 (C-13), 116.88 (C-22), 128.68 (C-14,18,16), 129.04 (C-19.15), 132.52 (C-21), 138.78 (C-17), 140.43 (C-12), 165.64 (C-11), 174.07 (C-6). HRMS (EI): *m*/*z* calcd for C_23_H_29_N_3_S: 379.2077; found 379.2073. [α]^D^_25_ *−*15.23 (c = 1.3 in CHCl_3_). IR (KBr, cm^−1^): ν 1656 (C=N) 1565 (C=N).

(*E*)-3-ethyl-4-p-tolyl-2-((*E*)-((1*R*,4*R*)-1,7,7-trimethylbicyclo[2.2.1]heptan-2-ylidene)hydrazono)-2,3-dihydrothiazole (**6c**), orange crystalline substance, yield 95% (0.35 g). ^1^H-NMR (CDCl_3_): δ_H_ 0.79 (3H, s, Me-9), 0.92 (3H, s, Me-8), 1.07 (3H, s, Me-10), 1.14 (3H, t, *J* = 7.2 Hz, Me-21), 1.19–1.29 (1H, m, H-3), 1.44–1.54 (1H, m, H-2), 1.67–1.76 (1H, m, H-2), 1.77–1.89 (2H, m, H-4, H-3), 2.12 (1H, d, *J* = 17.8 Hz, H-5), 2.37 (3H, s, H-22), 2.58 (1H, dt, *J* = 17.8 Hz, *J* = 3.8 Hz, H-5), 3.74 (2H, q, *J* = 7 Hz, H-20), 5.73 (1H, s, H-13), 7.20 (4H, s, H-15,16,18,19). ^13^C-NMR (CDCl_3_): δ_c_ 11.27 (Me-10), 13.06 (C-21), 18.79 and 19.64 (Me-8 and Me-9), 21.20 (C-22), 27.35 (C-3), 32.91 (C-2), 35.72 (C-5), 40.64 (C-20), 44.01 (C-4), 47.78 (C-7), 52.18 (C-1), 98.17 (C-13), 128.6 (C-14,18,16), 129.15 (C-19,15), 138.71 (C-17), 140.30 (C-12), 165.99 (C-11), 173.64 (C-6). HRMS (EI): *m*/*z* calcd for C_22_H_29_N_3_S: 367.2077; found 367.2080. [α]^D^_25_ *−*22.78 (c = 1.8 in CHCl_3_).

(*E*)-3-methyl-4-p-tolyl-2-((*E*)-((1*R*,4*R*)-1,7,7-trimethylbicyclo[2.2.1]heptan-2-ylidene)hydrazono)-2,3-dihydrothiazole (**6d**), orange crystalline substance, yield 96% (0.34 g). ^1^H-NMR (CDCl_3_): δ_H_ 0.79 (3H, s, Me-9), 0.92 (3H, s, Me-8), 1.06 (3H, s, Me-10), 1.19–1.28 (1H, m, H-3), 1.43–1.51 (1H, m, H-2), 1.67–1.76 (1H, m, H-2), 1.77–1.90 (2H, m, H-4, H-3), 2.13 (1H, d, *J* = 17.9 Hz, H-5), 2.37 (3H, s, H-21), 2.59 (1H, dt, *J* = 17.9 Hz, *J* = 3.9 Hz, H-5), 3.26 (3H, s, H-20), 5.80 (1H, s, H-13), 7.20 (4H, m, H-15,16,18,19). ^13^C-NMR (CDCl_3_): δ_c_ 11.26 (Me-10), 18.78 and 19.60 (Me-8 and Me-9), 21.19 (C-21), 27.32 (C-3), 32.92 (C-2), 33.36 (C-20), 35.70 (C-5), 44.03 (C-4), 47.73 (C-7), 52.28 (C-1), 98.7 (C-13), 128.41 (C-14,18,16), 129.22 (C-19,15), 138.8 (C-17), 140.62 (C-12), 167.4 (C-11), 173.8 (C-6),. HRMS (EI): *m*/*z* calcd for C_21_H_27_N_3_S: 353.1920; found 353.1917. [α]^D^_25_ *−*24.1 (c = 0.83 in CHCl_3_). IR (KBr, cm^−1^): ν 1656 (C=N), 1562 (C=N). M.p. 88.5 °C.

(*E*)-4-(4-methoxyphenyl)-3-phenyl-2-((*E*)-((1*R*,4*R*)-1,7,7-trimethylbicyclo[2.2.1]heptan-2-ylidene)hydrazono)-2,3-dihydrothiazole (**7a**), white crystalline substance, yield 50% (0.22 g). ^1^H-NMR (CDCl_3_): δ_H_ 0.75 (3H, s, Me-9), 0.89 (3H, s, Me-8), 1.05 (3H, s, Me-10), 1.11–1.25 (1H, m, H-3), 1.37–1.49 (1H, m, H-2), 1.60–1.84 (3H, m, H-2,3,4), 1.96 (1H, d, *J* = 18.1 Hz, H-5), 2.42 (1H, d, *J* = 17.9 Hz, H-5), 3.72 (3H, s, H-26), 5.94 (1H, s, H-13), 6.63–7.02 (4H, m, H-15,16,18,19), 7.11–7.30 (5H, m, H-21,22,23,24,25). ^13^C-NMR (CDCl_3_): δ_c_ 11.19 (Me-10), 18.75 and 19.61 (Me-8 and Me-9), 27.20 (C-3), 32.83 (C-2), 35.81 (C-5), 43.96 (C-4), 47.80 (C-7), 52.24 (C-1), 55.06 (C-26), 99.34 (C-13), 113.45 (C-16,18), 124.18 (C-14), 126.65 (C-23), 128.65 (C-25,21), 128.27 (C-18,16), 129.25 (C-22,24,15,19), 138.21 (C-20), 139.48 (C-12), 159.18 (C-17), 165.84 (C-11), 175.10 (C-6). HRMS (EI): *m*/*z* calcd for C_26_H_29_ON_3_S: 431.2026; found 431.2020. [α]^D^_25_ 0 (c = 0.7 in CHCl_3_). IR (KBr, cm^−1^): ν 1654 (C=N), 1556 (C=N), 1247 (C-O-C). M.p. 168.0–171.7 °C.

(*E*)-3-allyl-4-(4-methoxyphenyl)-2-((*E*)-((1*R*,4*R*)-1,7,7-trimethylbicyclo[2.2.1]heptan-2-ylidene)hydrazono)-2,3-dihydrothiazole (**7b**), yellow-white crystalline substance, yield 98% (0.39 g). ^1^H-NMR (CDCl_3_): δ_H_ 0.78 (3H, s, Me-9), 0.91 (3H, s, Me-8), 1.06 (3H, s, Me-10), 1.16–1.29 (1H, m, H-3), 1.41–1.53 (1H, m, H-2), 1.64–1.89 (3H, m, H-4, H-3, H-2), 2.08 (1H, d, *J* = 17.8 Hz, H-5), 2.54 (1H, dt, *J* = 18.1 Hz, *J* = 3.7 Hz, H-5), 3.82 (3H, s, H-23), 4.27 (2H, d, *J* = 4.9 Hz, H-20), 4.95–5.12 (2H, m, H-22), 5.74 (1H, s, H-13), 5.78–5.94 (1H, m, H-21), 6.86–7.29 (4H, m, H-15,16,18,19). ^13^C-NMR (CDCl_3_): δ_c_ 11.25 (Me-10), 18.79 and 19.63 (Me-8 and Me-9), 27.35 (C-3), 32.90 (C-2), 35.75 (C-5), 44.03 (C-4), 47.80 (C-20), 47.89 (C-7), 52.16 (C-1), 55.21 (C-23), 97.79 (C-13), 113.75 (C-18,16), 116.82 (C-22), 123.92 (C-14), 130.18 (C-19,15), 132.62 (C-21), 140.11 (C-12), 159.96 (C-17), 165.56 (C-11), 173.97 (C-6). HRMS (EI): *m*/*z* calcd for C_23_H_29_ON_3_S: 395.2026; found 395.2027. [α]^D^_25_ 0 (c = 0.82 in CHCl_3_). IR (KBr, cm^−1^): ν 1554 (C=N), 1245 (C-O-C). M.p. 110.7 °C.

(*E*)-3-ethyl-4-(4-methoxyphenyl)-2-((*E*)-((1*R*,4*R*)-1,7,7-trimethylbicyclo[2.2.1]heptan-2-ylidene)hydrazono)-2,3-dihydrothiazole (**7c**), white crystalline substance, yield 95% (0.36 g). ^1^H-NMR (CDCl_3_): δ_H_ 0.79 (3H, s, Me-9), 0.91 (3H, s, Me-8), 1.06 (3H, s, Me-10), 1.14 (3H, t, *J* = 7.1 Hz, Me-21), 1.20–1.27 (1H, m, H-3), 1.45–1.52 (1H, m, H-2), 1.68–1.75 (1H, m, H-2), 1.77–1.84 (1H, m, H-3), 1.87 (1H, t, *J* = 4.5 Hz, H-4), 2.10 (1H, d, *J* = 17.9 Hz, H-5), 2.56 (1H, dt, *J* = 17.7 Hz, *J* = 3.9 Hz, H-5), 3.73 (2H, q, *J* = 7.2 Hz, H-20), 3.91 (3H, s, H-22), 5.74 (1H, s, H-13), 6.90–7.52 (4H, m, H-15,16,18,19). ^13^C-NMR (CDCl_3_): δ_c_ 11.25 (Me-10), 13.04 (C-21), 18.79 and 19.63 (Me-8 and Me-9), 27.37 (C-3), 32.95 (C-2), 35.69 (C-5), 40.53 (C-20), 44.06 (C-4), 47.76 (C-7), 52.15 (C-1), 55.21 (C-22), 98.87 (C-13), 113.87 (C-18,16), 124.20 (C-14), 130.05 (C-19,15), 139.96 (C-12), 159.89 (C-17), 165.85 (C-11), 173.48 (C-6). HRMS (EI): *m*/*z* calcd for C_22_H_29_ON_3_S: 383.2026; found 383.2023. [α]^D^_25_ *−*36.16 (c = 1.77 in CHCl_3_). IR (KBr, cm^−1^): ν 1556 (C=N), 1247 (C-O-C). M.p. 40.0 °C.

(*E*)-4-(4-methoxyphenyl)-3-methyl-2-((*E*)-((1*R*,4*R*)-1,7,7-trimethylbicyclo[2.2.1]heptan-2-ylidene)hydrazono)-2,3-dihydrothiazole (**7d**), white crystalline substance, yield 95% (0.35 g). ^1^H-NMR (CDCl_3_): δ_H_ 0.79 (3H, s, Me-9), 0.92 (3H, s, Me-8), 1.06 (3H, s, Me-10), 1.21–1.27 (1H, m, H-3), 1.45–1.51 (1H, m, H-2), 1.68–1.75 (1H, m, H-2), 1.79–1.85 (1H, m, H-3), 1.88 (1H, t, *J* = 4.6 Hz, H-4), 2.14 (1H, d, *J* = 17.9 Hz, H-5), 2.59 (1H, dt, *J* = 17.8, *J* = 4 Hz, H-5), 3.25 (3H, s, H-20), 3.82 (3H, s, H-21), 5.77 (1H, s, H-13), 6.90–7.27 (4H, m, H-15,16,18,19). ^13^C-NMR (CDCl_3_): δ_c_ 11.26 (Me-10), 18.80 and 19.61 (Me-8 and Me-9), 27.36 (C-3), 32.98 (C-2), 33.18 (C-20), 35.68 (C-5), 44.10 (C-4), 47.74 (C-7), 52.27 (C-1), 55.25 (C-21), 97.78 (C-13), 113.97 (C-18,16), 123.80 (C-14), 129.90 (C-19,15), 140.30 (C-12), 159.96 (C-17), 167.39 (C-11), 173.51 (C-6). HRMS (EI): *m*/*z* calcd for C_21_H_27_ON_3_S: 369.1869; found 369.1868. M.p. 103.4 °C.

(*E*)-3,4-diphenyl-2-((*E*)-((1*R*,4*R*)-1,7,7-trimethylbicyclo[2.2.1]heptan-2-ylidene)hydrazono)-2,3-dihydrothiazole (**8a**), orange crystalline substance, yield 85% (0.34 g). ^1^H-NMR (CDCl_3_): δ_H_ 0.75 (3H, s, Me-9), 0.89 (3H, s, Me-8), 1.05 (3H, s, Me-10), 1.13–1.20 (1H, m, H-3), 1.39–1.47 (1H, m, H-2), 1.64–1.72 (1H, m, H-2), 1.72–1.82 (2H, m, H-4,3), 1.97 (1H, d, *J* = 17.9 Hz, H-5), 2.42 (1H, dt, *J* = 18.2 Hz, *J* = 4.3 Hz, H-5), 6.04 (1H, s, H-13), 7.04–7.26 (10H, m, H-15,16,17,18,19,21,22,23,24,25). ^13^C-NMR (CDCl_3_): δ_c_ 11.19 (Me-10), 18.72 and 19.69 (Me-8 and Me-9), 27.15 (C-3), 32.76 (C-2), 35.85 (C-5), 43.87 (C-4), 47.80 (C-7), 52.28 (C-1), 100.79 (C-13), 126.78 (C-23), 127.87 (C-17,20,21), 128.02 (C-15,19), 128.11(C-16,18), 128.27, (C-24,22), 131.50 (C-14), 137.95 (C-20), 139.77 (C-12), 165.92 (C-11), 175.46 (C-6). HRMS (EI): *m*/*z* calcd for C_25_H_27_N_3_S: 401.1920; found 401.1917. [α]^D^_25_ *−*7.58 (c = 0.66 in CHCl_3_). IR (KBr, cm^−1^): ν 1654 (C=N) 1554 (C=N).

(*E*)-3-allyl-4-phenyl-2-((*E*)-((1*R*,4*R*)-1,7,7-trimethylbicyclo[2.2.1]heptan-2-ylidene)hydrazono)-2,3-dihydrothiazole (**8b**), orange-brown amorphous substance, yield 95% (0.35 g). ^1^H-NMR (CDCl_3_): δ_H_ 0.78 (3H, s, Me-9), 0.91 (3H, s, Me-8), 1.06 (3H, s, Me-10), 1.19–1.26 (1H, m, H-3), 1.44–1.51 (1H, m, H-2), 1.67–1.75 (1H, m, H-2), 1.77–1.84 (1H, m, H-3), 1.86 (1H, t, *J* = 4.4 Hz, H-4), 2.08 (1H, d, *J* = 18 Hz, H-5), 2.54 (1H, dt, *J* = 18 Hz, *J* = 3.8 Hz, H-5), 4.29 (2H, d, *J* = 5.7 Hz, H-20), 4.96–5.10 (2H, m, H-22), 5.80 (1H, s, H-13), 5.81–5.91 (1H, m, H-21), 7.31–7.40 (5H, m, H-15,16,17,18,19). ^13^C-NMR (CDCl_3_): δ_c_ 10.94 (Me-10), 18.47 and 19.31 (Me-8 and Me-9), 27.01 (C-3), 32.54 (C-2), 35.44 (C-5), 43.65 (C-4), 47.49 (C-20), 47.66 (C-7), 51.85 (C-1), 98.25 (C-13), 116.62 (C-22), 128,04 (C-19,15,17), 128.44 (C-18,16), 131.23 (C-14), 132.14 (C-21), 140.06 (C-12), 165.32 (C-11), 173.87 (C-6). HRMS (EI): *m*/*z* calcd for C_22_H_27_N_3_S: 365.1920; found 365.1917. [α]^D^_25_ *−*10.82 (c = 1.94 in CHCl_3_). IR (KBr, cm^−1^): ν 1653 (C=N), 1554 (C=N).

(*E*)-3-ethyl-4-phenyl-2-((*E*)-((1*R*,4*R*)-1,7,7-trimethylbicyclo[2.2.1]heptan-2-ylidene)hydrazono)-2,3-dihydrothiazole (**8c**), orange-brown amorphous substance, yield 97% (0.34 g). ^1^H-NMR (CDCl_3_): δ_H_ 0.79 (3H, s, Me-9), 0.92 (3H, s, Me-8), 1.07 (3H, s, Me-10), 1.15 (3H, m, Me-21), 1.20–1.27 (1H, m, H-3), 1.45–1.52 (1H, m, H-2), 1.68–1.75 (1H, m, H-2), 1.78–1.85 (1H, m, H-3), 1.87 (1H, t, *J* = 4.4 Hz, H-4), 2.12 (1H, d, *J* = 17.8 Hz, H-5), 2.58 (1H, dt, *J* = 17.9 Hz, *J* = 3.6 Hz, H-5), 3.75 (2H, q, *J* = 6.8 Hz, H-20), 5.78 (1H, s, H-13), 7.30–7.42 (5H, m, H-15, H-16, H-17, H-18, H-19). ^13^C-NMR (CDCl_3_): δ_c_ 11.25 (Me-10), 13.03 (C-21), 18.77 and 19.62 (Me-8 and Me-9), 27.31 (C-3), 32.87 (C-2), 35.74 (C-5), 40.72 (C-20), 43.96 (C-4), 47.77 (C-7), 52.20 (C-1), 98.73 (C-13), 128.72 (C-17), 128.65 (C-18,16), 128.47 (C-19,15), 131.73 (C-14), 140.28 (C-12), 166.02 (C-11), 173.86 (C-6). HRMS (EI): *m*/*z* calcd for C_21_H_27_N_3_S: 353.1920; found 353.1922. [α]^D^_25_ *−*21.92 (c = 1.46 in CHCl_3_). IR (KBr, cm^−1^): ν 1653 (C=N), 1598 (C=N).

(*E*)-3-methyl-4-phenyl-2-((*E*)-((1*R*,4*R*)-1,7,7-trimethylbicyclo[2.2.1]heptan-2-ylidene)hydrazono)-2,3-dihydrothiazole (**8d**), orange-brown amorphous substance, yield 60% (0.20 g). ^1^H-NMR (CDCl_3_): δ_H_ 0.79 (3H, s, Me-9), 0.92 (3H, s, Me-8), 1.06 (3H, s, Me-10), 1.19–1.30 (1H, m, H-3), 1.42–1.52 (1H, m, H-2), 1.66–1.85 (2H, m, H-2, H-3), 1.89 (1H, t, *J* = 4.3 Hz, H-4), 2.13 (1H, d, *J* = 18 Hz, H-5), 2.59 (1H, dt, *J* = 18 Hz, *J* = 3.8 Hz, H-5), 3.27 (3H, s, Me-20), 5.84 (1H, s, H-13), 7.29–7.44 (5H, m, H-15,16,17,18,19). ^13^C-NMR (CDCl_3_): δ_c_ 11.25 (Me-10), 18.77 and 19.59 (Me-8 and Me-9), 27.31 (C-3), 32.90 (C-2), 33.35 (C-20), 35.67 (C-5), 43.99 (C-4), 47.72 (C-7), 52.25 (C-1), 98.67 (C-13), 128.74 (C-17), 128.54 (C-16,18), 128.45 (C-19,15), 131.32 (C-14), 140.54 (C-12), 167.46 (C-11), 173.74 (C-6). HRMS (EI): *m*/*z* calcd for C_20_H_25_N_3_S: 339.1764; found 339.1761. [α]^D^_25_ *−*23.19 (c = 1.38 in CHCl_3_). IR (KBr, cm^−1^): ν 1656 (C=N), 1556 (C=N).

### 3.2. Biological Studies

#### 3.2.1. Evaluation of the Anti-Vaccinia Virus Activity

A typical representative of orthopoxviruses, the vaccinia virus (strain Copenhagen), obtained from the State Collection of Virus Infection and Rickettsiosis Agents of VECTOR, was used in the work. The virus was grown in Vero cell culture. The virus concentration in the culture liquid was determined by plaque titration in Vero cell culture, calculated and expressed in decimal logarithms of plaque-forming units in 1 mL (log_10_ PFU/mL). The concentration of the virus in the samples used in the work was from 5.6 to 6.1 log_10_ PFU/mL. The series of virus with the indicated titre was stored and used when working at −70 °C.

The antiviral efficacy of compounds was evaluated as follows. In wells of 96-well plates, containing a monolayer of Vero cells in 100 μL of DMEM medium with 2% fetal serum, first 50 μL of serial dilutions of the test compounds were introduced and then 50 μL of dilution of orthopoxvirus at a dose of 1000 PFU/well. The toxicity of the compounds was determined by the Vero cell death caused by the drug in the wells of the plate, to which the virus was not introduced. Monolayers of cells were used as controls in the wells of the plate, into which virus without compounds was added (virus control), and monolayers of cells in wells into which neither the virus nor the compound were introduced (cell-culture control). After incubation for 4 days, the monolayer of cells was stained with vital dye neutral red for 2 h. After removing the dye and washing the wells from its unbound fraction, a lysis buffer was added. The amount of dye adsorbed by the living cells of the monolayer was evaluated by optical density (OD), which is an indication of the number of cells undisturbed under the influence of the virus in a monolayer. The OD was measured on an Emax spectrophotometer (Molecular Devices, San Jose, CA, USA) at a wavelength of 490 nm. Results were processed using the Soft Max Pro 4.0 program, which computed the 50% toxic concentration (CC_50_ in μM) and 50% inhibitory concentration (IC_50_ in μM). The selectivity index (SI) was determined as SI = CC_50_/IC_50_ using the corresponding concentrations.

#### 3.2.2. Plasmids

The plasmid ph-GPM containing the Marburg virus glycoprotein (Popp strain) was obtained by inserting the synthesized gene into the vector phMGFP. Prior to synthesis, codon optimization of the GP gene was performed.

#### 3.2.3. Cytotoxicity Assays

MTT reduction was used to study cytotoxicity of the compounds [D.M. Morgan, Tetrazolium (MTT) assay for cellular viability and activity, Methods Mol. Biol. 79 (1998) 179–183.] Briefly, series of two-fold dilutions of each compound (15.6–1000 μM) in 10% DMEM were prepared in 96-well plates. HEK293FT cells (100 mL at a density of 105 cells per ml) were added and incubated for 48 h at 37 °C in 5% CO_2_. Then, 20 mL (1/10 vol) of a solution of 3-(4,5-dimethylthiazolyl-2)-2,5-diphenyltetrazolium bromide (Sigma) (5 mg/mL) in phosphate-buffered saline was added to each well. After 2 h incubation, the solution was removed from the wells, and DMSO (100 mL per well) was added to dissolve the formazan crystals. The optical density of the cells was then measured on a Model 680 Microplate (Bio-Rad) Readerat 535 nm and plotted against the concentration of the compounds. Each concentration was tested in three parallels. The 50% cytotoxic dose (CC50) of each compound (i.e., the compound concentration that causes the death of 50% cells in a culture, or decreasing the optical density twice as compared to the control wells) was calculated from the data obtained.

#### 3.2.4. Production of Pseudoviruses Based on the Recombinant Vesicular Stomatitis Virus (VSV) Exposing Marburg Virus Glycoprotein on Their Surface

HEK293FT cells were maintained in DMEM (Invitrogen) containing 10% fetal bovine serum (FBS; Gibco), 0.6 mg/mL L-glutamine (Invitrogen), and 50 mg/mL gentamicin. To obtain pseudoviruses exposing Marburg virus glycoprotein (Popp strain) on its surface, HEK293FT cells grown in a T75 were transfected with a plasmid containing the gene of this glycoprotein (ph-GPM) (23 mg of plasmid per cell monolayer in a T75 culture vial). After 24 h from the transfection, a suspension of VSV of the firefly luciferase gene pseudotyped by surface VSV glycoprotein G (rVSV-ΔG-G) was added to the cells (5 μL, ~10^6^ of the transducing units). Six hours after the infection, the infecting pseudovirus was washed off, and the culture medium was replaced with the fresh one. Pseudoviruses were harvested after 48 h by filtering the culture medium through a 0.45-mm filter, after centrifugation to remove cell debris. Pseudoviruses were stored at −80 °C, and their functional activity was determined using a HEK293T cell culture, with the luminescence level recorded using a Stat Fax 4400 luminometer.

#### 3.2.5. Determination of Half Inhibitory Concentrations of the Compounds against the rVSV-ΔG-GPM Pseudoviruses and Calculation of Therapeutic Index (SI) Values

To estimate the contagious ability of pseudoviruses, the targeting cells of HEK293 line seeded into 96-well plates at a monolayer density of 80–90% were used. The infectivity of pseudoviruses in the presence of inhibitors and in the control (non-inhibited) samples was determined according to the luminescence index, measured 24 h after the infection. All the measurements were performed three times determining the average value and standard deviation. The half maximal inhibitory concentrations (IC_50_) against pVSV-ΔG-GPM pseudoviruses were determined for the studied compounds. For each compound, the selectivity index (SI) was, consequently, calculated as the ratio of cytotoxicity of the compound and its inhibitory activity against the virus (CC_50_/IC_50_).

## 4. Conclusions

As a result of our studies, we have synthesized a library of derivatives based on (+)- and (−)-camphor, which describes compounds containing a bornane backbone and fragments of thiosemicarbazone, 2-iminothiazolidin-4-one, and 2,3-dihydrothiazole. Among the obtained compounds, 32 were obtained for the first time; their structure was established using ^1^H, ^13^C NMR spectroscopy, high-resolution mass spectroscopy, IR, and X-ray diffraction. The synthesized compounds were tested against the vaccinia virus and the pseudovirus system, with the surface glycoproteins of the Marburg virus. Among them, effective inhibitors of these viruses were found, and some compounds were active against both viruses. Thus, it was shown that these camphor derivatives represent a promising class of antiviral agents active against a wide range of viruses.

## Figures and Tables

**Table 1:** Structures of synthesized compounds and reaction yields.

Compound	Camphor	Nitrogen-Containing Fragment	R_1_	R_2_	Yield, %
**2a**	(+)	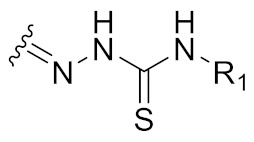	Ph	-	90
**2b**	(+)		Allyl	-	60
**2c**	(+)		Et	-	50
**2d**	(+)		Me	-	60
**2′a**	(−)		Ph	-	85
**2′b**	(−)		Allyl	-	55
**2′c**	(−)		Et	-	70
**2′d**	(−)		Me	-	65
**3a**	(+)	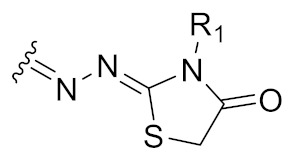	Ph	-	60
**3b**	(+)		Allyl	-	50
**3c**	(+)		Et	-	45
**3d**	(+)		Me	-	60
**3′a**	(−)		Ph	-	45 *
**3′b**	(−)		Allyl	-	60
**3′c**	(−)		Et	-	70
**3′d**	(−)		Me	-	60 *
**4a**	(+)	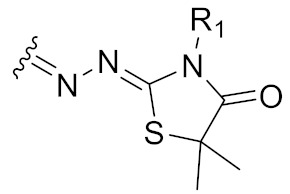	Ph	-	50 *
**4b**	(+)		Allyl	-	50 *
**4c**	(+)		Et	-	60 *
**4d**	(+)		Me	-	70
**5a**	(+)	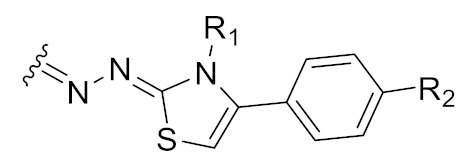	Ph	Cl	55 *
**5b**	(+)		Allyl	Cl	90
**5c**	(+)		Et	Cl	70
**5d**	(+)		Me	Cl	90
**6a**	(+)		Ph	Me	95
**6b**	(+)		Allyl	Me	97
**6c**	(+)		Et	Me	95
**6d**	(+)		Me	Me	96
**7a**	(+)		Ph	OMe	50 *
**7b**	(+)		Allyl	OMe	98
**7c**	(+)		Et	OMe	95
**7d**	(+)		Me	OMe	95
**8a**	(+)		Ph	H	85
**8b**	(+)		Allyl	H	95
**8c**	(+)		Et	H	97
**8d**	(+)		Me	H	60 *

* The product yield is given after purification by column chromatography.

**Table 2:** Antiviral activity of synthesized compounds against VV.

Compound	CC_50_^a^ μM	IC_50_^b^ μM	SI ^c^ (CC_50_/IC_50_)
**2a**	24 ± 3.4	6.7 ± 0.9	4
**2b**	200 ± 23.8	NA ^d^	-
**2c**	NT ^e^	NT	NT
**2d**	150 ± 17.9	97 ± 19.8	2
**2′a**	16 ± 2.8	4.3 ± 1.8	4
**2′b**	35 ± 4.6	12 ± 2.9	3
**2′c**	45 ± 6.6	14 ± 2.6	3
**2′d**	130 ± 14.2	35 ± 5.8	5
**3a**	290 ± 39.1	NA	-
**3b**	99 ± 11.8	72 ± 12.2	-
**3c**	87 ± 10.1	24 ± 3.9	4
**3d**	310 ± 41.3	100 ± 17.6	3
**3′a**	290 ± 36.9	75 ± 13.7	4
**3′b**	160 ± 20.8	66 ± 12.9	2
**3′c**	220 ± 28.6	64 ± 12.3	4
**3′d**	250 ± 31.4	NA	-
**4a**	270 ± 35.2	32.5 ± 2.8	8
**4b**	NT	NT	NT
**4c**	290 ± 34.9	NA	-
**4d**	310 ± 38.7	12.7 ± 1.4	24
**5a**	229 ± 24.7	NA	-
**5b**	19 ± 2.8	2.5 ± 0.3	8
**5c**	70 ± 8.9	12 ± 1.5	6
**5d**	144 ± 18.7	2.6 ± 0.2	55
**6a**	98 ± 11.6	NA	-
**6b**	12 ± 2.1	4 ± 0.6	3
**6c**	15 ± 2.9	NA	-
**6d**	17 ± 3.1	5.1 ± 0.7	3
**7a**	232 ± 29.6	NA	-
**7b**	200 ± 23.1	NA	-
**7c**	NT	NT	NT
**7d**	NT	NT	NT
**8a**	122 ± 14.7	18 ± 1.9	7
**8b**	17 ± 2.9	3 ± 0.4	6
**8c**	17 ± 3.3	3 ± 0.5	6
**8d**	14 ± 2.4	1.5 ± 0.2	9
Cidofovir	475.3 ± 74.9	40.1 ± 2.8	12

^a^ CC_50_—50% cytotoxic concentration, at which 50% of cells in an uninfected monolayer are destroyed; ^b^ IC_50_—50% virus-inhibiting concentration, at which 50% of the cells in the infected monolayer are not destroyed (retain viability); ^c^ SI—selectivity index, SI = CC_50_/IC_50_; ^d^ NA—not active; ^e^ NT—not tested; CC_50_ and IC_50_ values are presented as M ± SD, where M is the mean value, SD is the standard deviation with the number of measurements *n* = 3.

**Table 3:** Antiviral activity against Marburg pseudovirus (GP MARV).

Compound	CC_50_^a^ μM	IC_50_^b^ μM	SI ^c^ (CC_50_/IC_50_)
**2a**	700.0 ± 43.1	398.1 ± 33.2	2
**2b**	128.3 ± 37.8	24.5 ± 3.8	5
**2c**	NT ^d^	NT	NT
**2d**	62.8 ± 17.1	28.0 ± 4.2	-
**2′a**	280.5 ± 38.6	5.9 ± 2.1	47
**2′b**	56.5 ± 8.2	3.8 ± 1.9	15
**2′c**	529.6 ± 35.4	9.1 ± 3.9	16
**2′d**	66.9 ± 18.2	29.2 ± 4.2	2
**3a**	1259.3 ± 52.7	237.2 ± 5.9	5
**3b**	108.0 ± 6.5	22.9 ± 0.7	5
**3c**	54.1 ± 9.2	30.7 ± 6.8	2
**3d**	NT	NT	NT
**3′a**	NT	NT	NT
**3′b**	121.1 ± 6.5	45.8 ± 6.5	3
**3′c**	170.4 ± 40.9	68.2 ± 20.4	3
**3′d**	182.5 ± 14.3	28.6 ± 10.7	6
**4a**	324.7 ± 36.1	94.7 ± 8.1	3
**4b**	959.5 ± 6.8	254.9 ± 45.0	4
**4c**	777.7 ± 82.8	171.1 ± 46.7	5
**4d**	513.9 ± 34.1	39.0 ± 3.3	13
**5a**	871.5 ± 52.8	103.2 ± 6.9	8
**5b**	80.0 ± 20.0	30.0 ± 10.0	3
**5c**	51.6 ± 15.5	25.8 ± 5.2	2
**5d**	107.0 ± 5.3	10.7 ± 2.7	10
**6a**	134.7 ± 19.2	60.2 ± 14.4	2
**6b**	39.5 ± 4.8	5.3 ± 2.6	7
**6c**	NT	NT	NT
**6d**	79.2 ± 8.5	28.3 ± 8.5	3
**7a**	973.1 ± 61.6	127.4 ± 23.2	8
**7b**	37.9 ± 8.3	13.9 ± 2.5	3
**7c**	117.3 ± 10.4	13.0 ± 5.2	9
**7d**	97.4 ± 21.6	27.1 ± 8.1	4
**8a**	89.6 ± 19.9	29.9 ± 5.0	3
**8b**	<41.0	19.2 ± 2.7	<2
**8c**	<42.4	28.3 ± 2.8	<2
**8d**	<44.2	26.5 ± 5.9	<2
Sertraline	408 ± 35.9	3.5 + 2.0	116

^a^ CC_50_—50% cytotoxic concentration, at which 50% of cells in an uninfected monolayer are destroyed; ^b^ IC_50_—50% virus-inhibiting concentration that causes death of 50% of cells infected with pseudovirus particles, which display GP belonging to the Marburg (MARV) virus on their surface; ^c^ SI—selectivity index, SI = CC_50_/IC_50_; ^d^ NT—not tested; CC_50_ and IC_50_ values are presented as M ± SD, where M is the mean value, SD is the standard deviation with the number of measurements *n* = 3.

## Data Availability

Not applicable.

## Supplementary Materials

The following supporting information can be downloaded at: https://www.mdpi.com/article/10.3390/molecules27154761/s1. ^1^H NMR and ^13^C NMR spectra of compounds **2a-8d**; High Resolution Mass Spectra of Compounds **2a-8d**.

## Sample Availability

Samples of the compounds are not available from the authors.
