# Supplementary material for: Synthesis and Antiviral Properties of Camphor-Derived Iminothiazolidine-4-Ones and 2,3-Dihydrothiazoles
Source: Molecules. 2022 Jul 25;27(15):4761. doi: 10.3390/molecules27154761 (PMC9331314; doi:10.3390/molecules27154761)

Synthesis and Antiviral Properties of Camphor-Derived Iminothiazolidine-4-Ones and 2,3-Dihydrothiazoles

Vladislav V. Oreshko [1,2], Kseniya S. Kovaleva [1], Ekaterina D. Mordvinova [2,3], Olga I. Yarovaya [1,2,*], Yuri V. Gatilov [1], Dmitry N. Shcherbakov [3], Nikolai I. Bormotov [3], Olga A. Serova [3], Larisa N. Shishkina [3] and Nariman F. Salakhutdinov [1]

[1] Department of Medicinal Chemistry, N. N. Vorozhtsov Novosibirsk Institute of Organic Chemistry, Siberian Branch of the Russian Academy of Sciences, Lavrentiev Ave. 9, 630090 Novosibirsk, Russia; vladoreshko28121999@gmail.com (V.V.O.); kseniya.kovaleva3103@yandex.ru (K.S.K.); gatilov@nioch.nsc.ru (Y.V.G.); anvar@nioch.nsc.ru (N.F.S.)

[2] Zelman Institute for Medicine and Psychology, Novosibirsk State University, Pirogova St., 1, 630090 Novosibirsk, Russia; mordvinova97@mail.ru

[3] State Research Center of Virology and Biotechnology VECTOR, Rospotrebnadzor, 630559 Koltsovo, Russia; dnshcherbakov@gmail.com (D.N.S.); bormotov_ni@vector.nsc.ru (N.I.B.); serova.olga.a@yandex.ru (O.A.S.); shish@vector.nsc.ru (L.N.S.)

* Correspondence: ooo@nioch.nsc.ru; Tel.: +7-383-330-88-70

Table of content

1. ^{1}H NMR and ^{13}C NMR spectra of compounds 2a-8d 2

2. High Resolution Mass Spectra of Compounds 2a-8d 38

1. ^{1}H NMR and ^{13}C NMR spectra of compounds 2a-8d

1.1. ^{1}H NMR and ^{13}C NMR spectra of compound 2a

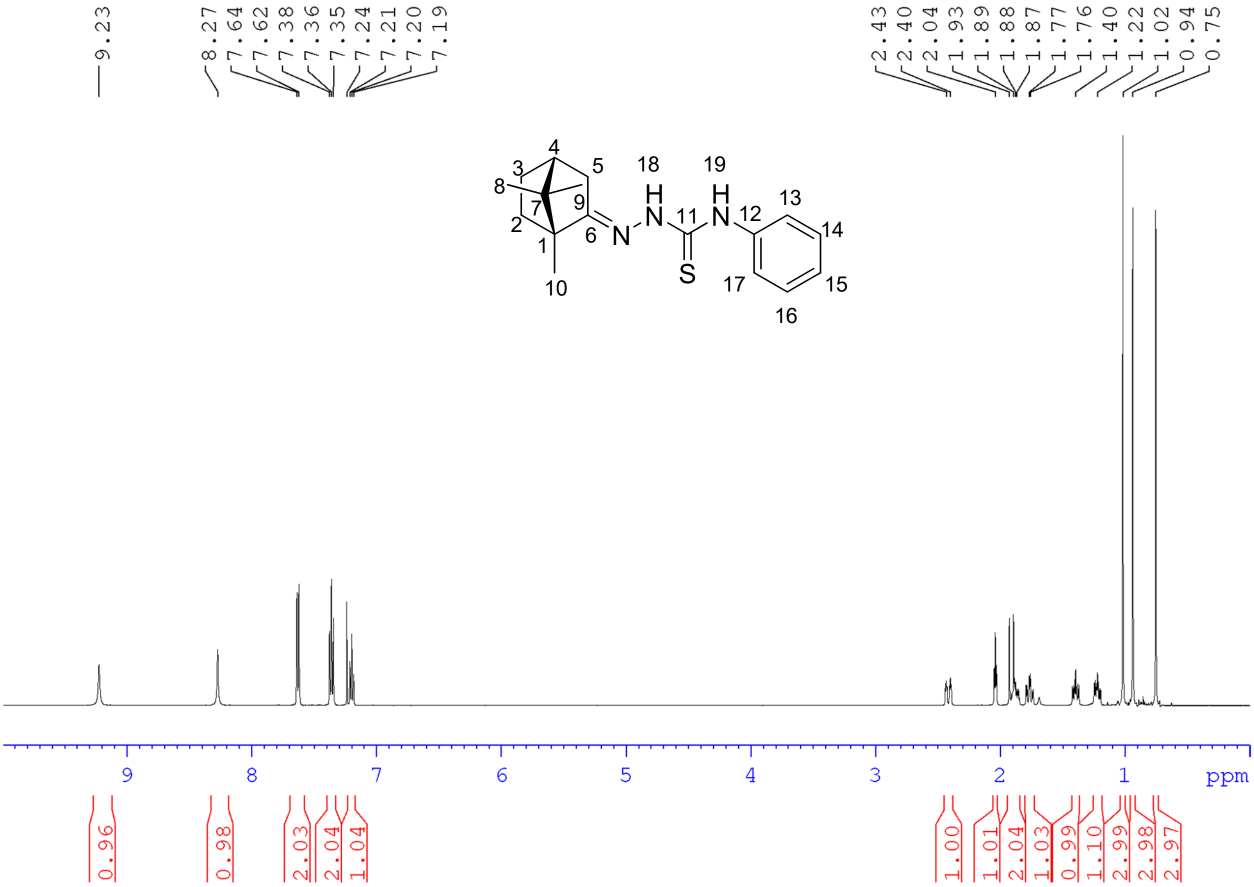

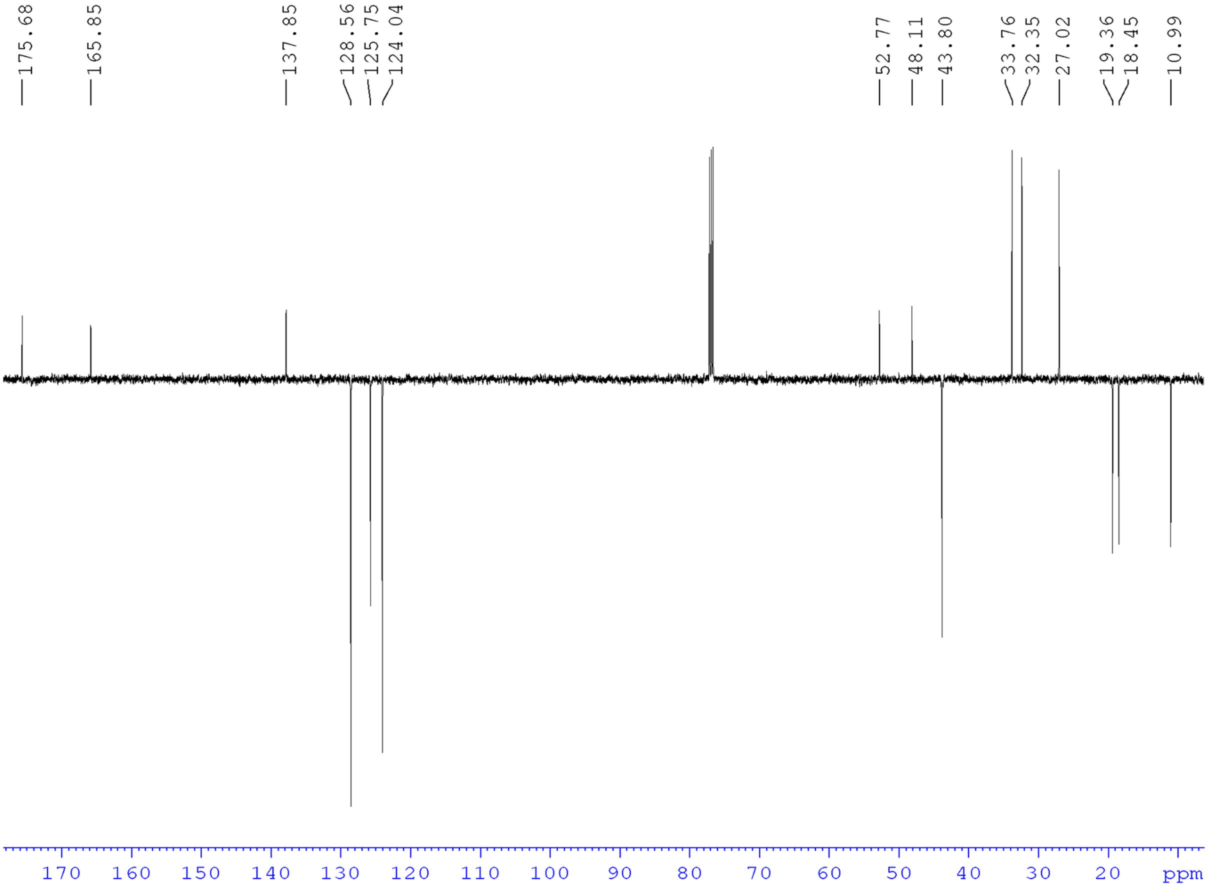

1.2. ¹H NMR and ¹³C NMR spectra of compound 2b

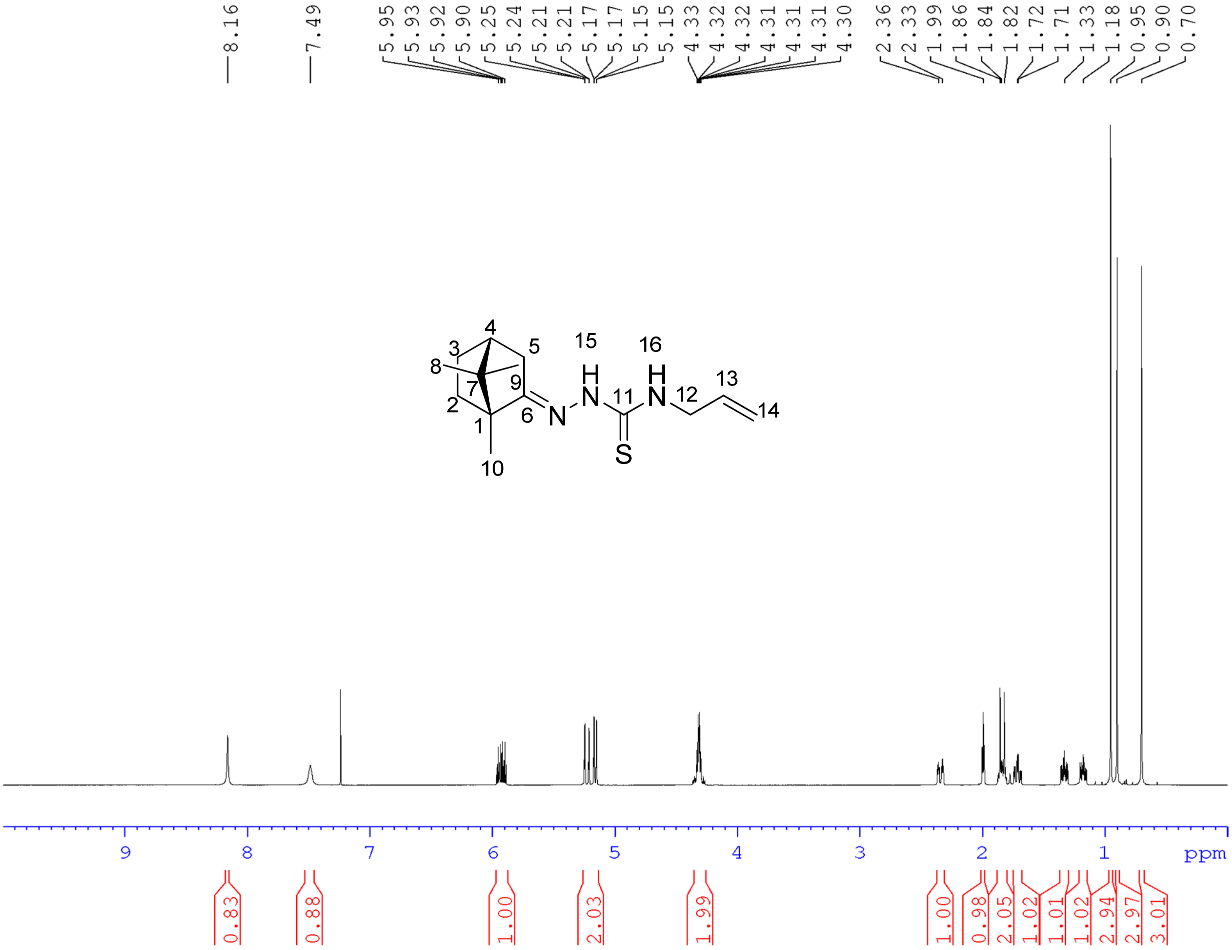

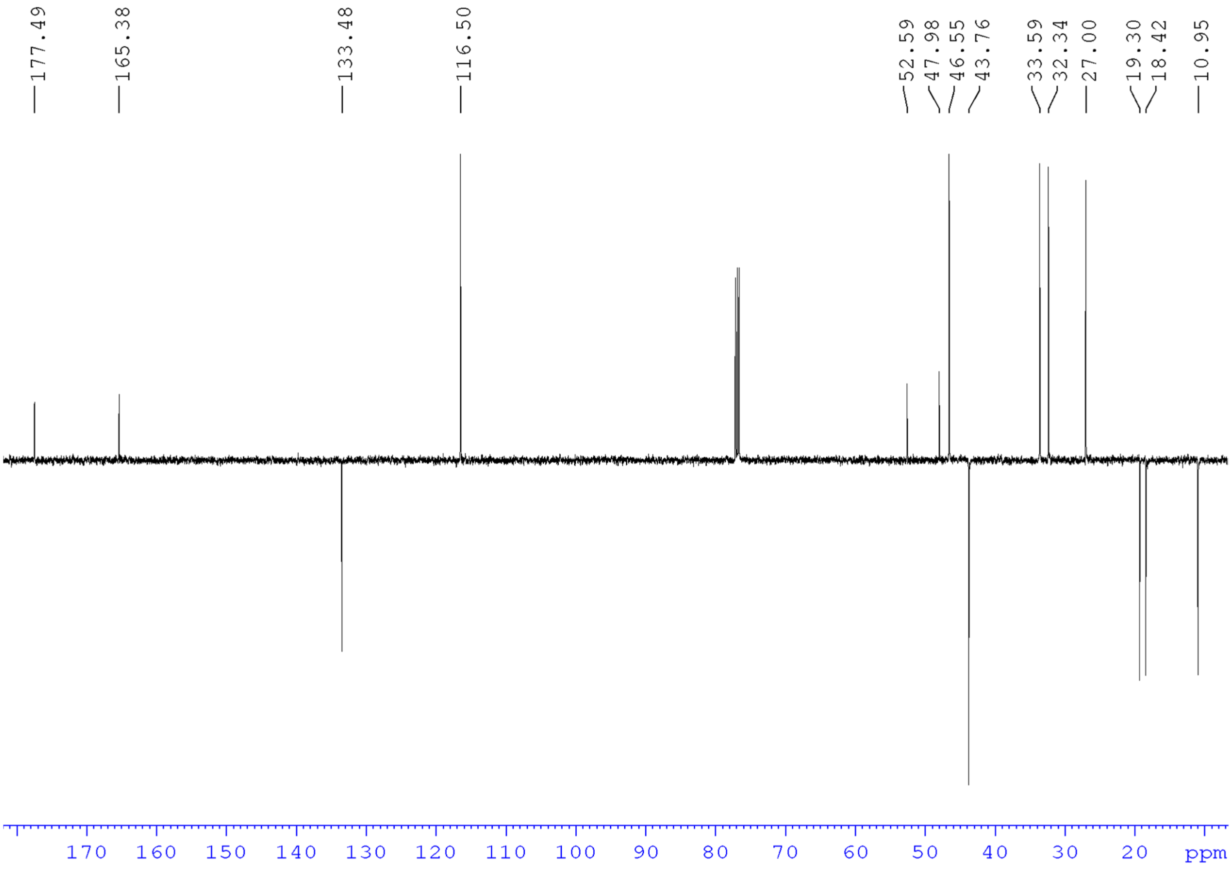

1.3. ^{1}H NMR and ^{13}C NMR spectra of compound 2c

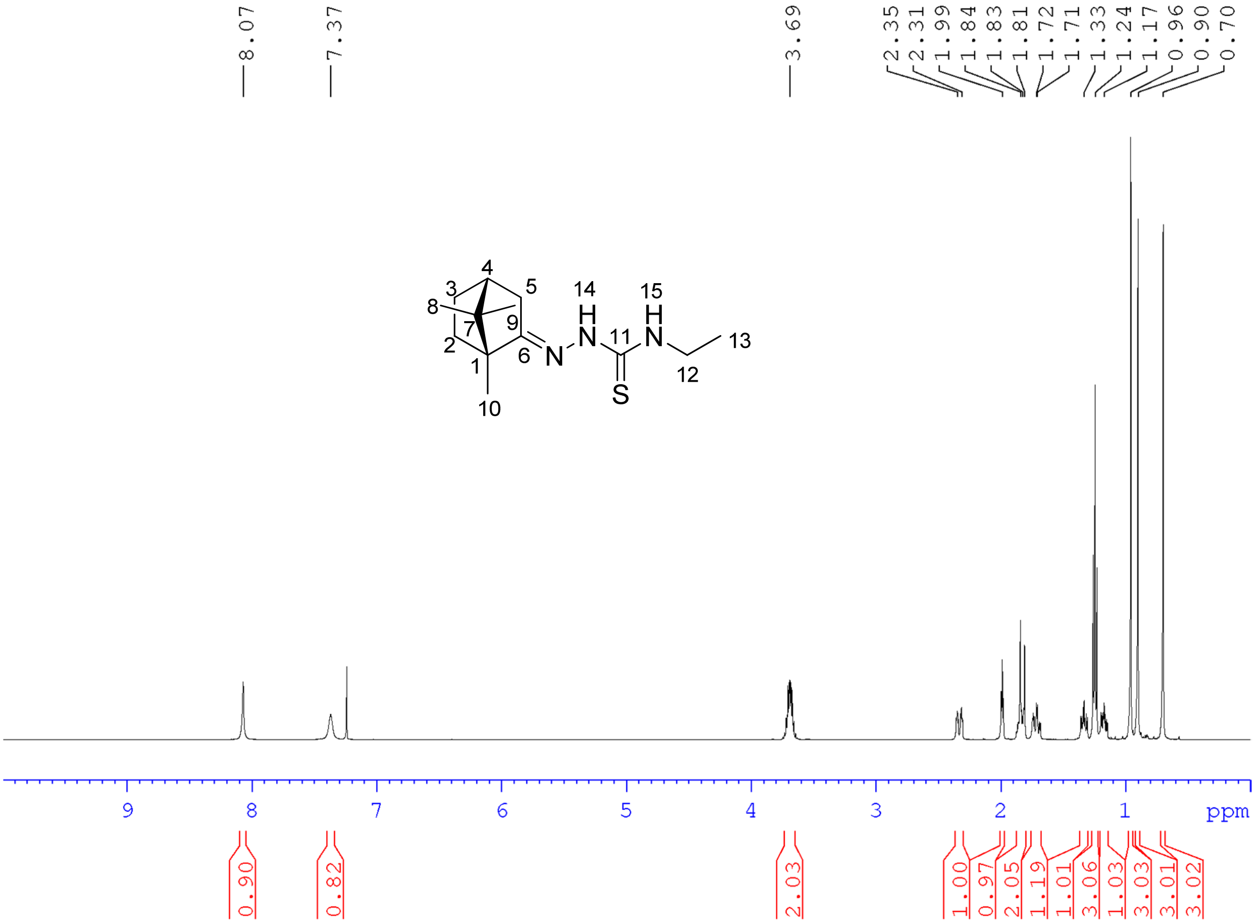

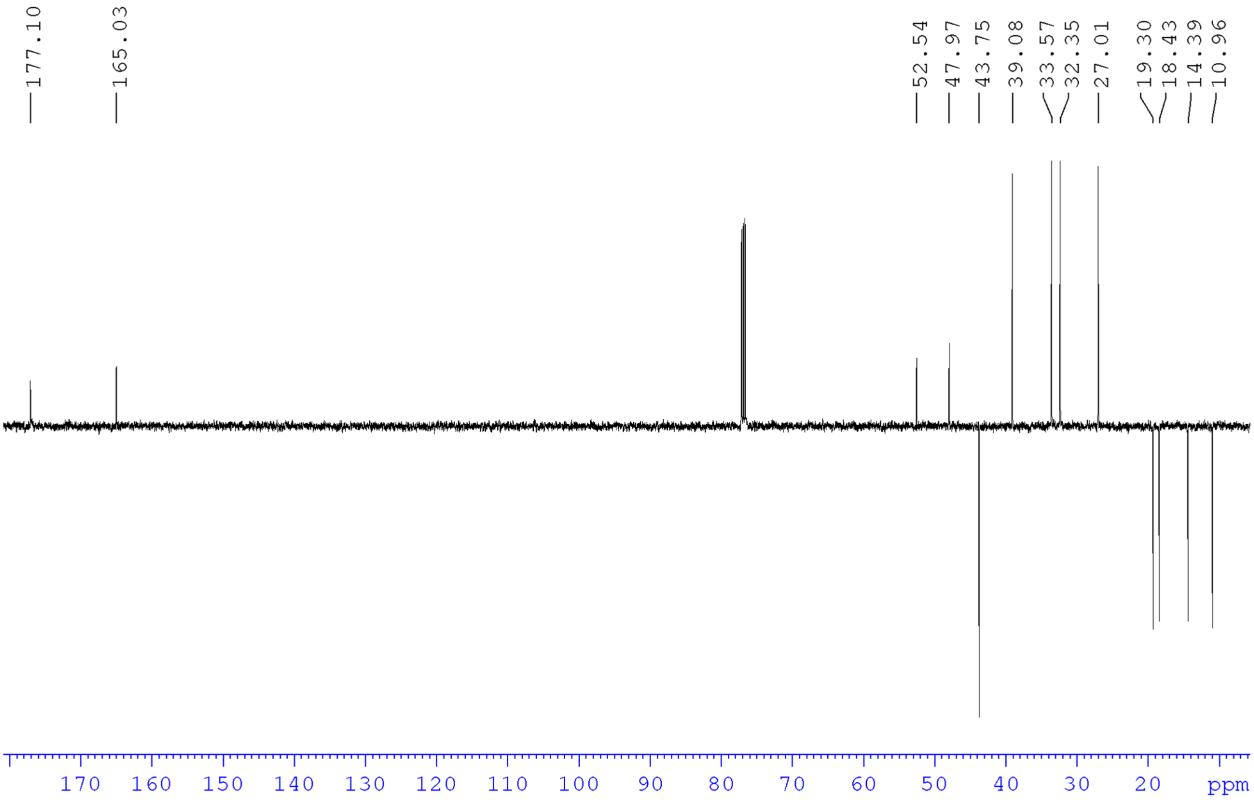

1.4. ^{1}H NMR and ^{13}C NMR spectra of compound 2d

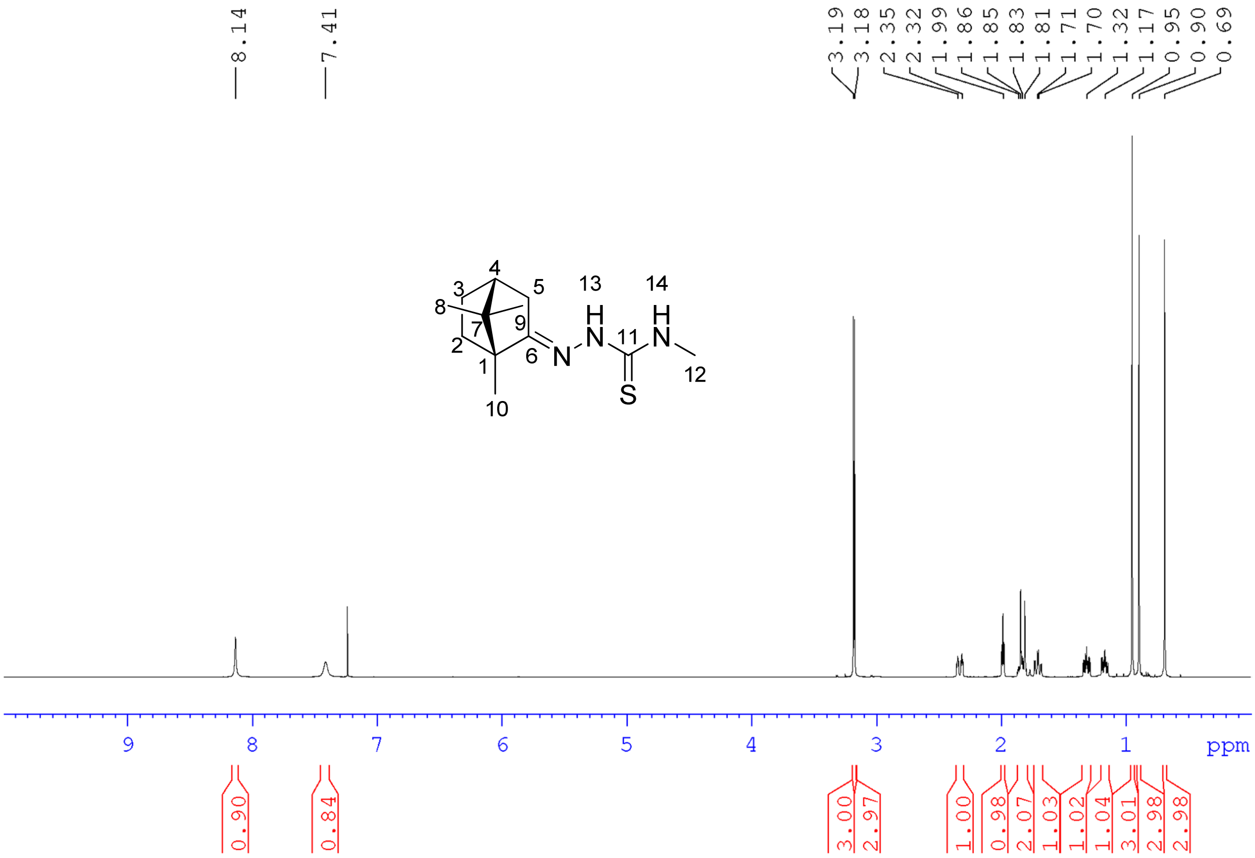

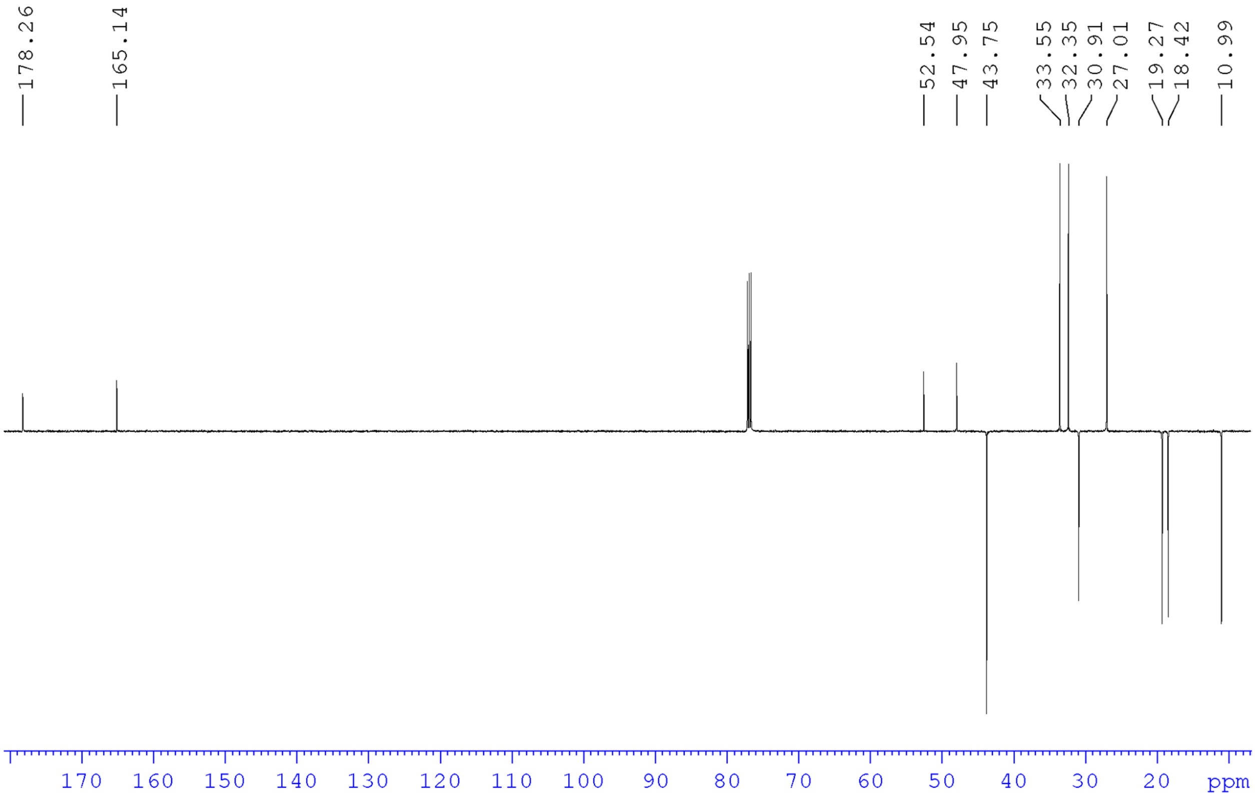

1.5. ^{1}H NMR and ^{13}C NMR spectra of compound 2′a

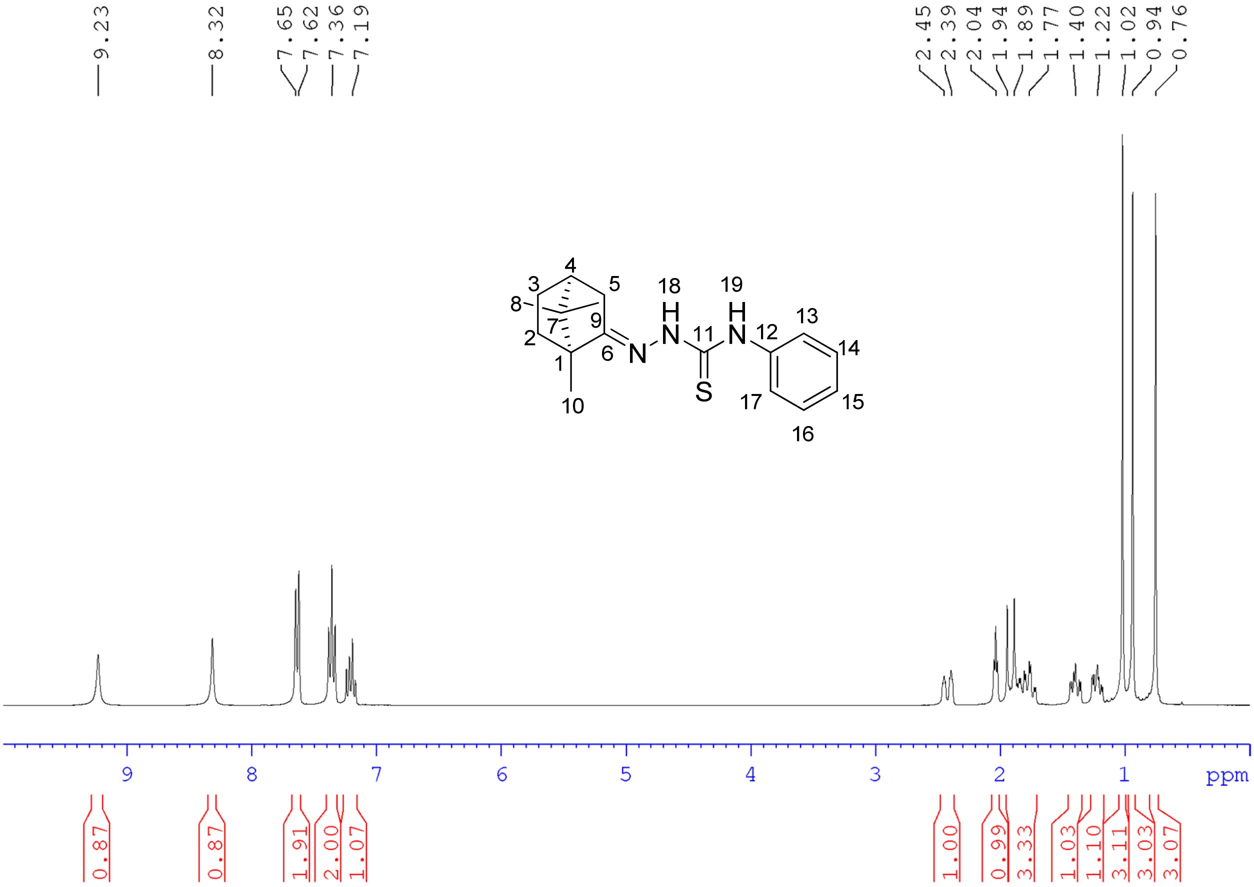

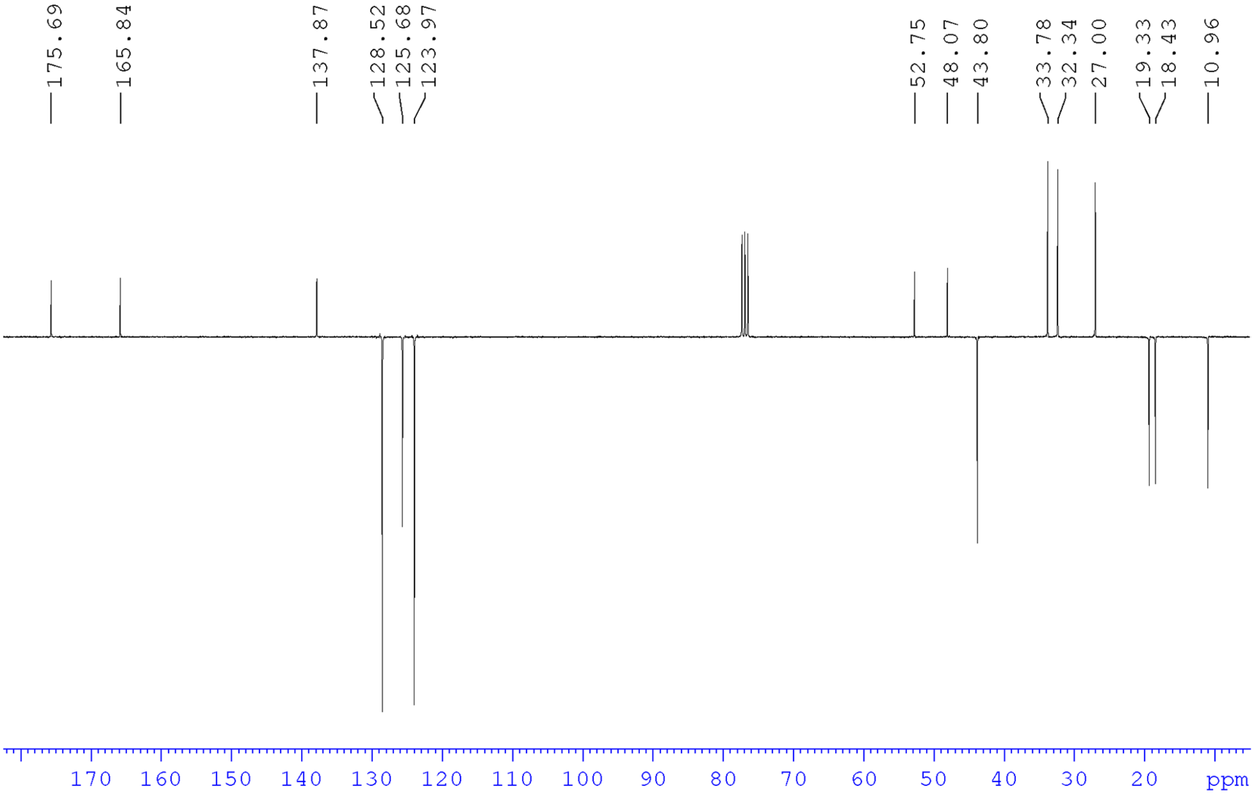

1.6. ¹H NMR and ¹³C NMR spectra of compound 2′b

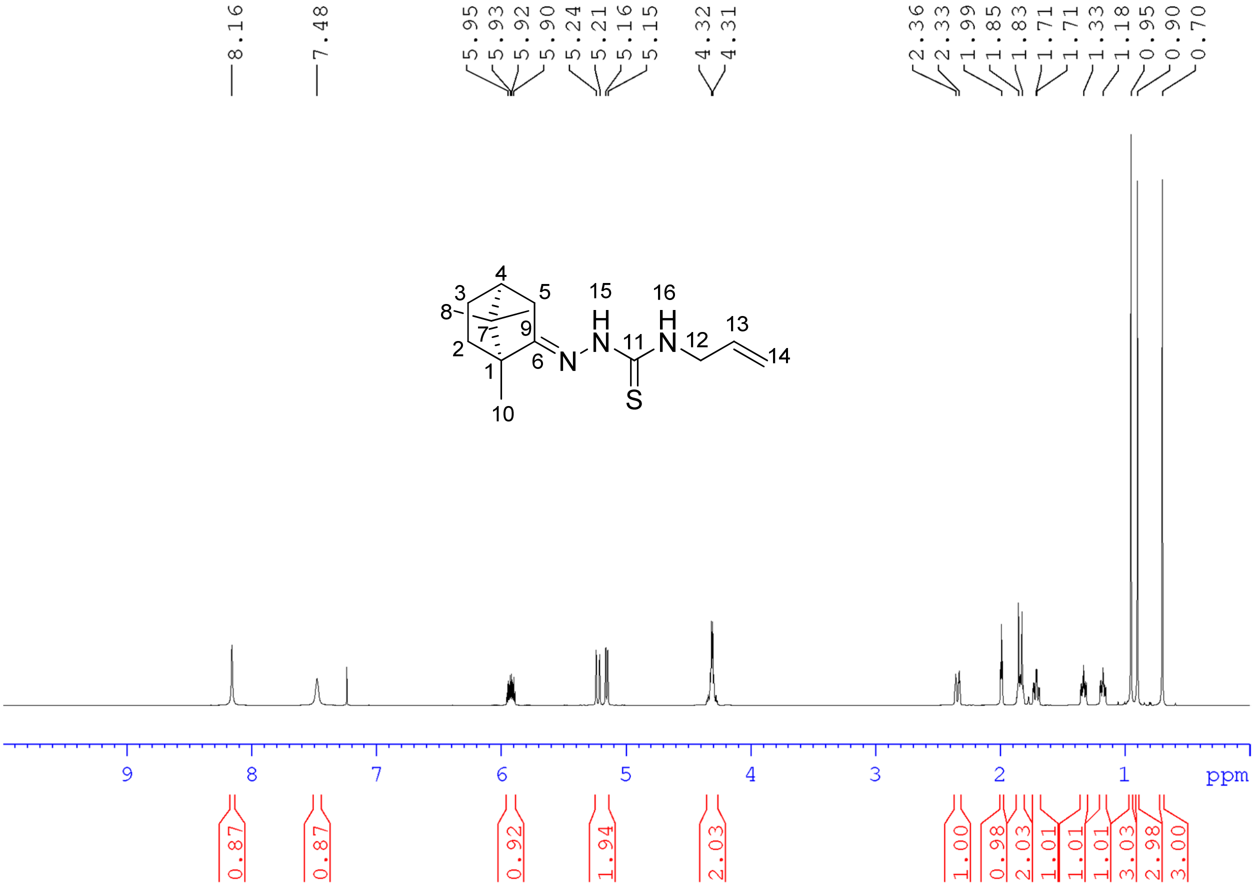

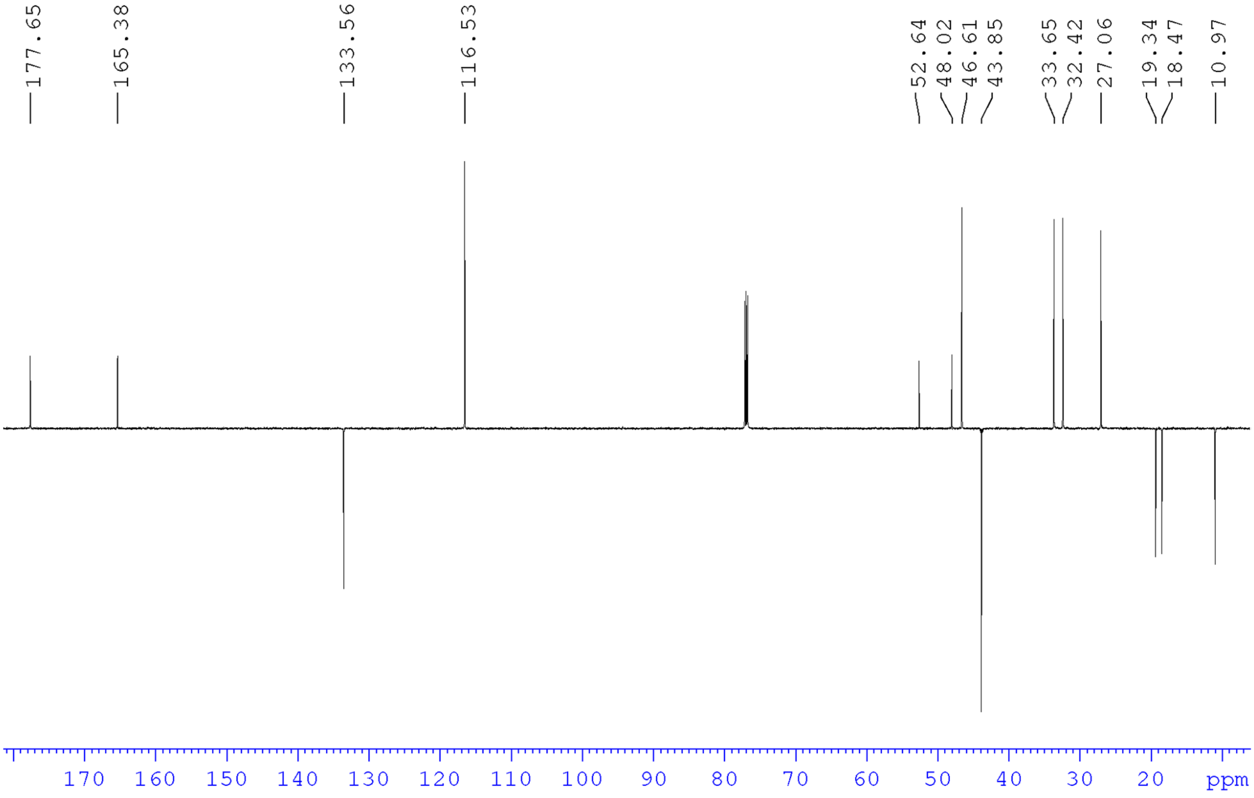

1.7. **¹H NMR and ¹³C NMR spectra of compound 2′c**

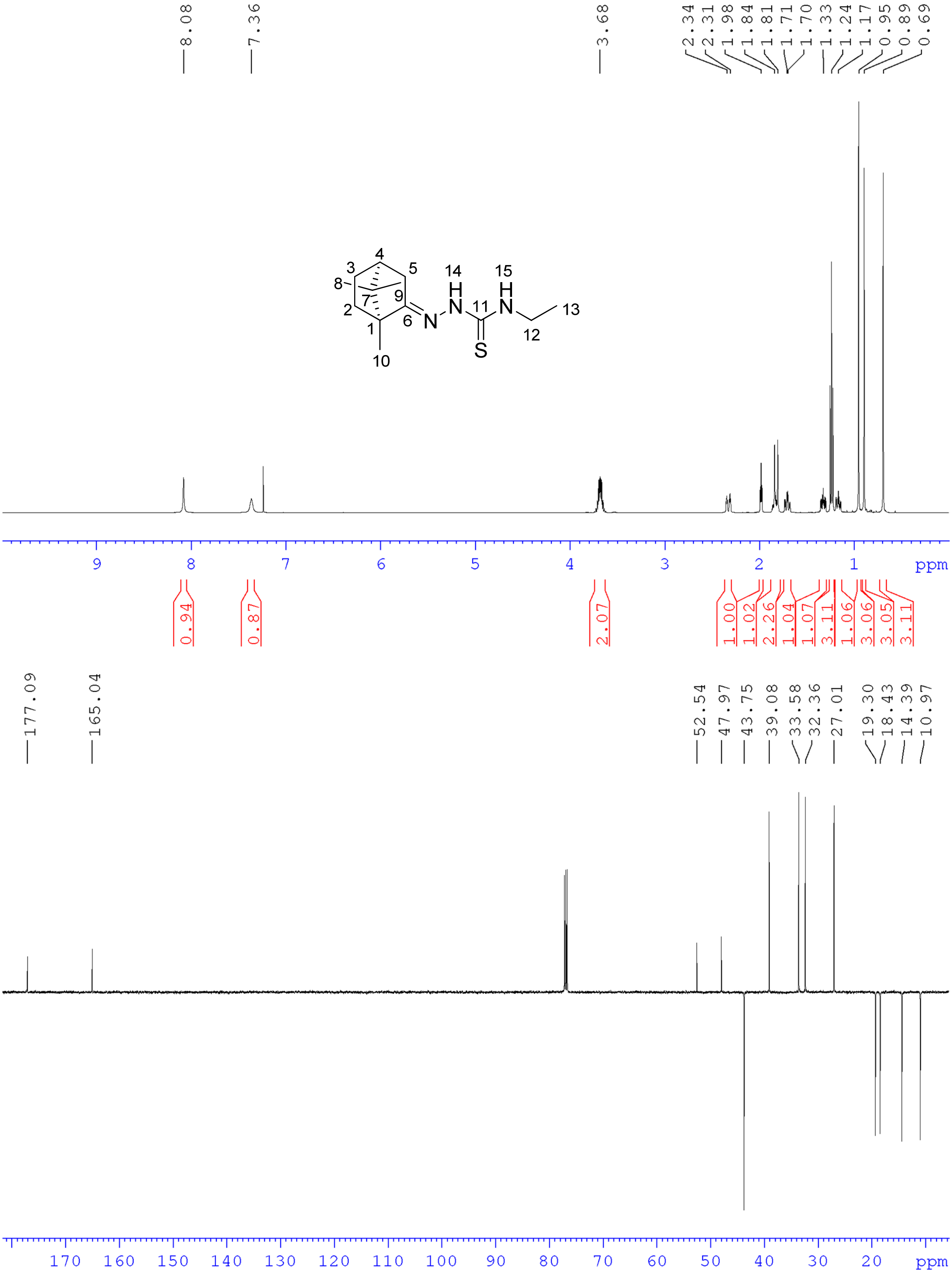

1.8. ^{1}H NMR and ^{13}C NMR spectra of compound 2′d

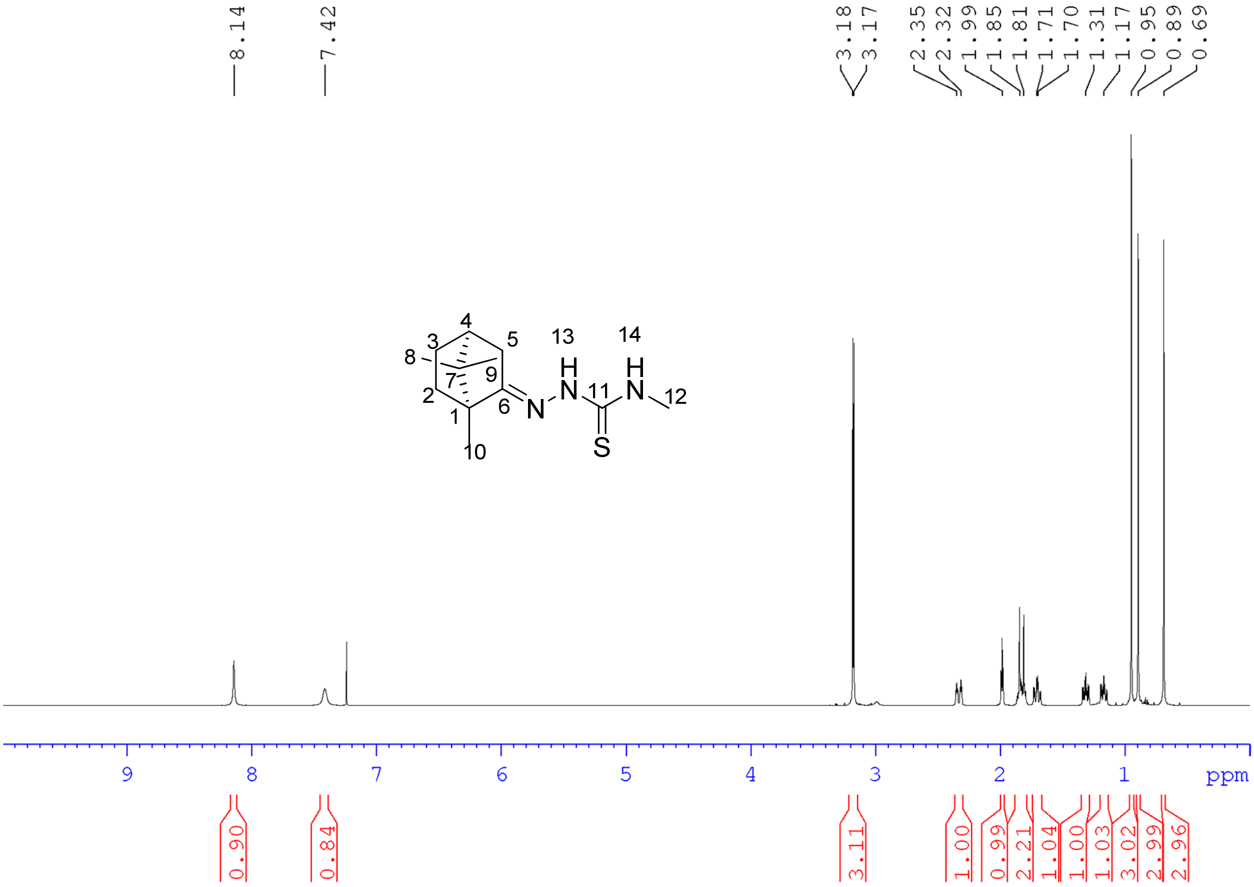

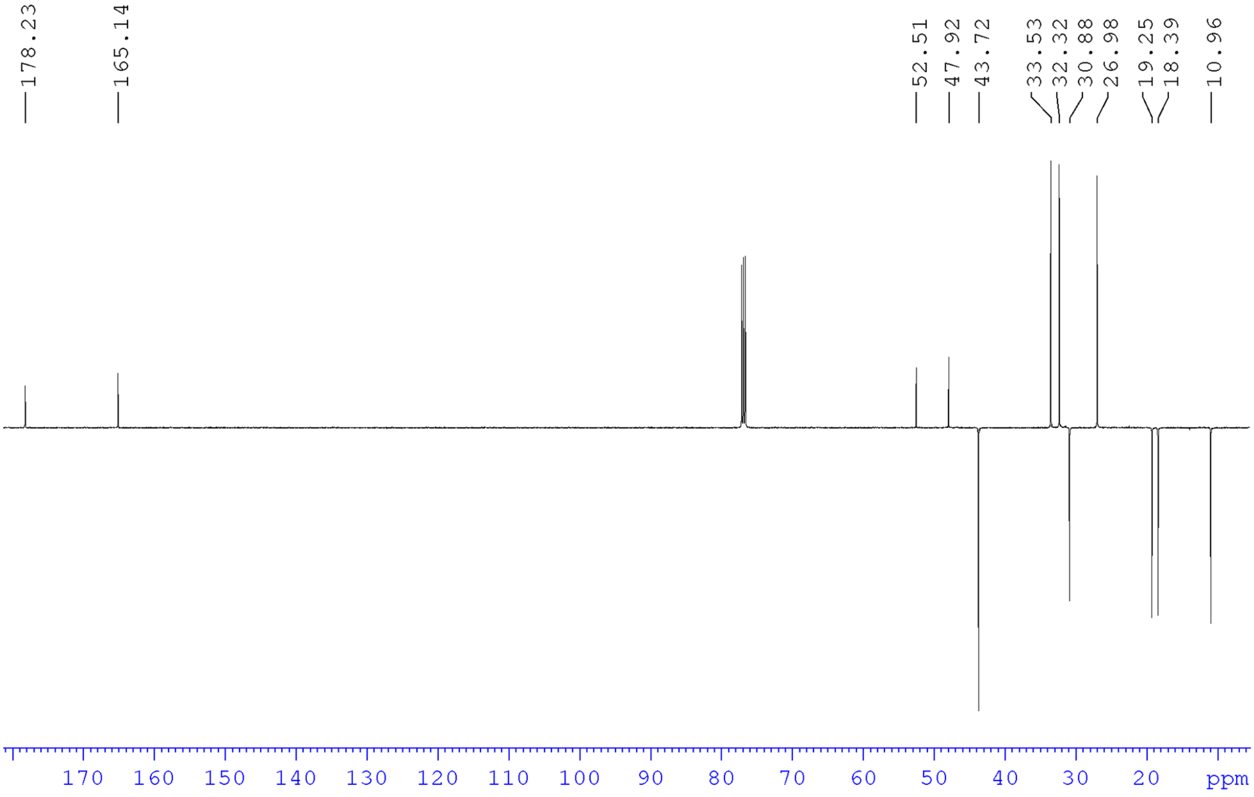

1.9. ^{1}H NMR and ^{13}C NMR spectra of compound 3a

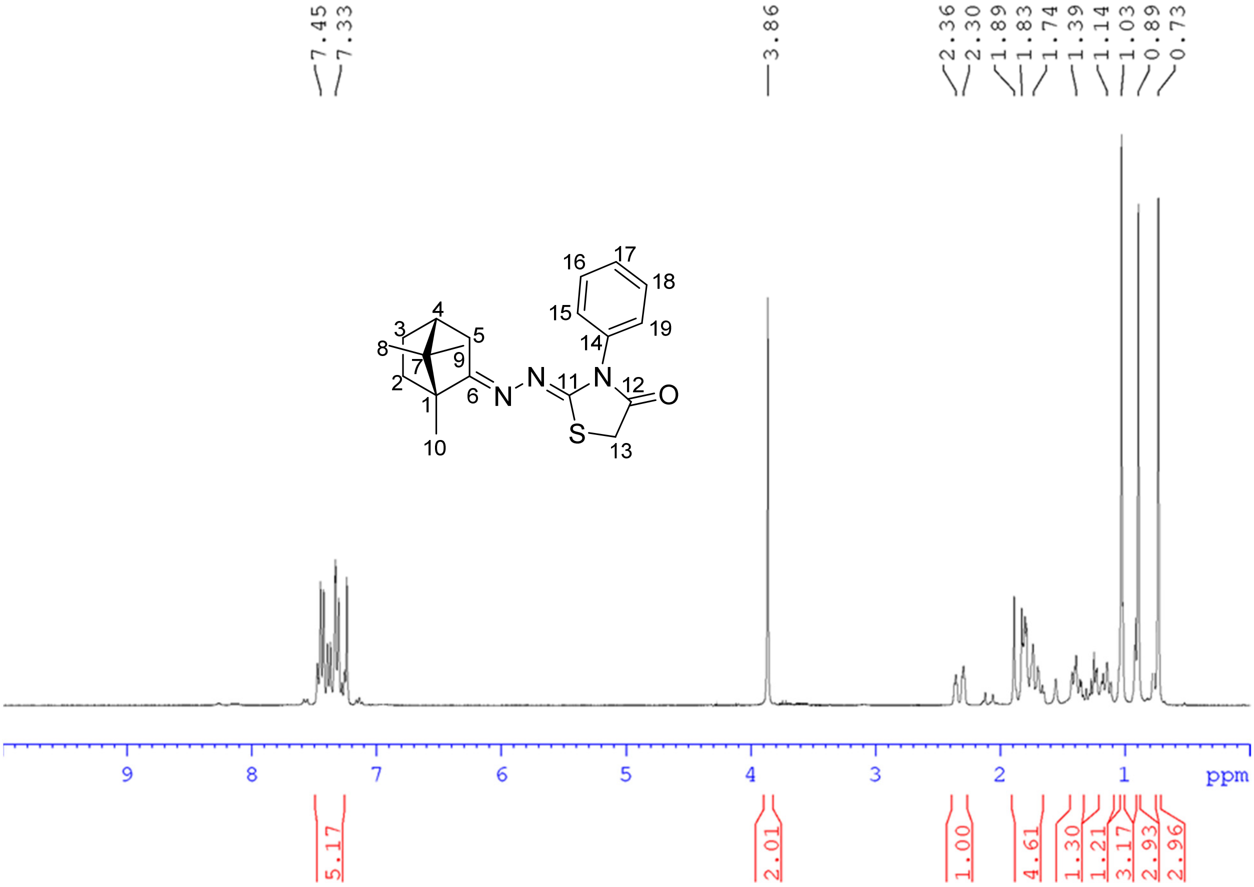

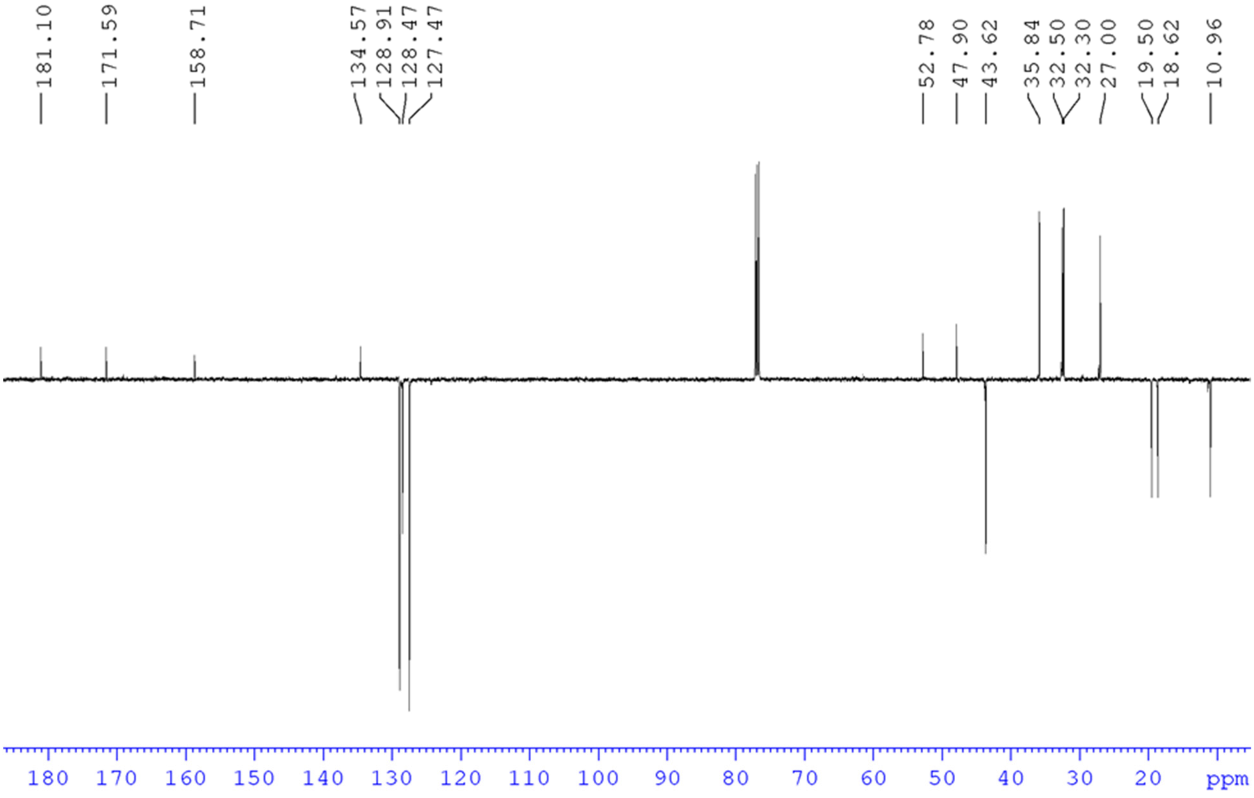

1.10. ^{1}H NMR and ^{13}C NMR spectra of compound 3b

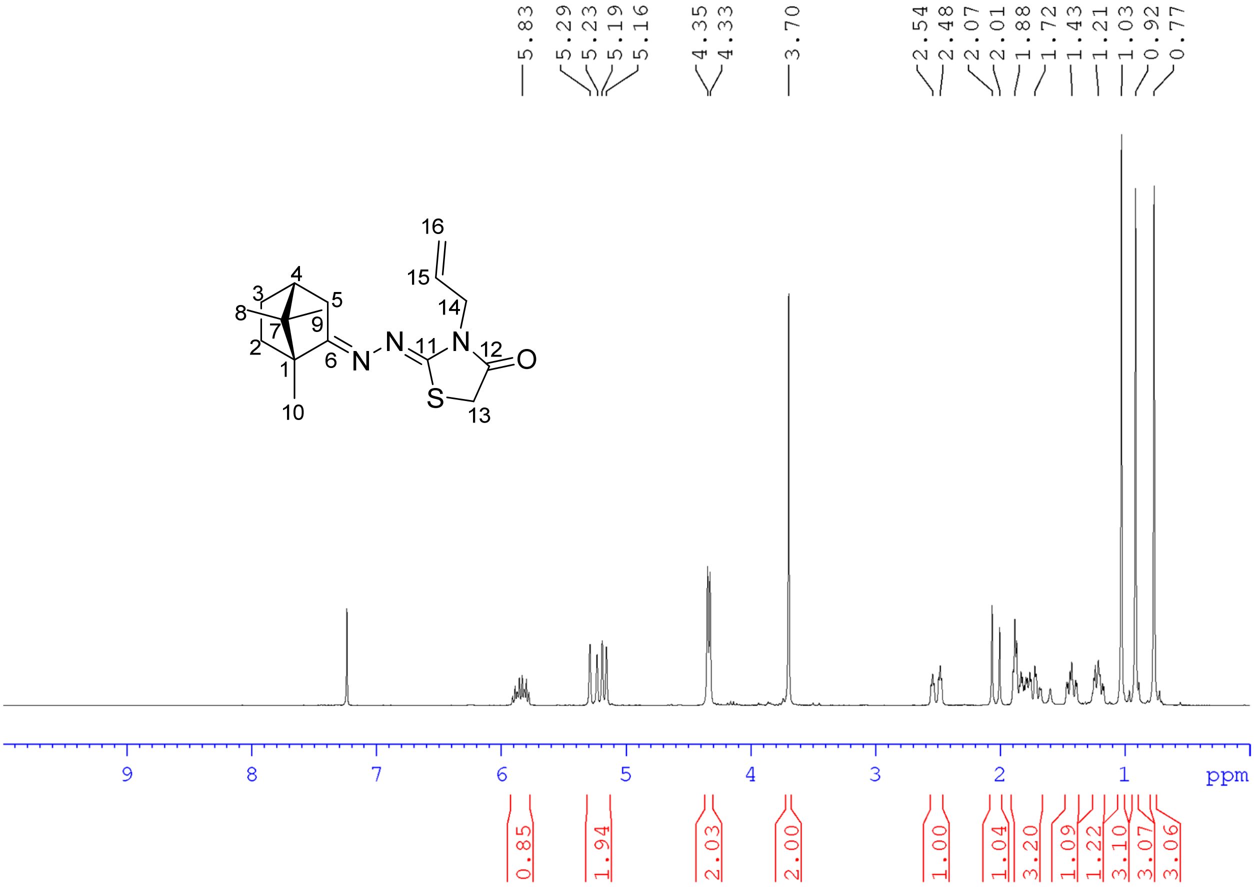

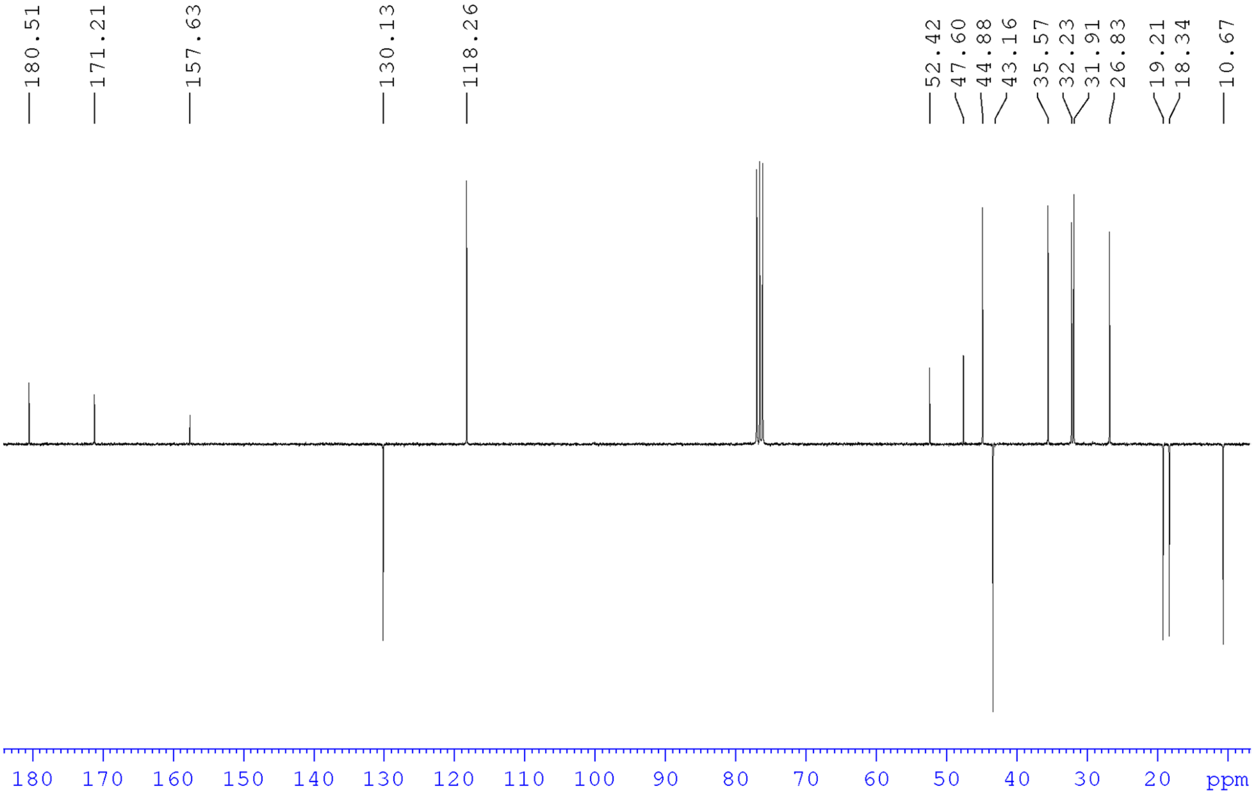

1.11. ^{1}H NMR and ^{13}C NMR spectra of compound 3c

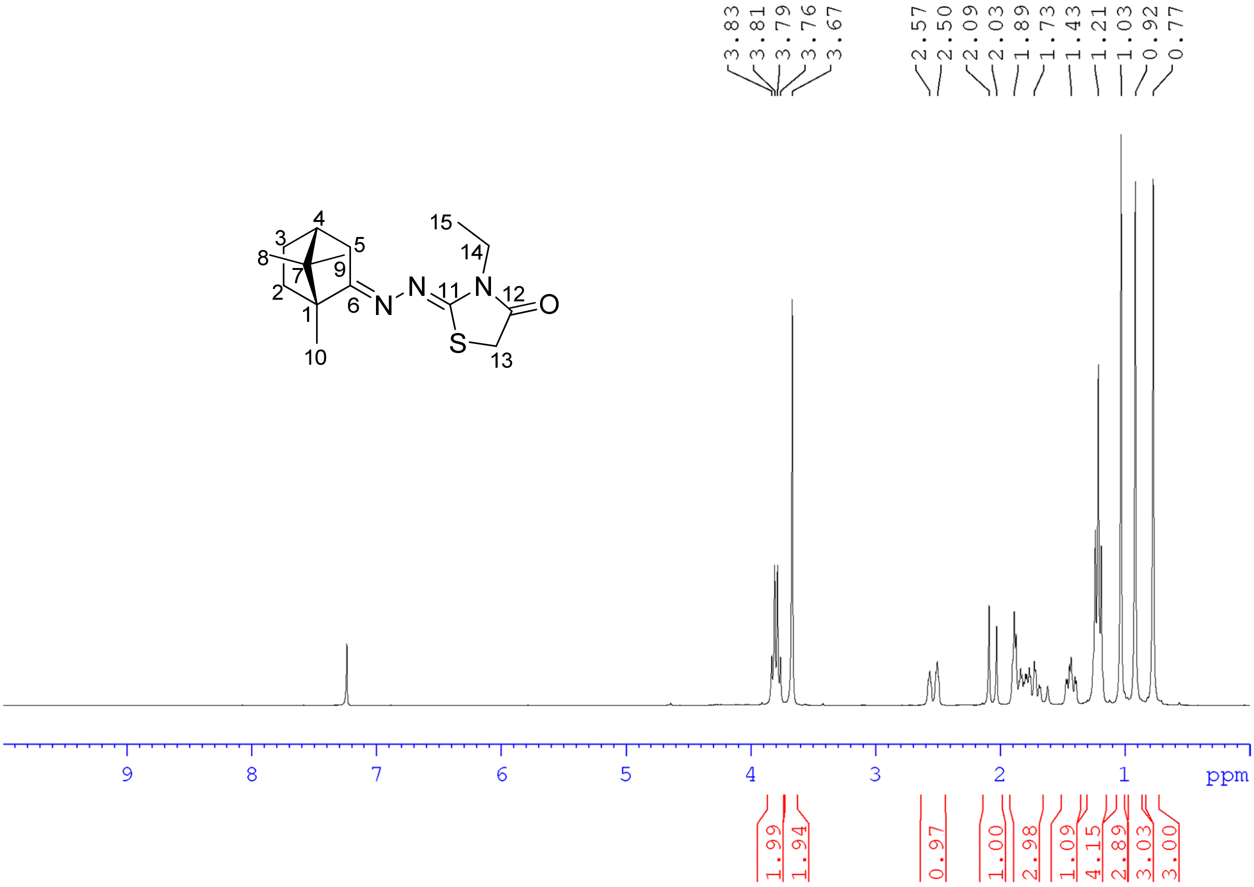

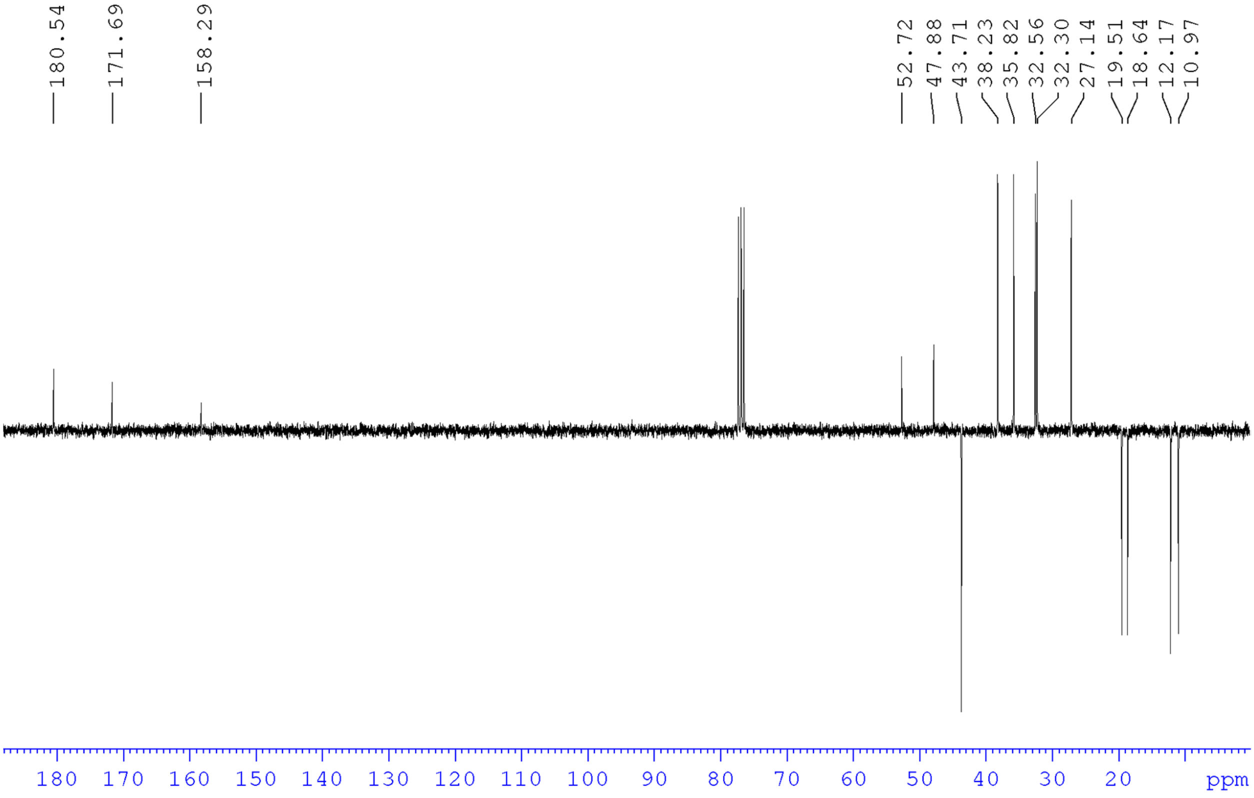

1.12. [1]**H NMR and** [13]**C NMR spectra of compound 3d**

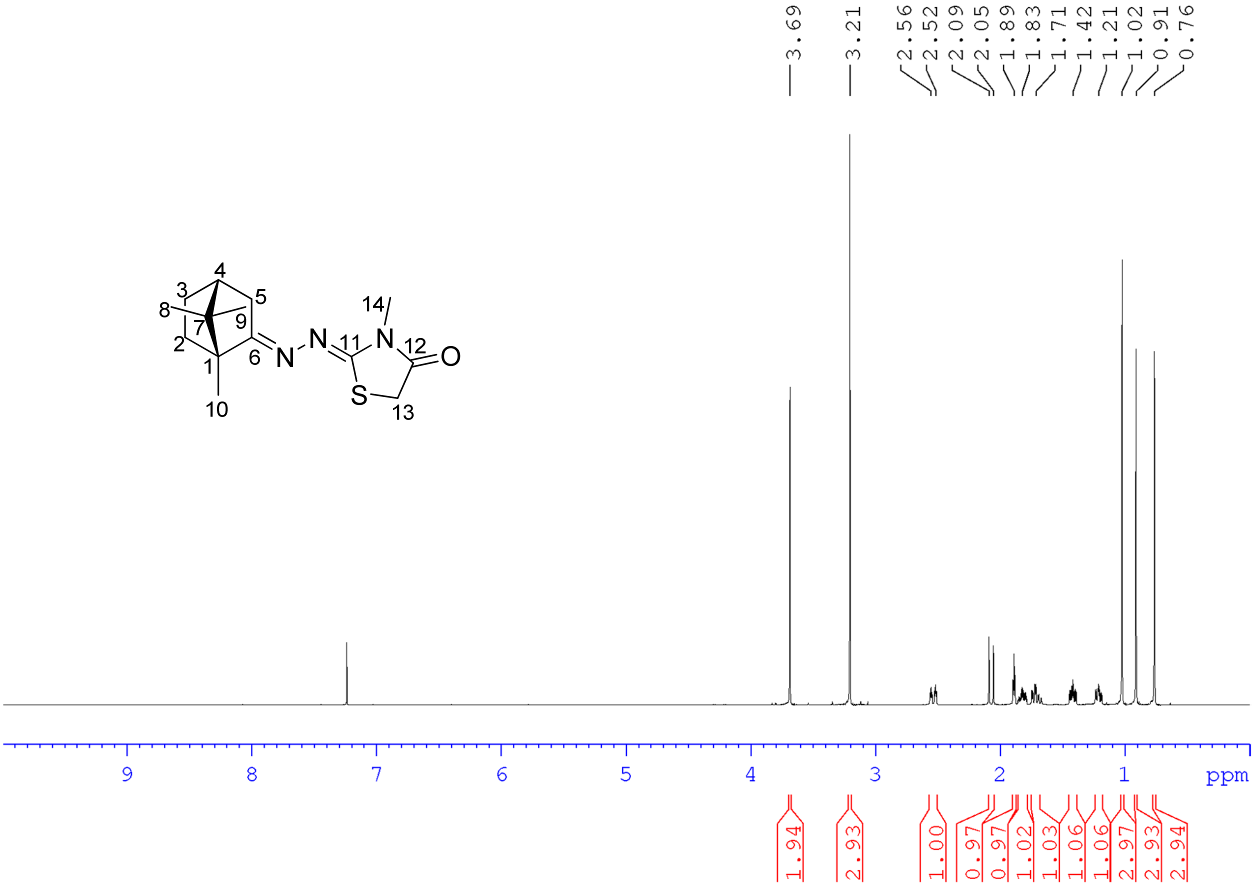

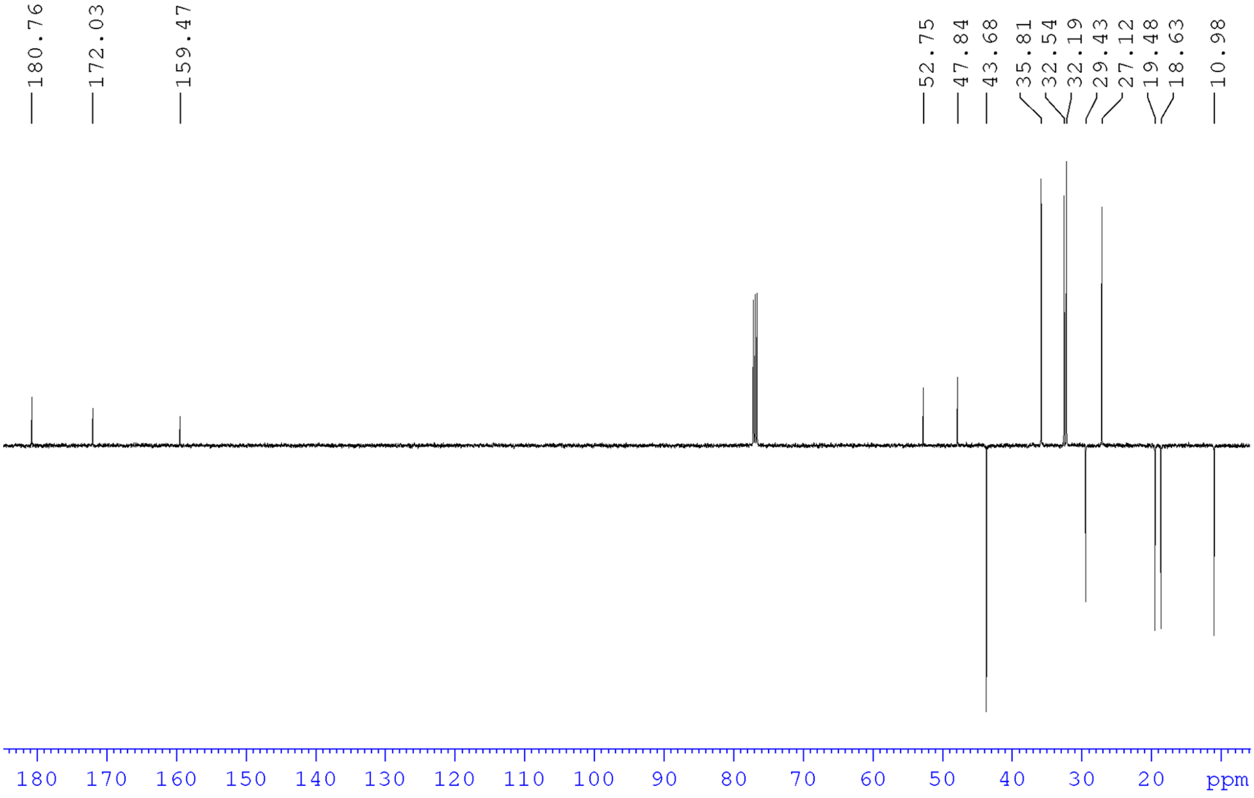

1.13. **^{1}H NMR and ^{13}C NMR spectra of compound 3′a**

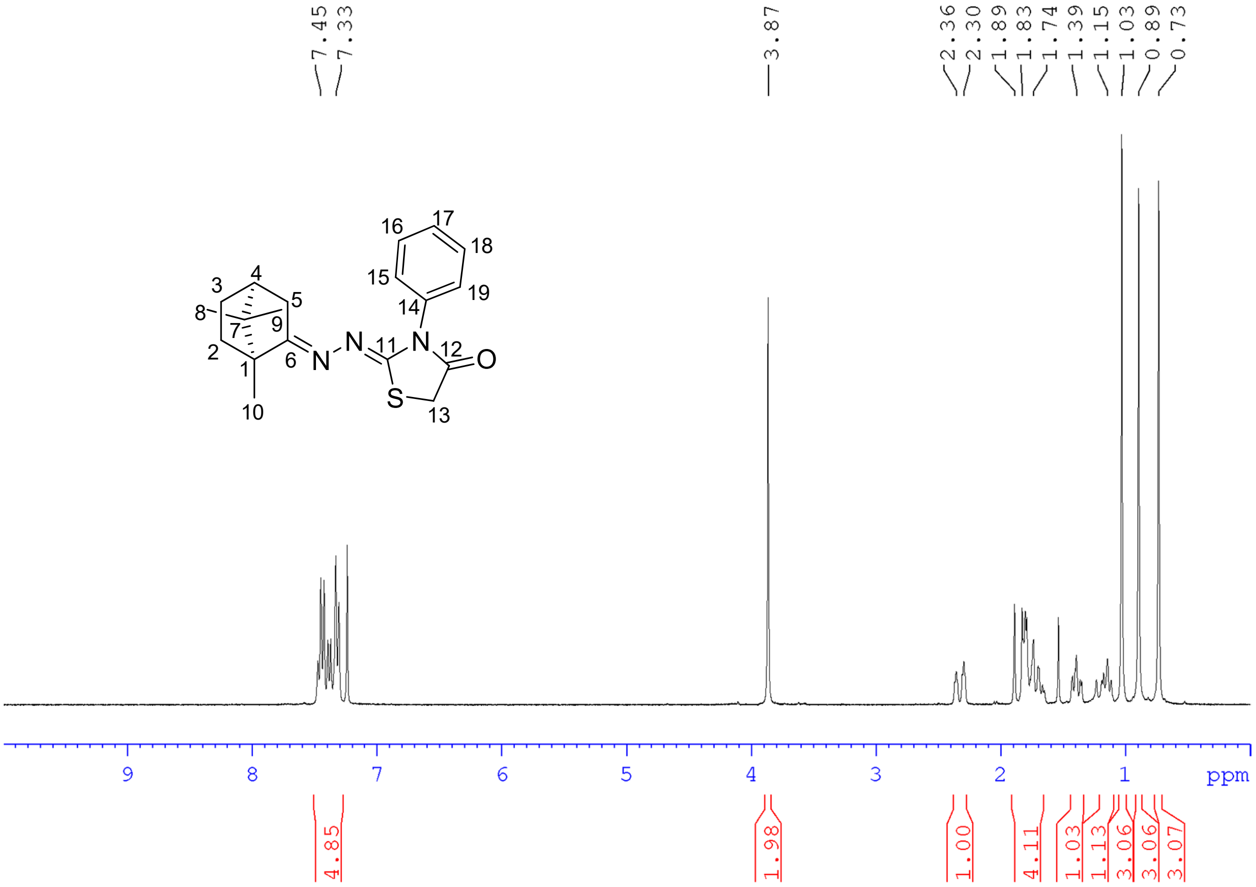

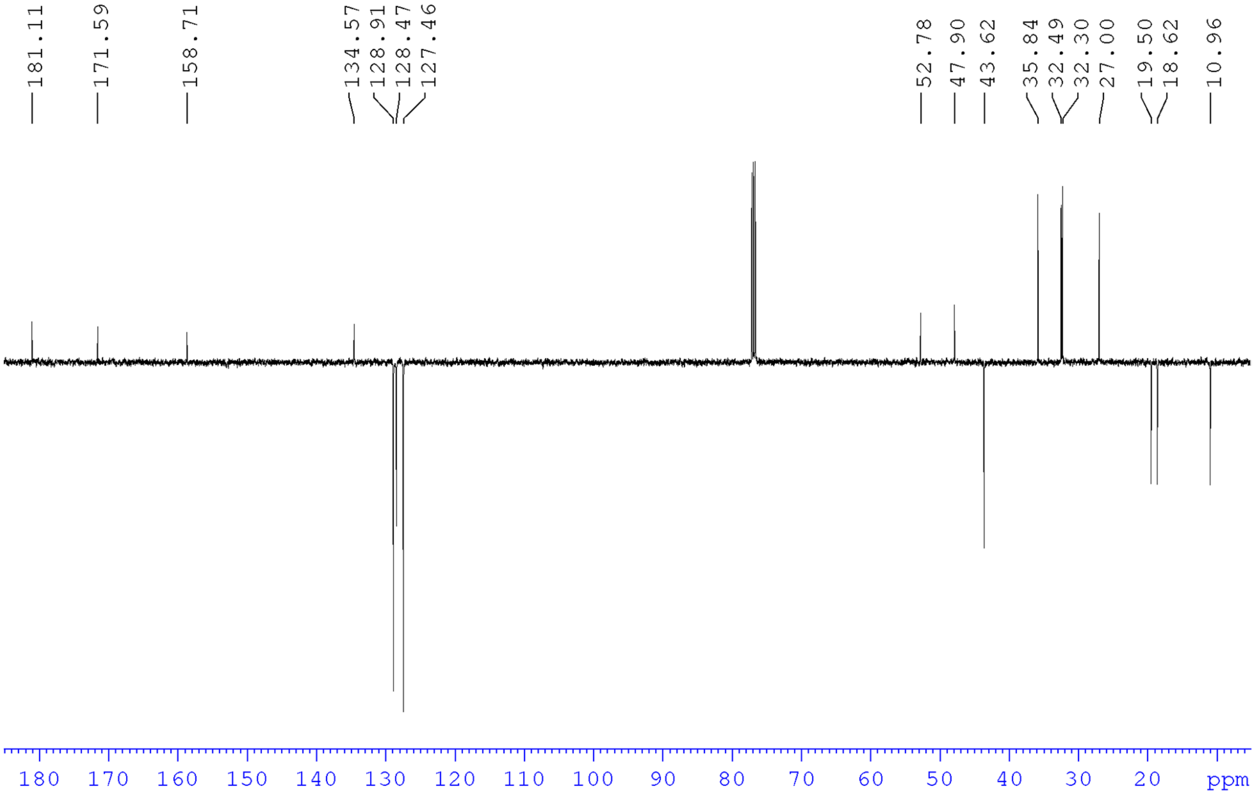

1.14. ^{1}H NMR and ^{13}C NMR spectra of compound 3′b

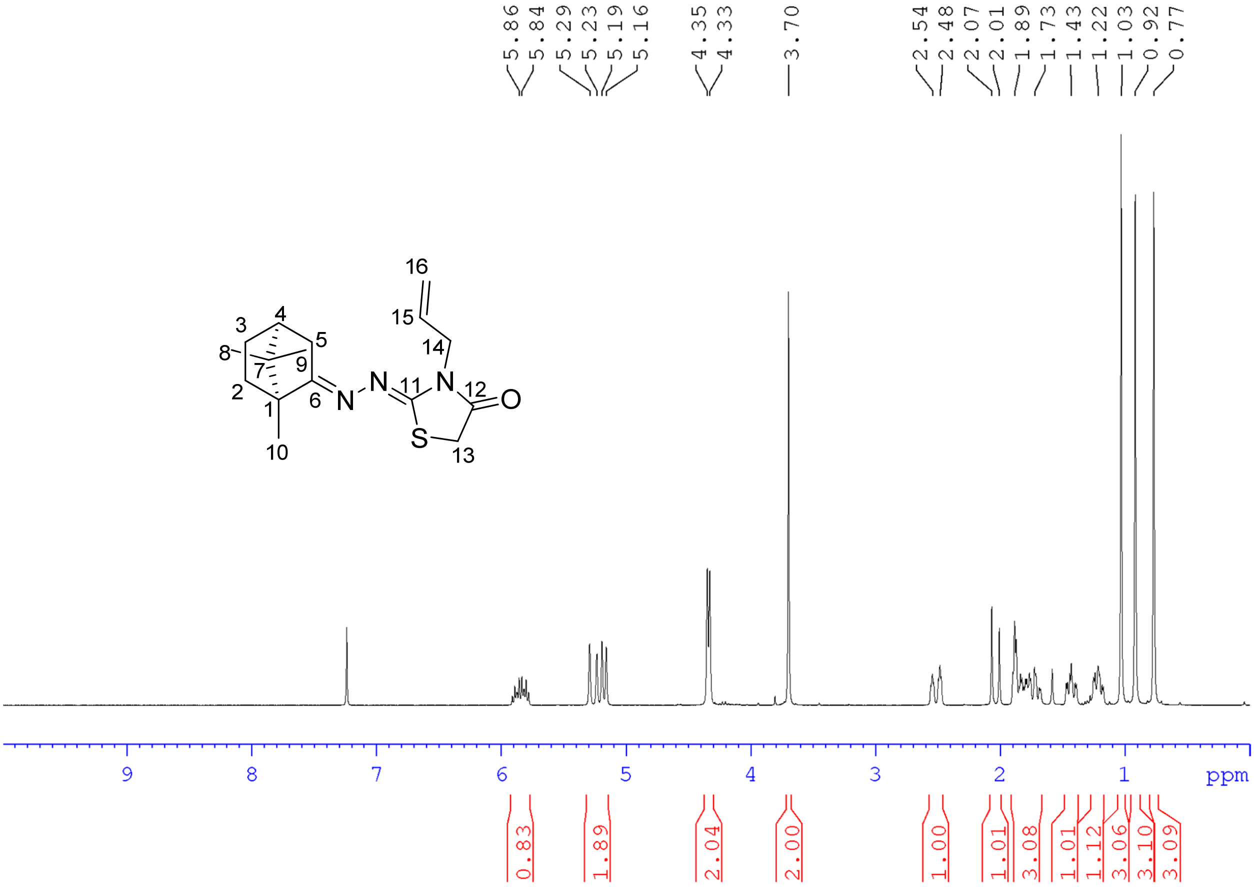

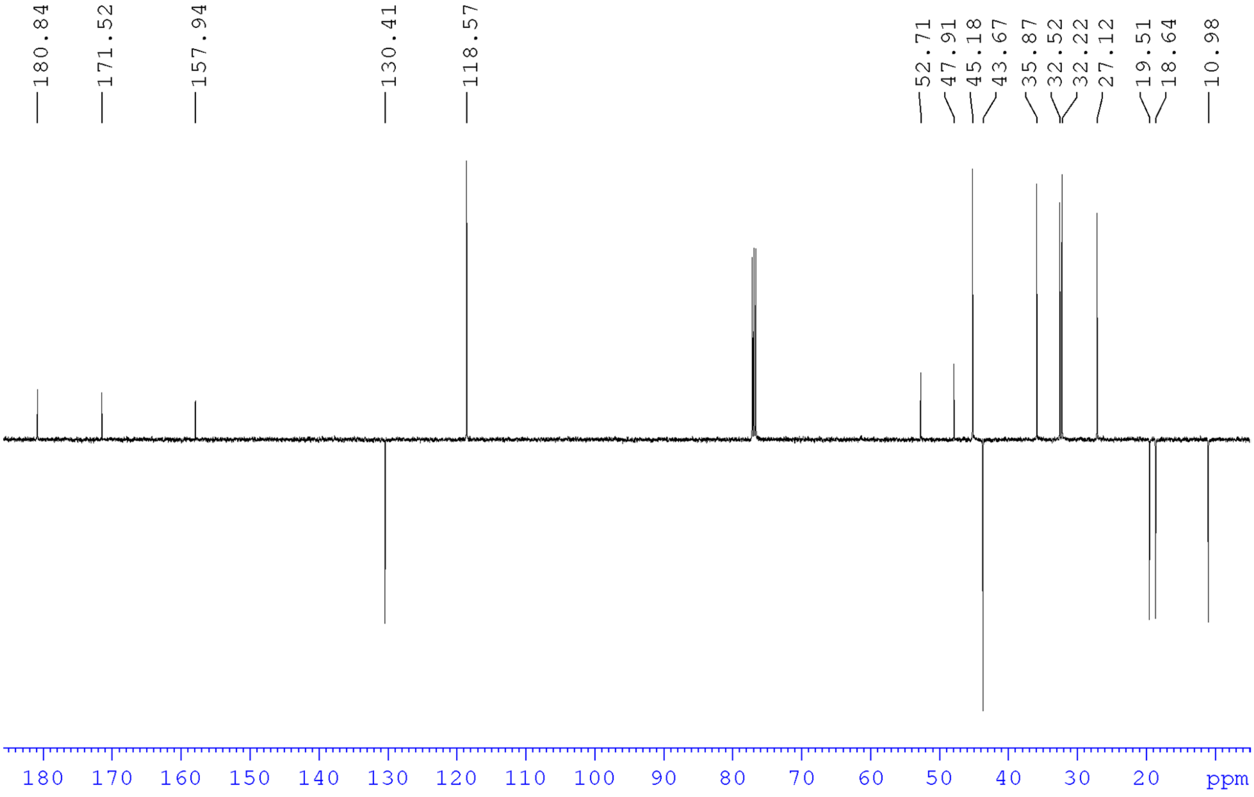

1.15. [1]**H NMR and** [13]**C NMR spectra of compound 3′c**

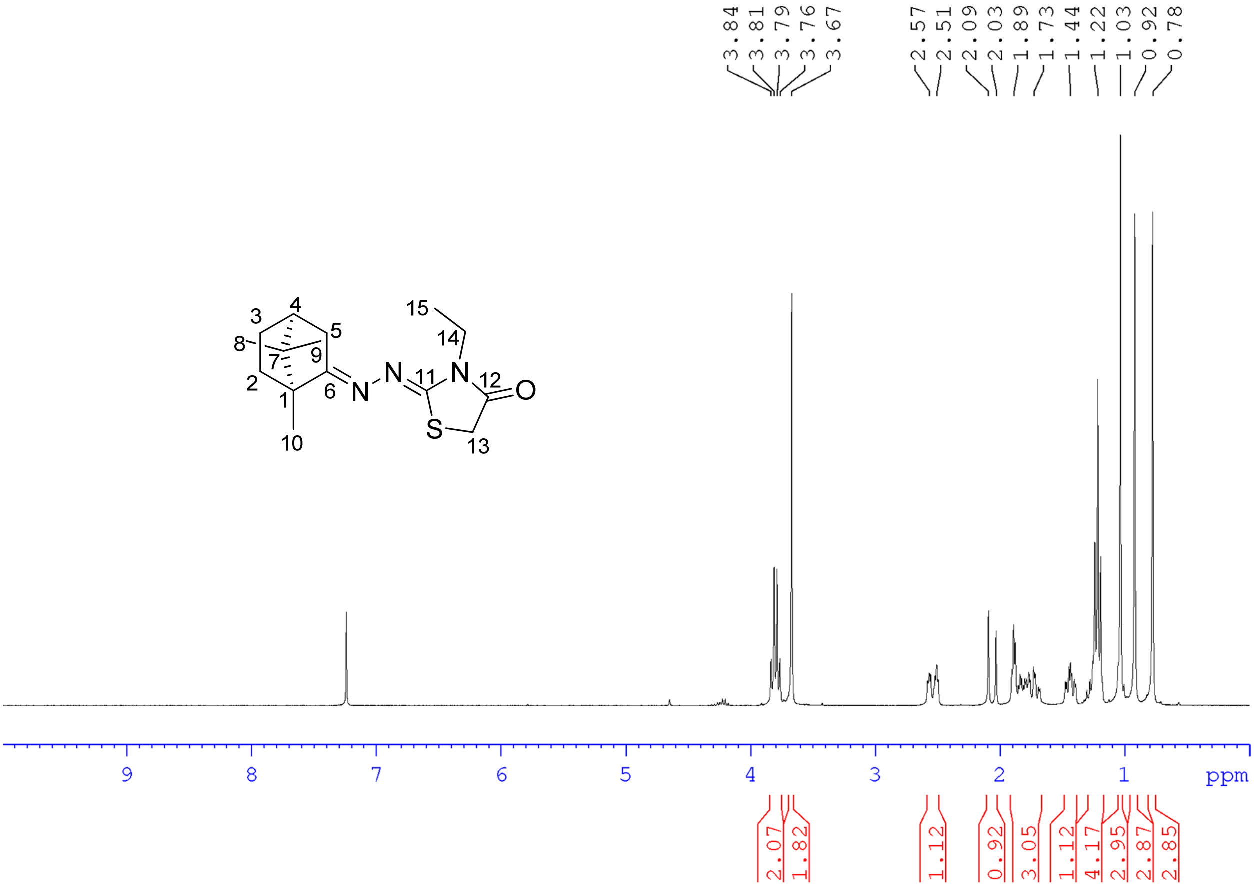

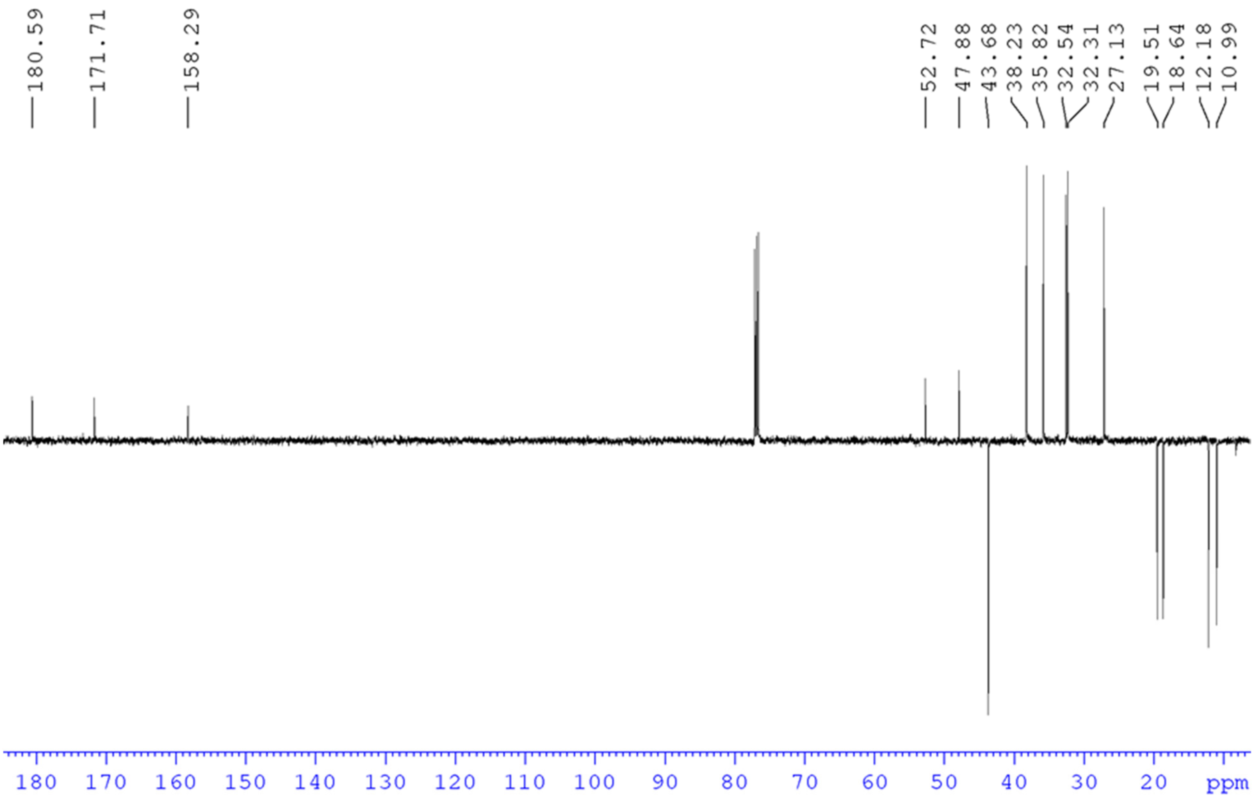

1.16. ^{1}H NMR and ^{13}C NMR spectra of compound 3′d

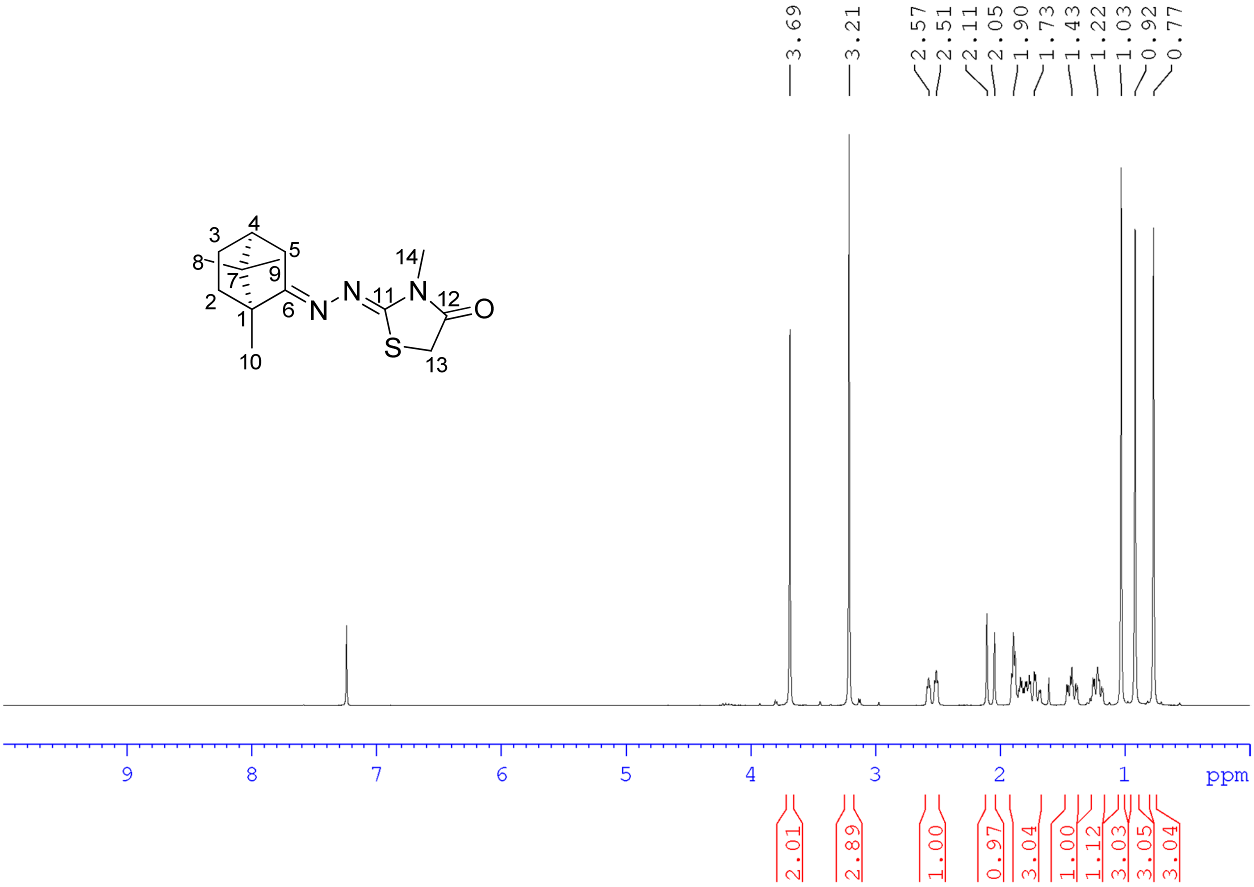

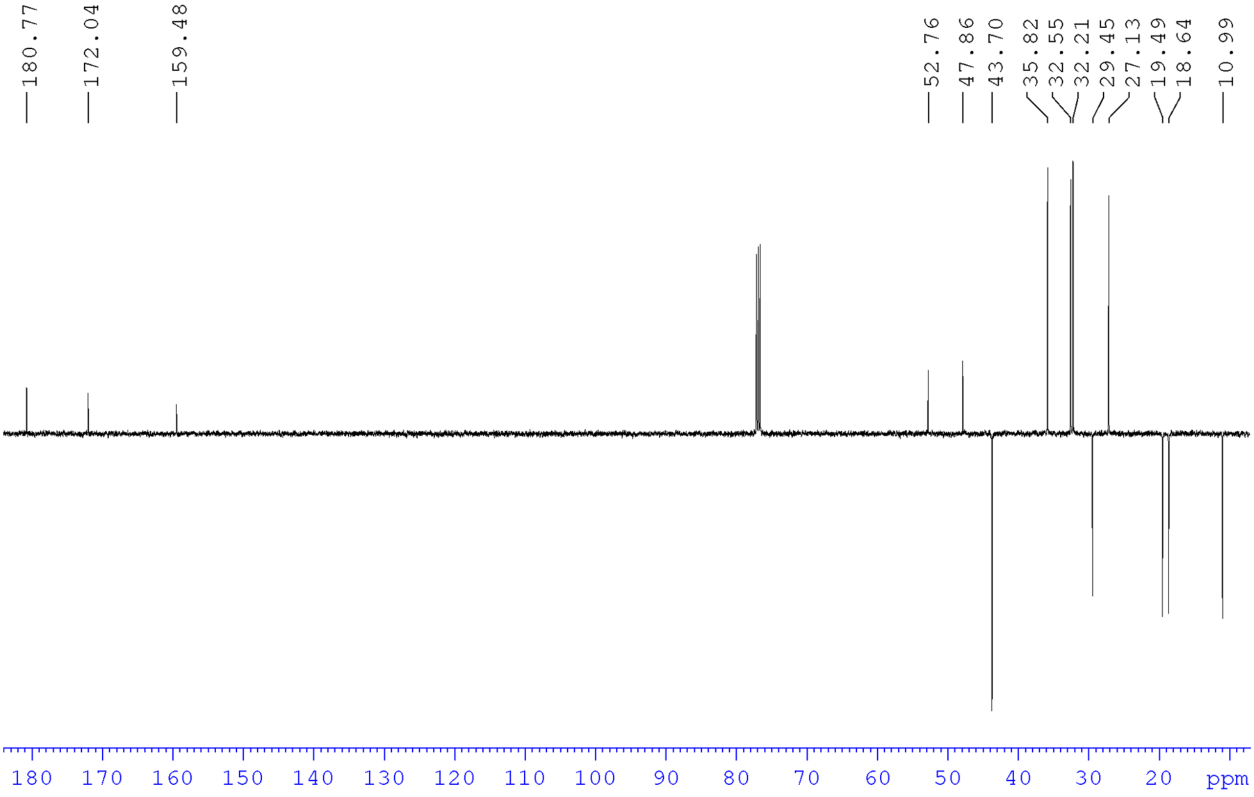

1.17. ^{1}H NMR and ^{13}C NMR spectra of compound 4a

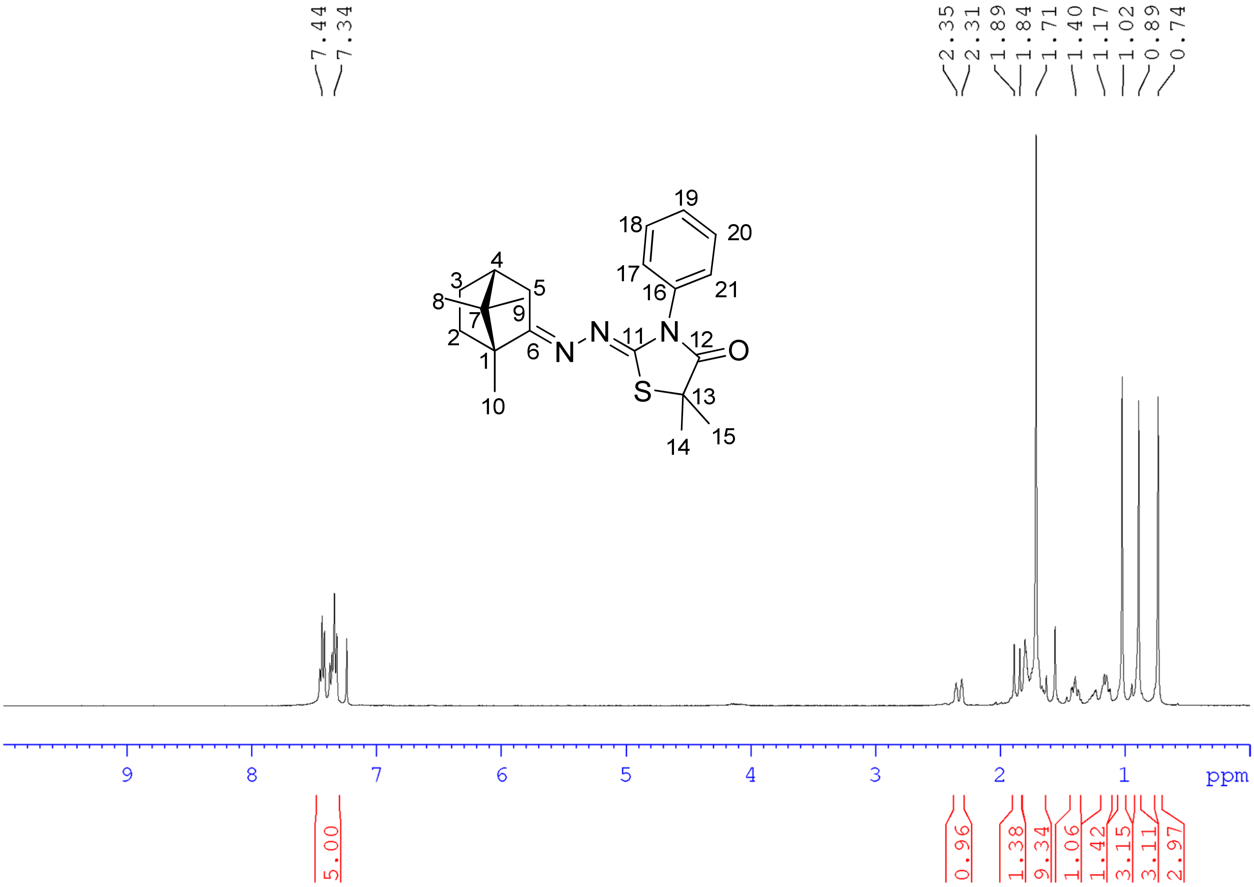

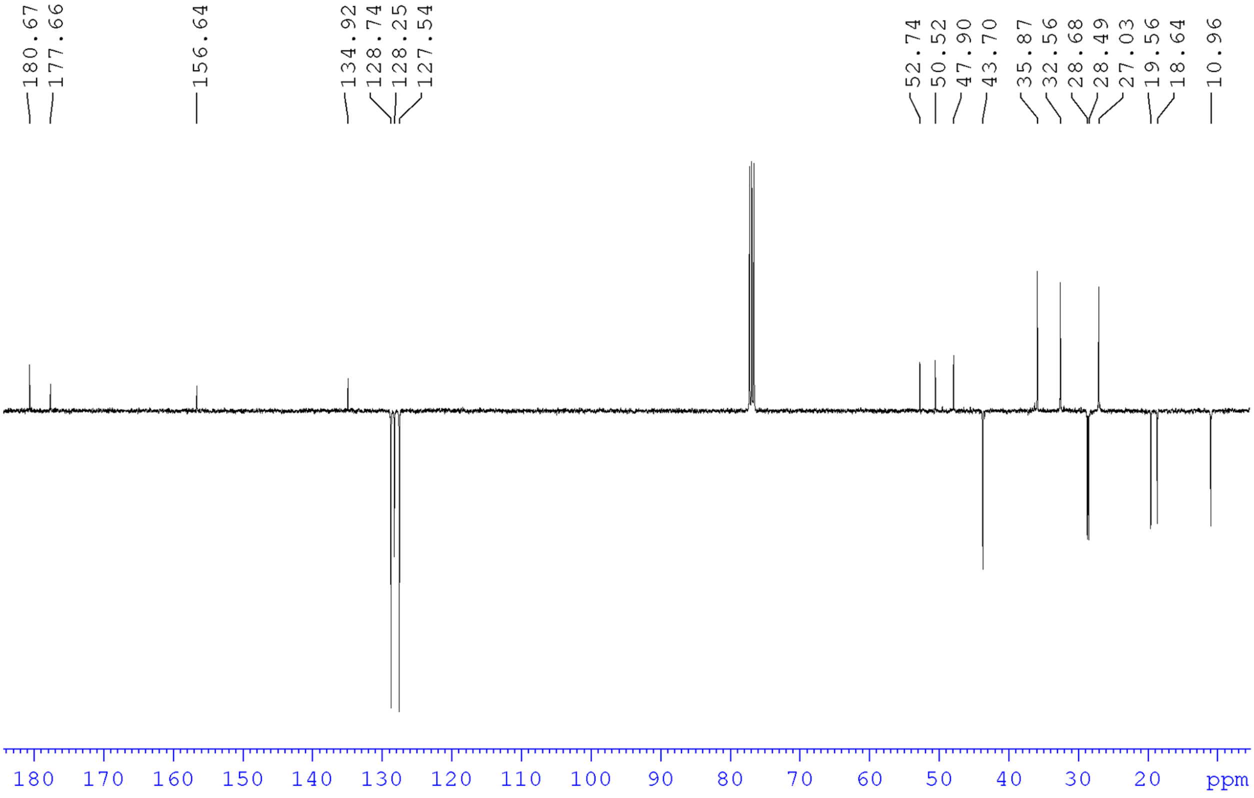

1.18. ¹H NMR and ¹³C NMR spectra of compound 4b

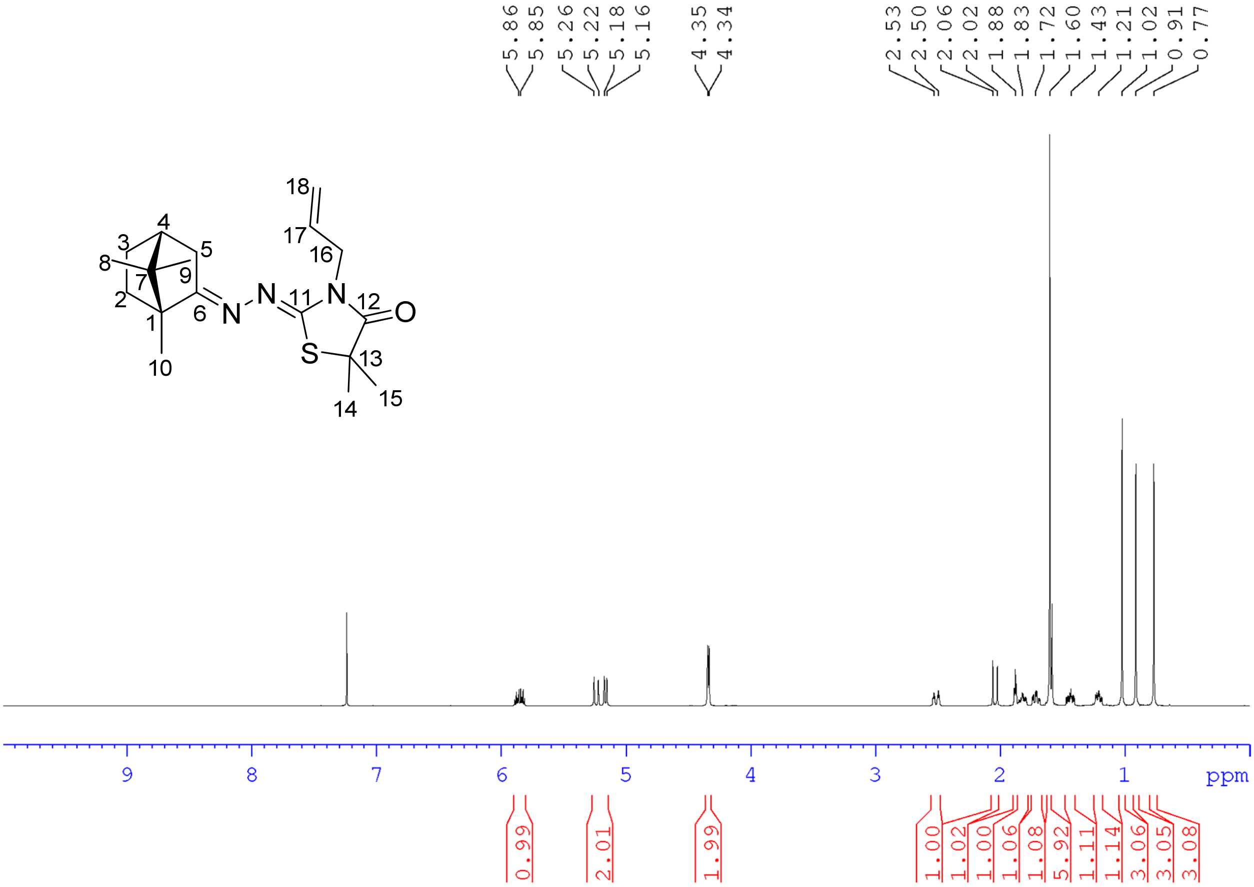

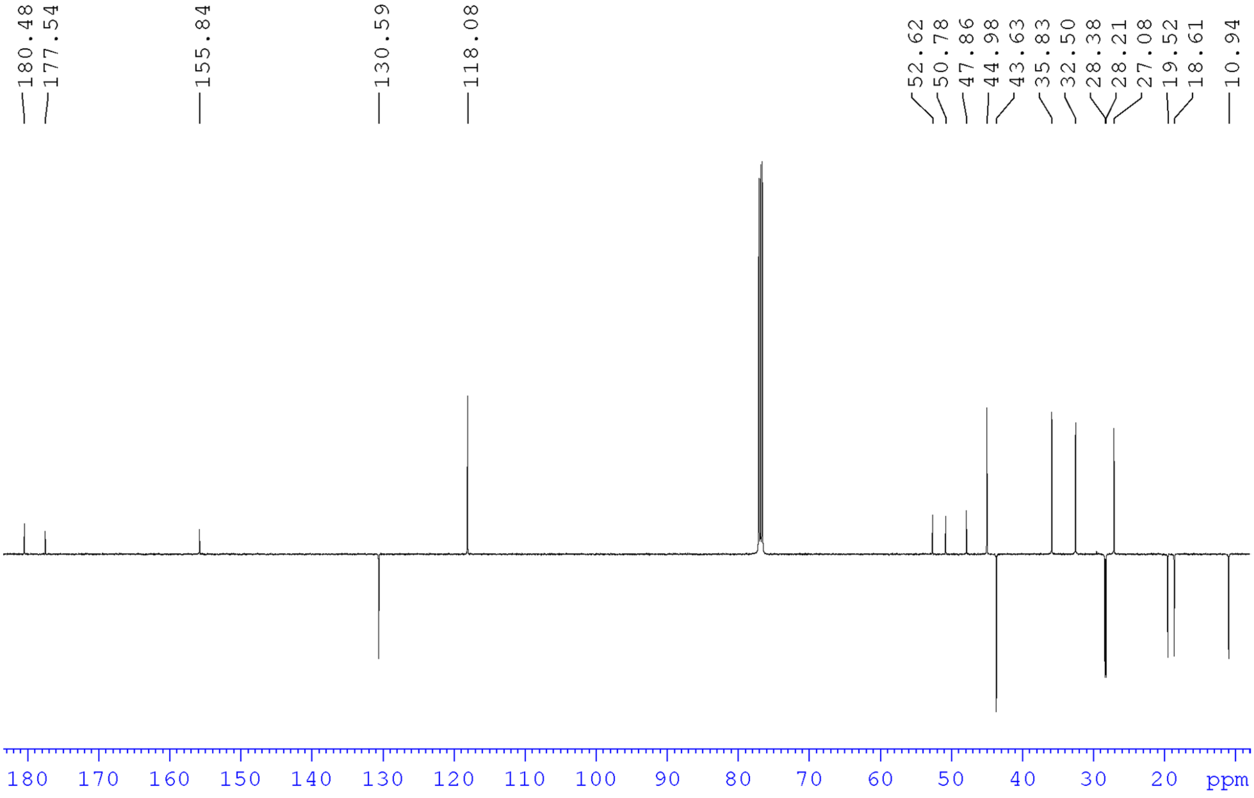

1.19. [1]**H NMR and** [13]**C NMR spectra of compound 4c**

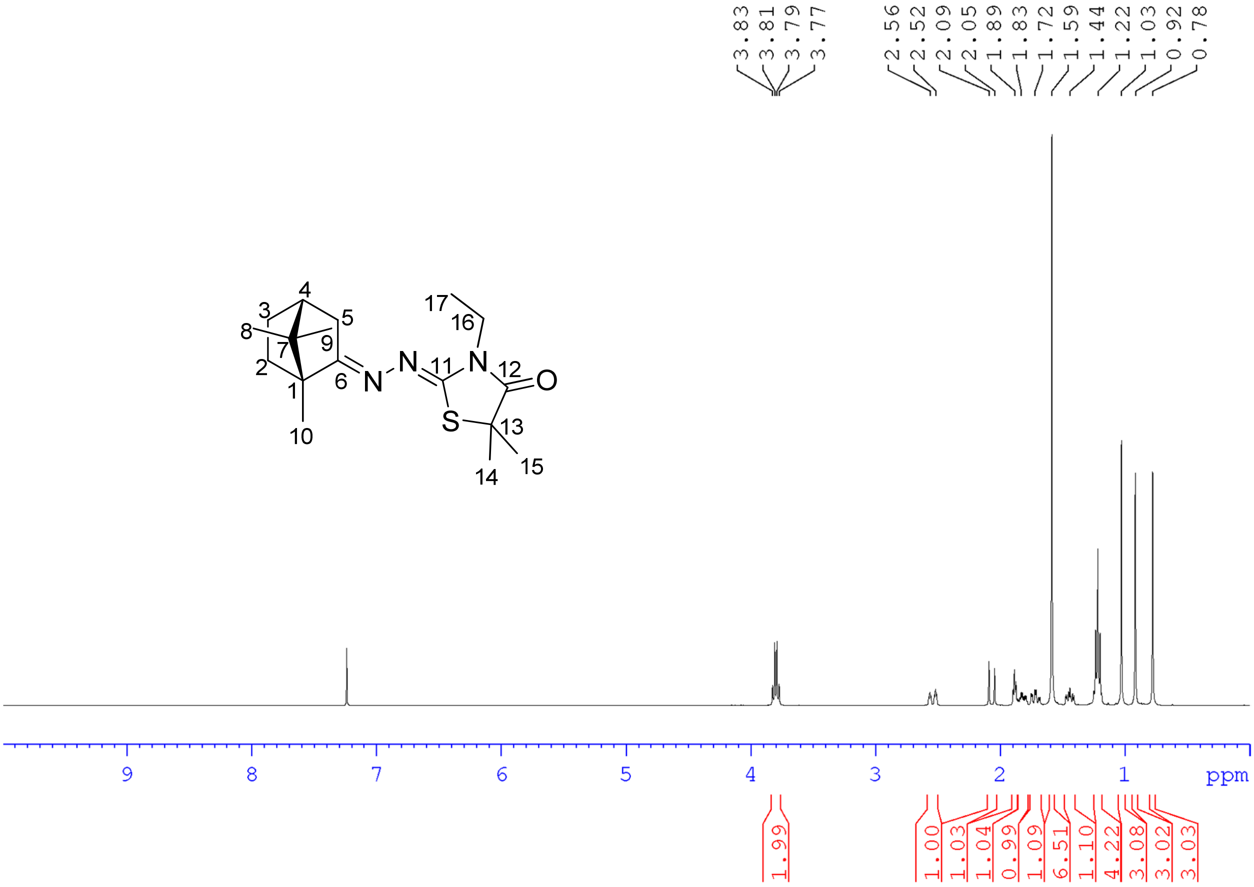

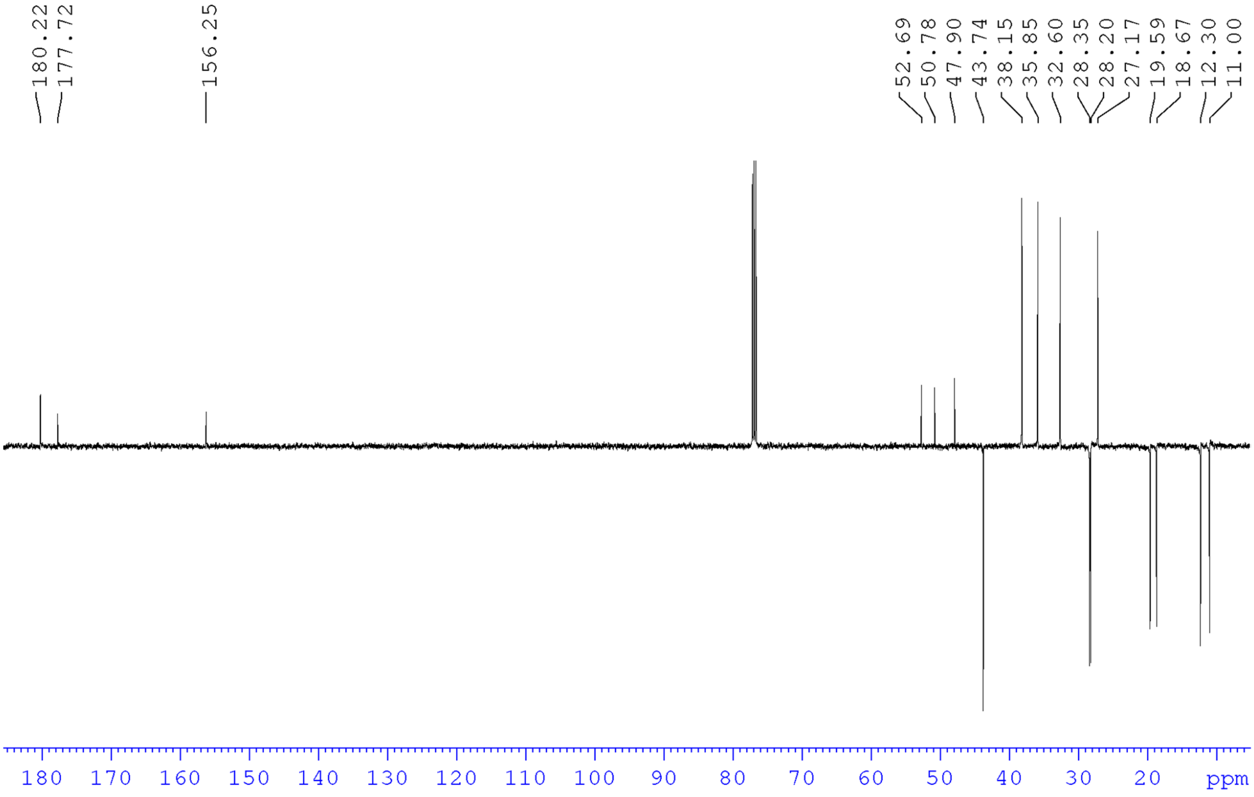

1.20. [1]**H NMR and** [13]**C NMR spectra of compound 4d**

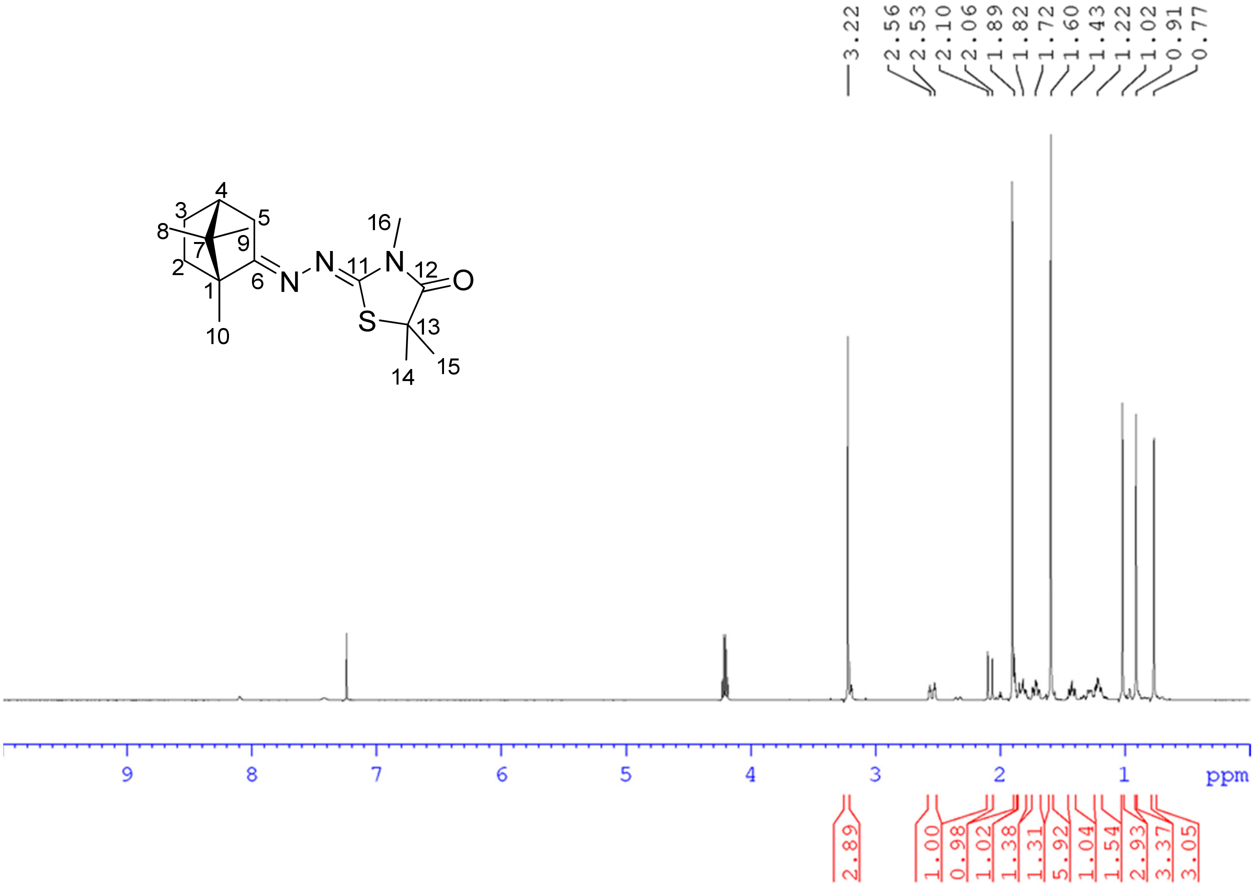

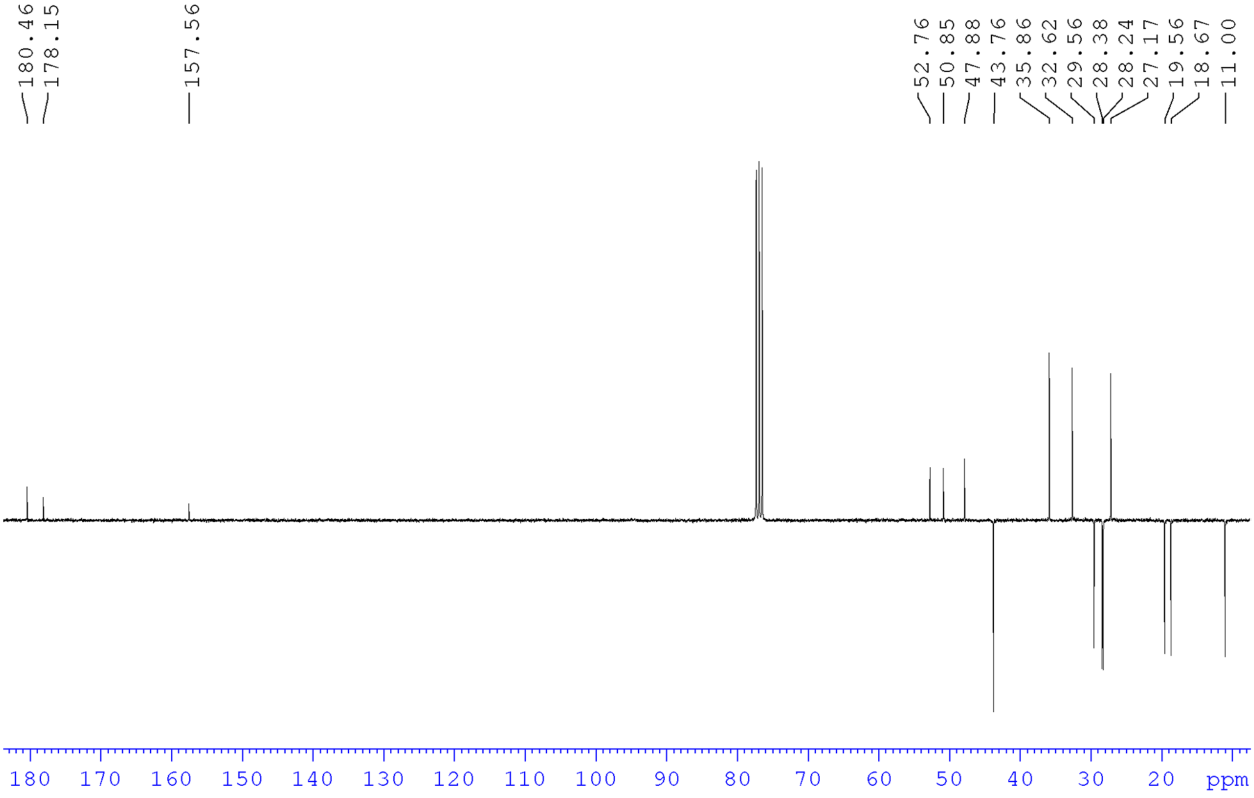

1.21. [1]**H NMR and** [13]**C NMR spectra of compound 5a**

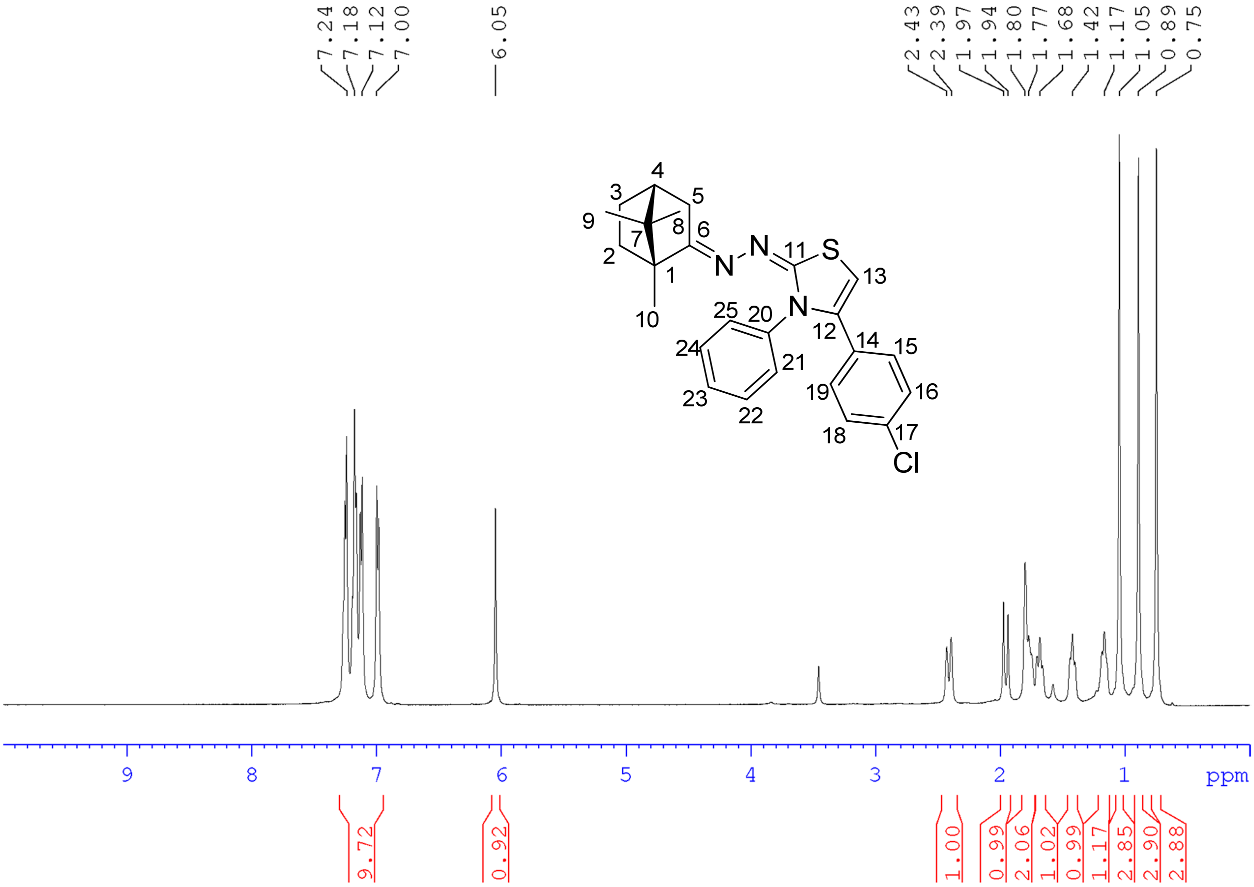

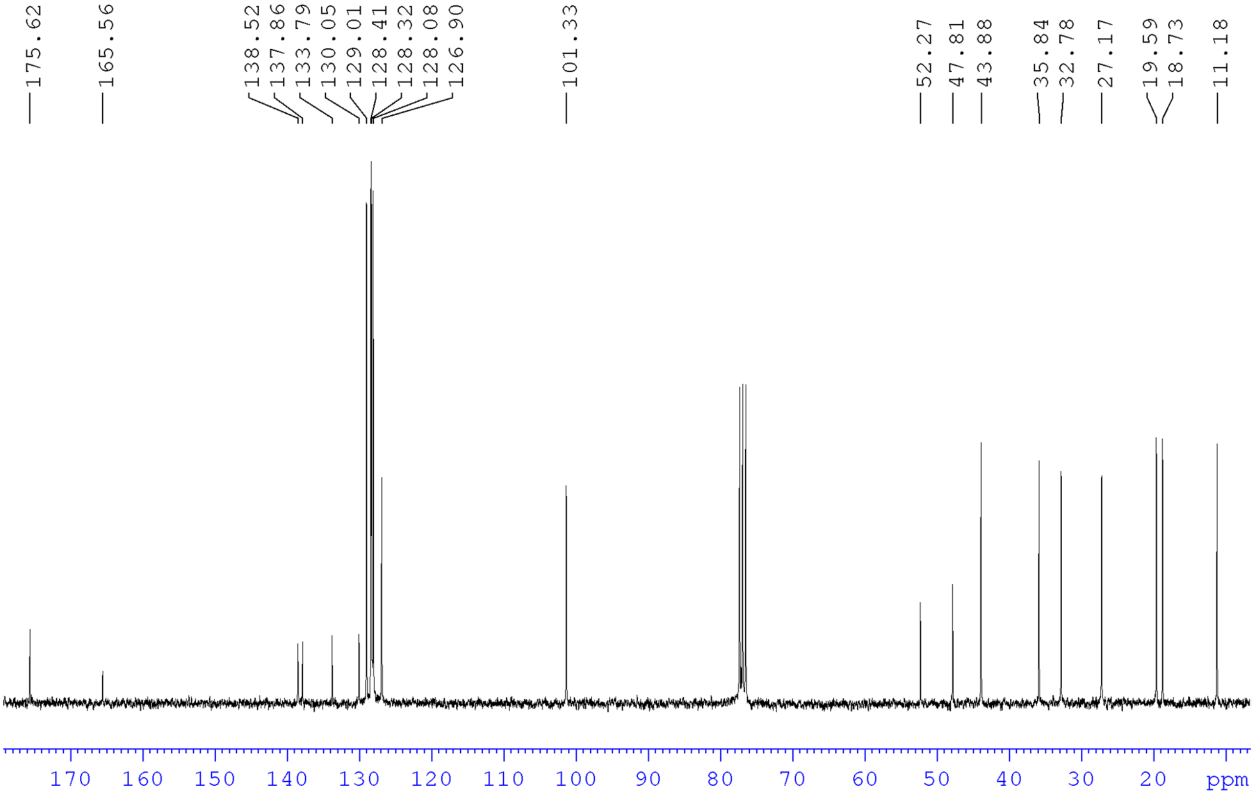

1.22. ^{1}H NMR and ^{13}C NMR spectra of compound 5b

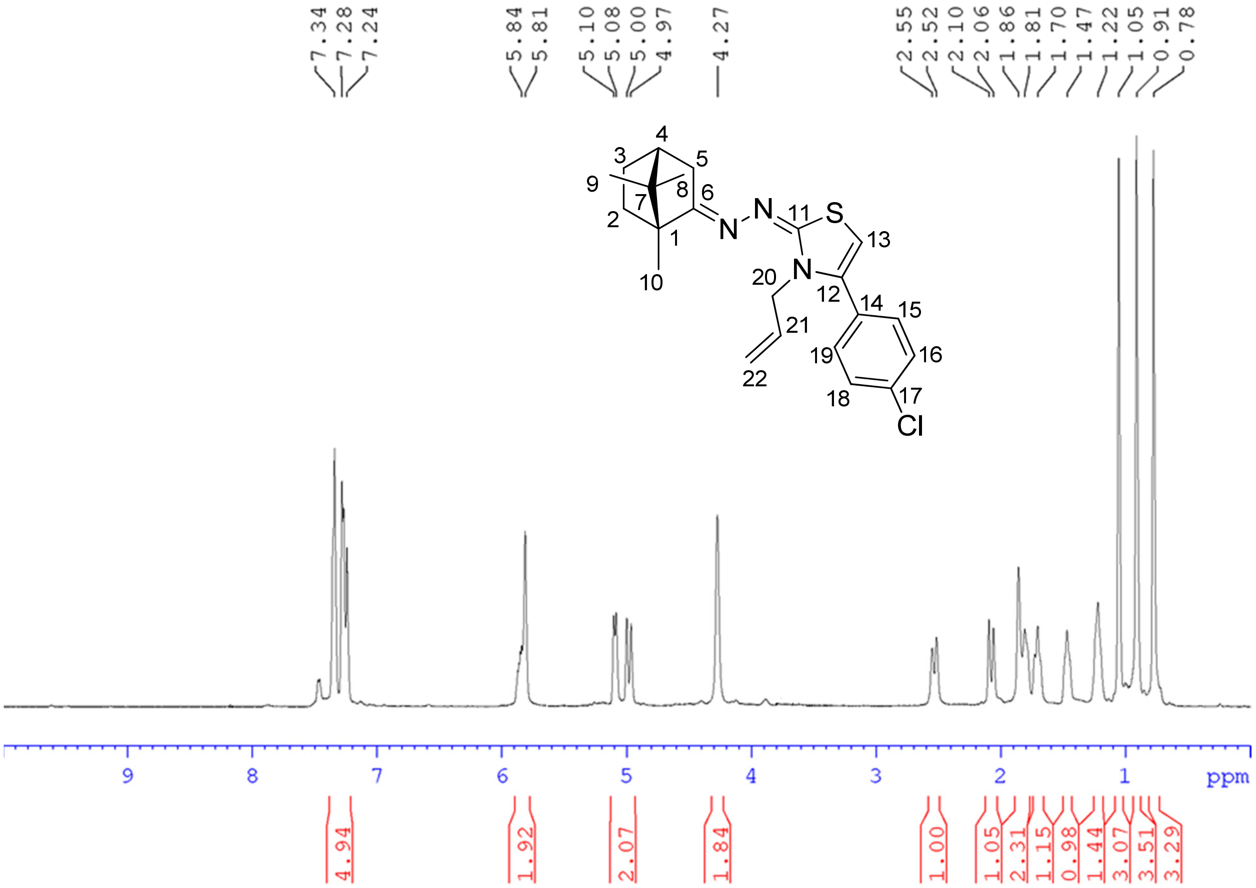

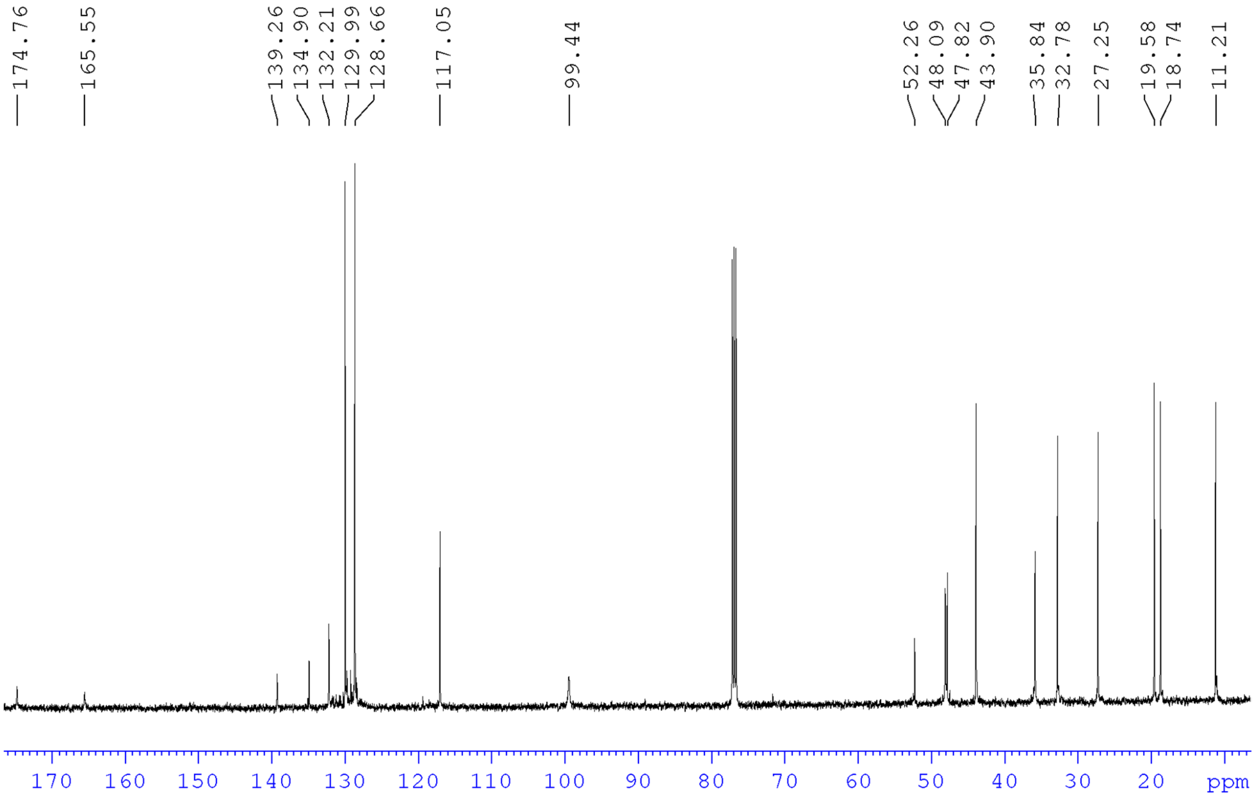

1.23. ^{1}H NMR and ^{13}C NMR spectra of compound 5c

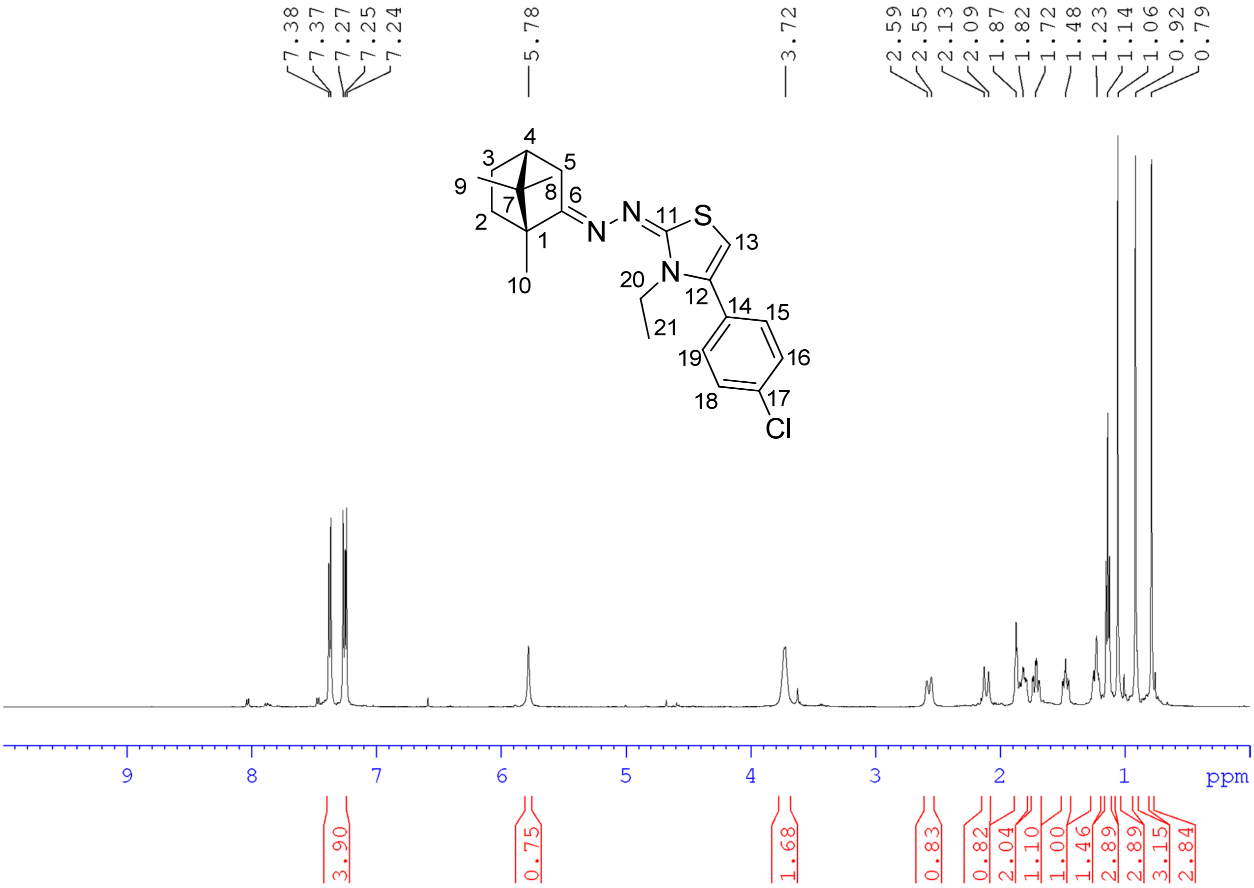

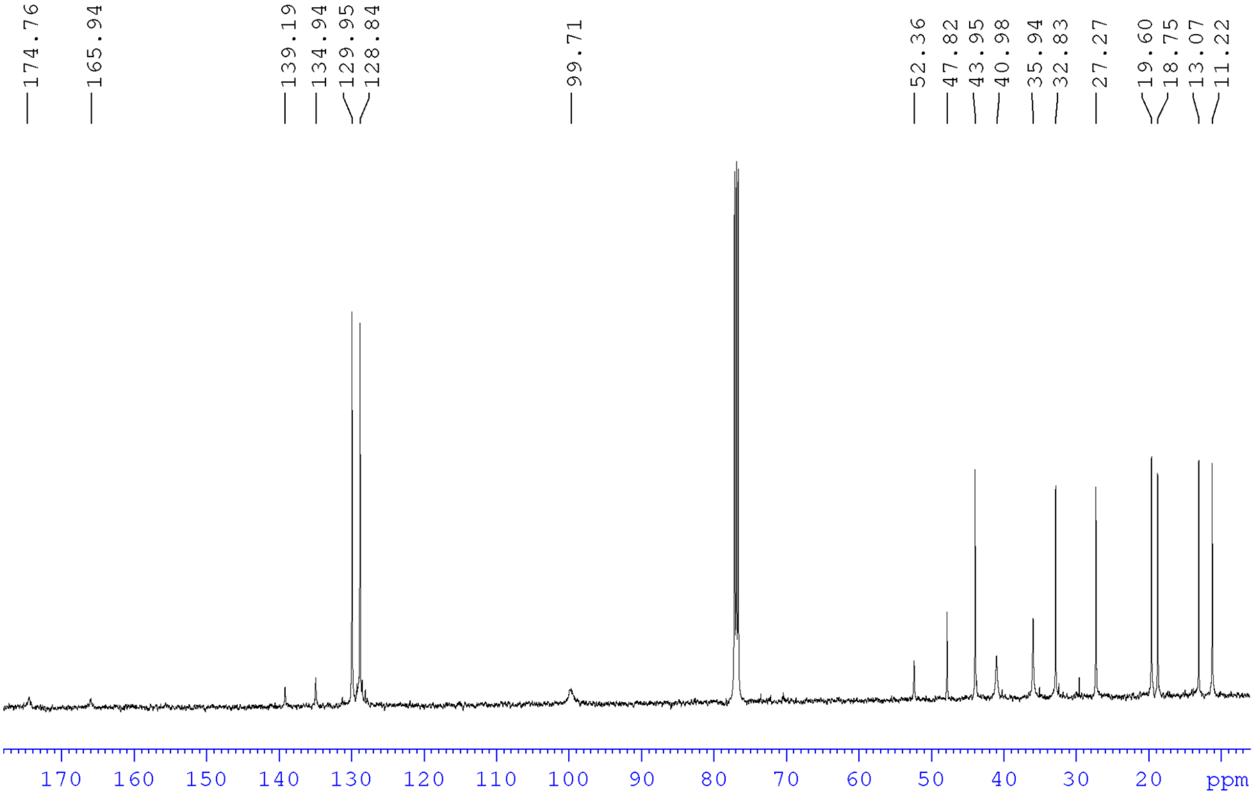

1.24. ^{1}H NMR and ^{13}C NMR spectra of compound 5d

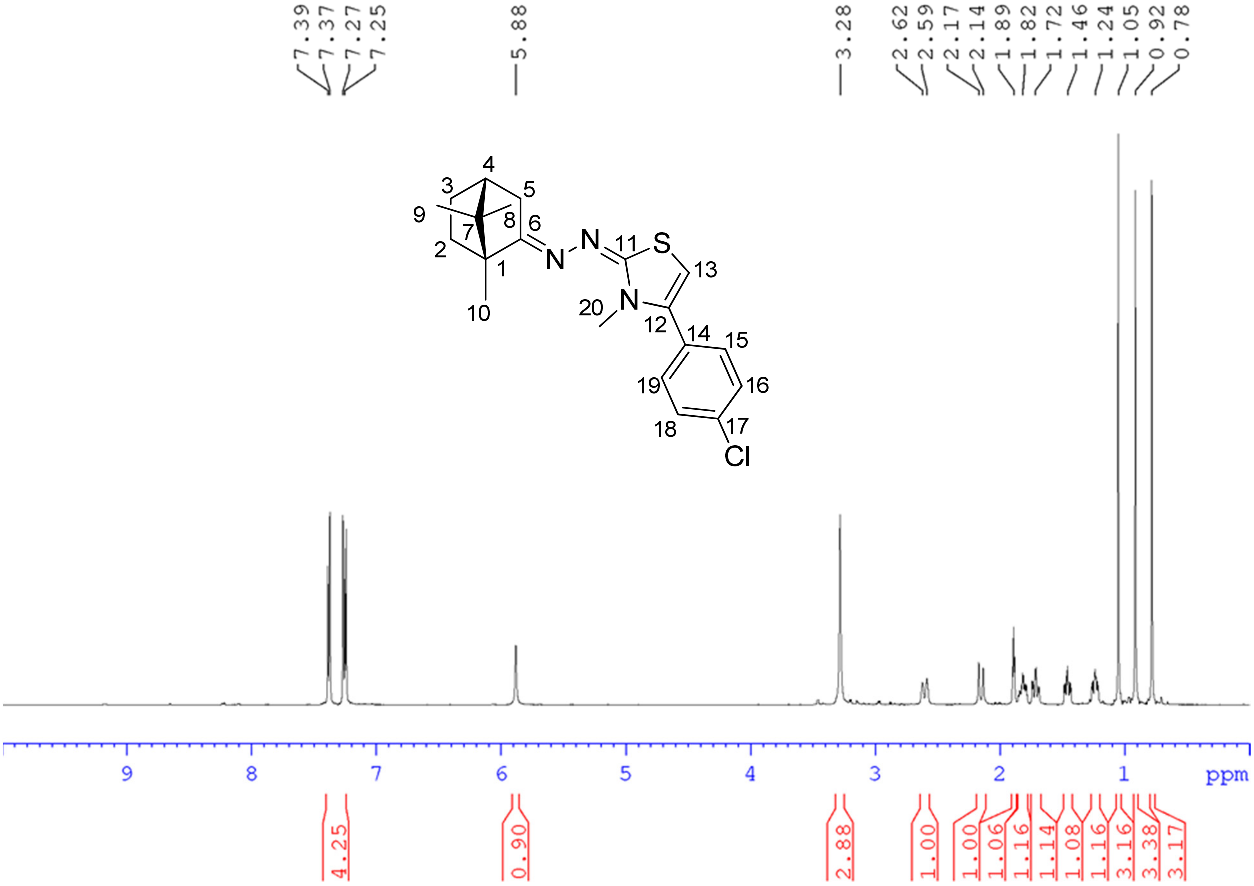

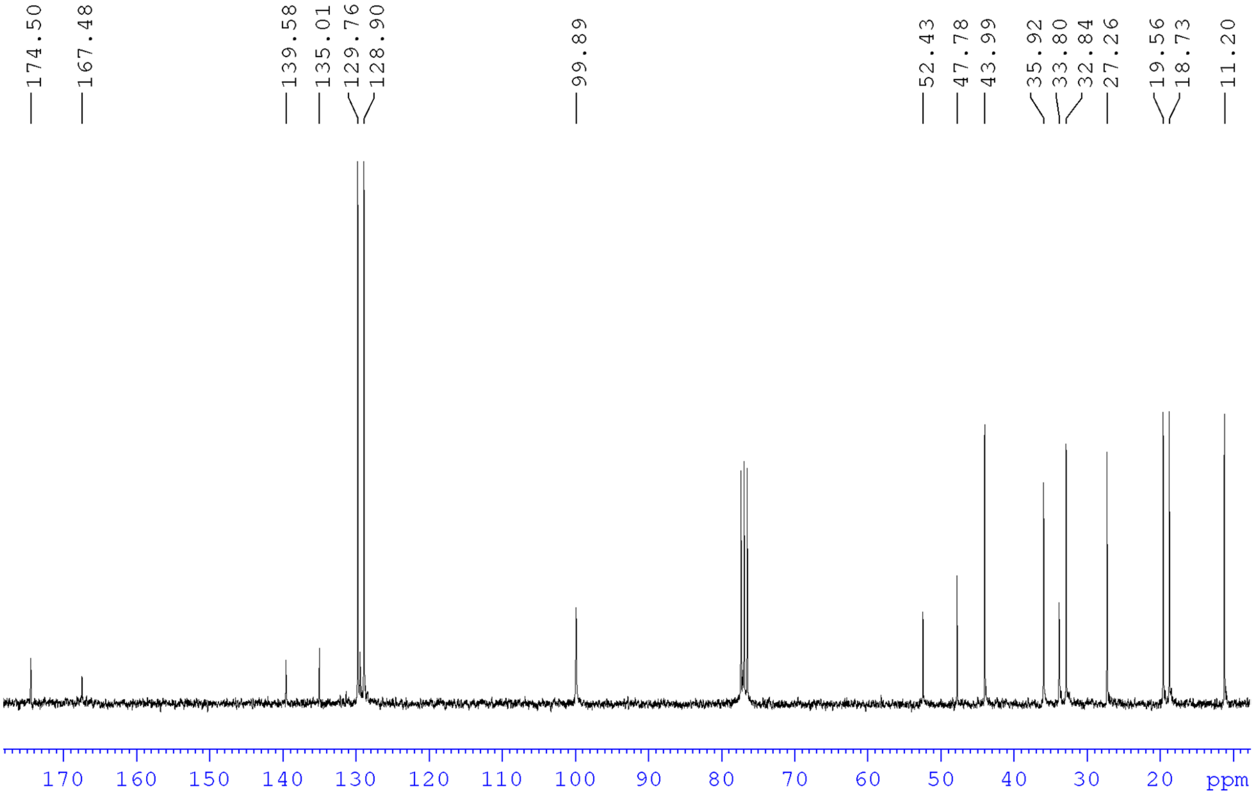

1.25. ¹H NMR and ¹³C NMR spectra of compound 6a

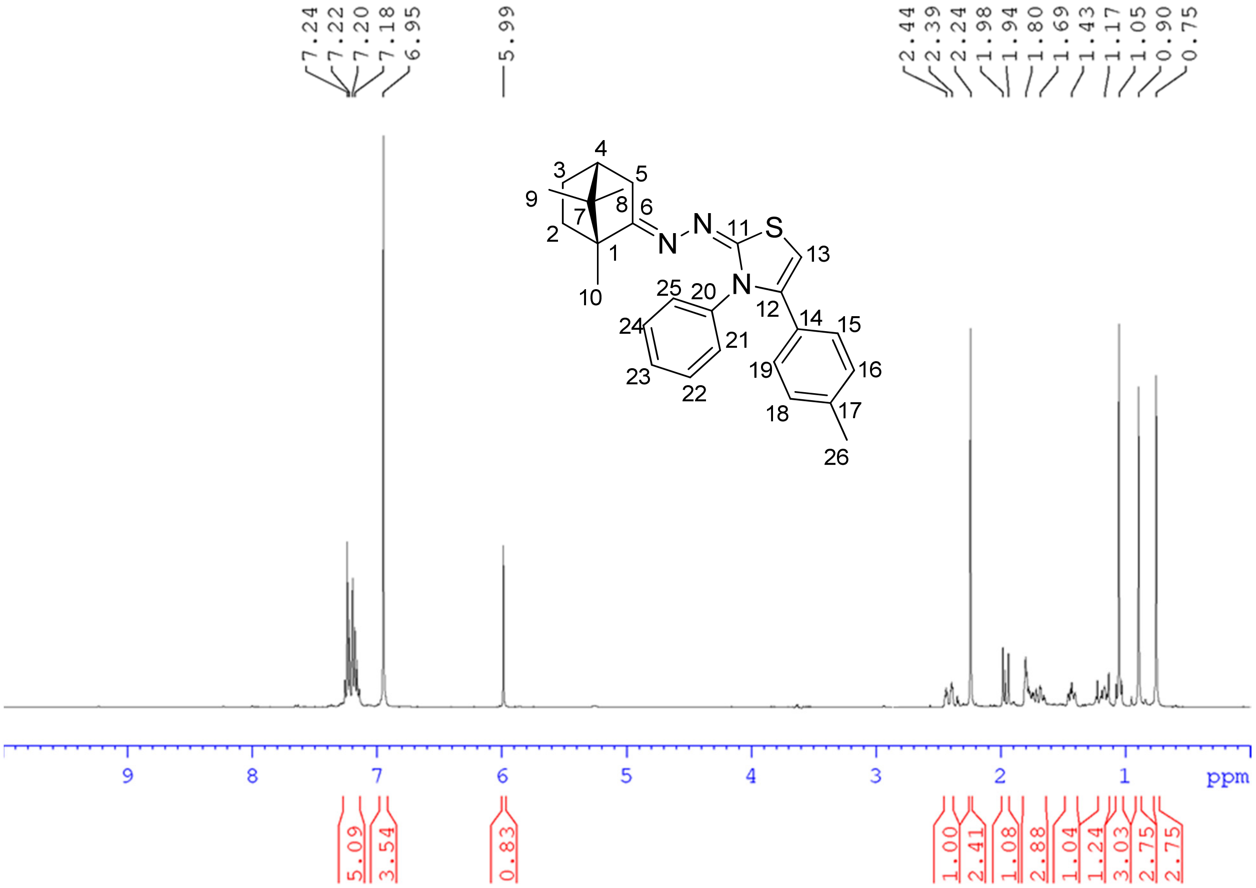

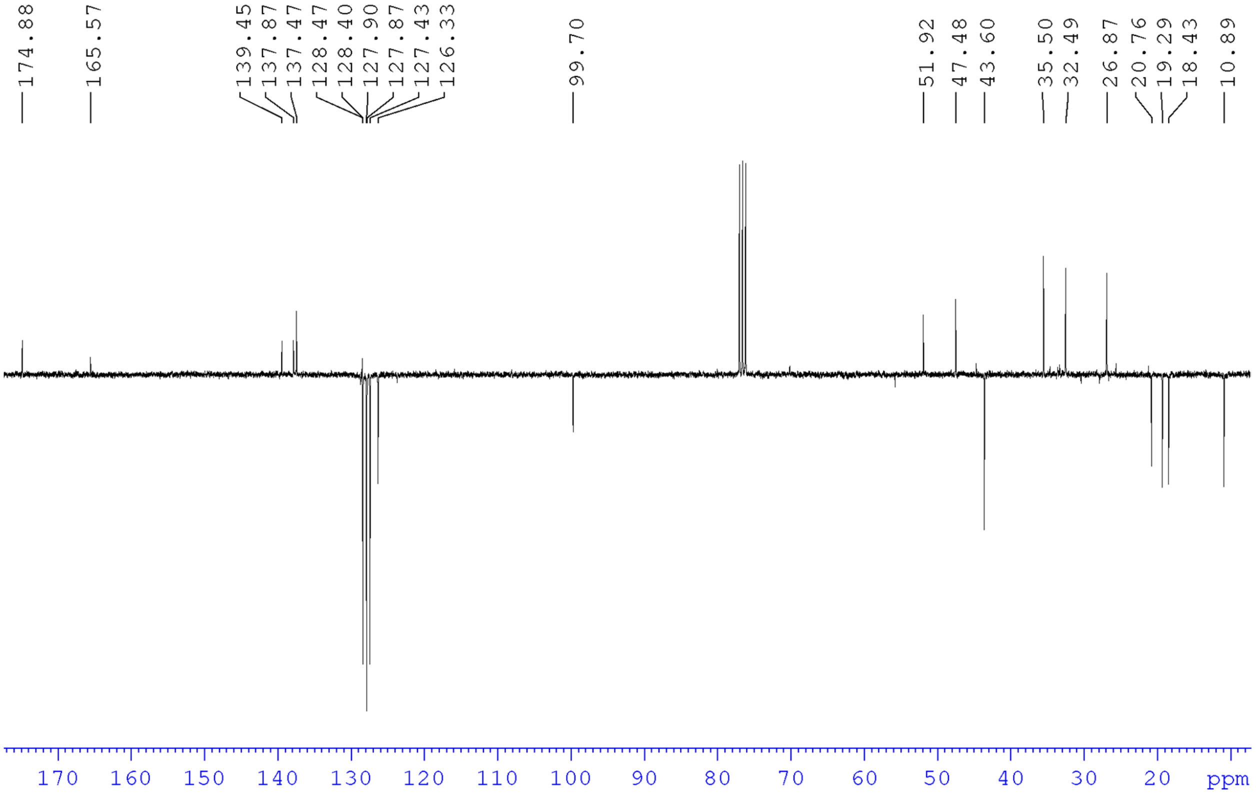

1.26. ^{1}H NMR and ^{13}C NMR spectra of compound 6b

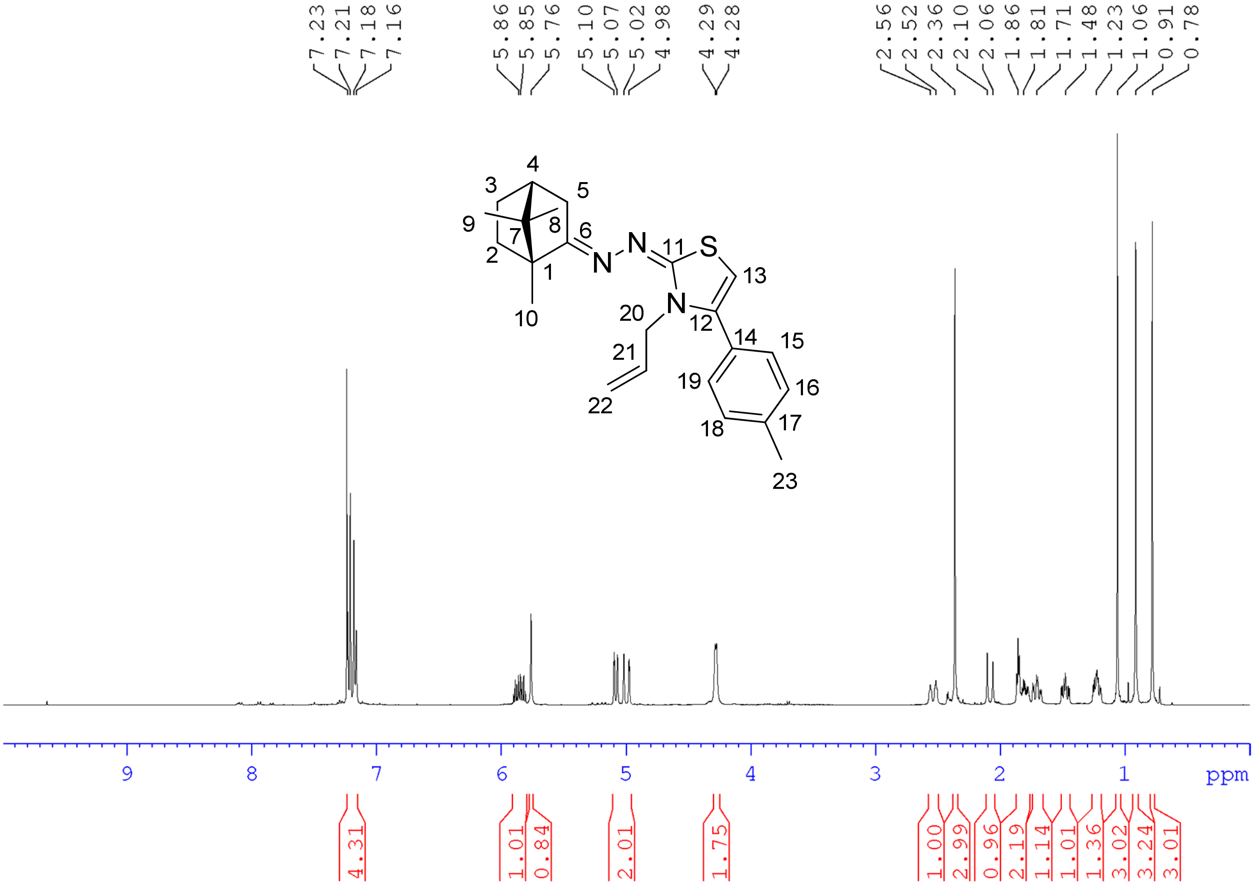

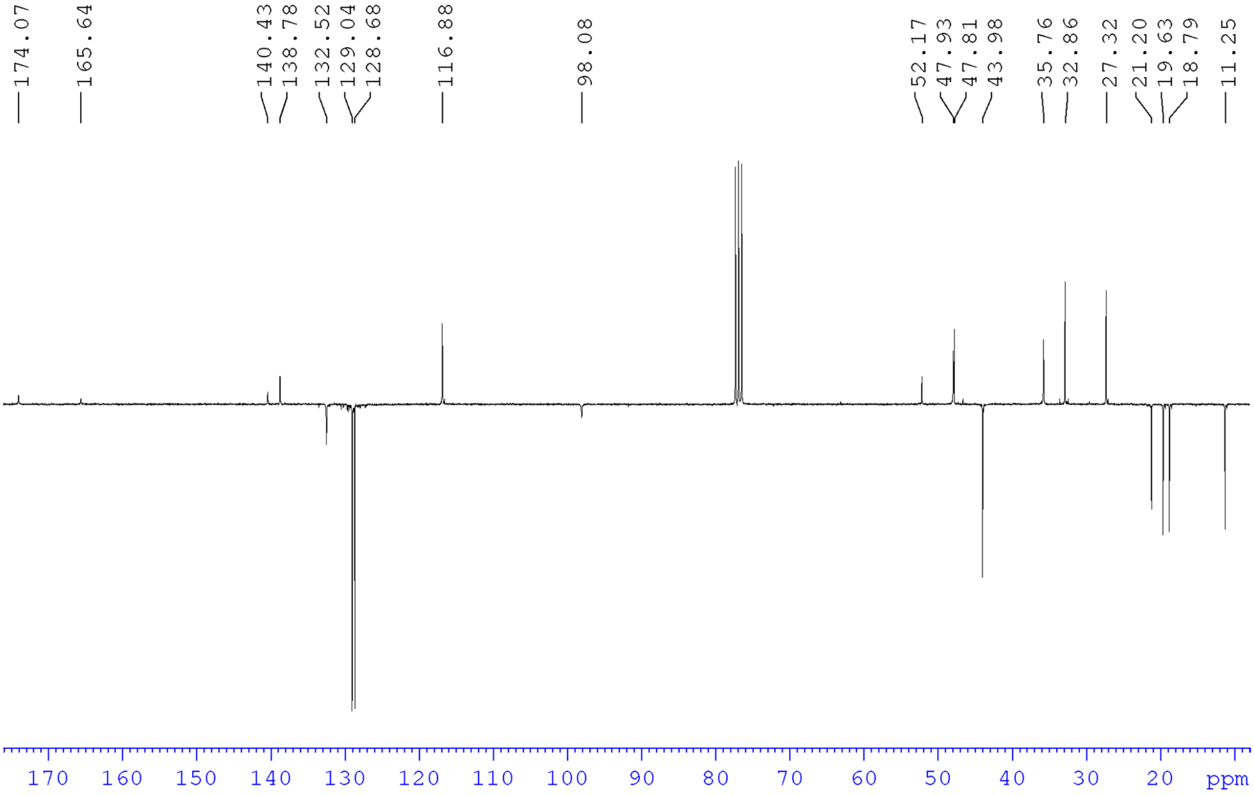

1.27. [1]**H NMR and** [13]**C NMR spectra of compound 6c**

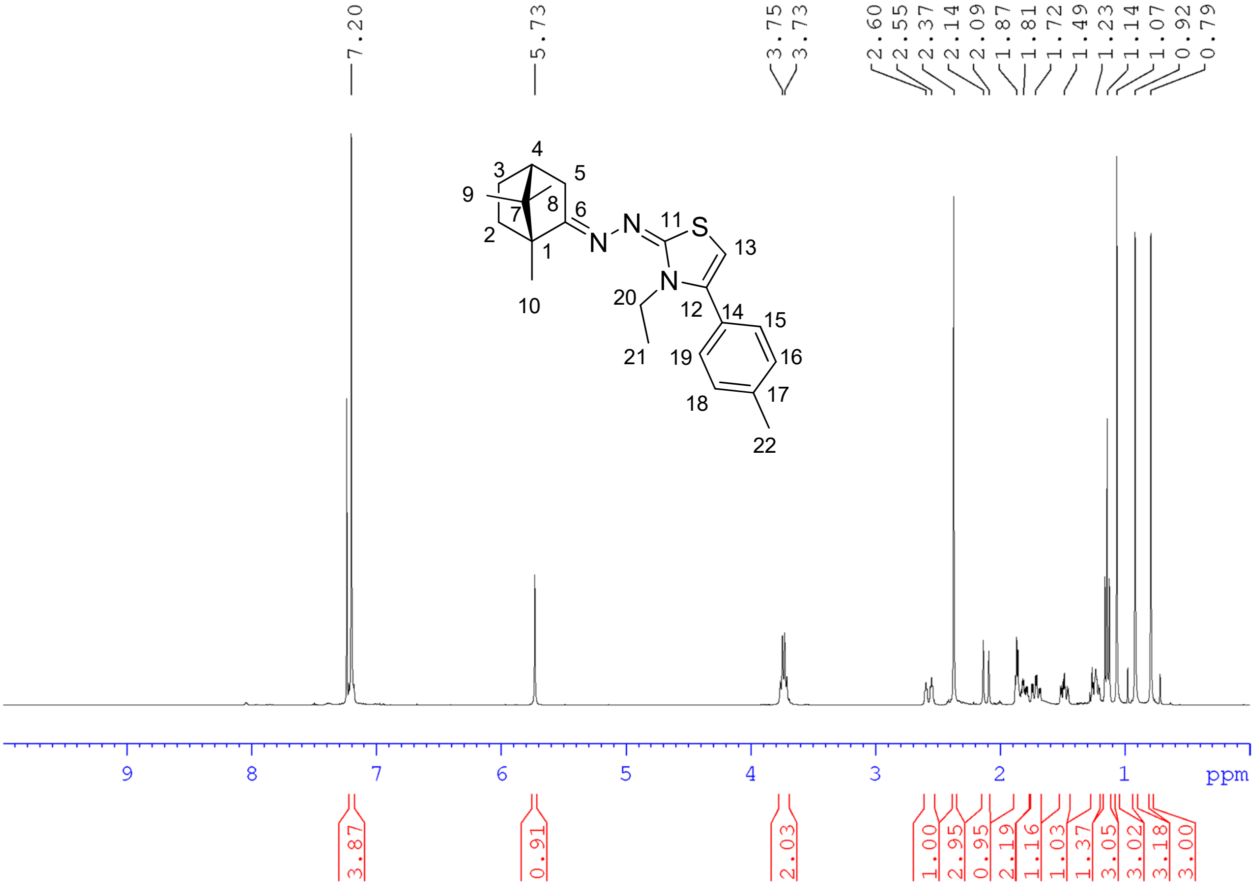

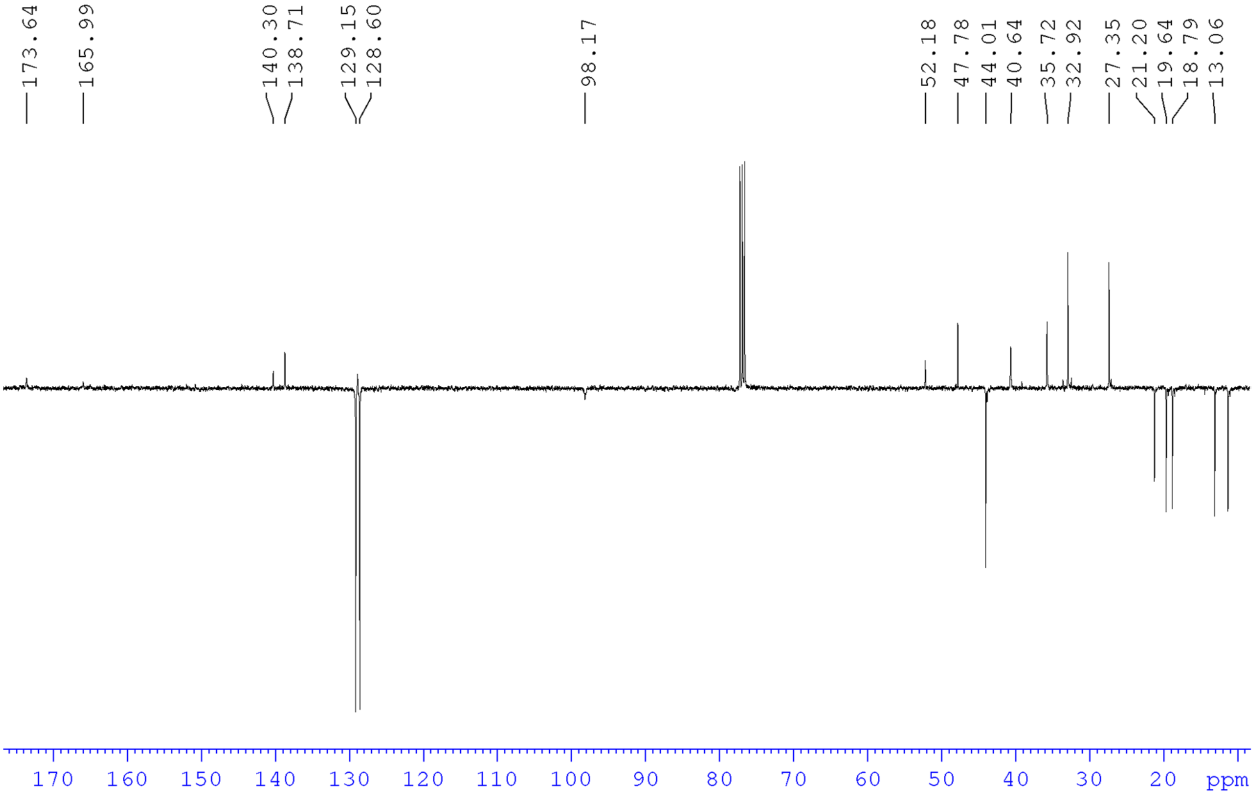

1.28. [1]**H NMR and** [13]**C NMR spectra of compound 6d**

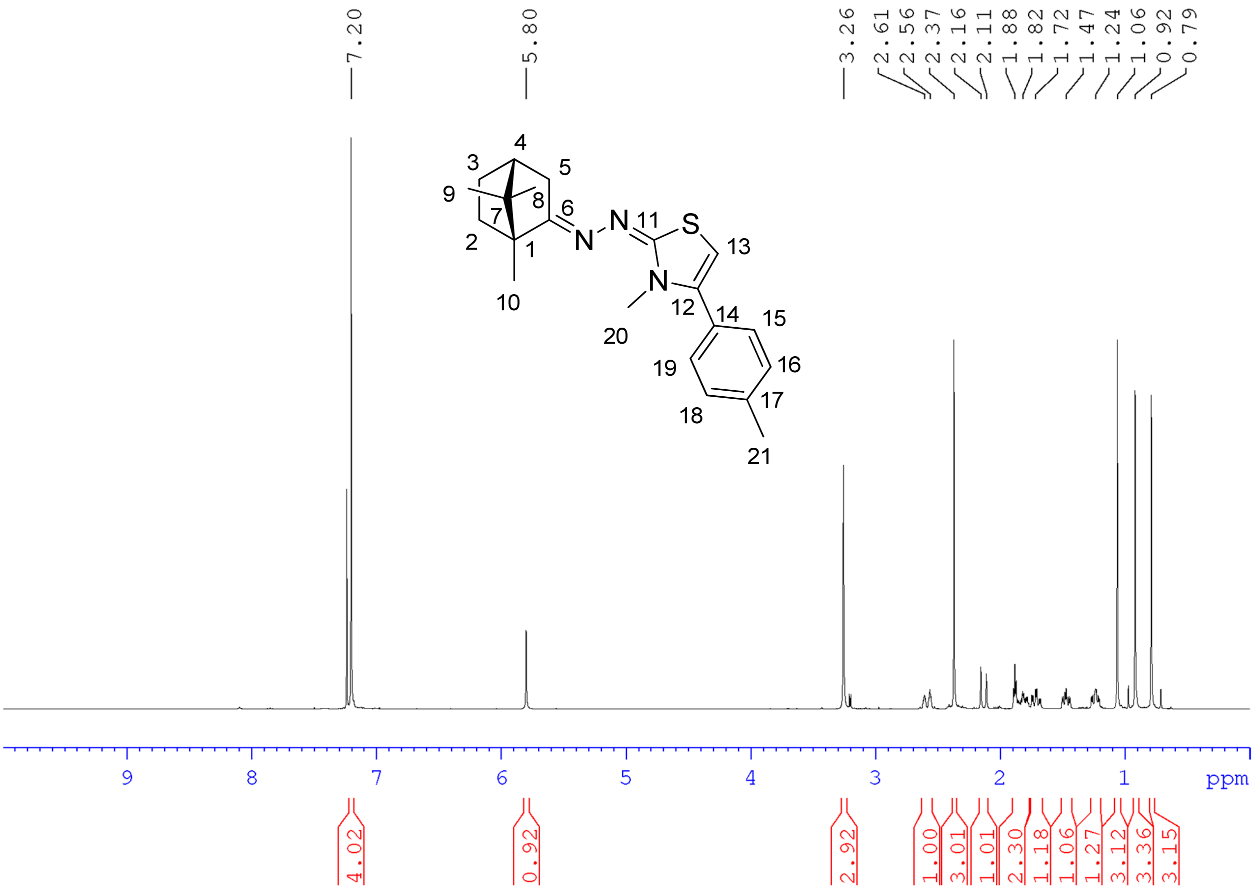

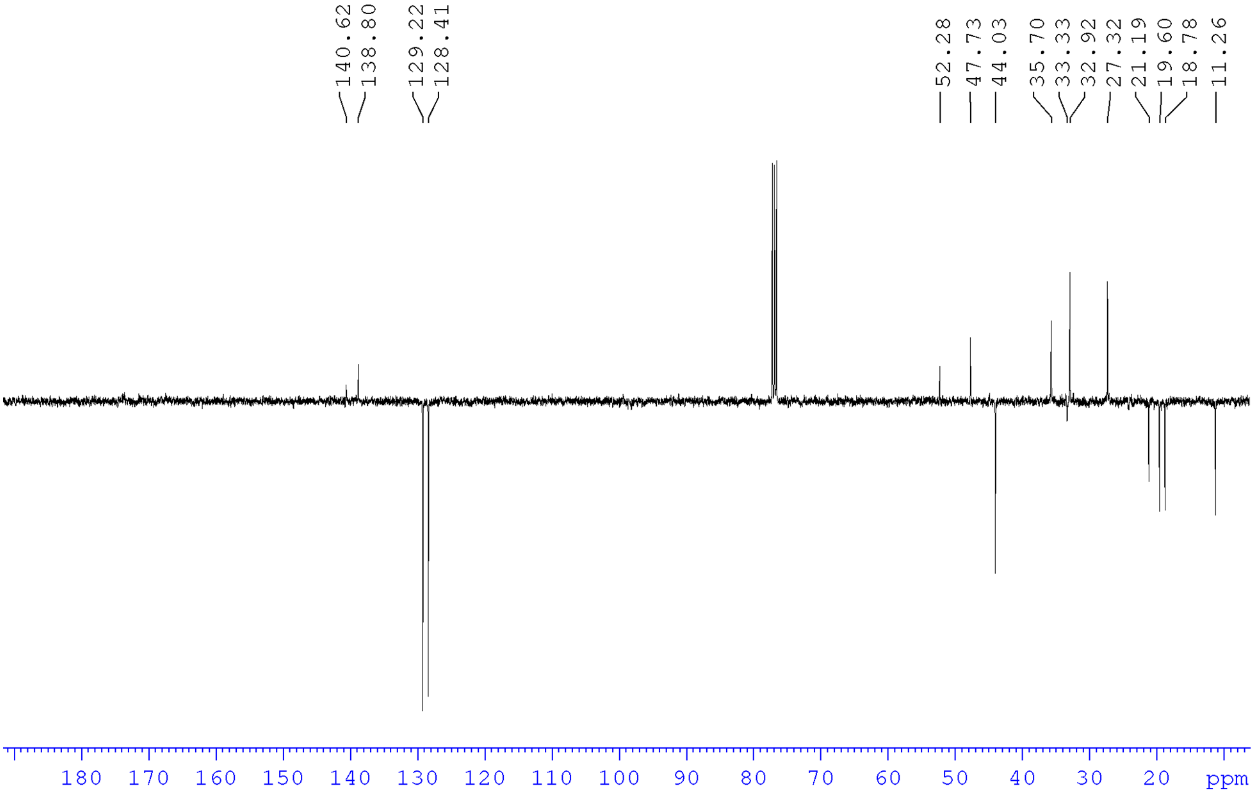

1.29. ^{1}H NMR and ^{13}C NMR spectra of compound 7a

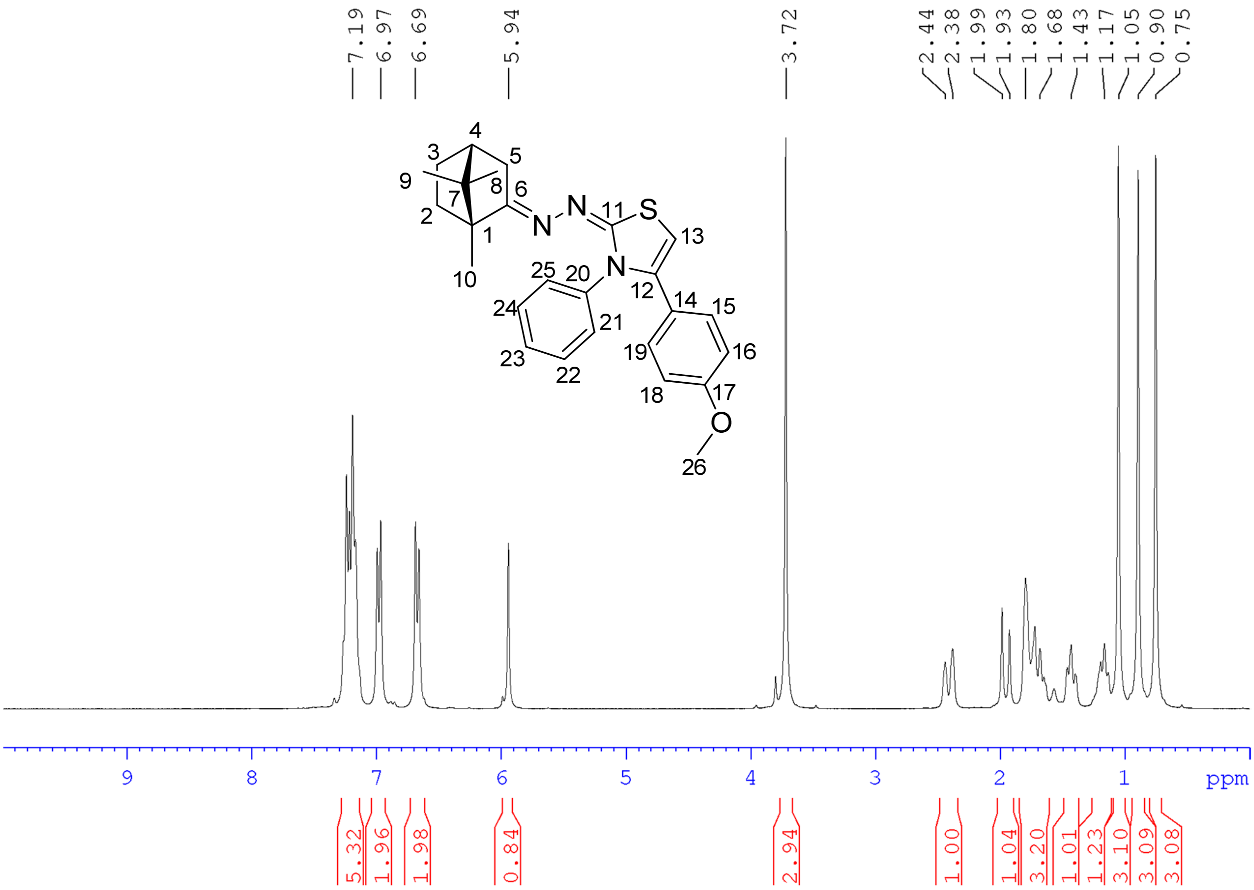

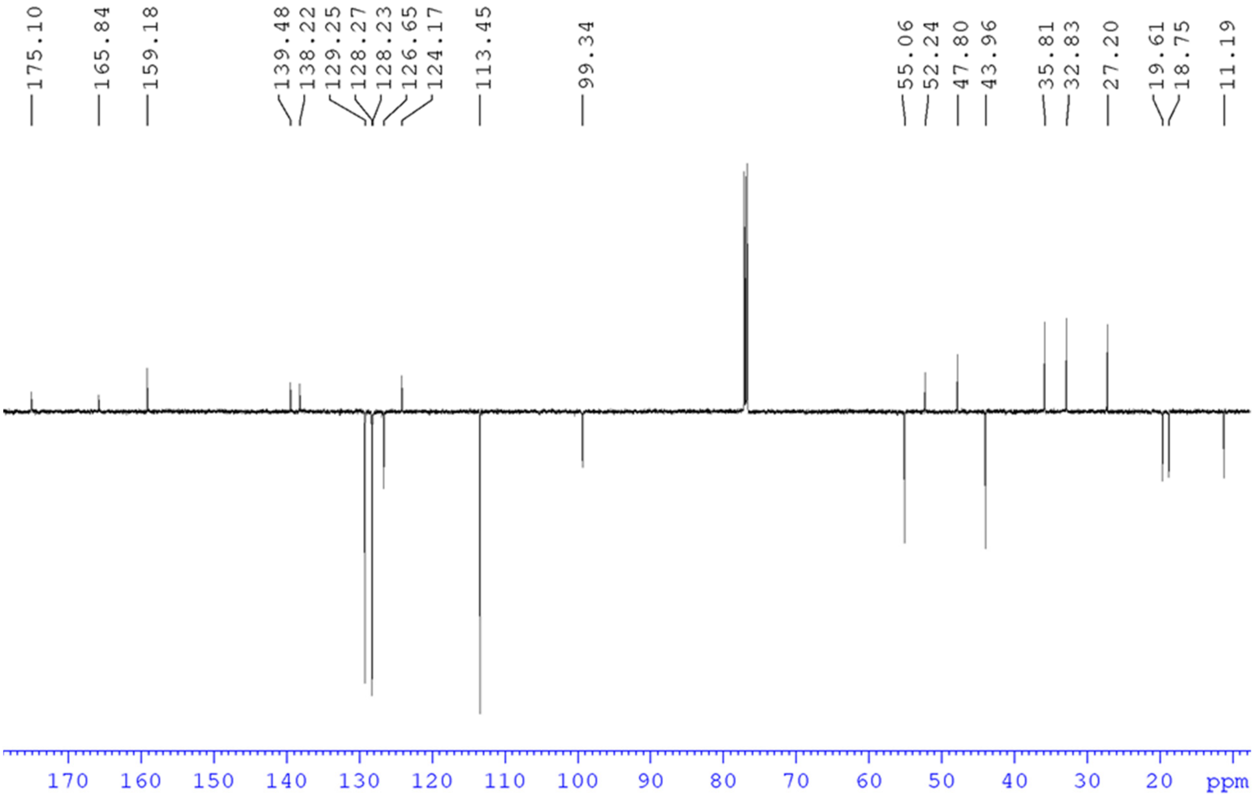

1.30. ^{1}H NMR and ^{13}C NMR spectra of compound 7b

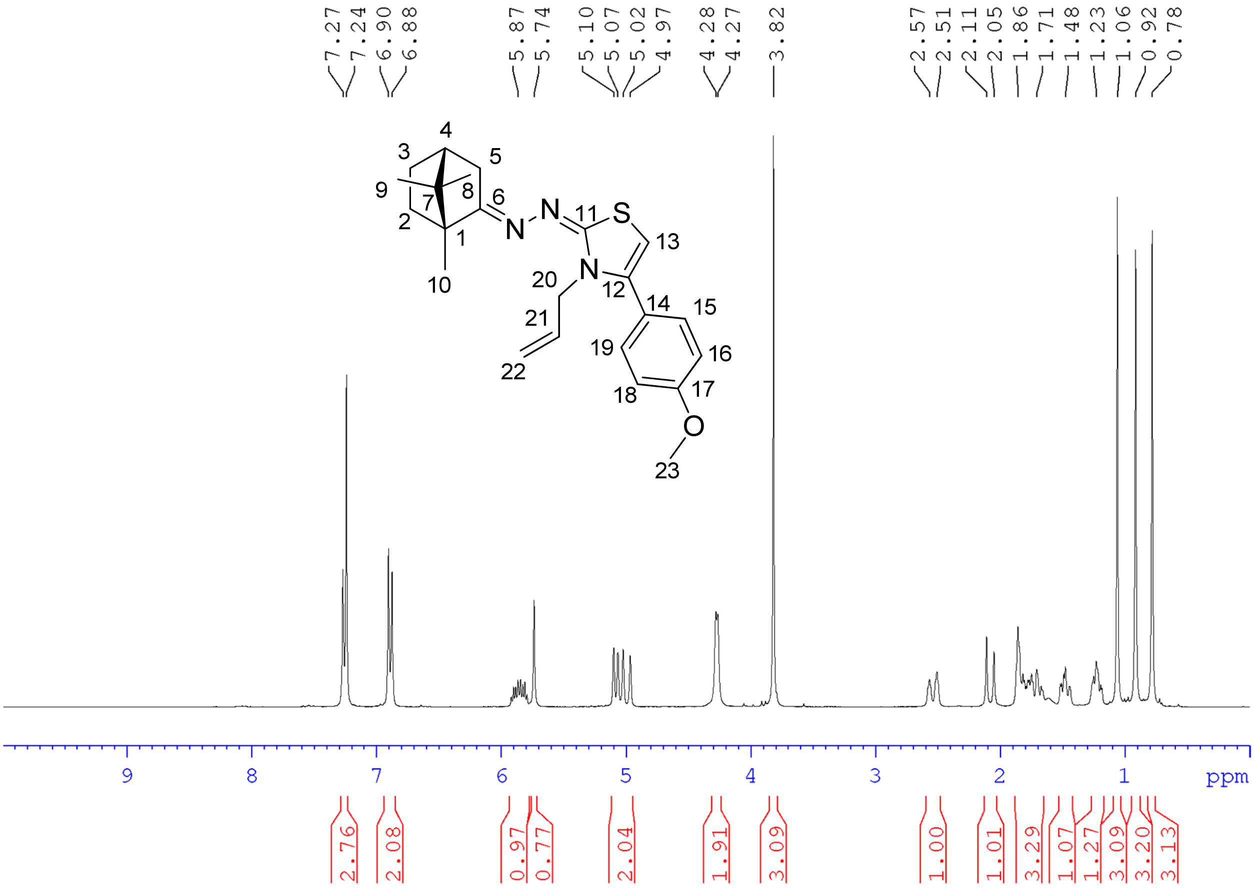

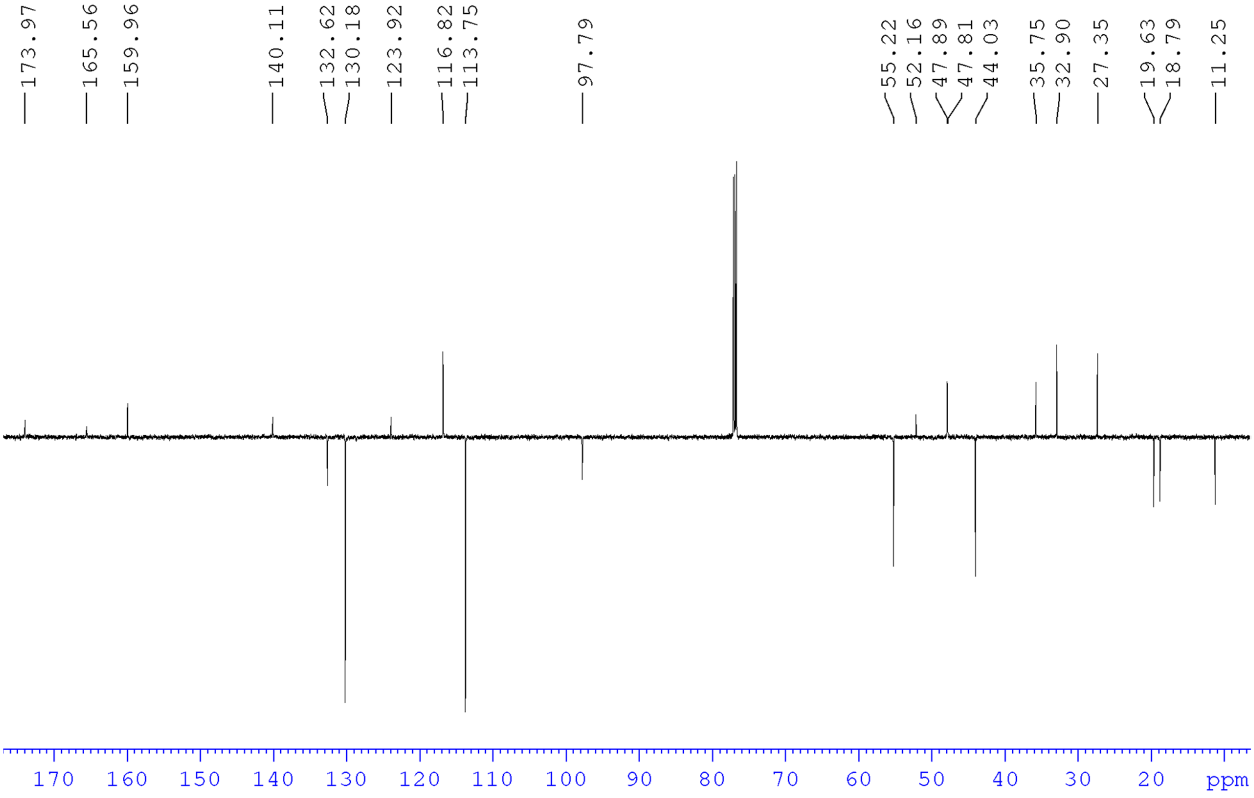

1.31. ^{1}**H NMR and** ^{13}**C NMR spectra of compound 7c**

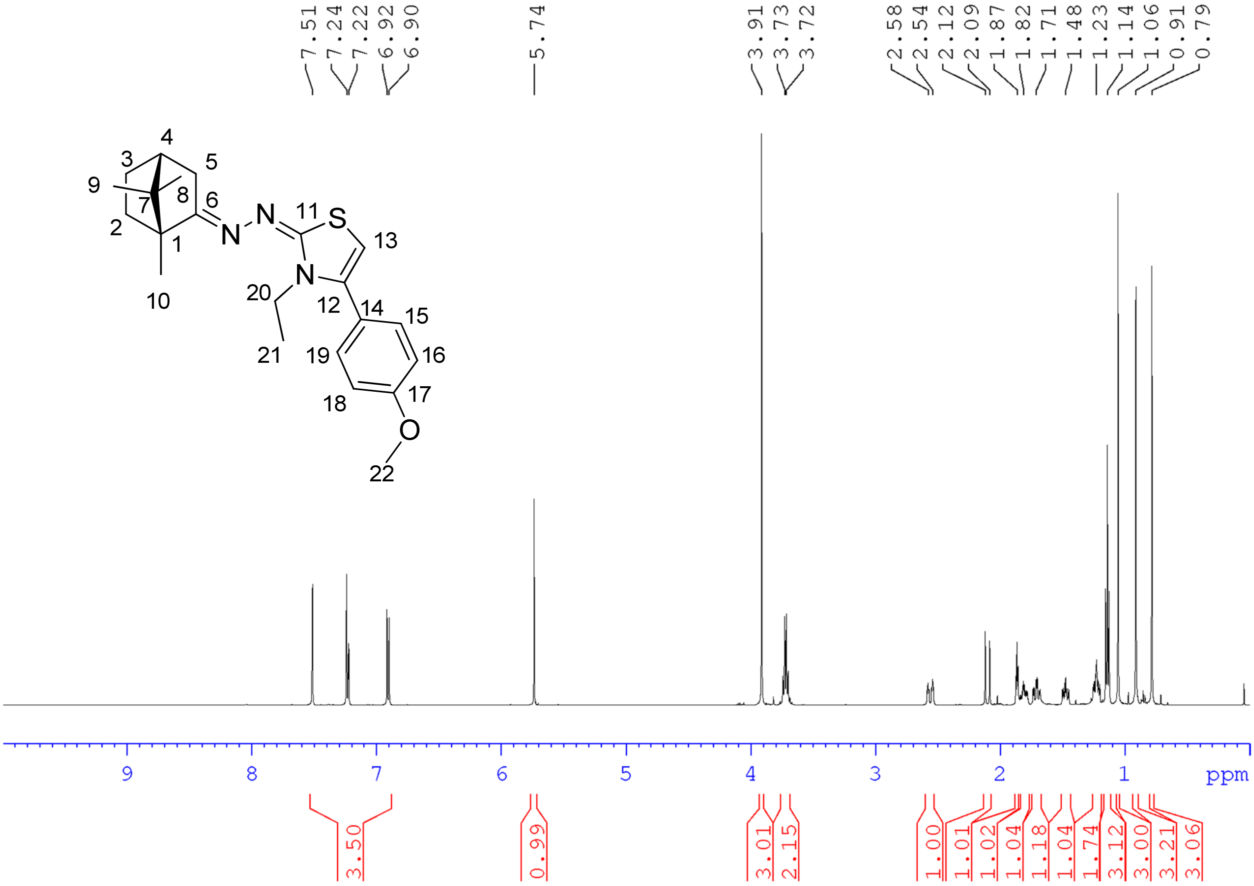

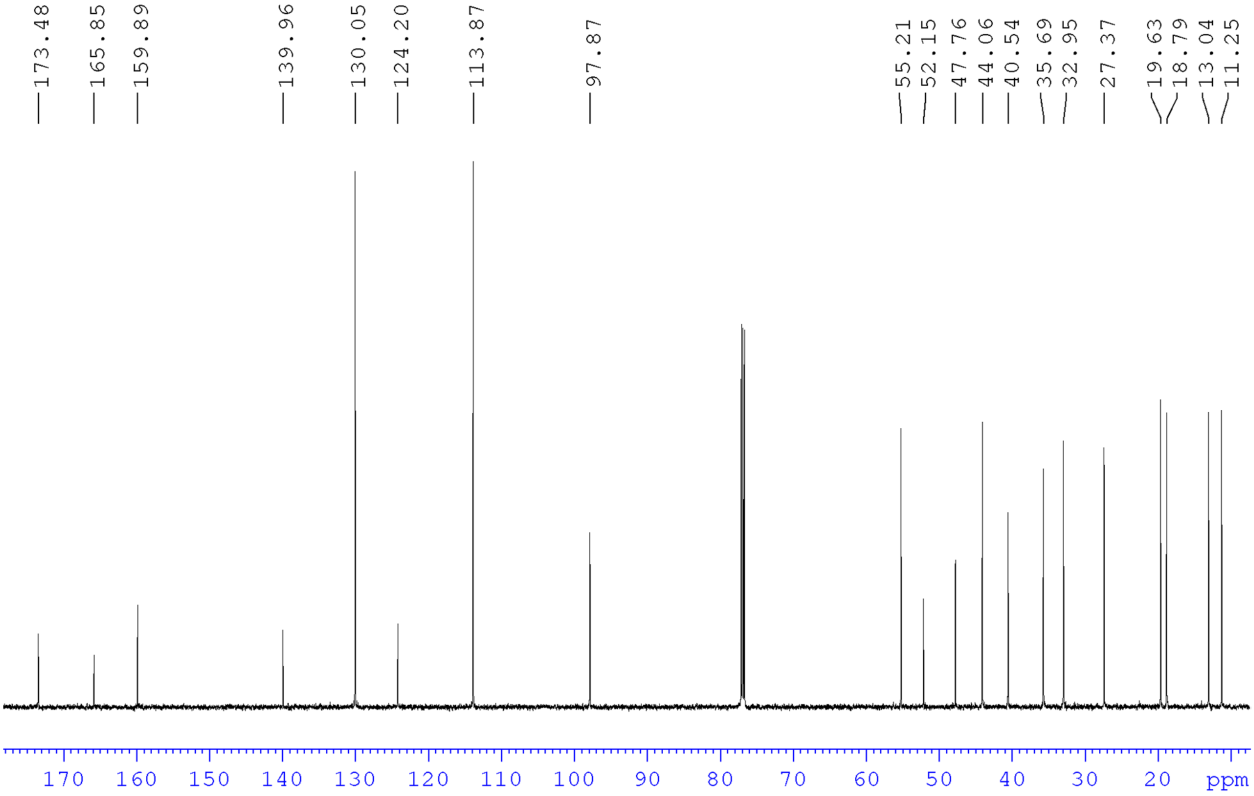

1.32. ¹H NMR and ¹³C NMR spectra of compound 7d

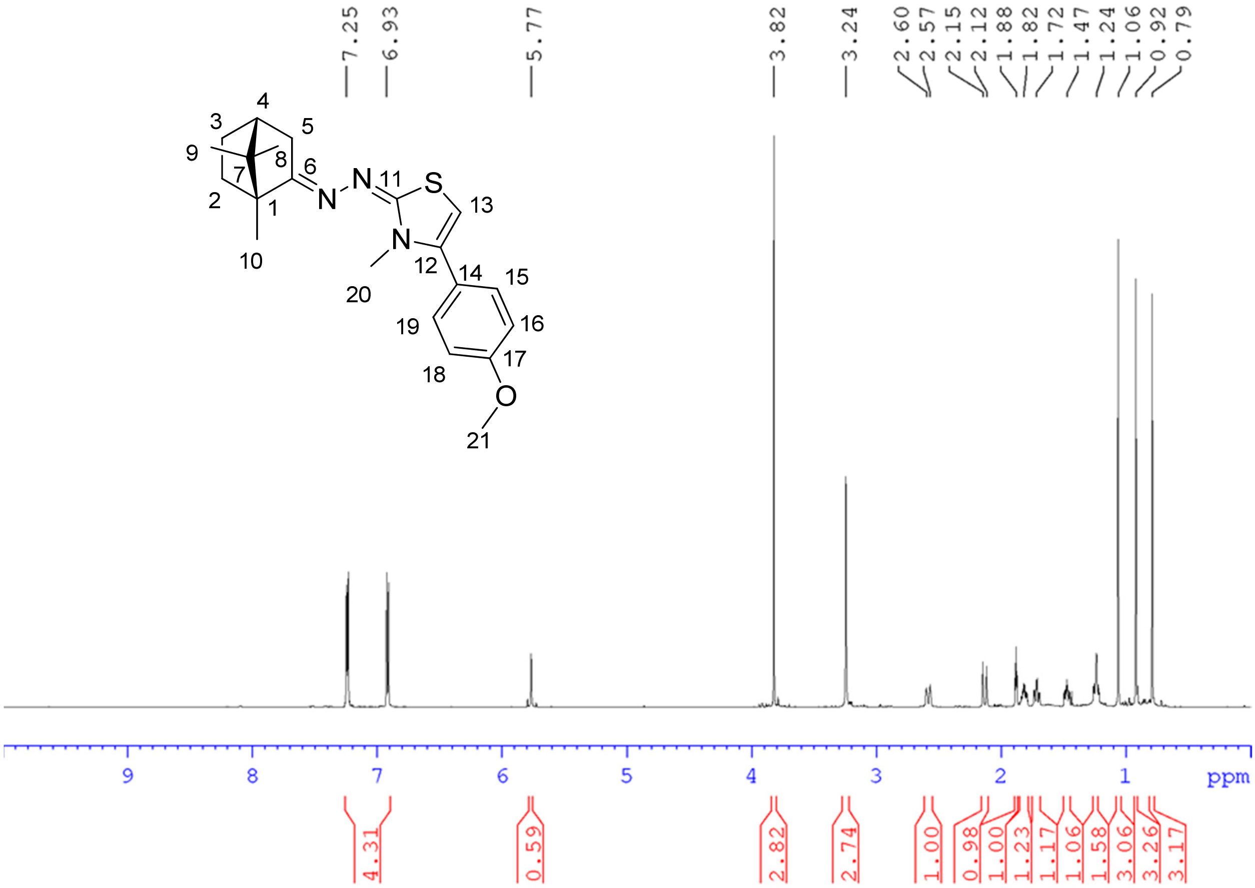

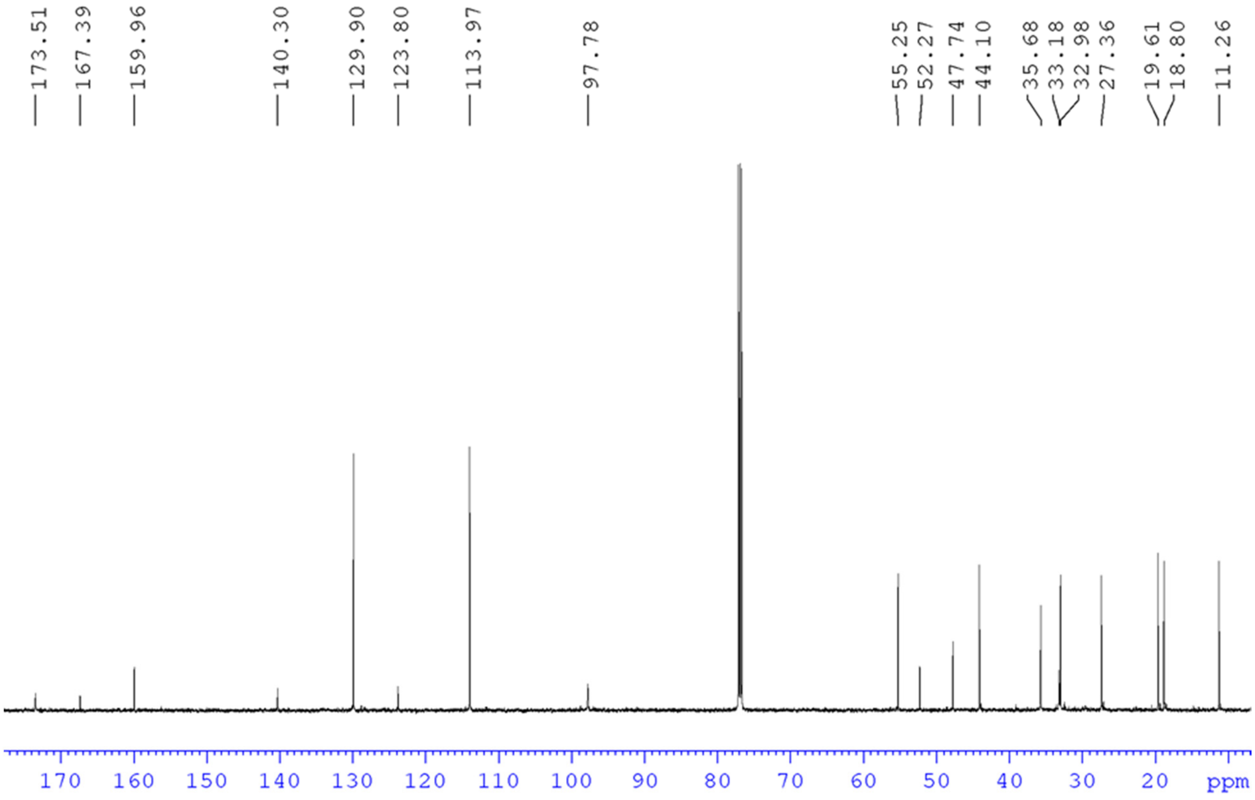

1.33. **^{1}H NMR and ^{13}C NMR spectra of compound 8a**

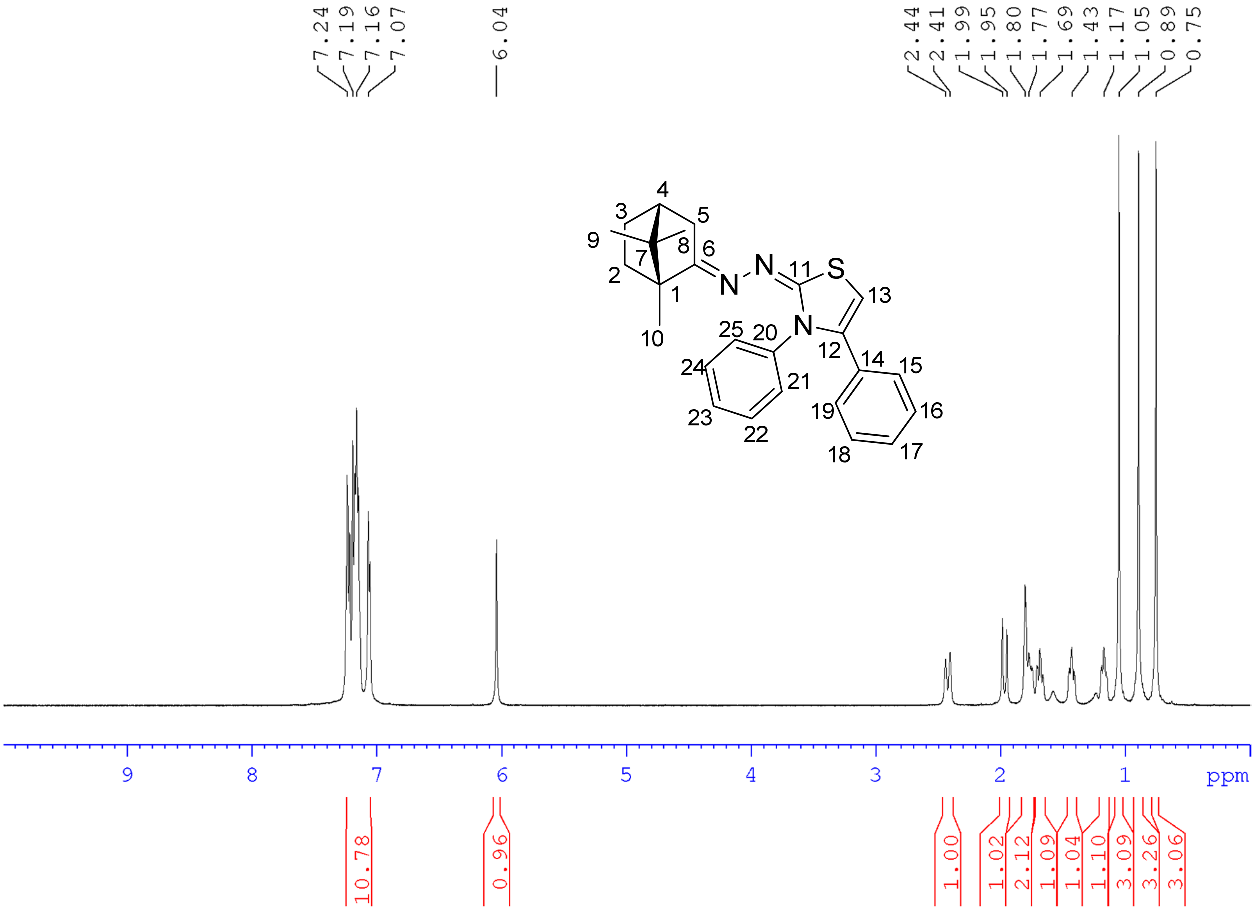

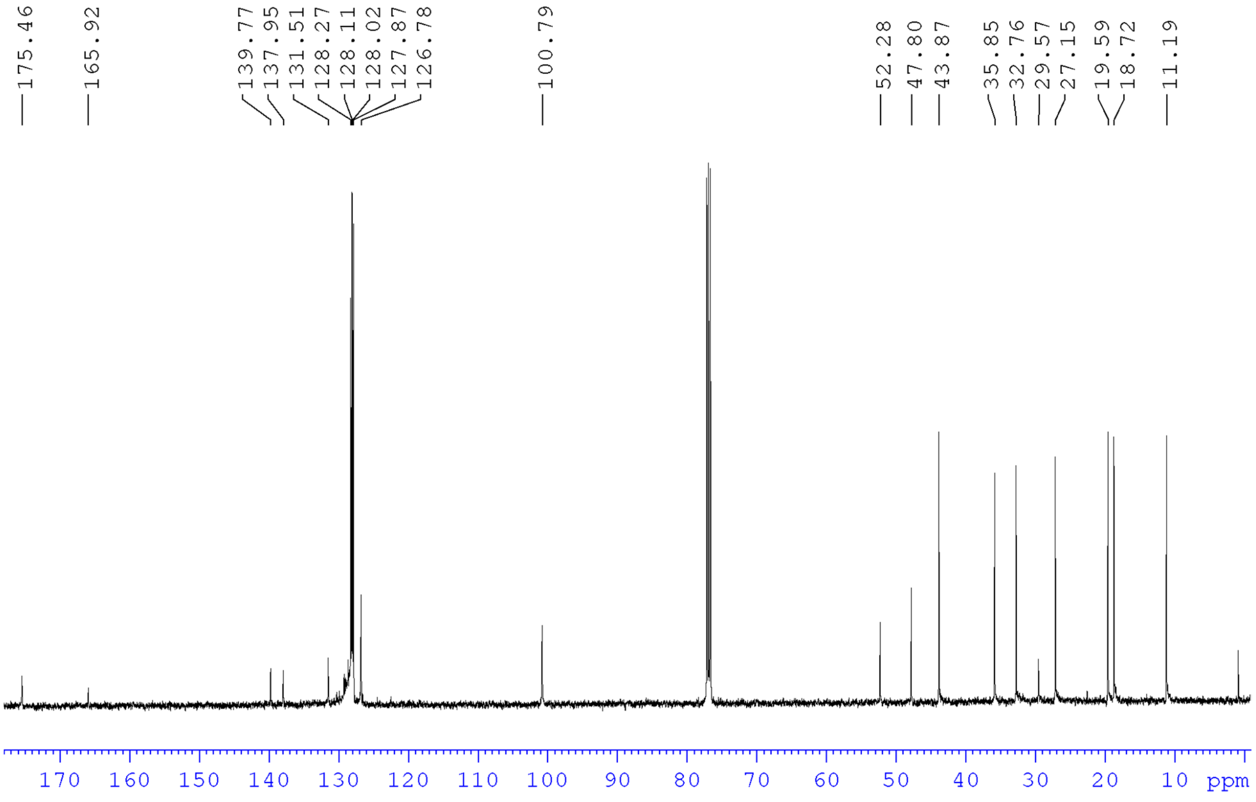

1.34. **^{1}H NMR and ^{13}C NMR spectra of compound 8b**

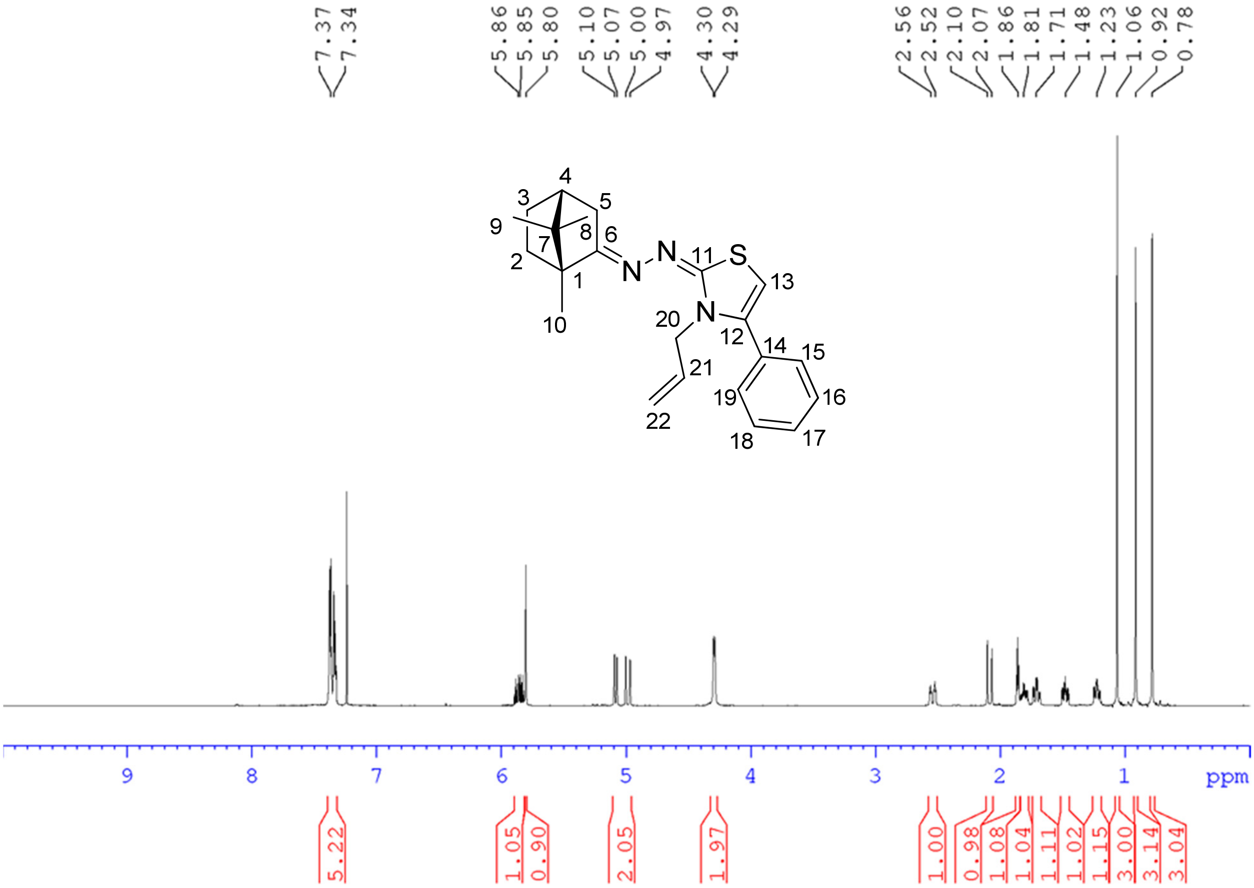

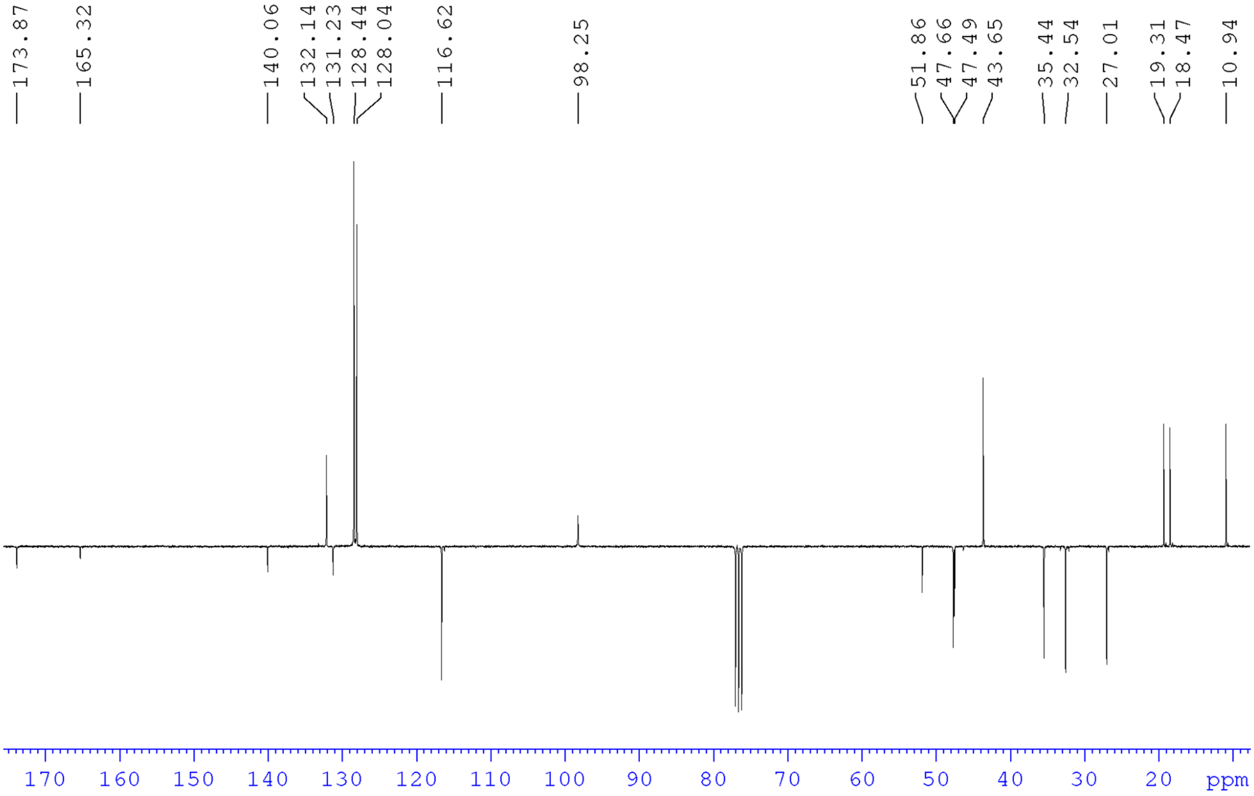

1.35. **¹H NMR and ¹³C NMR spectra of compound 8c**

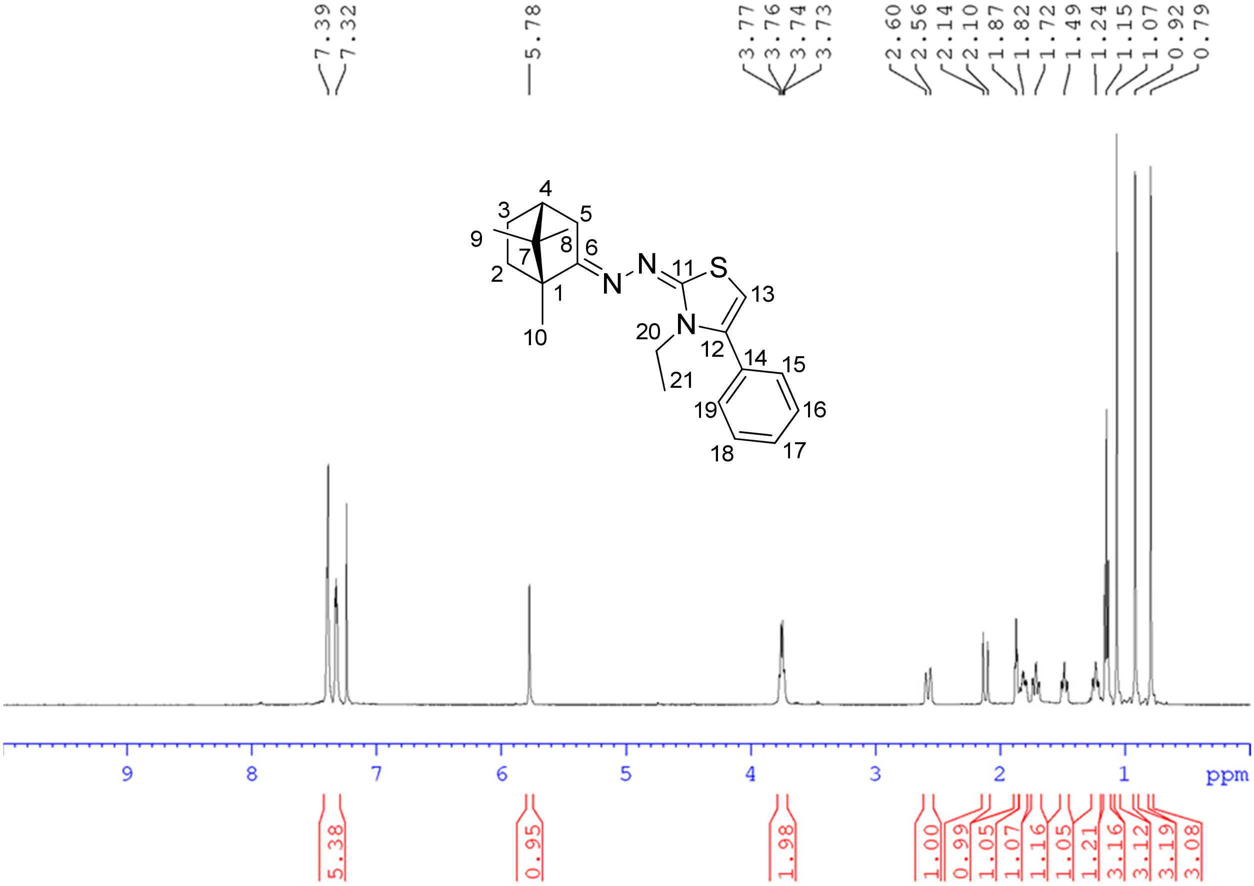

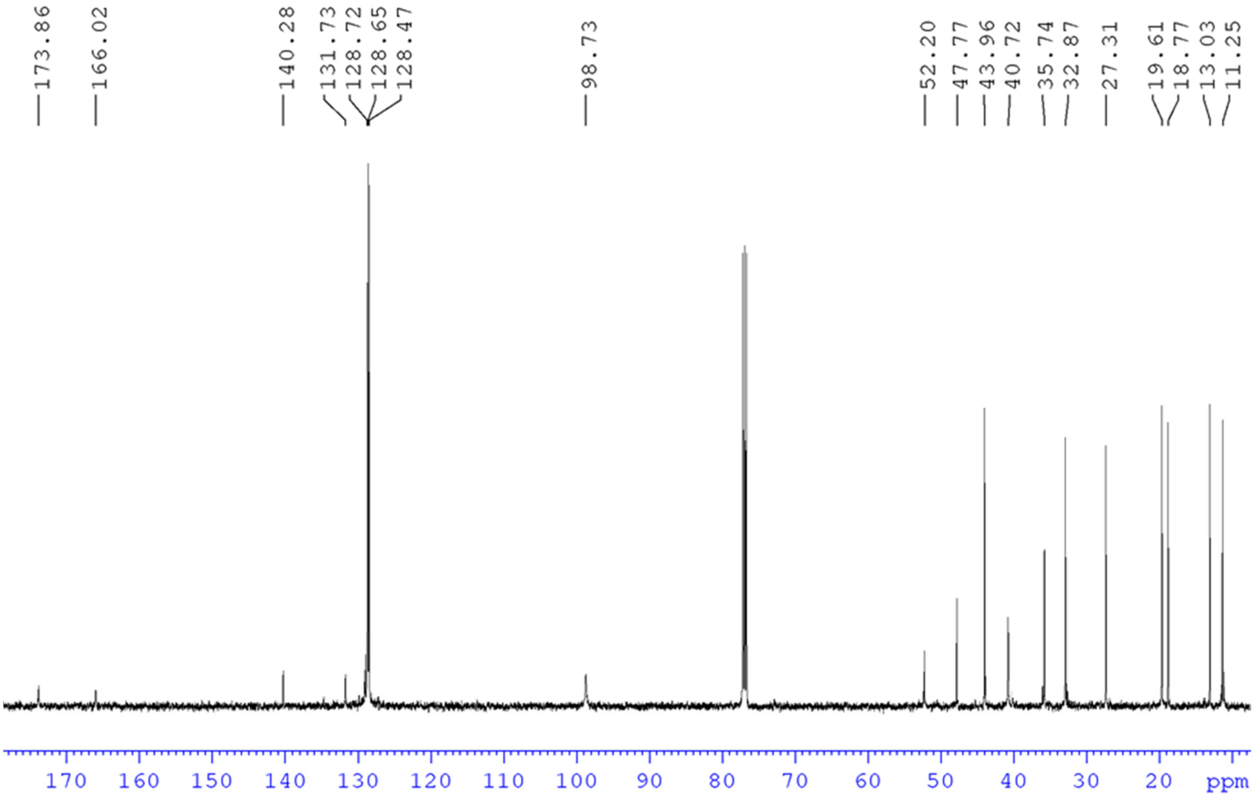

1.36. ^{1}H NMR and ^{13}C NMR spectra of compound 8d

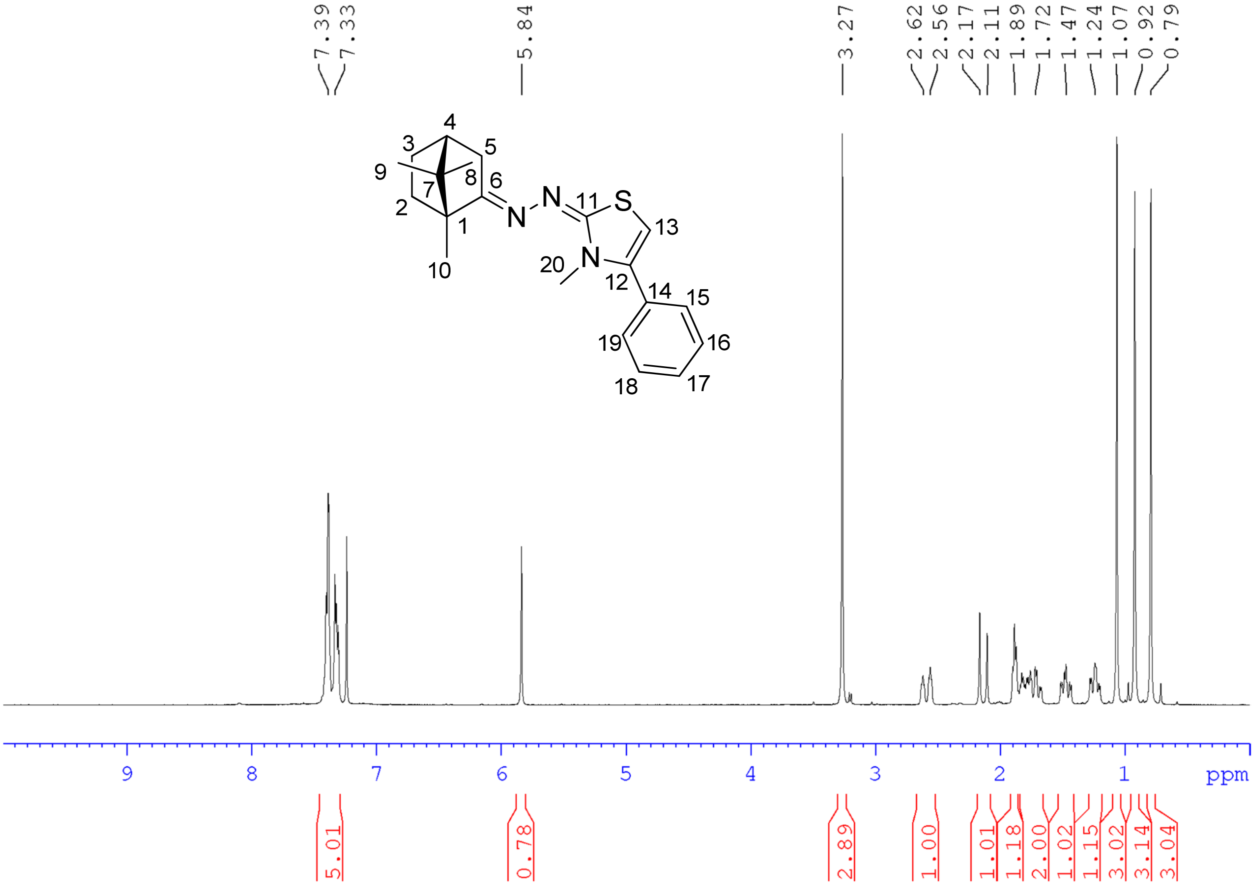

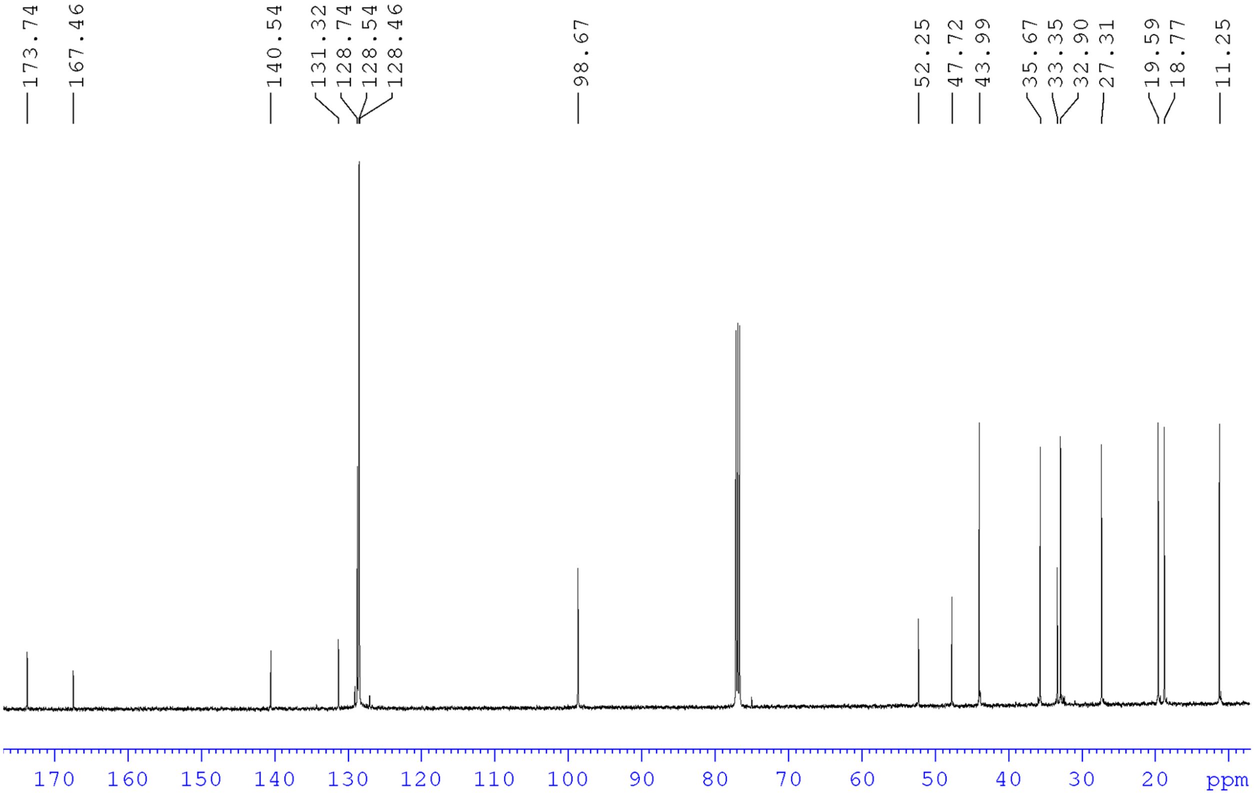

2. High Resolution Mass Spectra of Compounds 2a-8d

2.1. High Resolution Mass Spectra of Compound 2a

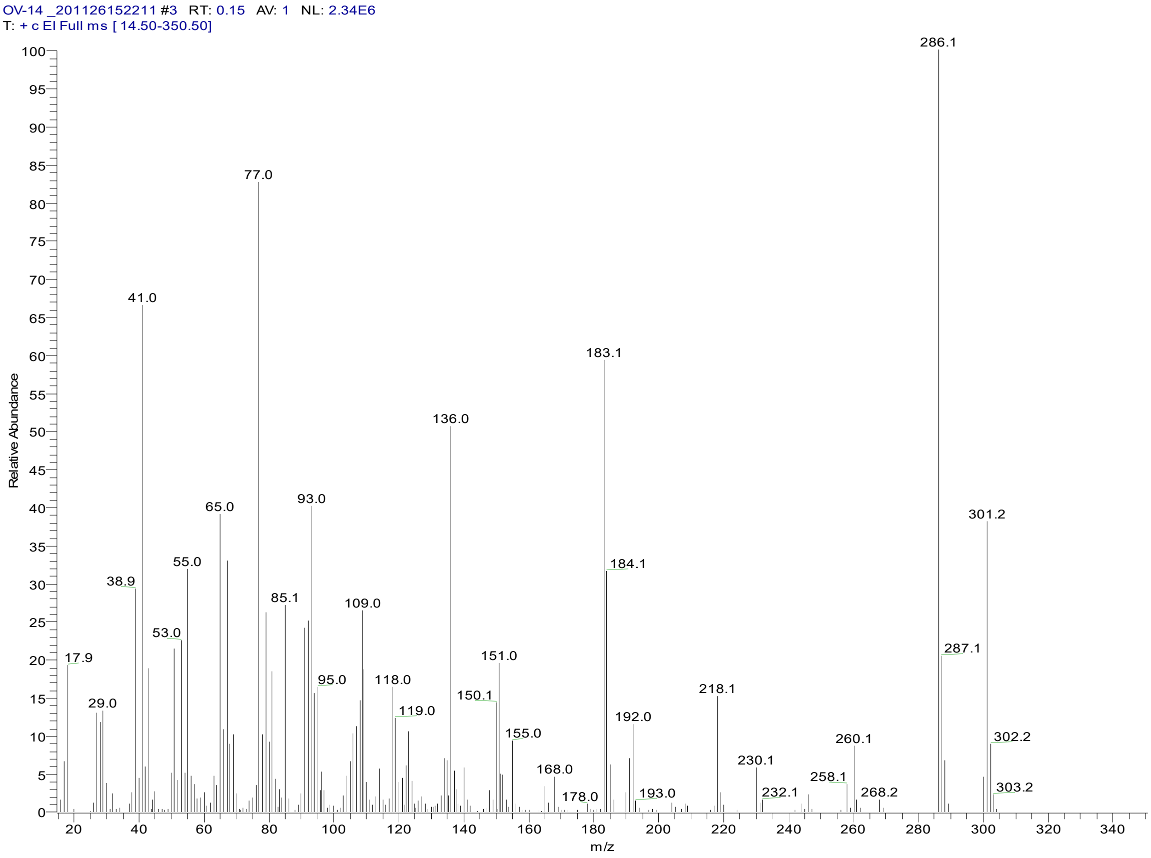

2.2. High Resolution Mass Spectra of Compound 2b

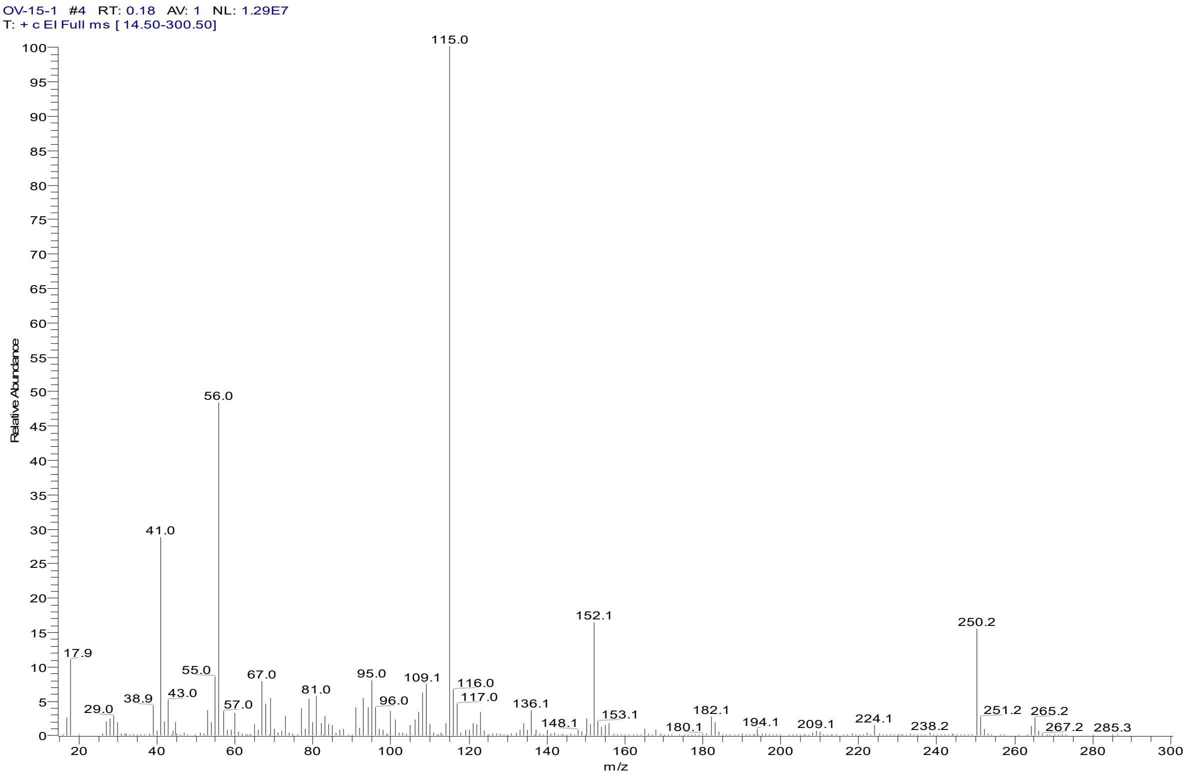

2.3. High Resolution Mass Spectra of Compound 2c

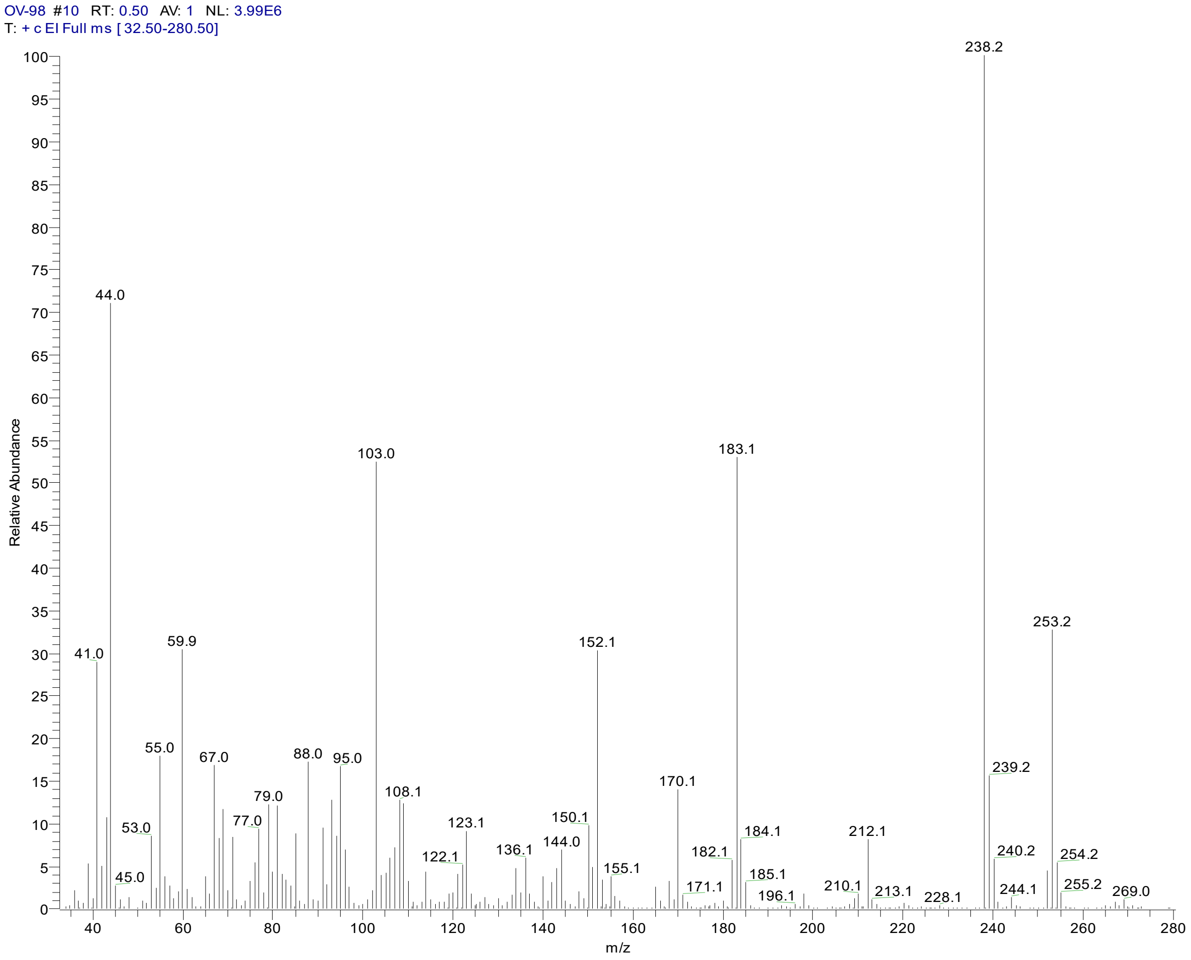

2.4. High Resolution Mass Spectra of Compound 2d

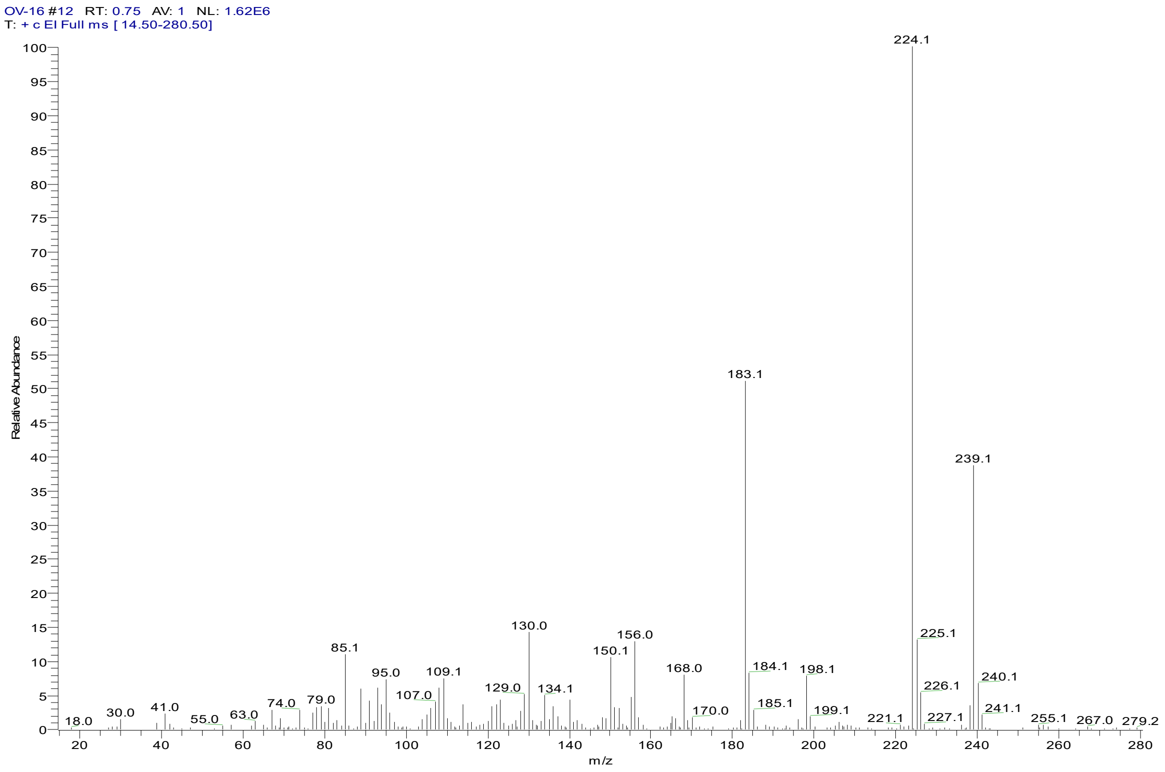

2.5. High Resolution Mass Spectra of Compound 2’a

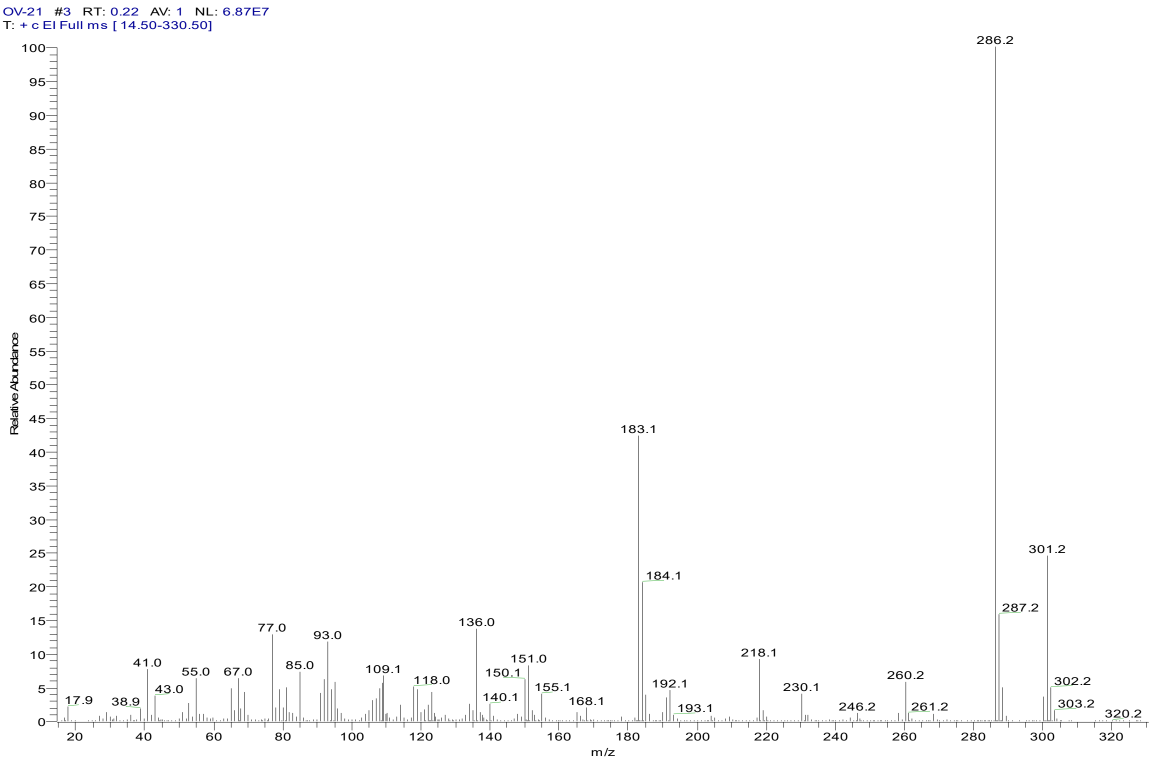

2.6. High Resolution Mass Spectra of Compound 2’b

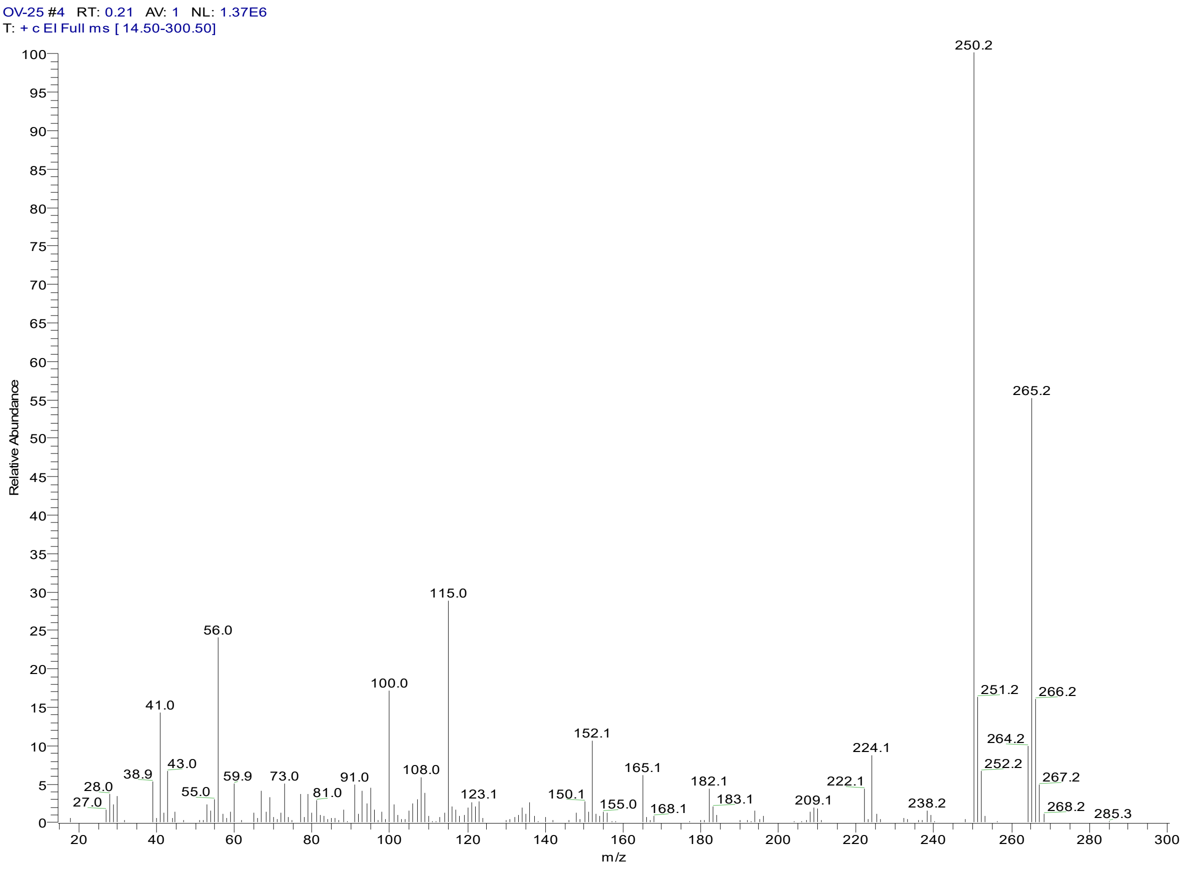

2.7. High Resolution Mass Spectra of Compound 2′c

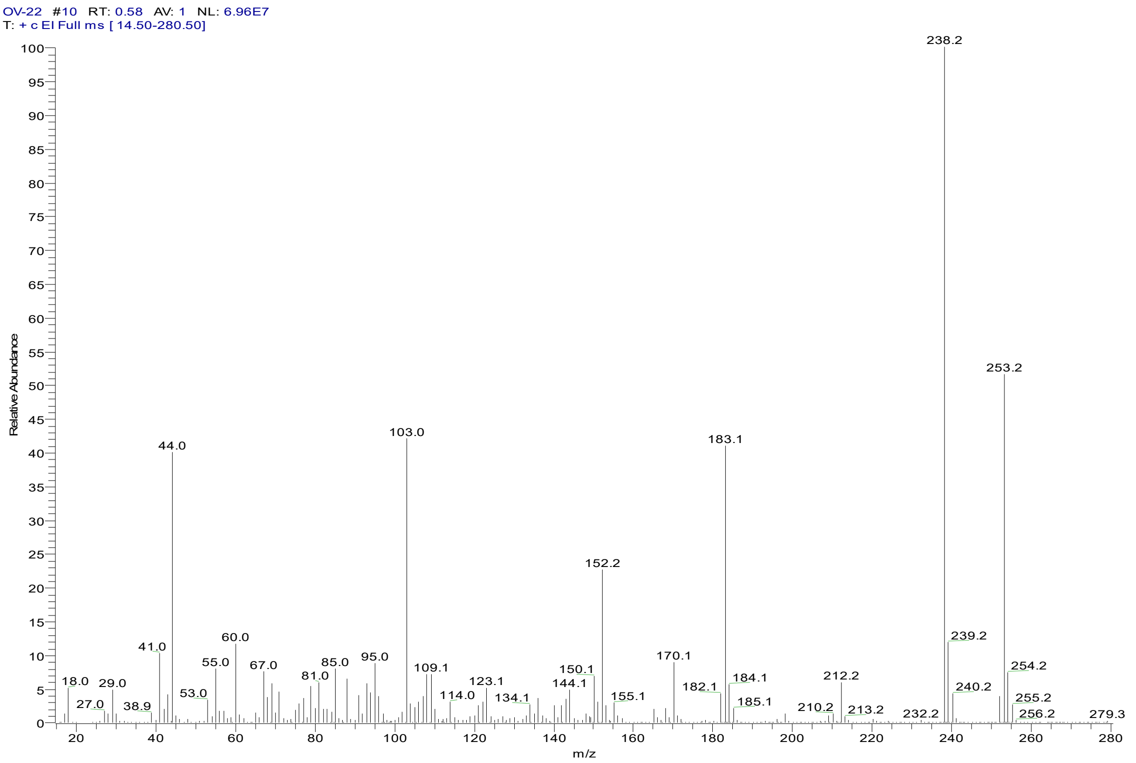

2.8. High Resolution Mass Spectra of Compound 2′d

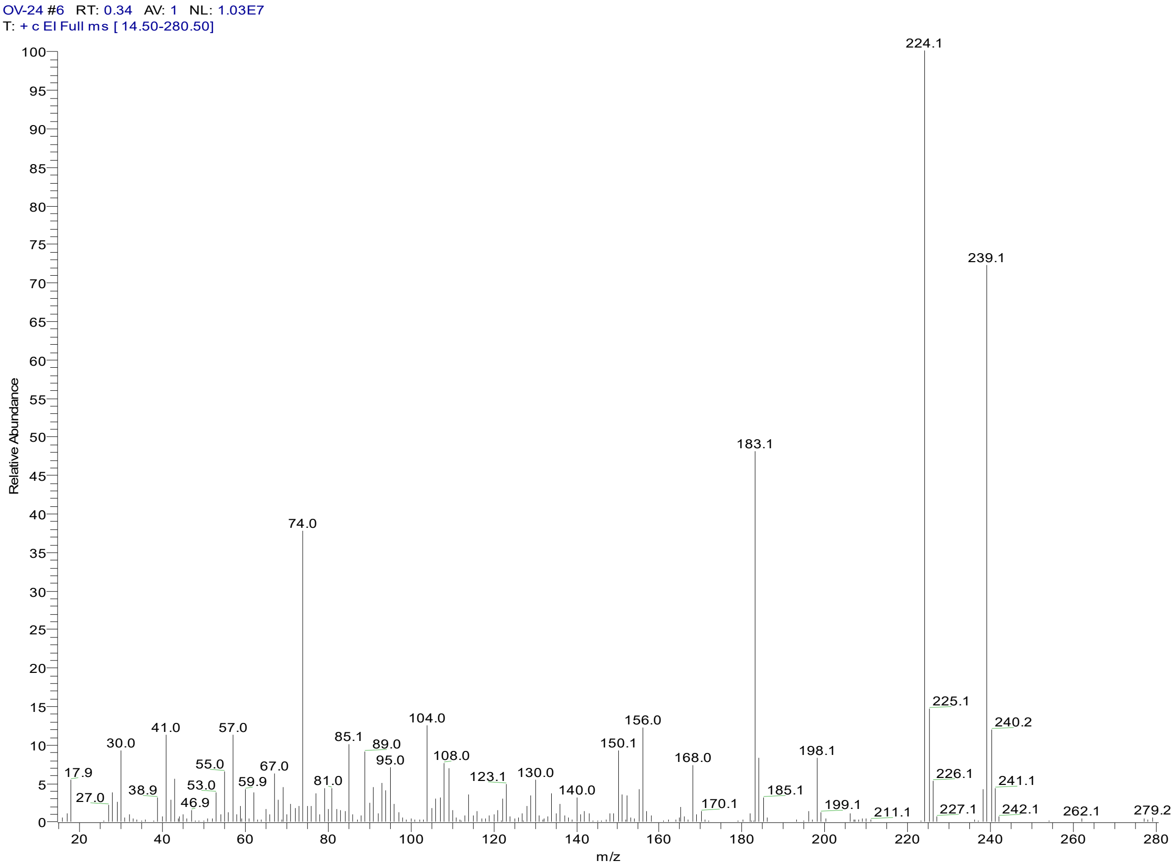

2.9. High Resolution Mass Spectra of Compound 3a

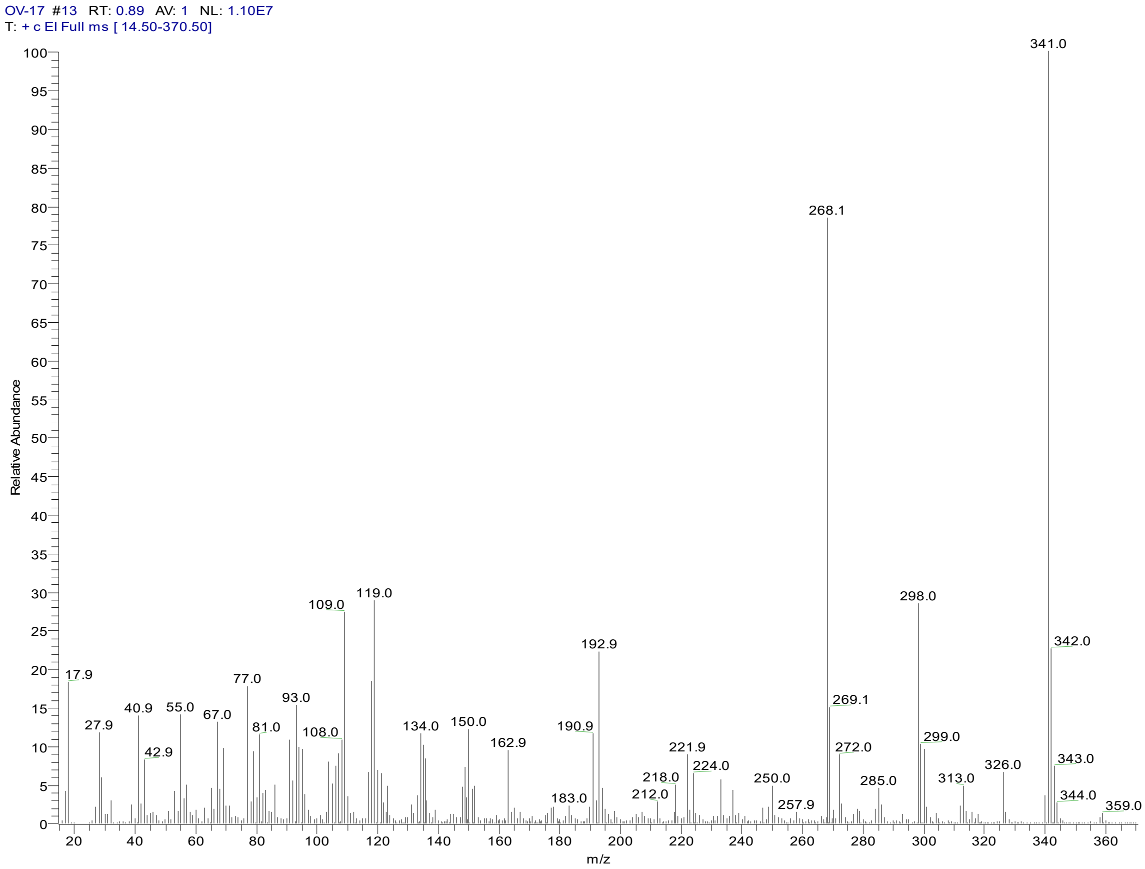

2.10. High Resolution Mass Spectra of Compound 3b

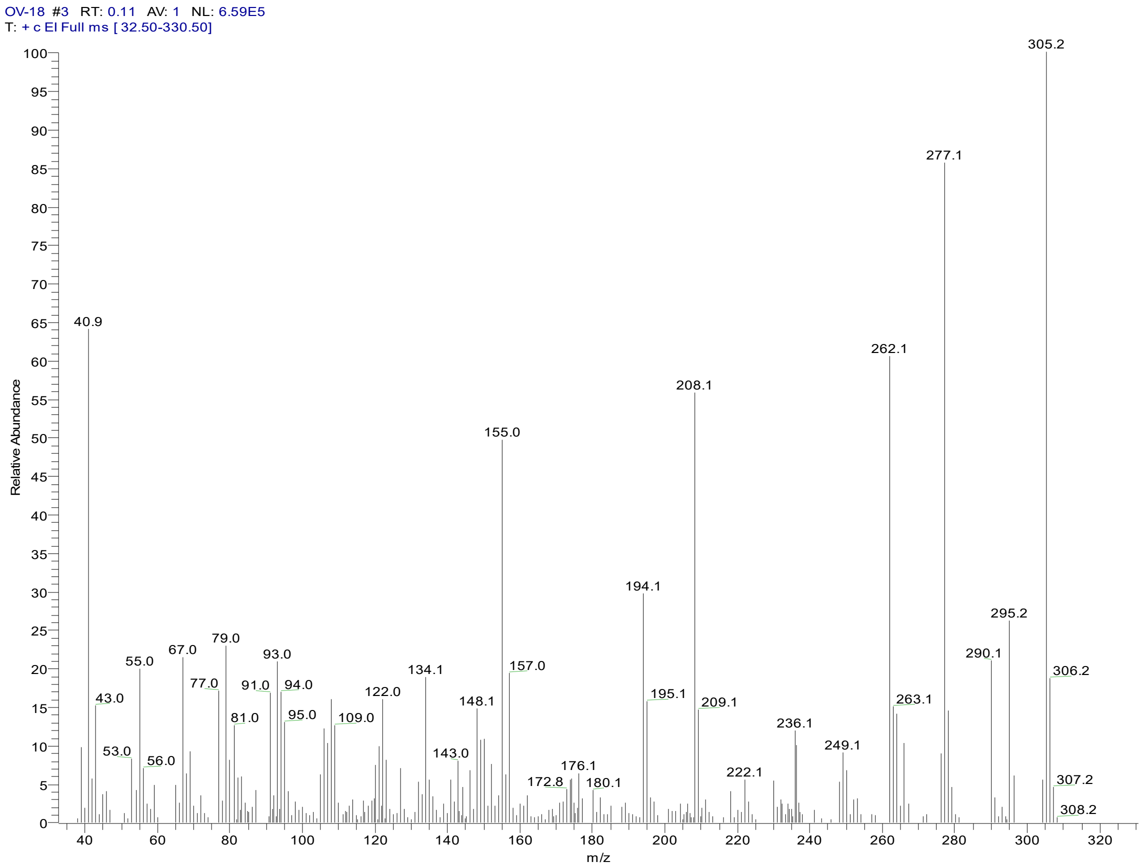

2.11. High Resolution Mass Spectra of Compound 3c

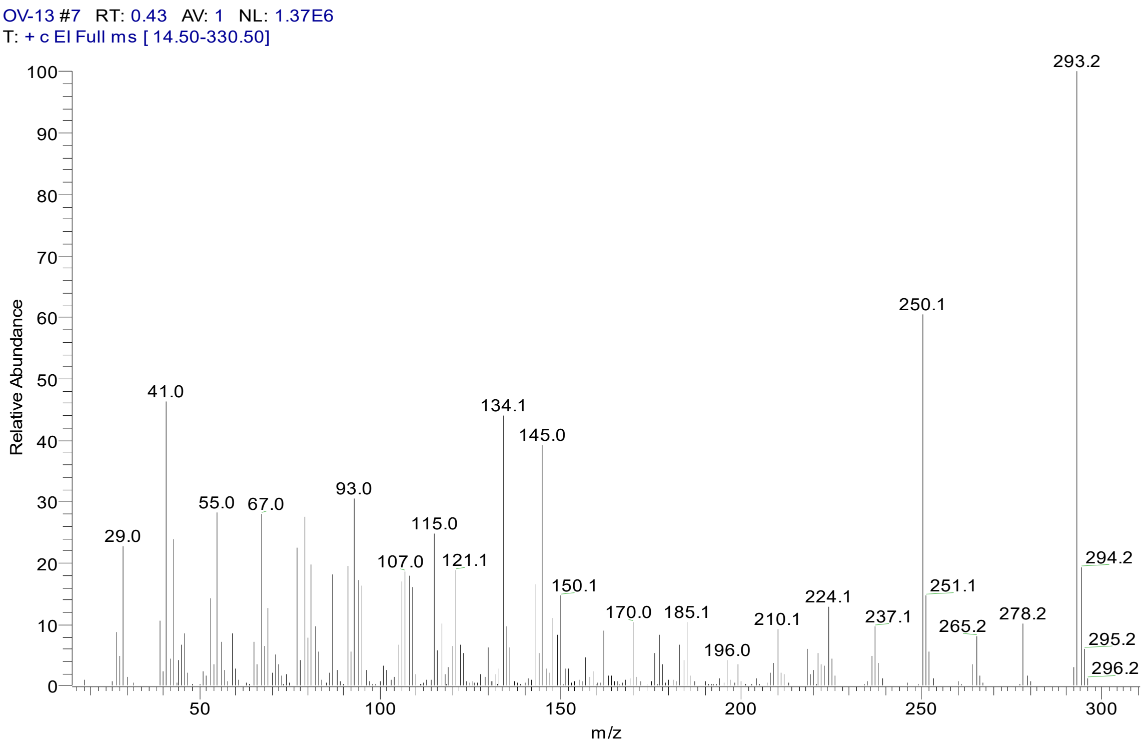

2.12. High Resolution Mass Spectra of Compound 3d

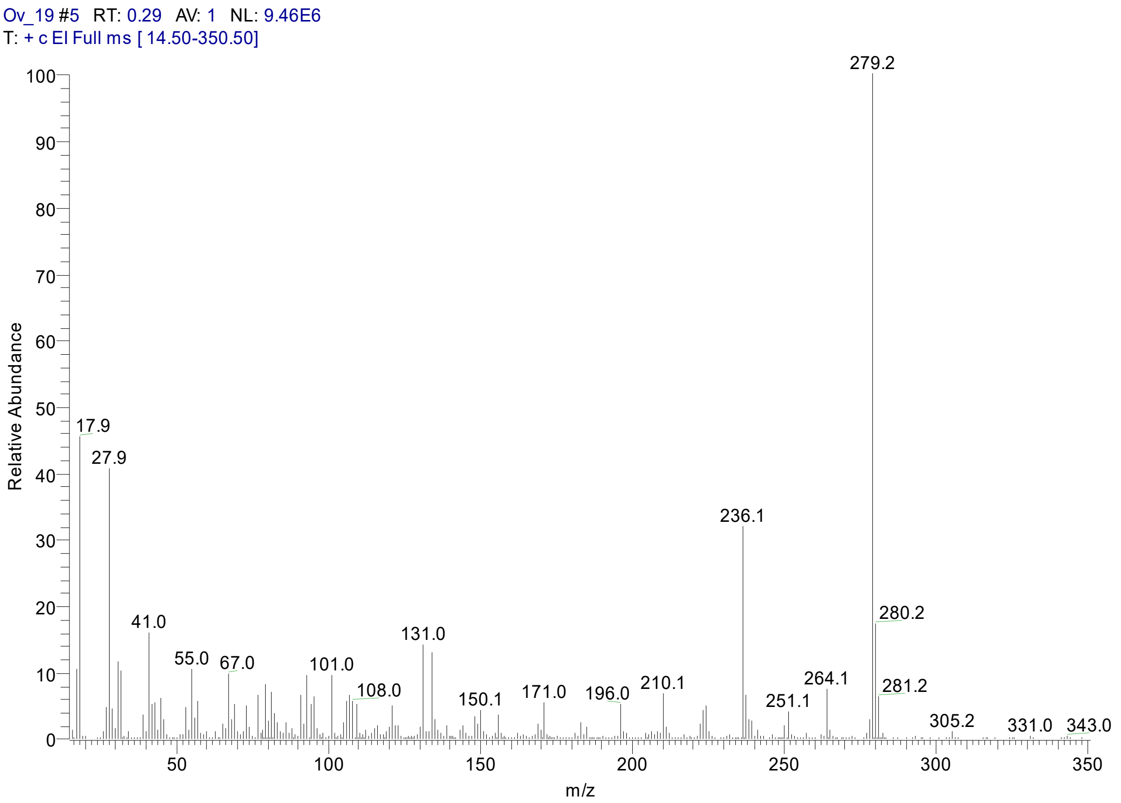

2.13. High Resolution Mass Spectra of Compound 3’a

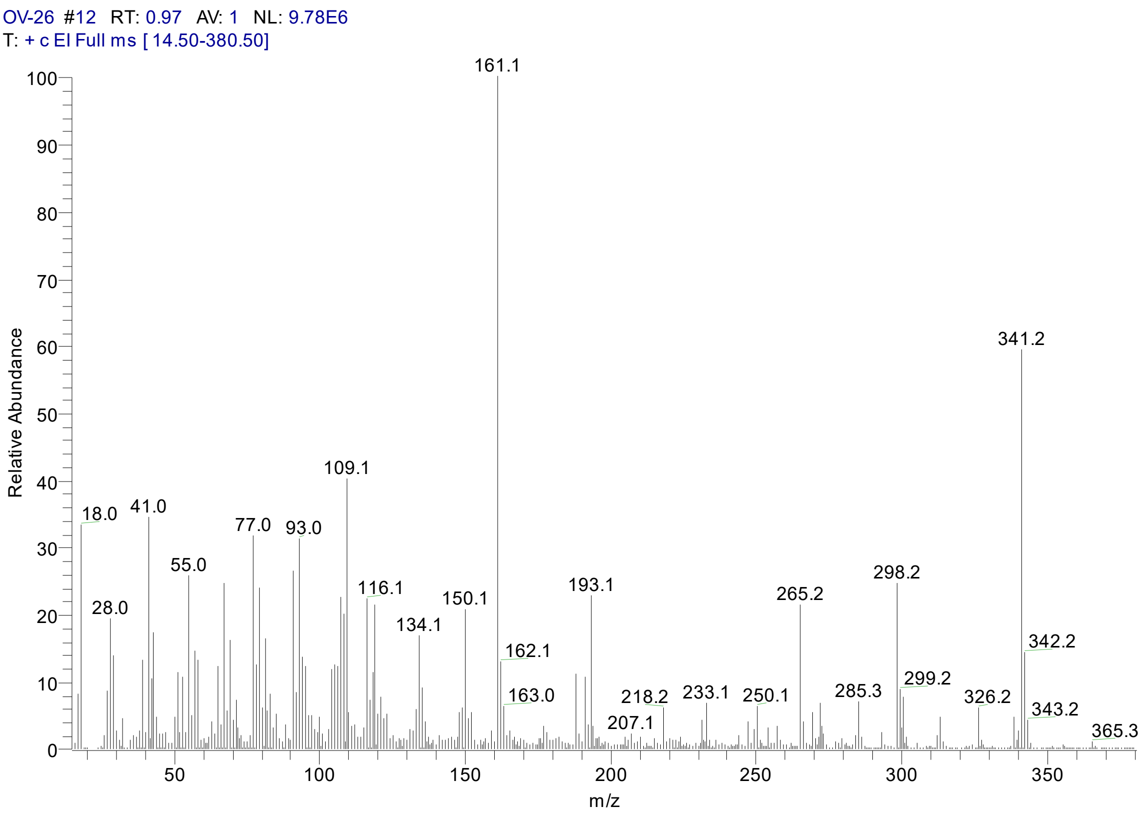

2.14. High Resolution Mass Spectra of Compound 3’b

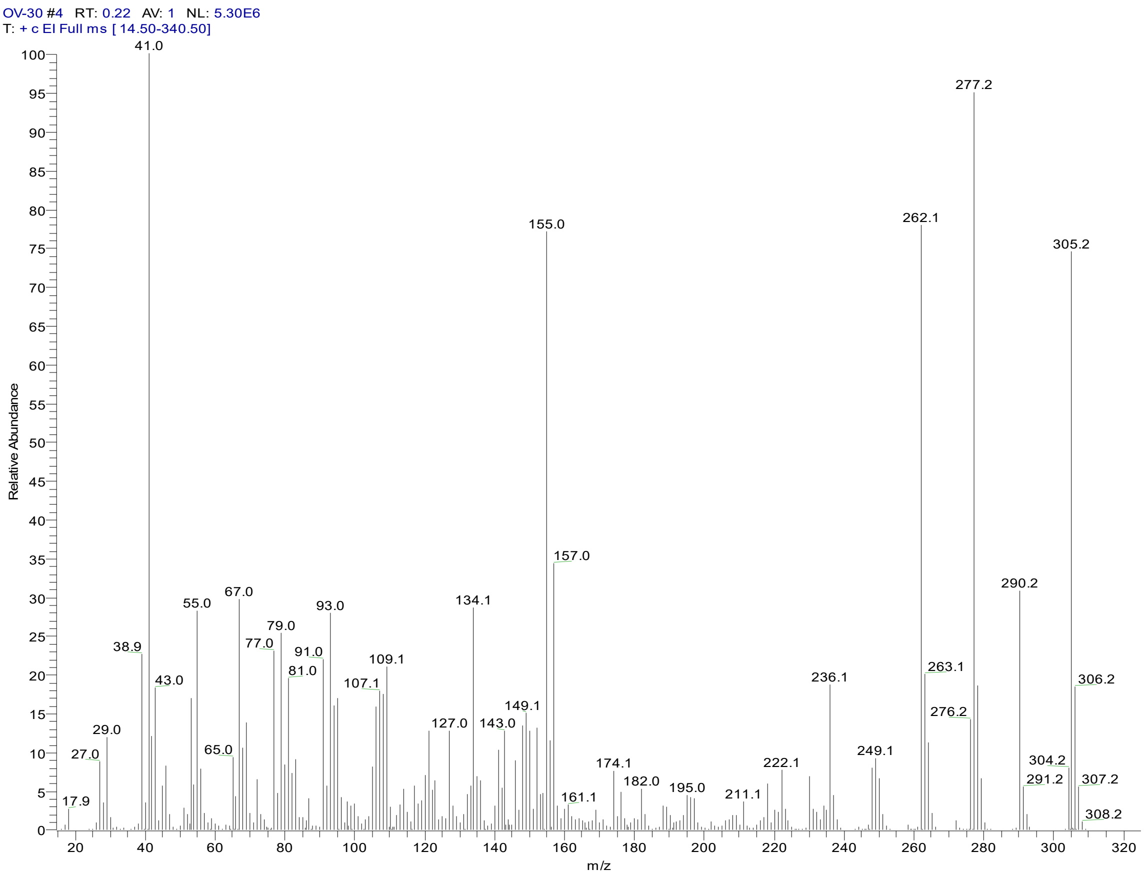

2.15. High Resolution Mass Spectra of Compound 3’c

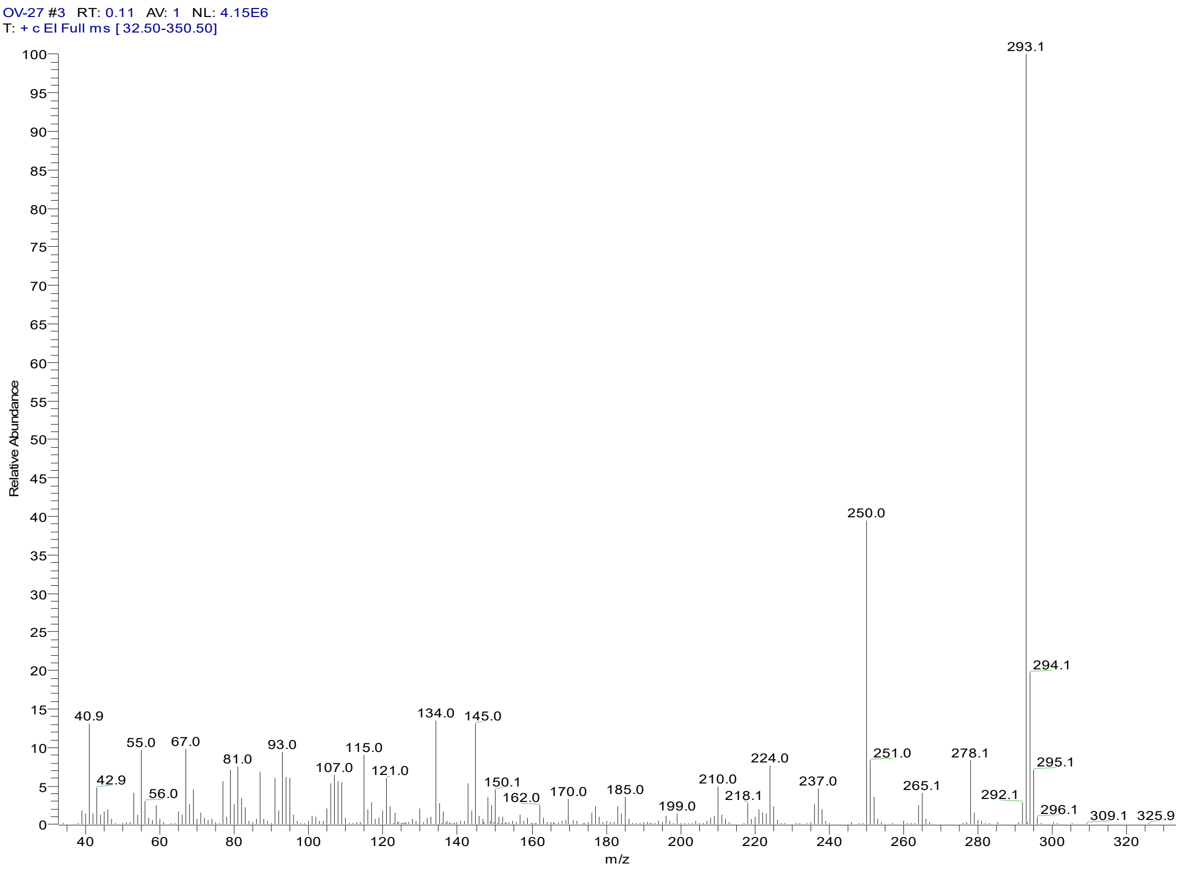

2.16. High Resolution Mass Spectra of Compound 3’d

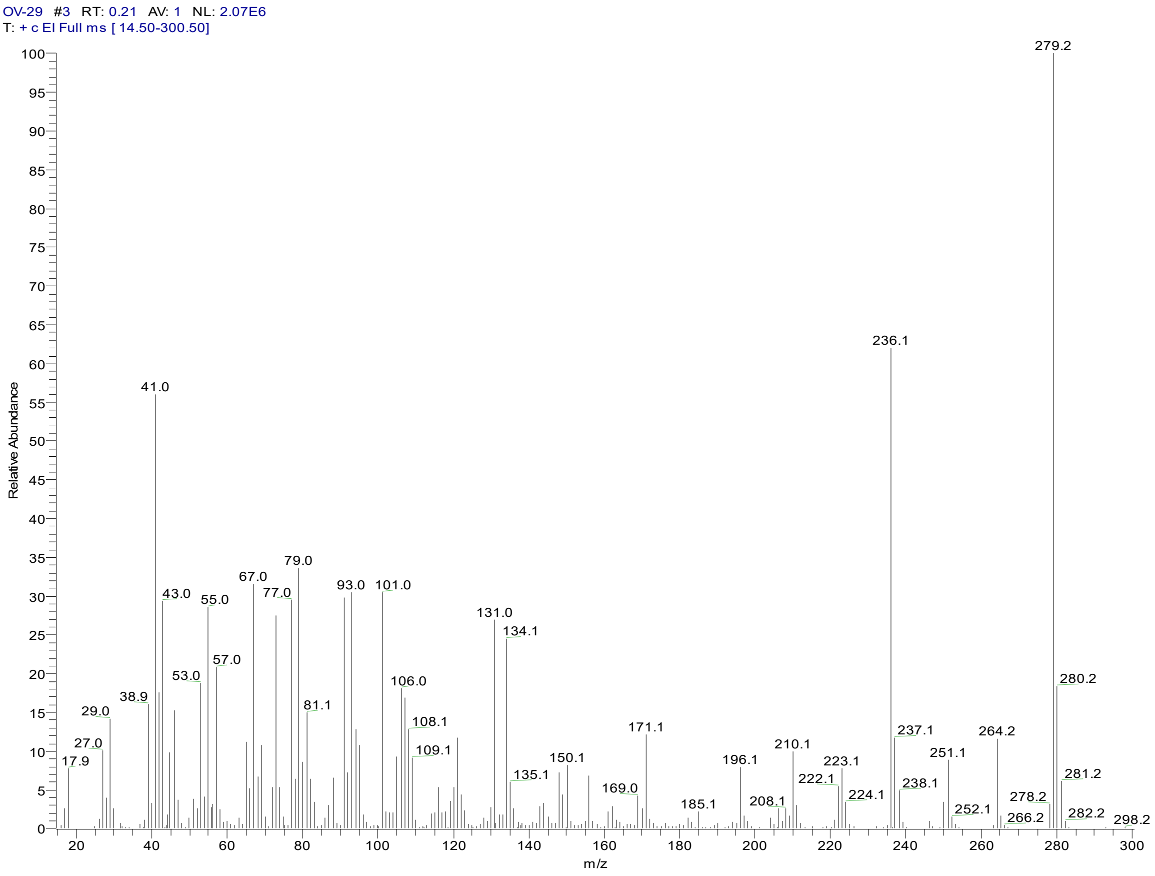

2.17. High Resolution Mass Spectra of Compound 4a

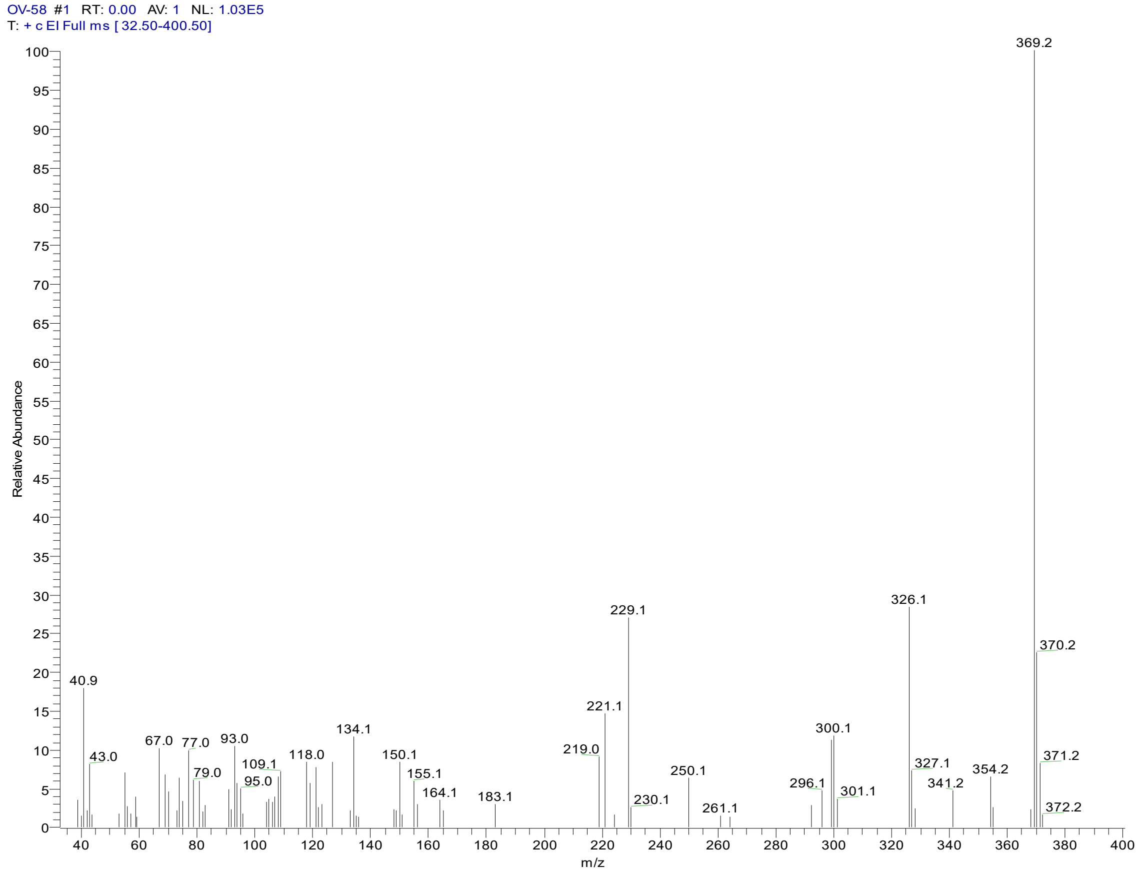

2.18. High Resolution Mass Spectra of Compound 4b

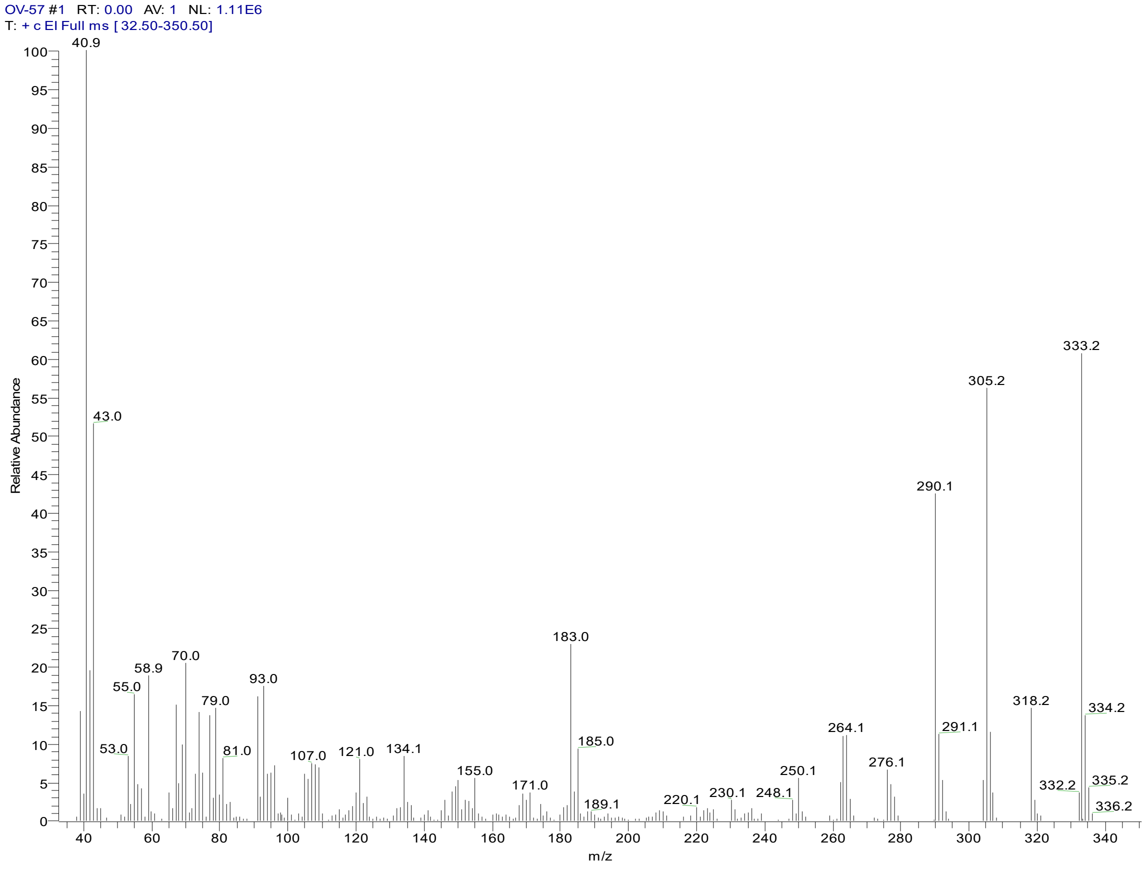

2.19. High Resolution Mass Spectra of Compound 4c

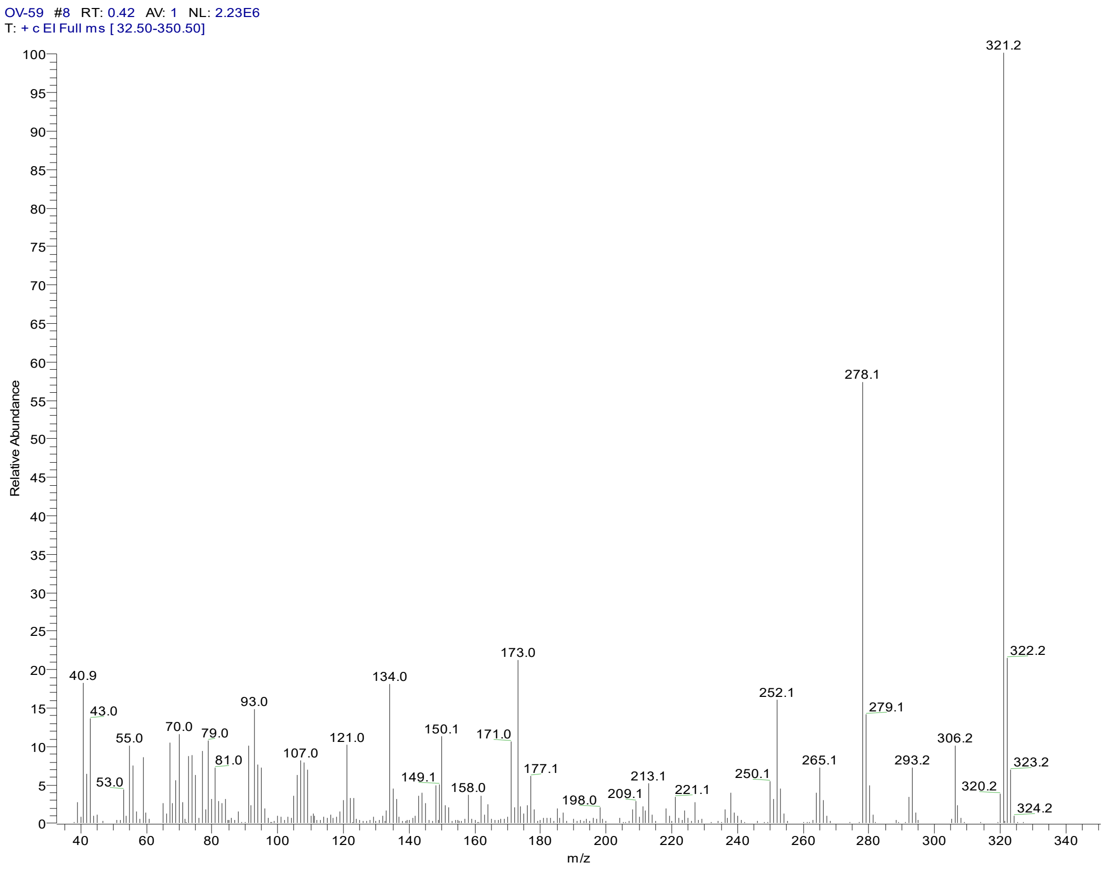

2.20. High Resolution Mass Spectra of Compound 4d

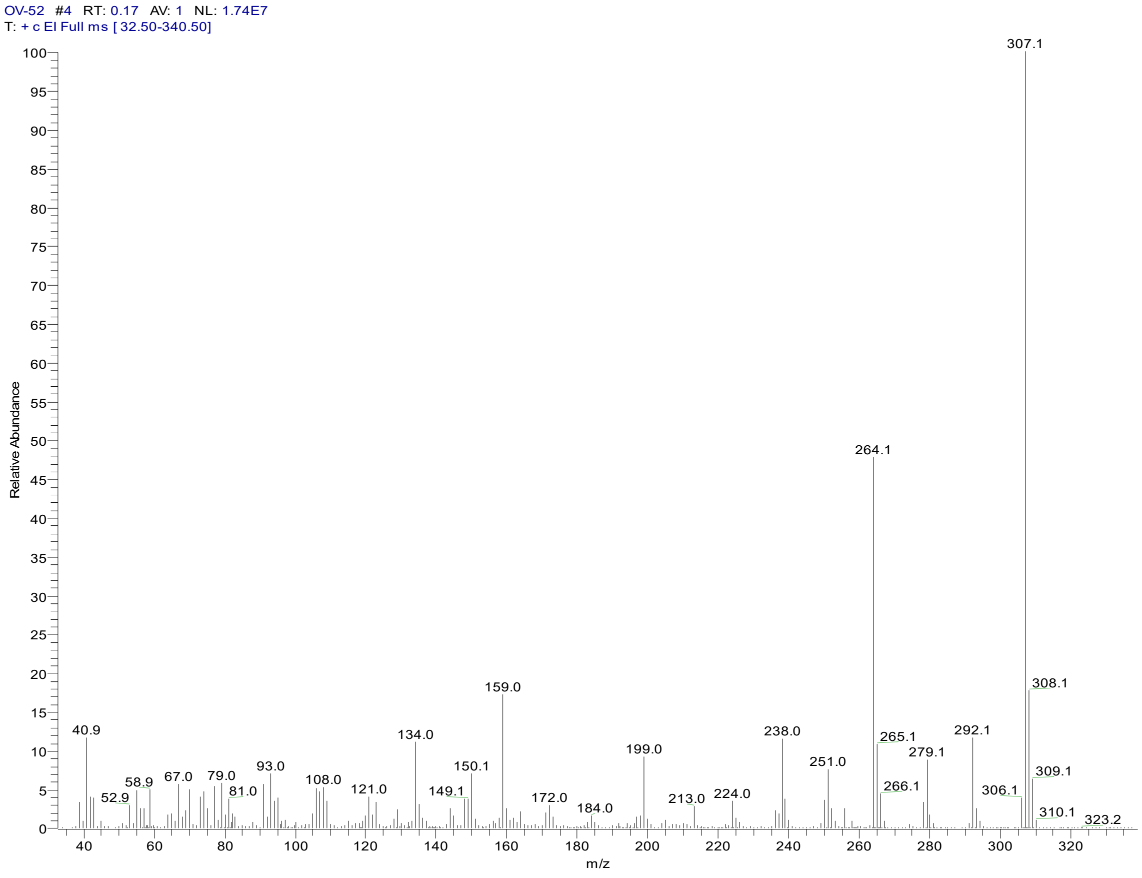

2.21. High Resolution Mass Spectra of Compound 5a

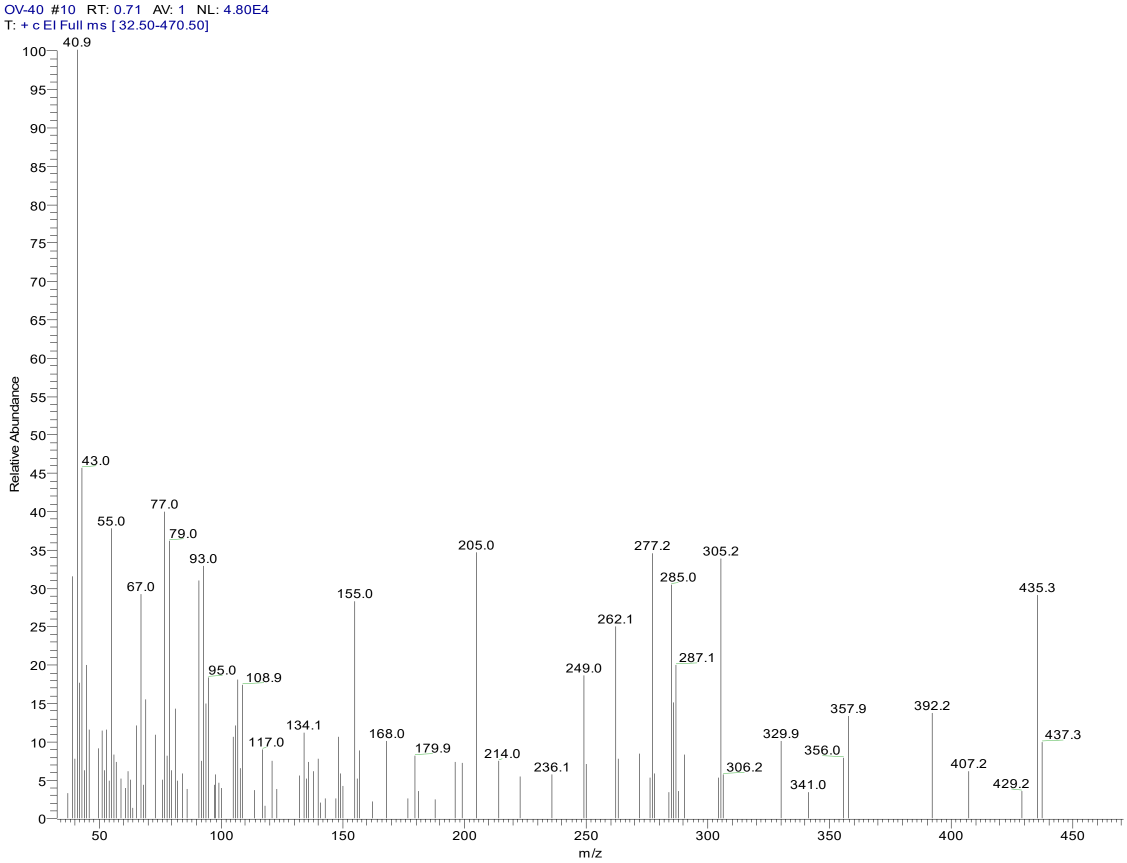

2.22. High Resolution Mass Spectra of Compound 5b

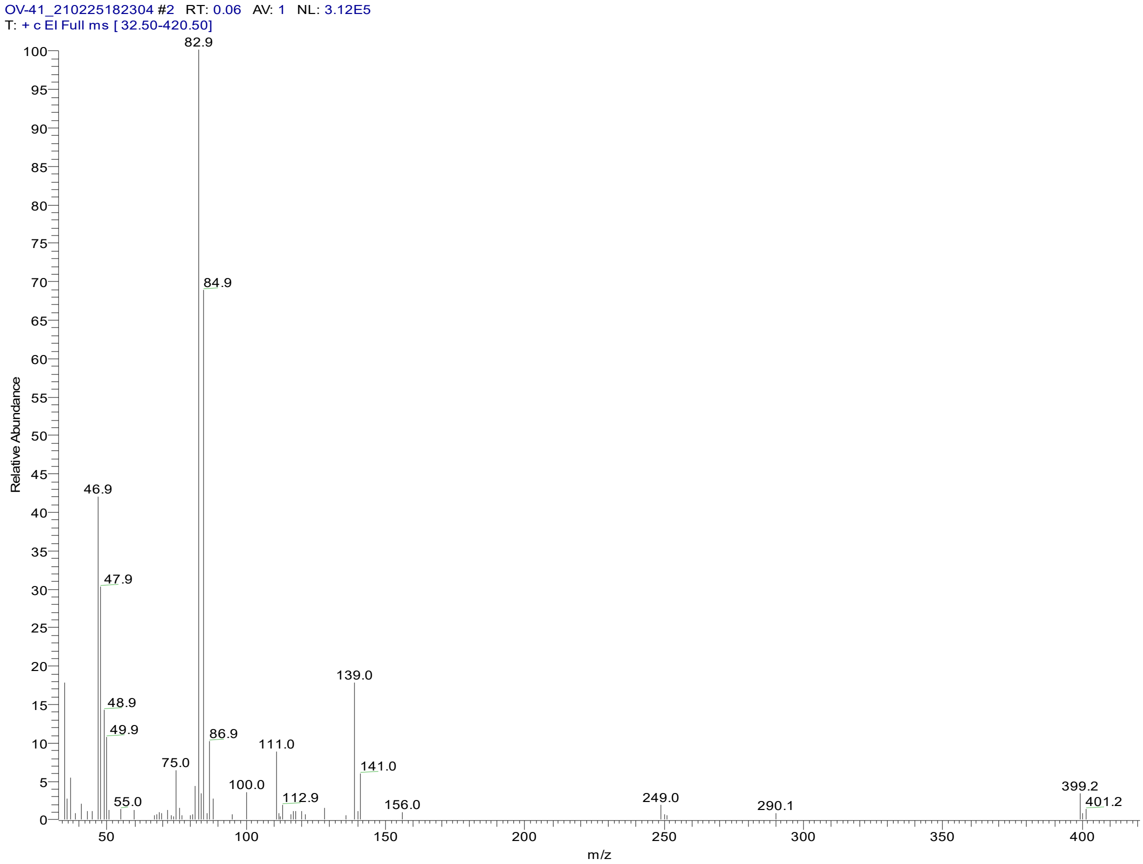

2.23. High Resolution Mass Spectra of Compound 5c

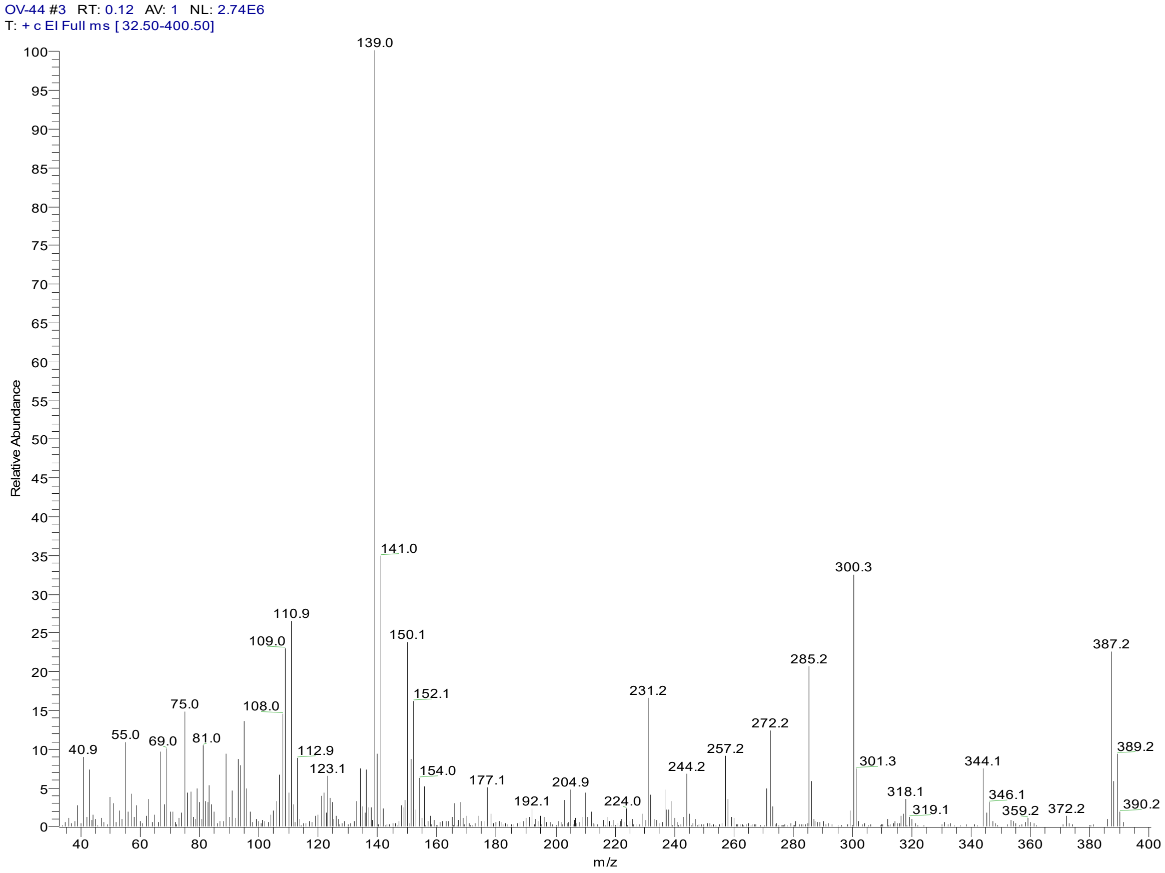

2.24. High Resolution Mass Spectra of Compound 5d

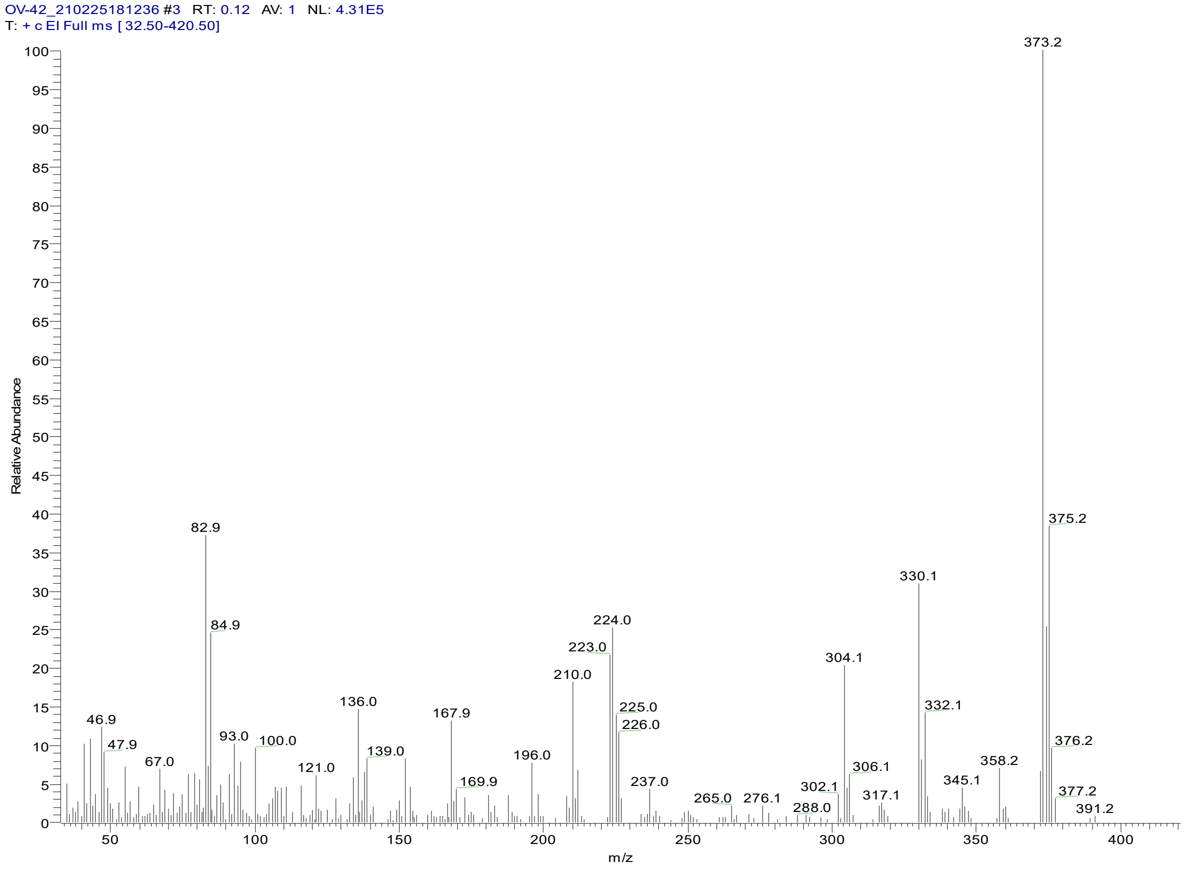

2.25. High Resolution Mass Spectra of Compound 6a

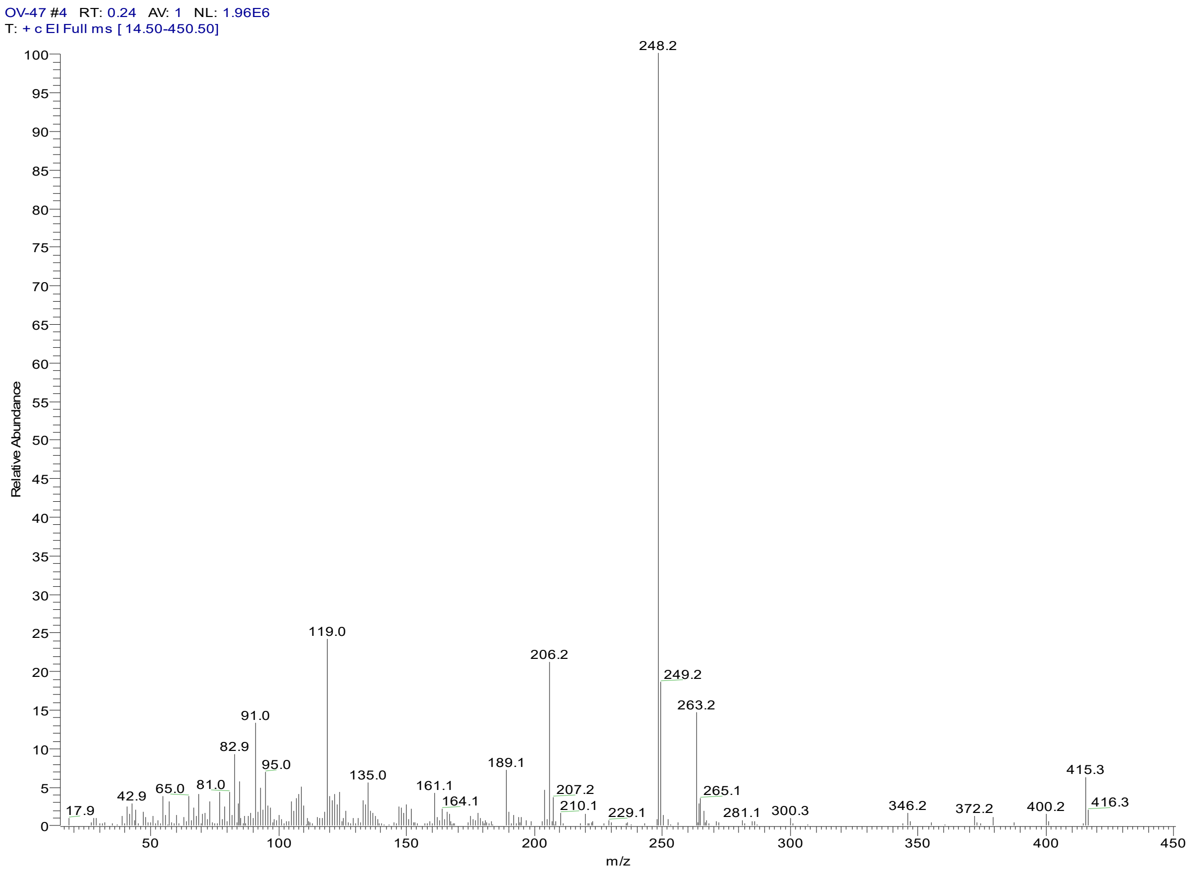

2.26. High Resolution Mass Spectra of Compound 6b

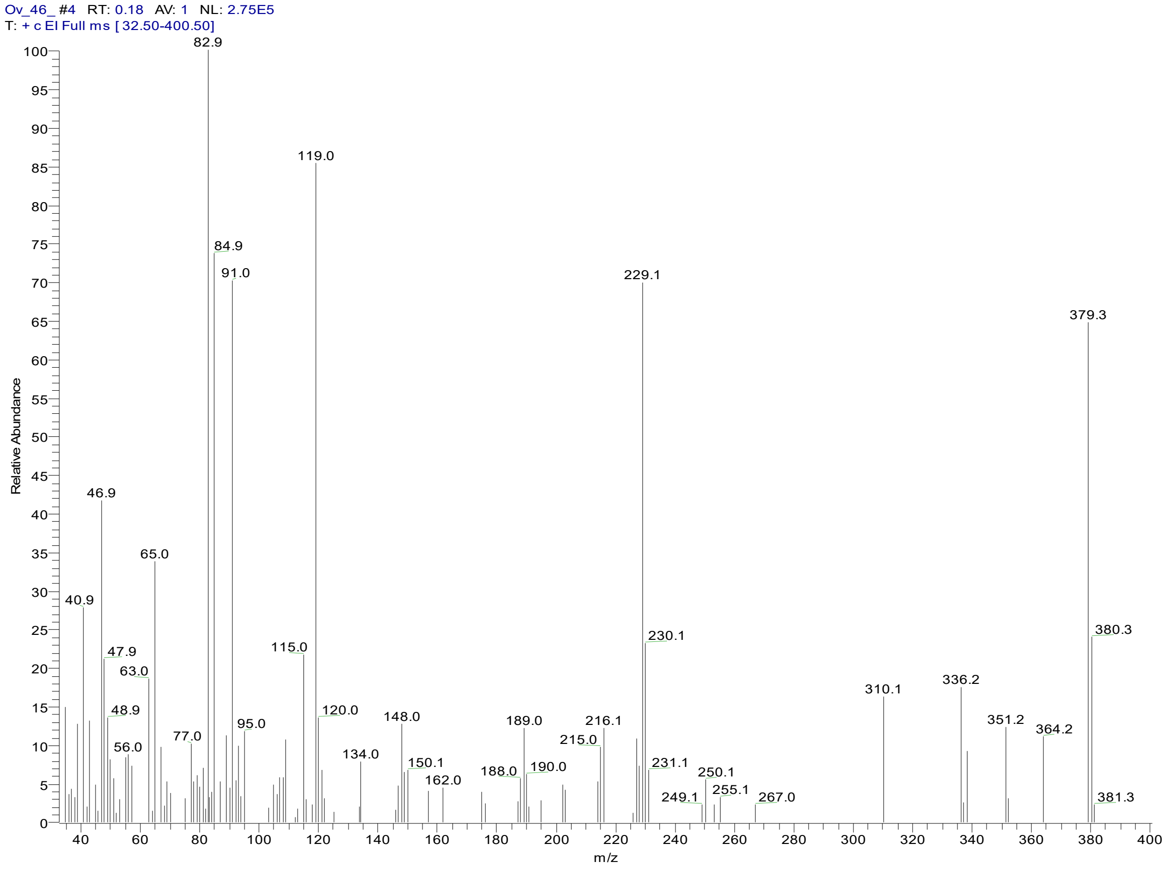

2.27. High Resolution Mass Spectra of Compound 6c

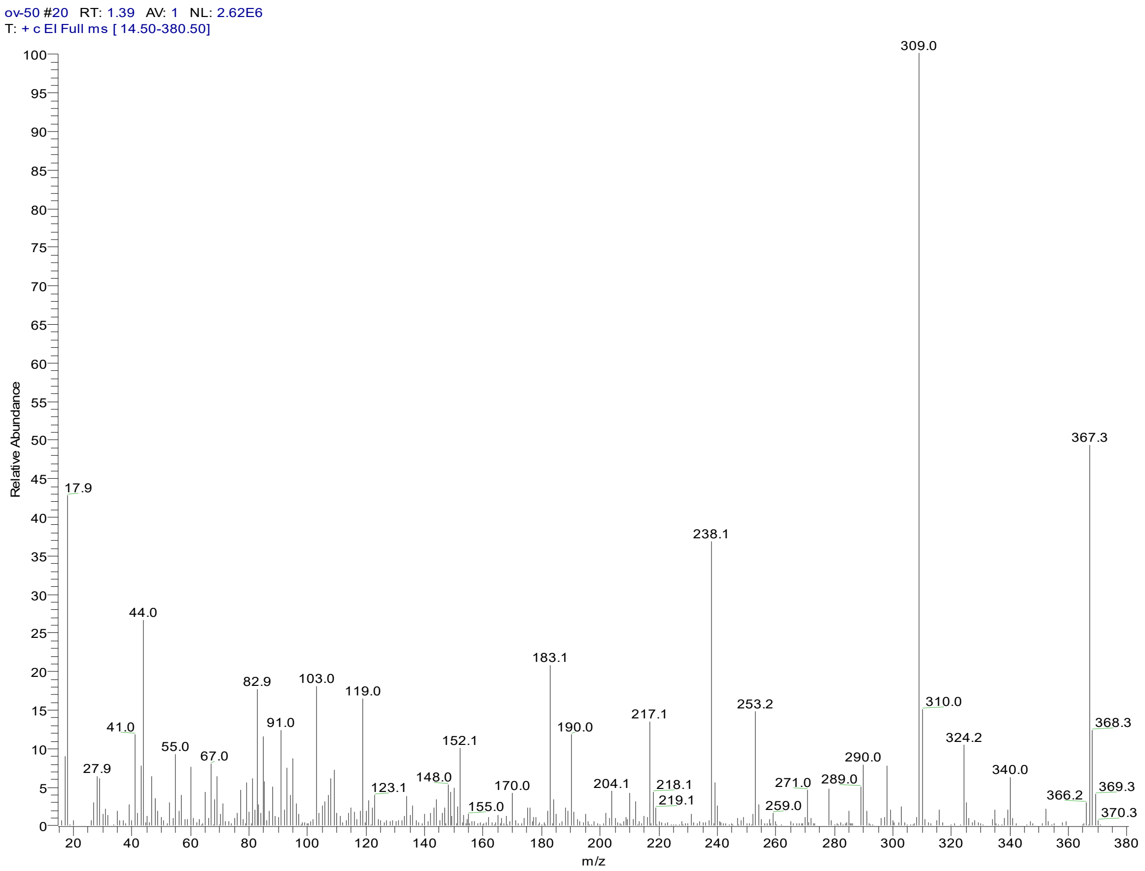

2.28. High Resolution Mass Spectra of Compound 6d

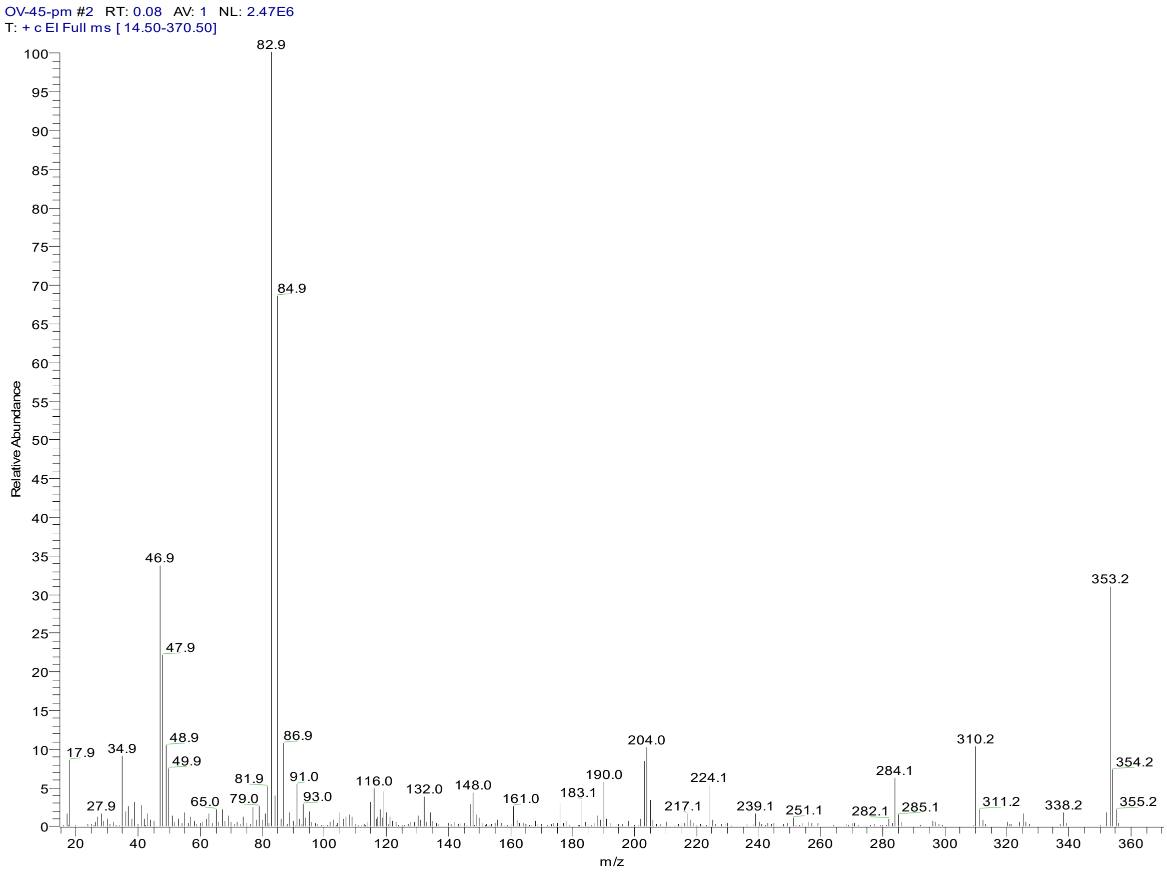

2.29. High Resolution Mass Spectra of Compound 7a

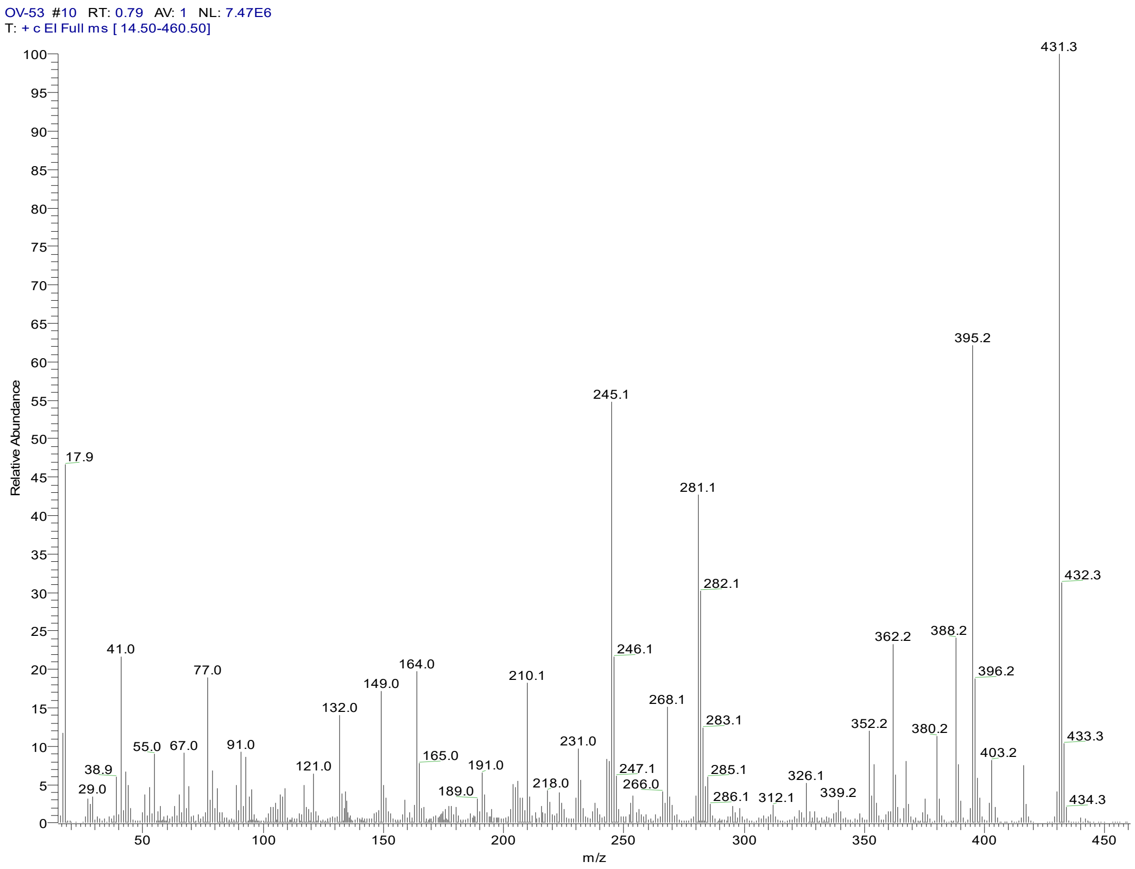

2.30. High Resolution Mass Spectra of Compound 7b

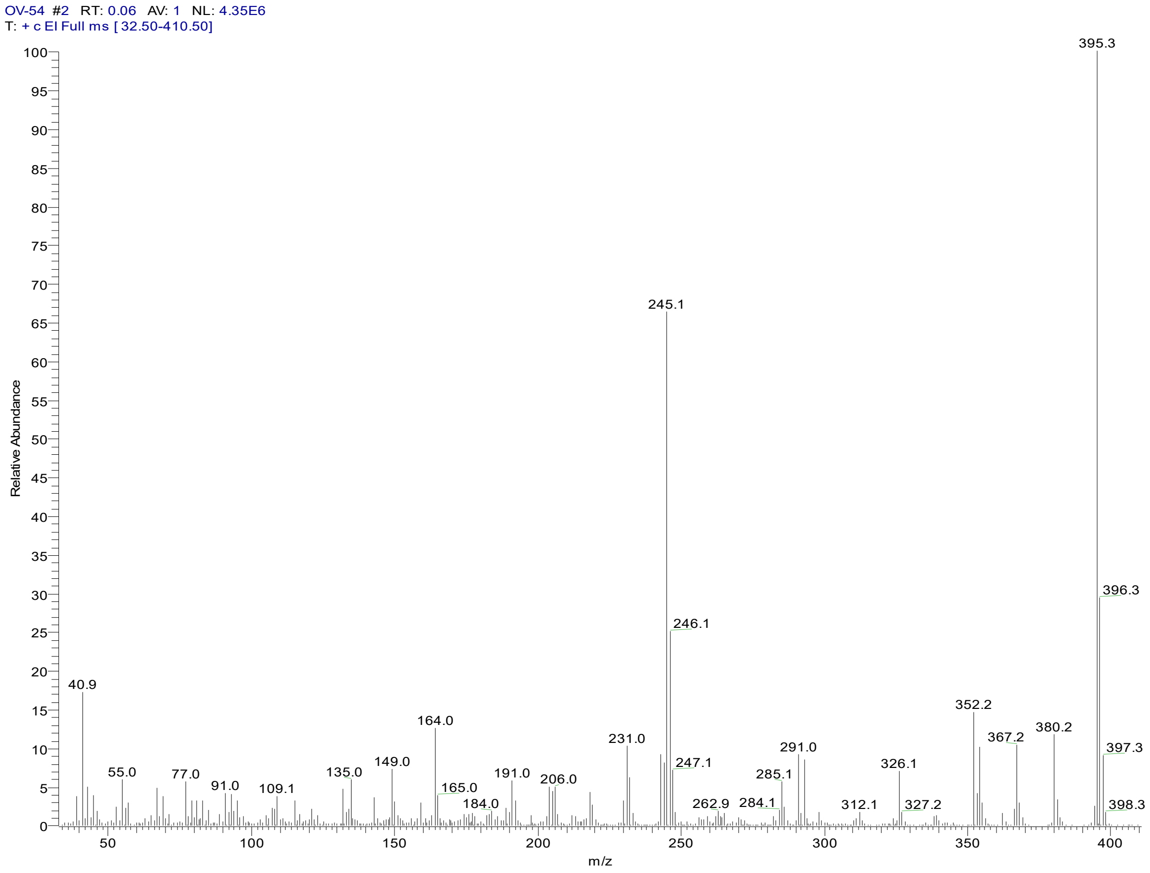

2.31. High Resolution Mass Spectra of Compound 7c

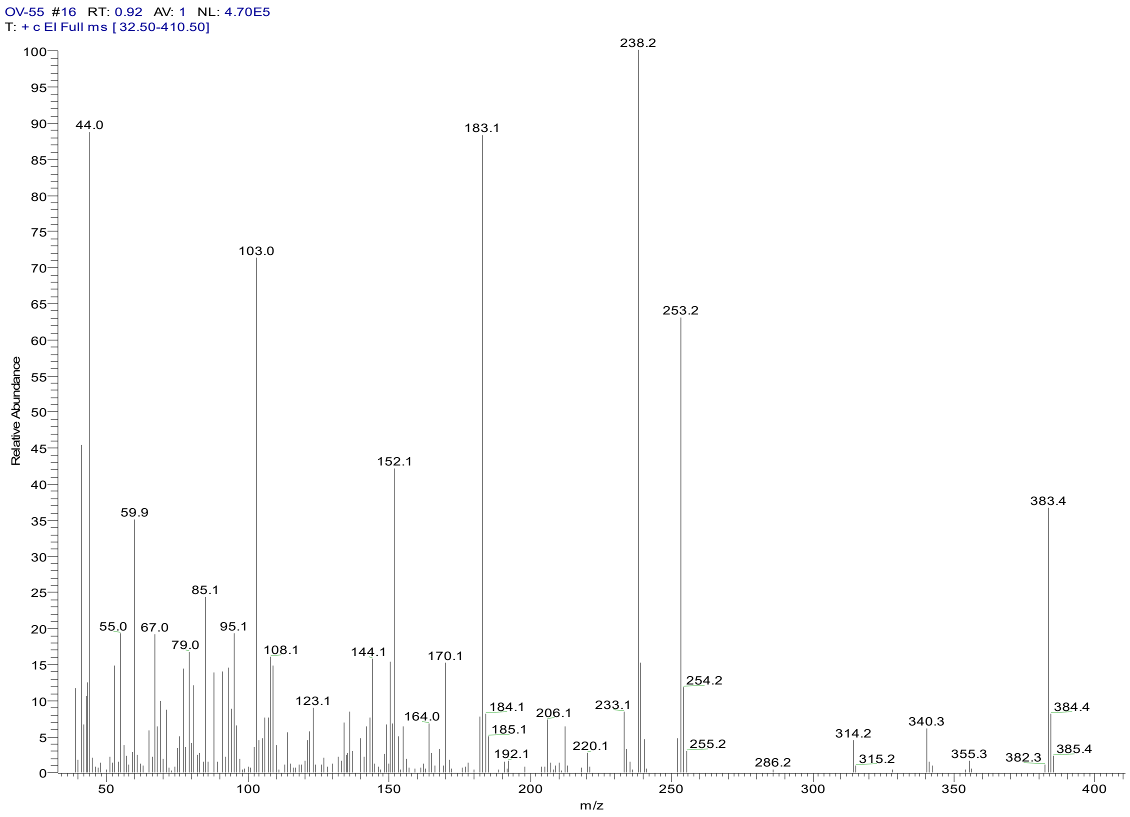

2.32. High Resolution Mass Spectra of Compound 7d

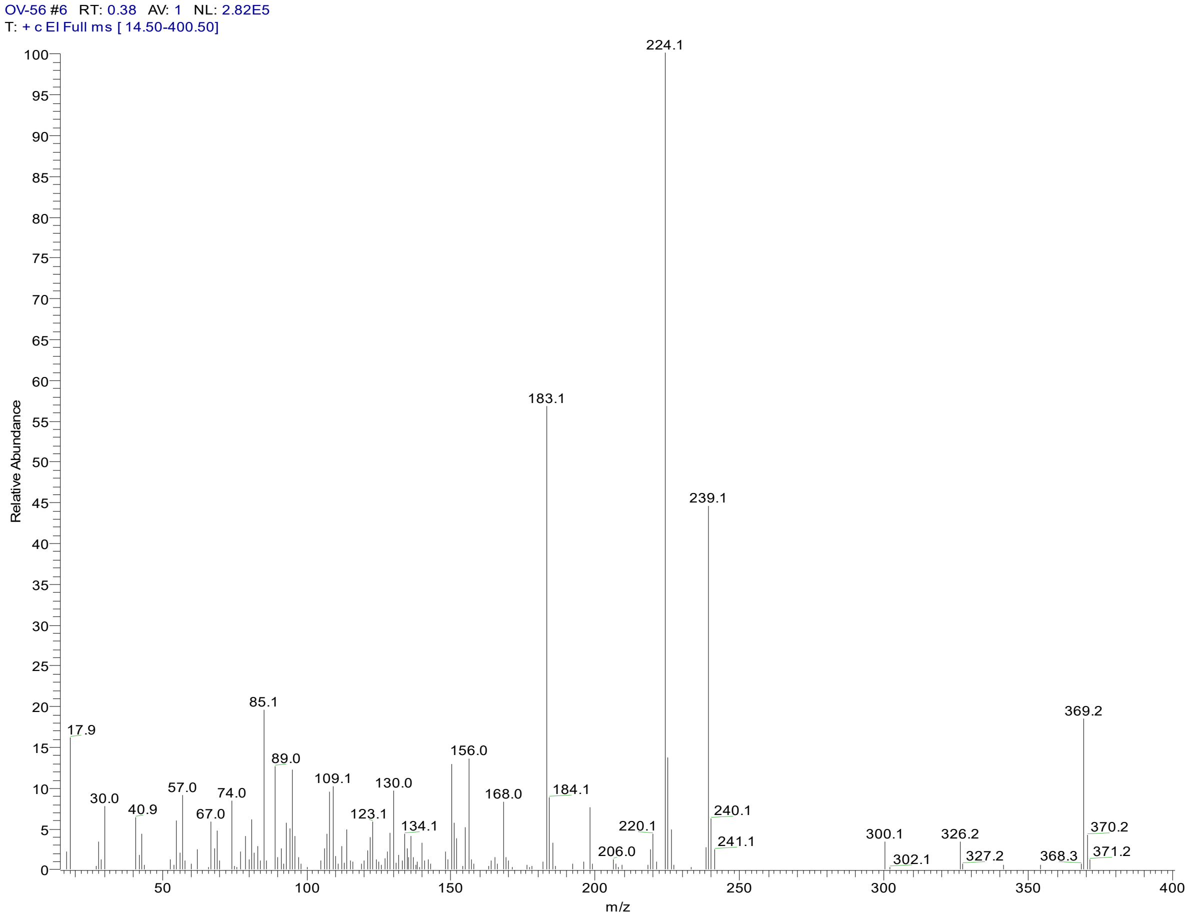

2.33. High Resolution Mass Spectra of Compound 8a

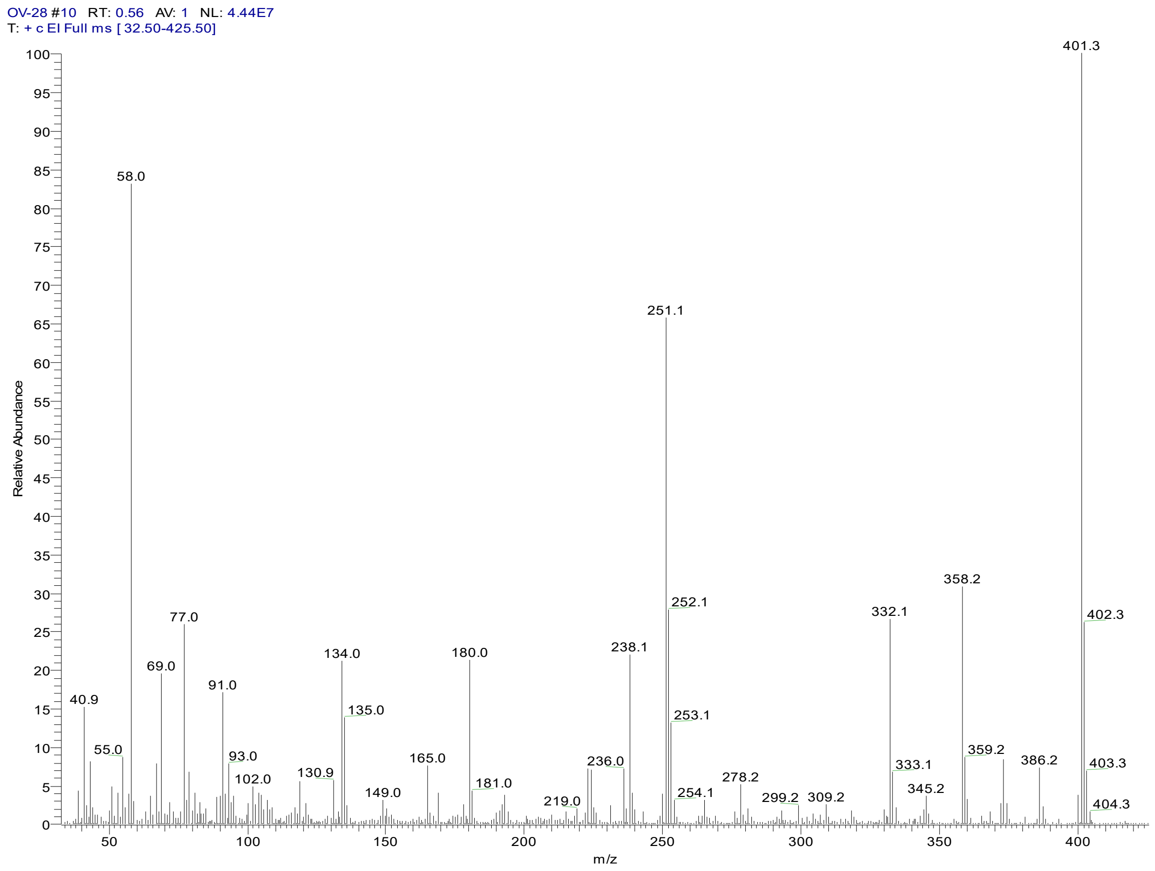

2.34. High Resolution Mass Spectra of Compound 8b

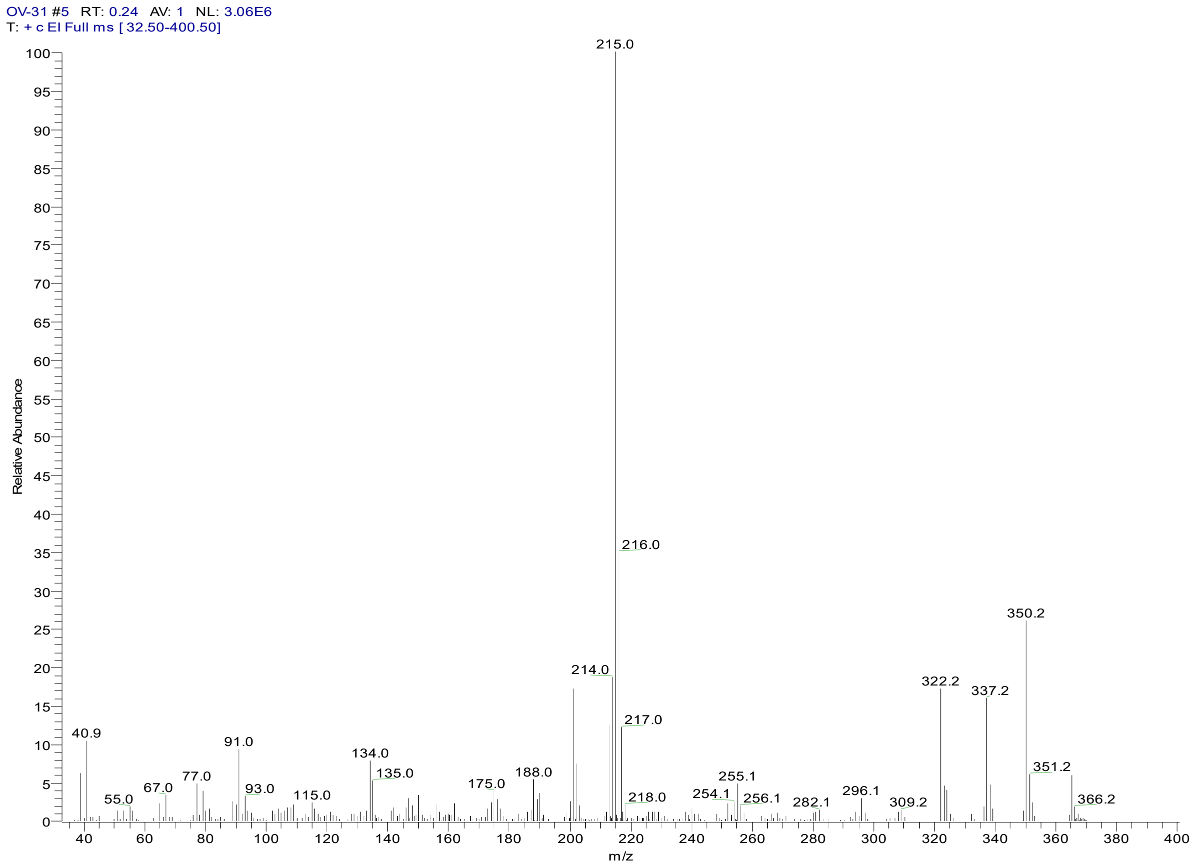

2.35. High Resolution Mass Spectra of Compound 8c

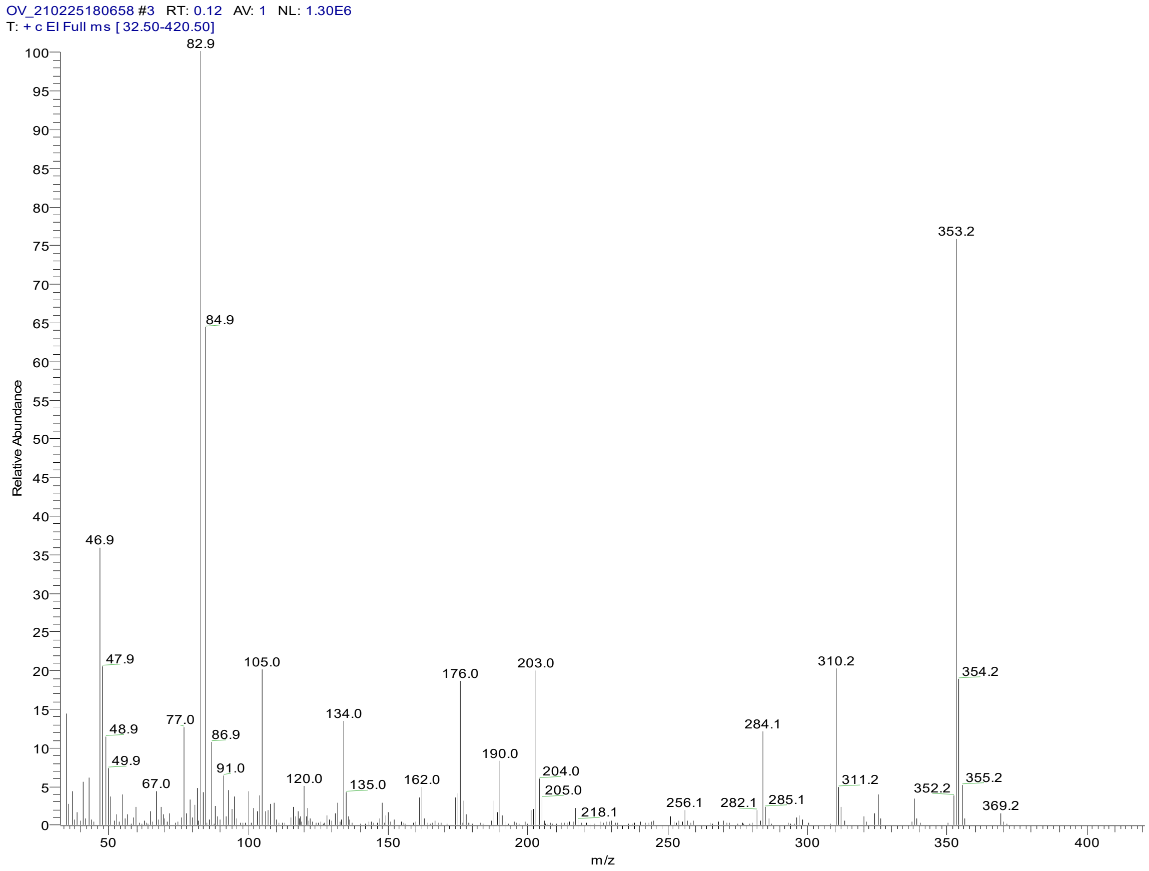

2.36. High Resolution Mass Spectra of Compound 8d

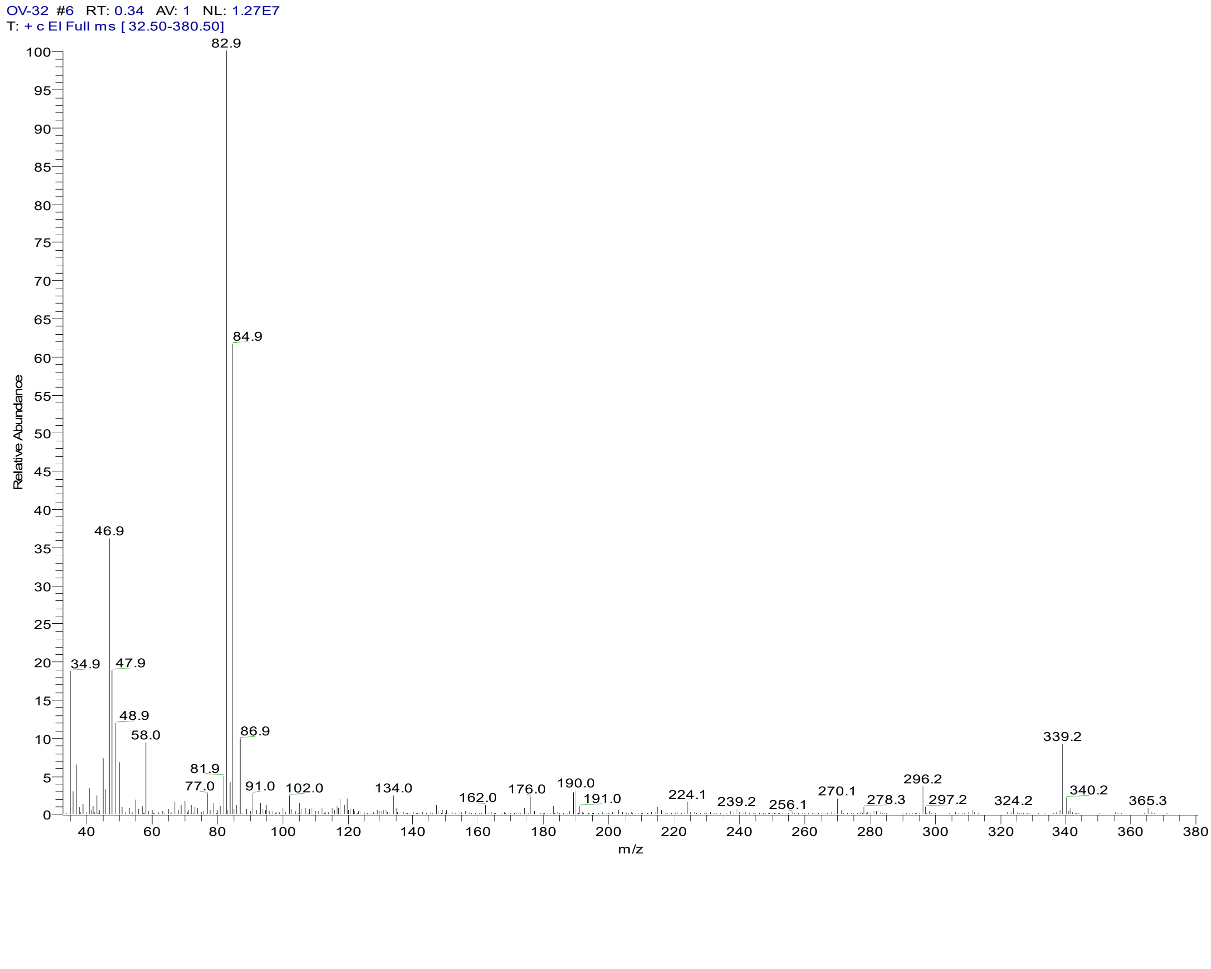

Supplement: Supplementary file 1 [file molecules-27-04761-s001.zip › molecules-1812649-supplementary.pdf]
